# Introducing Savie: A Biodegradable Surfactant Enabling Chemo- and Biocatalysis and Related Reactions in Recyclable Water

**DOI:** 10.1021/jacs.2c13444

**Published:** 2023-02-08

**Authors:** Joseph R. A. Kincaid, Madison J. Wong, Nnamdi Akporji, Fabrice Gallou, David M. Fialho, Bruce H. Lipshutz

**Affiliations:** † Department of Chemistry and Biochemistry, University of California, Santa Barbara, California 93106, United States; ‡ Novartis Pharma AG, CH-4057 Basel, Switzerland

## Abstract

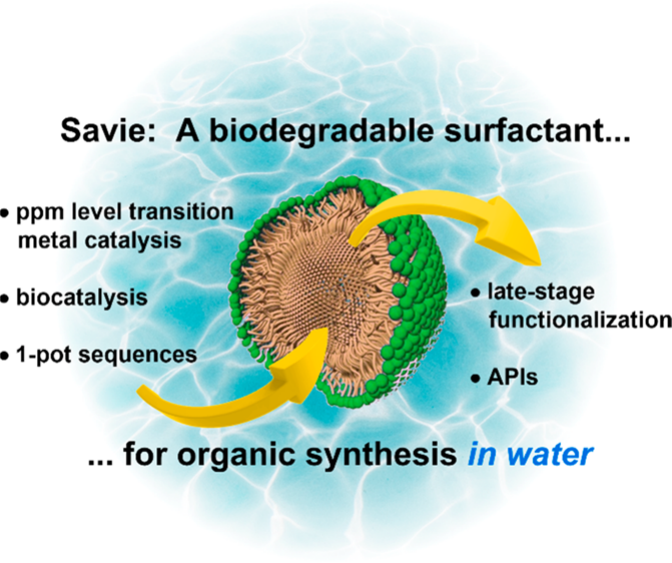

Savie is a biodegradable surfactant derived from vitamin E and polysarcosine (PSar) developed for use in organic synthesis in recyclable water. This includes homogeneous catalysis (including examples employing only ppm levels of catalyst), heterogeneous catalysis, and biocatalytic transformations, including a multistep chemoenzymatic sequence. Use of Savie frequently leads to significantly higher yields than do conventional surfactants, while obviating the need for waste-generating organic solvents.

## Introduction

### Nonionic Surfactants in Organic Synthesis

Organic solvents have long been the medium in which organic synthesis is performed and thus account for half of all waste generated by the pharmaceutical industry.^1^ As the bar is continuously raised on the environmental friendliness of industrial processes, and safety concerns inherent to many organic solvents (e.g., flammability, toxicity, teratogenicity, carcinogenicity) come increasingly into focus while governmental regulations on their use increase in number,^2^ the demand for alternative reaction media has increased. Over the last 20 years, this has led to the development of several options, such as fluorous media,^3^ ionic liquids,^4^ and supercritical CO_2_.^5^ However, the greenest replacement for organic solvents is nature’s chosen medium: water. Water is nonflammable, nontoxic, inexpensive, and ubiquitous, and while its presence in reactions has long been considered the bane of organic synthesis, owing to issues of substrate insolubility and reagent moisture sensitivity, both issues can be obviated by the inclusion of a small amount (typically 2 wt %) of specially designed nonionic surfactants. When placed in water, these amphiphiles self-assemble into nanometer-scale micelles containing a lipophilic core, into which lipophilic substrates and catalysts can be solubilized and protected from interactions with the surrounding water. This allows otherwise water-sensitive^6^ and insoluble compounds to be dispersed within a bulk aqueous medium. Another virtue associated with micellar catalysis^7^ is that the close proximity of reagents and catalysts within the micellar core leads to characteristically high concentrations, and thereby faster reaction rates.^8^

**Figure 1:**
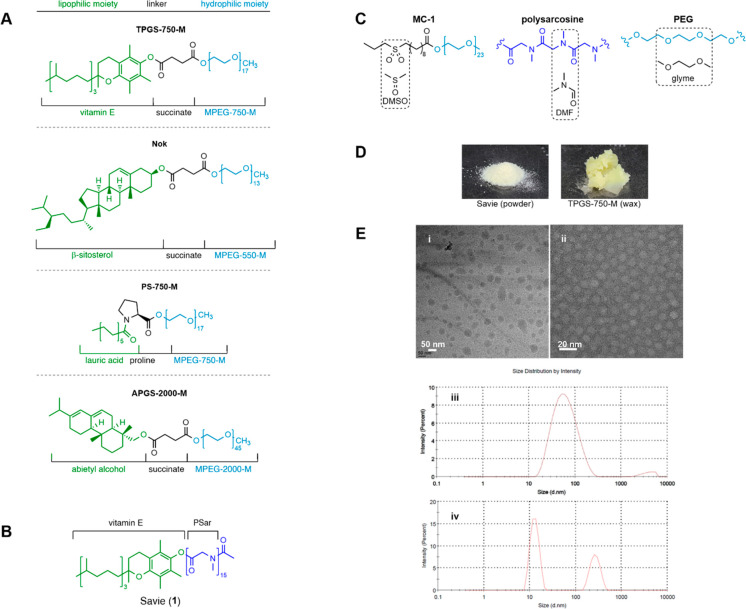
(**A**) Various PEGylated “designer” surfactants; (**B**) structure of Savie (**1**); (**C**) comparisons demonstrating the “solvent-like” nature of various surfactant moieties; (**D**) physical appearance of Savie and TPGS-750-M; (**E**) cryo-TEM images of (i) TPGS-750-M and (ii) Savie; DLS spectra of (iii) TPGS-750-M and (iv) Savie.

A number of “designer” surfactants (so named as they are specifically engineered to maximize effectiveness for use in organic synthesis in water) have been developed over the years (Figure 1A), including the vitamin E-based TPGS-750-M^9^ and β-sitosterol-derived Nok^10^ described by our group, the fatty acid and proline-based PS-750-M from Handa and co-workers,^11^ and the rosin-based APGS-2000-M from the Huang group.^12^ Notably, all of these examples, as well as several other designer surfactants,^13,14^ incorporate simple and/or bioderived lipophilic moieties in keeping with the environmental aims that spurred their development. Unfortunately, in all of the designer surfactants described thus far, polyethylene glycol (PEG) functions as the hydrophilic moiety, potentially inhibiting the biodegradability of the derived surfactant. As a polyether, PEG is known to form hydroperoxides^15^ on prolonged exposure to air, an undesirable trait when performing oxidant-sensitive reactions within a micellar medium. Furthermore, and most notably, non-readily biodegradable PEGylated surfactants may complicate downstream wastewater treatment since the aqueous waste must often be processed to remove the surfactant prior to disposal.^16^ Therefore, a biodegradable, non-peroxide-forming “drop in” replacement for PEG, and therefore, TPGS-750-M in particular, was sought.

### Polysarcosine vs PEG

The use of polypeptides as PEG alternatives has been previously investigated for bioconjugation applications, including PASylation technology (polypeptides containing only proline, alanine, and serine) developed by Schlapschy and co-workers.^17^ These peptides minimize internal hydrogen bonding and, as a result, adopt random coil conformations in water (similar to PEG), thereby avoiding deleterious secondary structures that would limit their solubility. The technologies that enable bioconjugation with PAS peptides (namely, encoding the PAS sequence directly into the gene for the peptide that is being conjugated), however, is not transferable to surfactant synthesis; instead, traditional (i.e., environmentally egregious) methods of peptide synthesis are necessary. The polymerization of amino acid monomers in the form of *N*-carboxyanhydrides (NCAs) to afford polydisperse peptide homopolymers is an attractive alternative that avoids the use of traditional coupling reagents and generates minimal waste (one equivalent of CO_2_ per equivalent of monomer).^18^ Unfortunately, the use of NCAs derived from any of the proteinogenic amino acids would lead to homopolymers that form undesirable secondary structures due to inter- and intramolecular hydrogen bonding.^19,20^ Use of an *N*-methylated amino acid, on the other hand, is known to afford the corresponding *N*-functionalized polypeptide (i.e., polypeptoid), which obviates this potential issue and allows for adoption of a PEG-like random coil conformation in water. The simplest *N*-methylated amino acid, *N*-methylglycine (i.e., sarcosine), is a biogenic, but nonproteinogenic amino acid used in the body for the biosynthesis of compounds such as creatine and is biodegraded to glycine by sarcosine dehydrogenase.^21^ Sarcosine itself has found widespread use in the synthesis of biodegradable surfactants such as sodium lauroyl sarcosinate.^22^ Polysarcosine (PSar) has also been employed as a biodegradable PEG replacement^23^ in a number of bioconjugation^24^ and materials applications,^25^ as well as in the design of a micelle-forming amphiphile by Barz and co-workers.^26^ Unlike PEG, it is nonimmunogenic, does not form hydroperoxides on exposure to air, and is fully biocompatible, all while retaining very similar characteristics to PEG (e.g., adoption of a random coil conformation in water). As such, it was selected as the polypeptoid of choice for the development of a novel, non-PEGylated designer surfactant.

### Savie; A New, Biodegradable Alternative to PEGylated Surfactants

In this work we describe, to the best of our knowledge, the first designer surfactant using a polypeptoid as the hydrophilic moiety. We chose vitamin E (α-tocopherol) as the lipophilic moiety, as it is environmentally and toxicologically benign and biodegradable and because of its successful implementation in the surfactant TPGS-750-M, which has achieved the most extensive adoption in industry among the designer surfactants.^27^ The derived tocopheryl polysarcosinate surfactant is named Savie (**1**; Figure 1B; **Sa**rcosine + **vi**tamin **E**). Herein, Savie is utilized as a “drop in” replacement for TPGS-750-M in several reaction types, focusing especially on those most prevalent in medicinal chemistry,^28^ while demonstrating late-stage functionalization and amenability to a number of catalyst types and reaction conditions. Comparisons were drawn to TPGS-750-M in order to highlight the effect of the hydrophilic moiety, as both Savie and TPGS-750-M utilize vitamin E as their lipophilic interiors. TPGS-750-M was also chosen as a point of comparison to demonstrate the benefits of Savie over the most widely used designer surfactant currently employed in industrial applications.^29^

In facilitating organic reactions in water, it was found that Savie often performed better, sometimes significantly so, in terms of yields compared to TPGS-750-M. It has been demonstrated (*vide infra*) to be amenable to a number of technologies unique to micellar media, including the “nano-to-nano” effect in applications using heterogeneous catalysts,^30^ and multistep, one-pot chemoenzymatic catalysis. In a similar vein to the previously described “DMSO-like” lipophilic moiety of the surfactant MC-1,^14^ the “DMF-like” nature of the polyamide PSar moiety endows Savie with an improved emulsifying capacity compared to “glyme-like” PEGylated TPGS-750-M (Figure 1C). This particular property associated with Savie allows for greater homogeneity of micellar reaction mixtures *without the need for organic cosolvents*, all while achieving typically higher yields.

## Results and Discussion

### Synthesis of Savie

**Scheme 1:**
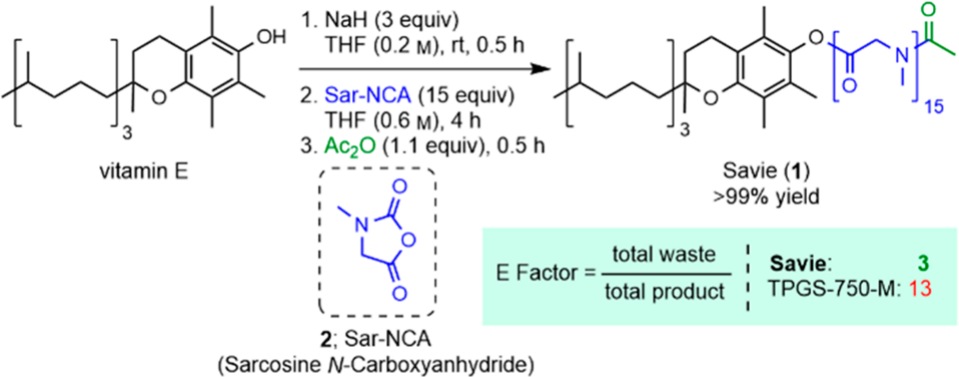
Synthesis of Savie (**1**)

Savie (**1**) was prepared in a simple three-step, one-pot process (Scheme 1). First, vitamin E was deprotonated using NaH in anhydrous THF. The resulting tocopheryl phenoxide was then used as the initiator in the polymerization of sarcosine *N*-carboxyanhydride (**2**; Sar-NCA), which was added slowly to the initiator as a solution in THF in order to ensure a controlled polymerization. Once all of the monomer was consumed, the resulting terminal secondary amine was capped using acetic anhydride to afford Savie. Workup involved filtration to remove precipitated sodium acetate and unreacted NaH (alternatively, NaH can be quenched by adding AcOH) followed by removal and recovery of the THF *in vacuo* to afford Savie as a finely divided white powder (*vide infra*).

Because the only byproducts of the synthesis are H_2_ and CO_2_, which bubble out of solution, and sodium acetate, which is removed by filtration, the recovered THF can be directly reused, indefinitely, to prepare subsequent batches of surfactant, leading to a process that generates very little waste.^31^ A comparison with the literature procedure for the synthesis of TPGS-750-M^9^ (albeit over 10 years old) reveals a more than 4-fold decrease in environmental impact for the synthesis of Savie as measured by Sheldon’s E factor (Scheme 1).^32^ Notably, the decreased E factor does not reflect the elimination of *hazardous* waste generated when comparing Savie to TPGS-750-M (e.g., the DCM and DMAP used previously to synthesize TPGS-750-M are both absent from the synthesis of Savie). Furthermore, the use of the lipophilic moiety, vitamin E, as the initiator in the polymerization obviates the need for a linker, a desirable trait from the viewpoint of downstream waste management.^33^

Because all of the Sar-NCA monomer is consumed during the reaction (i.e., the polymerization is allowed to go to completion), the average chain length of the PSar section of Savie is controlled by the molar ratio of monomer to initiator. As such, a library of α-tocopheryl polysarcosinate surfactants with varying chain lengths was synthesized and used in several micelle-enabled reactions. The results were then compared to those obtained in both pure water (to ensure a micellar catalytic effect was involved) and several other surfactants (TPGS-750-M, Coolade,^13^ Brij 30; see SI, Section 3). These studies indicated that a PSar length of 15 monomer units, on average, is optimal, and it is this 15mer that was named Savie.

Savie will soon be available from Sigma-Aldrich under catalog number #926981.

### Properties of Savie and Its Derived Nanomicelles

Savie exists as a finely divided powder that rapidly dissolves in water (on the order of seconds). By contrast, TPGS-750-M is a sticky, waxy material that is comparatively more difficult to handle (Figure 1D) and requires hours of constant stirring for full dissolution in water. Since solutions of Savie can be prepared immediately before use, there is no longer any incentive to prepare an aqueous reaction medium containing the surfactant significantly ahead of time, thereby lowering the barrier for use of micellar catalysis on scale. Furthermore, solid surfactants are shelf-stable indefinitely, as is the case with Savie, whereas aqueous solutions of both Savie and TPGS-750-M have limited shelf lives (typically ca. three months) owing to the presence of a potentially hydrolyzable ester linkage.

While both TPGS-750-M and Savie have hydrophilic moieties each with ca. 15 repeating units derived from their respective monomers, Savie is far more polar owing to the greater hydrophilicity of its polyamide. Moreover, the sarcosine monomer has a greater mass as compared to ethylene oxide, reflected in Savie’s hydrophilic–lipophilic balance (HLB) value of 14.4 (cf. 12.7 for TPGS-750-M).^34^ Since the extent of foaming observed for a given surfactant is determined predominantly, if not solely, by the nature of the *lipophilic* moiety,^35^ no difference was expected, nor observed, in the foaming characteristics of aqueous solutions of either Savie or TPGS-750-M (since both possess the same vitamin E lipophilic moiety).

Savie self-assembles into nanomicelles at a critical micelle concentration (CMC) of 0.015 wt %, compared to a CMC of 0.06 wt % associated with TPGS-750-M. Historically, cryo-TEM imaging and dynamic light scattering (DLS) have been used to assess the size, shape, and aggregation properties of micelles derived from PEGylated “designer” surfactants. TPGS-750-M, for instance, almost exclusively exists as 45–60 nm micellar aggregates consisting of roughly 30–40 ca. 10 nm spherical micelles (Figure 1E, i and iii).^9^ Savie, on the other hand, exists as a mixture of predominantly individual (ca. 13 nm) spherical micelles, as well as larger (ca. 270 nm) aggregates as determined by DLS and cryo-TEM imaging (Figure 1E, ii and iv). This seems to go against the narrative hypothesized for PEGylated surfactants that the formation of micellar aggregates of 45–60 nm is unique, leading to their enabling properties. This divergence from expectation implies that “new rules”^36^ must exist for PSar-containing surfactants vs those containing PEG chains, the properties of which are governed by other factors (e.g., the polarity of the PEG replacement).

A property common among PEGylated compounds is the existence of its cloud point, or the temperature at which the hydrogen-bonding interactions between PEG and water weaken to the point that a micelle-to-vesicle, micelle-to-sheet, or other morphological transitions cause the size of self-assembled particles to increase to the point that a cloudy solution forms.^37^ Aqueous TPGS-750-M is observed to reach a cloud point at ca. 75 °C, and at temperatures around 90–100 °C, the surfactant is observed to drop out of solution entirely (see SI, Figure S8). Aqueous Savie, however, does not reach a cloud point even up to the boiling point of the solution, suggesting that the H-bonding interactions between the polyamide and surrounding water are much stronger than those involving PEG.^38^ This suggests that Savie might perform better than TPGS-750-M in high-temperature applications where stable emulsions are required, a possibility that is currently being investigated in our group using high-temperature plug flow conditions. A related phenomenon is the propensity for PEGylated surfactants to precipitate out of solution as the ionic strength increases, notably with the use of ionic bases such as potassium carbonate and potassium phosphate (bases routinely used in reactions such as Suzuki–Miyaura cross couplings).^39^ This occurs because the salt competes with PEG for interactions with water (i.e., the “salting out” effect), eventually pushing PEG out of solution. This has implications for applications where homogeneity is paramount, such as in microfluidic flow applications. While TPGS-750-M is shown to precipitate from solution at K_3_PO_4_ concentrations as low as 0.25 M, Savie is not observed to precipitate until K_3_PO_4_ concentrations exceed 0.45 M (see SI, Table S4).

The stability of aqueous Savie under high ionic strength conditions, in addition to its high degree of insolubility in most commonly used extraction solvents typically used to recover products from micellar catalytic reaction mixtures (e.g., isopropyl acetate, *i*-PrOAc, and methyl *tert*-butyl ether, MTBE), means that it is not readily extracted from aqueous reaction mixtures. Indeed, it was found that only ca. 3% of the total amount of Savie from a 2 wt % aqueous solution containing 0.75 M K_3_PO_4_ (a typical concentration of base used in many cross-coupling reactions) was extracted with either MTBE or *i*-PrOAc (cf. 66% and 44% for TPGS-750-M in MTBE and *i*-PrOAc, respectively).^40^ This translates into better recycling properties of the aqueous reaction medium and significantly less surfactant contaminating the final product, thereby potentially simplifying workup and downstream processing.

Computational studies indicated that Savie, like TPGS-750-M, is essentially nontoxic, showing no evidence of mutagenic toxicity.^41^

### Suzuki–Miyaura Cross Couplings

**Scheme 2:**
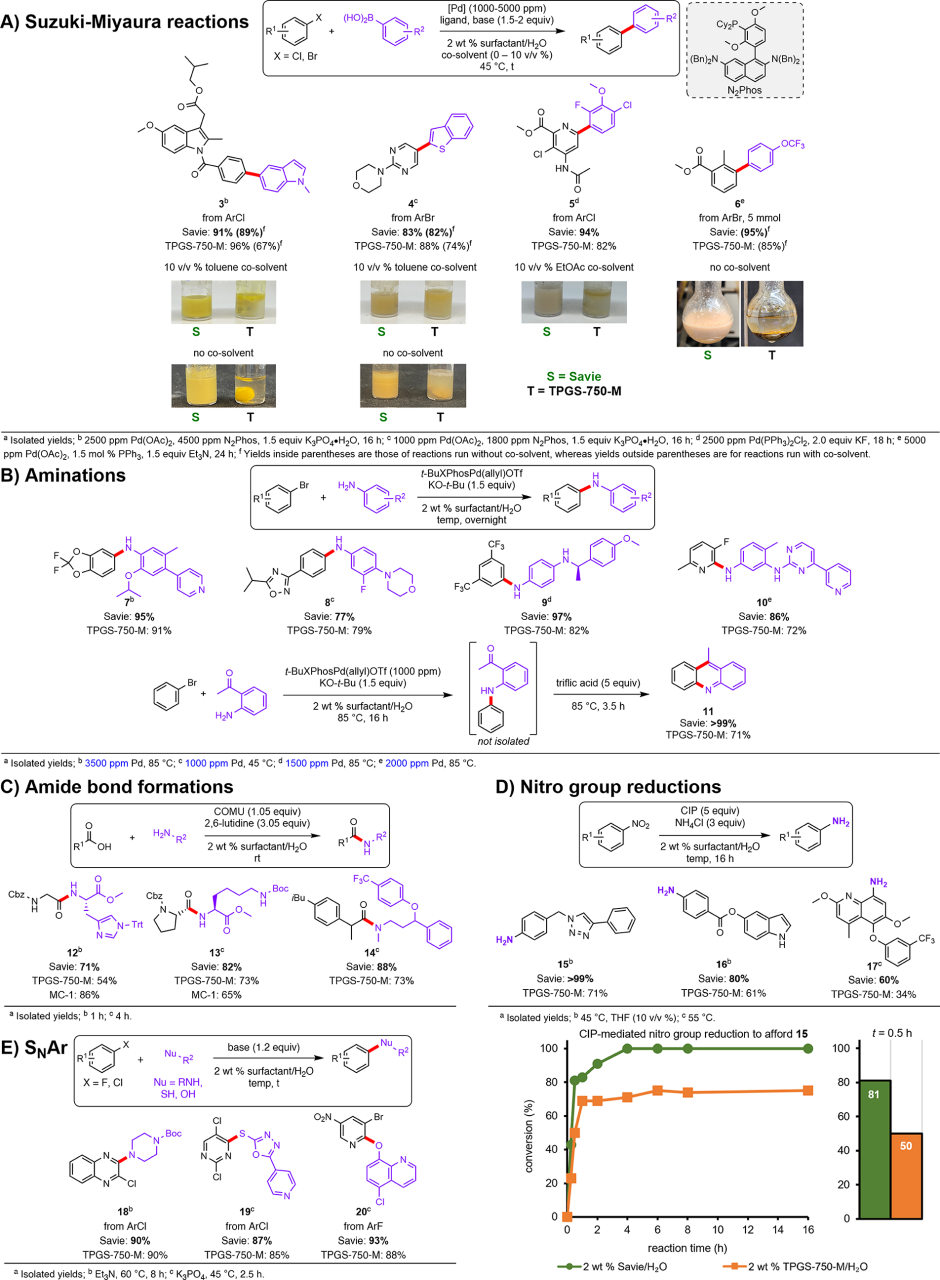
Reactions Performed in Aqueous Surfactant Media: (A) Suzuki–Miyaura Cross Couplings and Their Associated Reaction Mixtures; (B) Aminations, Including Two-Step, One-Pot Synthesis of Acridine **11**; (C) Amide Bond Formations; (D) Nitro Group Reductions and Rate Comparison in 2 wt % Aqueous Solutions of Savie and TPGS-750-M; (E) S_N_Ar Reactions

As stated previously, we chose to perform the most commonly used reactions in medicinal chemistry^28^ to demonstrate Savie’s practical utility and applicability. Suzuki–Miyaura cross couplings (SMCs) are a case in point and have been extensively optimized for use in aqueous micellar media. This has included development of new ligands with each being “matched” to chemistry in water, thereby enabling ppm levels of palladium loading in these important C–C bond-forming processes. One example is N_2_Phos (see Scheme 2A),^42^ a bulky phosphine ligand that promotes formation of a more active 1:1 Pd/ligand complex (as opposed to the less active 1:2 complex seen in previous iterations of similar ligands),^43^ as well as possessing excellent hydrophobicity. This characteristic presumably enhances its binding constant, together with its ligated Pd, increasing its time spent within the micellar lipophilic core and, hence, in close proximity to the reaction partners, thereby enhancing both rate and conversion. This ligand technology was applied to ppm Pd-catalyzed SMCs using industrially relevant compounds, such as indomethacin derivative **3** and the heteroatom-rich biaryl **4** (Scheme 2A). When the typical 10 v/v% of toluene was included as cosolvent, TPGS-750-M and Savie performed identically in terms of reaction efficiency. However, reactions in Savie appeared to be significantly more homogeneous and formed stable emulsions, particularly in the case affording the indomethacin derivative **3**. To further examine the apparently greater emulsifying capacity of Savie, the reactions were repeated *in the absence of cosolvent* (i.e., using water containing only a surfactant). The reaction mixture containing Savie remained nicely emulsified, giving the same yield, within experimental error, as the reactions run in the presence of toluene cosolvent. By contrast, the reactions run in aqueous TPGS-750-M tended to form clumps, thereby occluding starting materials and impeding full conversion, as reflected by the lower yields vs those run in the presence of cosolvent.

The SMC to form an intermediate **5** en route to Corteva’s Arylex was also evaluated. An improved yield was obtained compared to that isolated from the corresponding reaction run in TPGS-750-M. This is likely due to the effect of the more polar PSar residue (compared to PEG). Furthermore, the reaction in TPGS-750-M formed an unstable emulsion (i.e., the product oiled out), whereas the emulsion in Savie was stable. For this specific, extremely insoluble reaction partner combination, a cosolvent was required independent of surfactant, suggesting a limitation in solubilizing properties associated with Savie in such demanding situations. However, it was found that comparatively greener EtOAc could be used in place of toluene as cosolvent to achieve high levels of conversion and, hence, isolated yield (94%).

To demonstrate the potential applicability of this novel surfactant to larger-scale synthesis, a gram-scale reaction producing biaryl **6** en route to the antitumor agent sonidegib was performed. The expected emulsifying properties were observed, along with a high level of conversion and, hence, isolated yield (95%), without added cosolvent. Savie’s greater solubilizing capacity also has significant implications in pilot-scale reactions using aqueous surfactant media, as poor emulsions can lead to conversion issues in multikilogram reactions. For example, process chemists at Novartis will typically employ up to 50 v/v % of organic cosolvent in order to guarantee adequate emulsions using TPGS-750-M;^44^ use of Savie may dramatically reduce this dependence on cosolvent use, thereby avoiding otherwise environmentally egregious organic solvents.

### Aminations

In order to form valuable C–N bonds between amines and aryl/heteroaryl coupling partners, Pd-catalyzed cross-couplings are commonly employed (i.e., Buchwald–Hartwig aminations). Typical contemporary methods use environmentally egregious organic solvents, elevated temperatures, and most notably excessive palladium loadings, oftentimes in the unsustainable and costly 1–10 mol % range, as confirmed in a recent review.^45^ Previously, we reported such Pd-catalyzed aminations in water containing TPGS-750-M using only *ppm quantities of palladium*,^46^ run under mild conditions using the Colacot catalyst (*t*-BuXPhosPd(allyl)OTf).^47^ This same technology has now been examined using a series of complex partners in aqueous Savie in the total absence of organic cosolvent (Scheme 2B). While for products **7** and **8**, both surfactants performed comparably in terms of yields, diarylamine product **9** and the nitrogen-rich polycyclic product **10** were obtained in higher yields using aqueous mixtures containing Savie. It is important to highlight that each of these couplings could be run using between 1000 and 3500 ppm of a Pd catalyst (i.e., 0.10–0.35 mol %) in water and that no other existing *general* procedures are in any way competitive with this technology, even in organic solvents.

One application of this coupling advance focuses on forming acridine **11** via a two-step, one-pot ppm Pd-catalyzed amination followed by an acid-catalyzed cyclization. This sequence is aided by use of a lipophilic (sulfonic) acid, e.g., triflic acid, which presumably accesses the lipophilic micellar inner core. Product **11** was isolated in quantitative yield enabled by aqueous Savie, while under otherwise identical conditions, only 71% yield was isolated using an aqueous TPGS-750-M solution. Although the amination went to full conversion (by TLC) in both surfactants using only 0.1 mol % of the Pd catalyst, it was the cyclization step that proceeded best in Savie.

### Amide Bond Formation

This type of reaction represents, by far, the most widely utilized transformation in the pharmaceutical industry.^28^ Commonly used reagents and methods for these syntheses can produce large volumes of hazardous waste, with associated PMIs in excess of 45.^48^ Our previously developed methodology utilizes the relatively benign coupling reagent COMU (compared to HATU, DCC, DIC, etc.), together with the mild base 2,6-lutidine to form amide bonds in water.^14^ Products **12** and **13** were formed to demonstrate the applicability of using an aqueous medium containing Savie to enable peptide synthesis (Scheme 2C), the outcomes from which were compared directly with both TPGS-750-M and the sulfone-containing surfactant MC-1 (Figure 1A), which was specifically engineered to enable facile peptide bond formation in water. The reaction partners chosen reflected the difficulty associated with their emulsification in aqueous TPGS-750-M. In both cases, Savie’s “DMF-like” polyamide led to more effective emulsions compared to TPGS-750-M, leading to better isolated yields. With amide **12**, however, MC-1 significantly outperformed both TPGS-750-M and Savie, with Savie still leading to a much higher yield than TPGS-750-M. In comparison, MC-1 fared worse than both surfactants en route to peptide **13**, with use of Savie improving the isolated yield by nearly 10% over TPGS-750-M.

A comparison between TPGS-750-M and Savie was also made in the amide bond-forming reaction to afford the relatively lipophilic product **14** derived from starting materials racemic fluoxetine HCl and ibuprofen. Once again, Savie led to a greater extent of conversion and, hence, a higher isolated yield. Given that the lipophilic moiety common to both TPGS-750-M and Savie is identical, the difference between the two surfactants’ performance in these amide bond-forming reactions must be attributable to the difference in their hydrophilic residues. In addition to enabling better emulsions, it is plausible that Savie primarily solubilizes the highly polar starting materials and reaction intermediates within the PSar region of its micelles.

### Nitro Group Reductions

These are commonly utilized transformations for the synthesis of anilines and are applied widely across the chemical enterprise. A method of particular interest is the use of carbonyl iron powder (CIP),^49^ which is a type of pure iron in the form of a finely divided powder that avoids the potential issues of low reactive surface area (and associated slow reaction rates) and abrasiveness of traditional iron filings, as well as the pyrophoric nature of nanoiron, all while being remarkably inexpensive. CIP is highly reactive toward, and selective for, the reduction of nitroarenes to anilines in micellar media. One common challenge encountered in this chemistry is the highly crystalline, and thus difficult to emulsify, nature of nitroarenes. To soften the crystal lattice, pretreatment with water-miscible THF is often employed. Indeed, this THF pretreatment was still required for reactions in Savie for particularly insoluble substrates, e.g., leading to products **15** and **16** (Scheme 2D). Nonetheless, reactions in aqueous TPGS-750-M still significantly lagged behind those run in Savie, suggesting that the polyamide was better at breaking up the crystal lattice of the nitroarene and exposing more of the substrate to reactive CIP. It is also plausible that the more polar Savie is better able to clean the surface of the iron, thereby increasing the reactive surface area. The yield in the nitro reduction to afford aniline **17**, a precursor to the antimalarial drug tafenoquine,^50^ nearly doubled using a micellar medium composed of Savie instead of TPGS-750-M.

To better elucidate reaction kinetics, a rate study was conducted by comparing the conversion in a nitro group reduction to aniline **15** at various time points in aqueous solutions of Savie vs in TPGS-750-M. These data reveal a marked increase in rate for this reaction performed in Savie, reaching 81% conversion in only 30 min, compared to only 50% conversion in TPGS-750-M. After 4 h, the reaction in Savie was complete, whereas in TPGS-750-M the same reaction plateaued at 75% conversion after 6 h.

### S_N_Ar Reactions

S_N_Ar reactions have been thoroughly explored in micellar media,^51^ where these conditions have been found to effectively replace the common use of dipolar, aprotic solvents for such purposes. For this study, substrates were chosen for purposes of demonstrating the array of nucleophiles that can be utilized, including an amine leading to product **18**, a thiol affording product **19**, and a phenol giving rise to product **20** (Scheme 2E). In all cases, reactions in Savie showed comparable results to those using TPGS-750-M.

### Various Transition Metal-Catalyzed Reactions

**Scheme 3:**
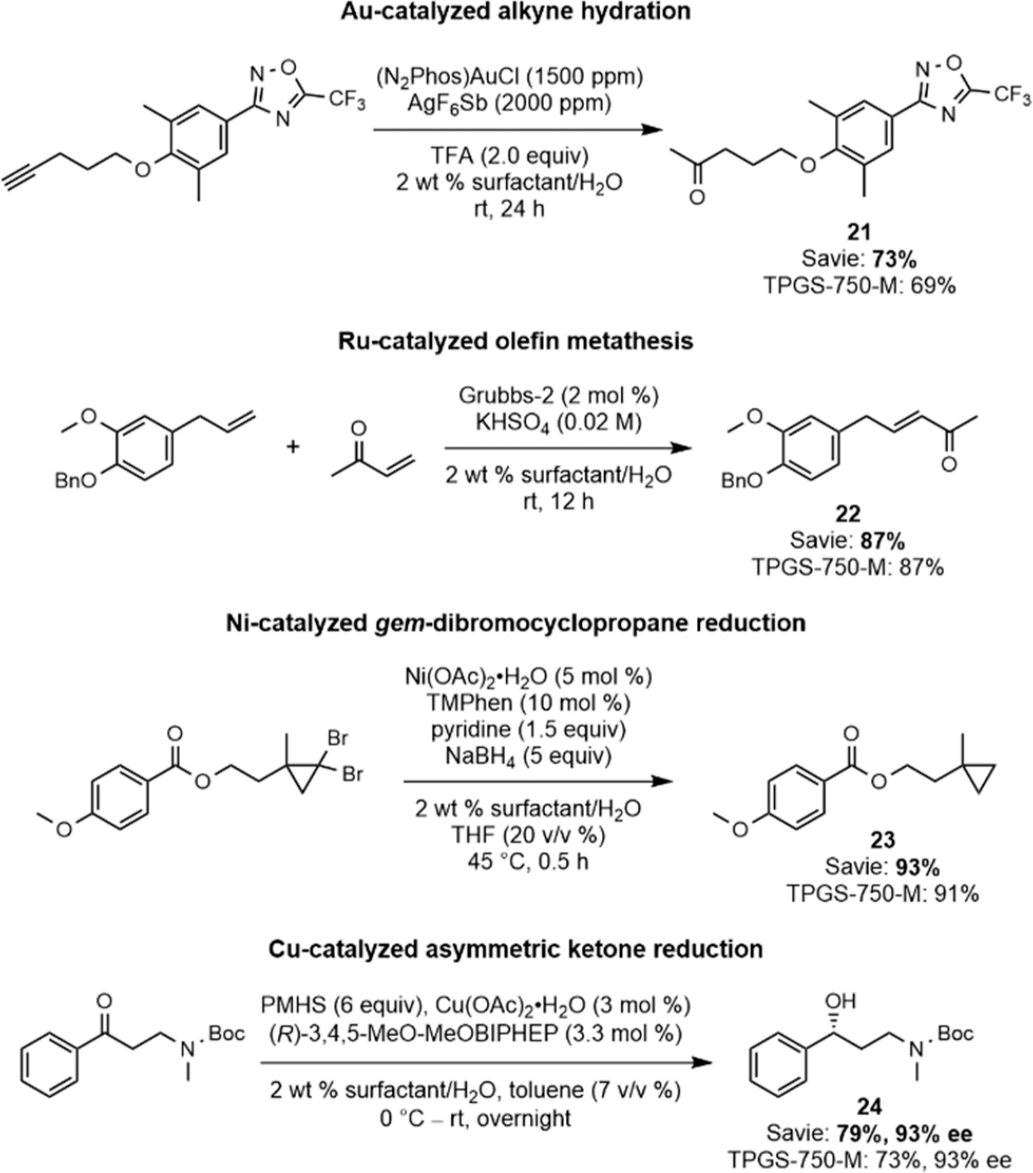
Various Transition Metal-Catalyzed Reactions Performed in Aqueous Surfactant Media, Demonstrating the Compatibility of Savie with Various Catalyst Types

To demonstrate that the polyamide (i.e., PSar) of Savie does not interfere with various transition metal catalysts, reactions (shown in Scheme 3) were performed using catalysts containing gold (e.g., Au-catalyzed alkyne hydration of a precursor to the antiviral drug pleconaril to afford ketone **21**),^52^ ruthenium (e.g., Ru-catalyzed olefin metathesis to afford enone **22**),^53^ nickel (e.g., Ni-catalyzed reduction of a *gem*-dibromocyclopropane to afford ester **23**),^54^ and copper (e.g., asymmetric CuH-catalyzed reduction of a ketone to afford alcohol **24**, an intermediate toward fluoxetine).^55^ In all cases, Savie performed as well as TPGS-750-M. In the case of fluoxetine precursor **24** synthesized via asymmetric Cu-catalyzed reduction of the ketone, the enantioselectivity was identical between the two surfactants.

### Heterogeneous Catalysis and the “Nano-to-Nano” Effect

**Scheme 4:**
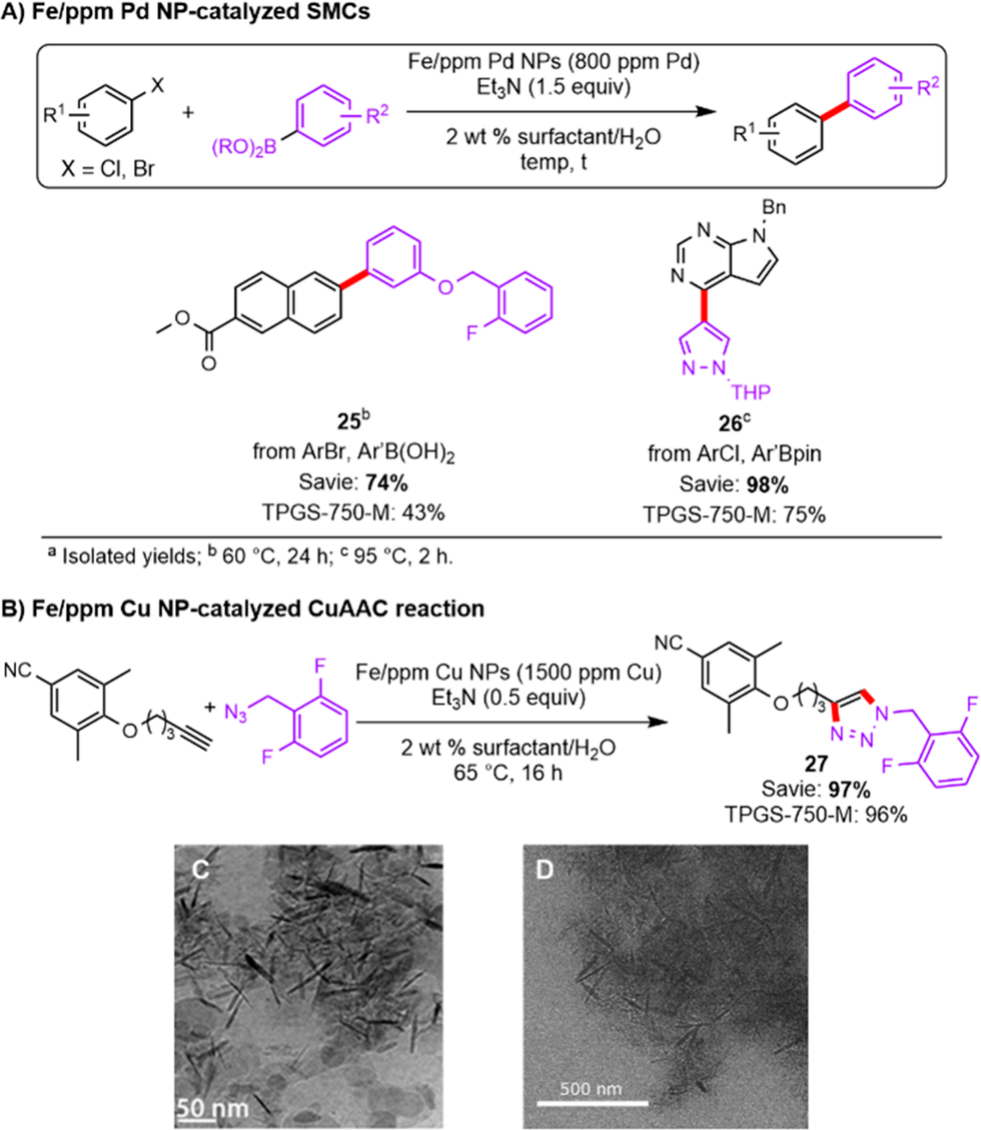
Fe/ppm Metal Nanoparticle (NP) Catalyzed Reactions in Aqueous Surfactant Media: (A) Suzuki–Miyaura Cross Couplings (SMCs); (B) Cu-Catalyzed Azide Alkyne Cycloaddition (CuAAC); Visual Representation of the “Nano-to-Nano” Effect, i.e., Nanomicelles Associated with Fe/ppm Pd NPs Illustrated by (C) Cryo-TEM Image of TPGS-750-M Micellar Aggregates (2 wt %) and NPs and (D) TEM Image of Savie Micelles (2 wt %) and NPs

Work by Hou and co-workers demonstrated that the polyether backbone of PEG forms favorable interactions between the ethereal oxygen atoms and Pd nanoparticles (NPs), thereby stabilizing them and preventing Ostwald ripening.^56^ This formed the basis of research conducted in our group which demonstrated that specially formulated, rod-shaped Fe/ppm Pd NPs were stabilized by the PEG moiety of TPGS-750-M in aqueous solution.^57^ This stabilization is seen in the agglomeration of nanomicelles around the metal nanorods of catalyst (see cryo-TEM image in Scheme 4C), and this phenomenon has been referred to as the “nano-to-nano” effect.^30,58^ Importantly, the close proximity of substrate-laden nanomicelles to the catalytic surface of the nanorods enabled not only very low loadings of catalyst (typically lower than 1000 ppm Pd) to achieve excellent levels of conversion, but also for these heterogeneous reactions to occur under atypically mild conditions. In addition to NPs containing Pd, this technology has been applied to NPs containing other metals including Cu^59^ and Ni.^60^

In a similar vein, it has been previously reported that the amide group of DMF is capable of associating with and stabilizing various metal nanoparticles,^61^ including those composed of Pd^62^ and Cu.^63^ By way of extrapolation, it was expected that the DMF-like PSar moiety of Savie would serve in a similar capacity, leading to an amide-promoted nano-to-nano effect. Indeed, TEM imaging confirmed that Savie-derived nanomicelles are found associated with Fe/ppm Pd NPs (Scheme 4D). To confirm that this amide-promoted nano-to-nano effect still gives rise to ppm level catalysis, two Pd-catalyzed Suzuki–Miyaura cross-coupling reactions (SMC; Scheme 4A) and a copper-catalyzed azide–alkyne cycloaddition reaction (CuAAC; Scheme 4B) were performed in 2 wt % aqueous solutions of both Savie and TPGS-750-M using the corresponding Fe/ppm metal NPs. In the case of the SMC reactions to afford biaryl products **25** and **26** (a precursor to several JAK inhibitors, e.g., ruxolitinib and baricitib), and using only 800 ppm Pd (0.08 mol % catalyst), Savie significantly outperformed TPGS-750-M likely owing to its greater emulsifying capacity in the absence of organic cosolvent. The CuAAC reaction relying on only 1500 ppm of Cu led to nearly quantitative conversion to **27** in both surfactants. These results satisfactorily confirmed the existence of an amide-induced nano-to-nano effect in Savie, enabling efficient ppm metal catalysis.

### Biocatalytic Transformations

**Figure 2:**
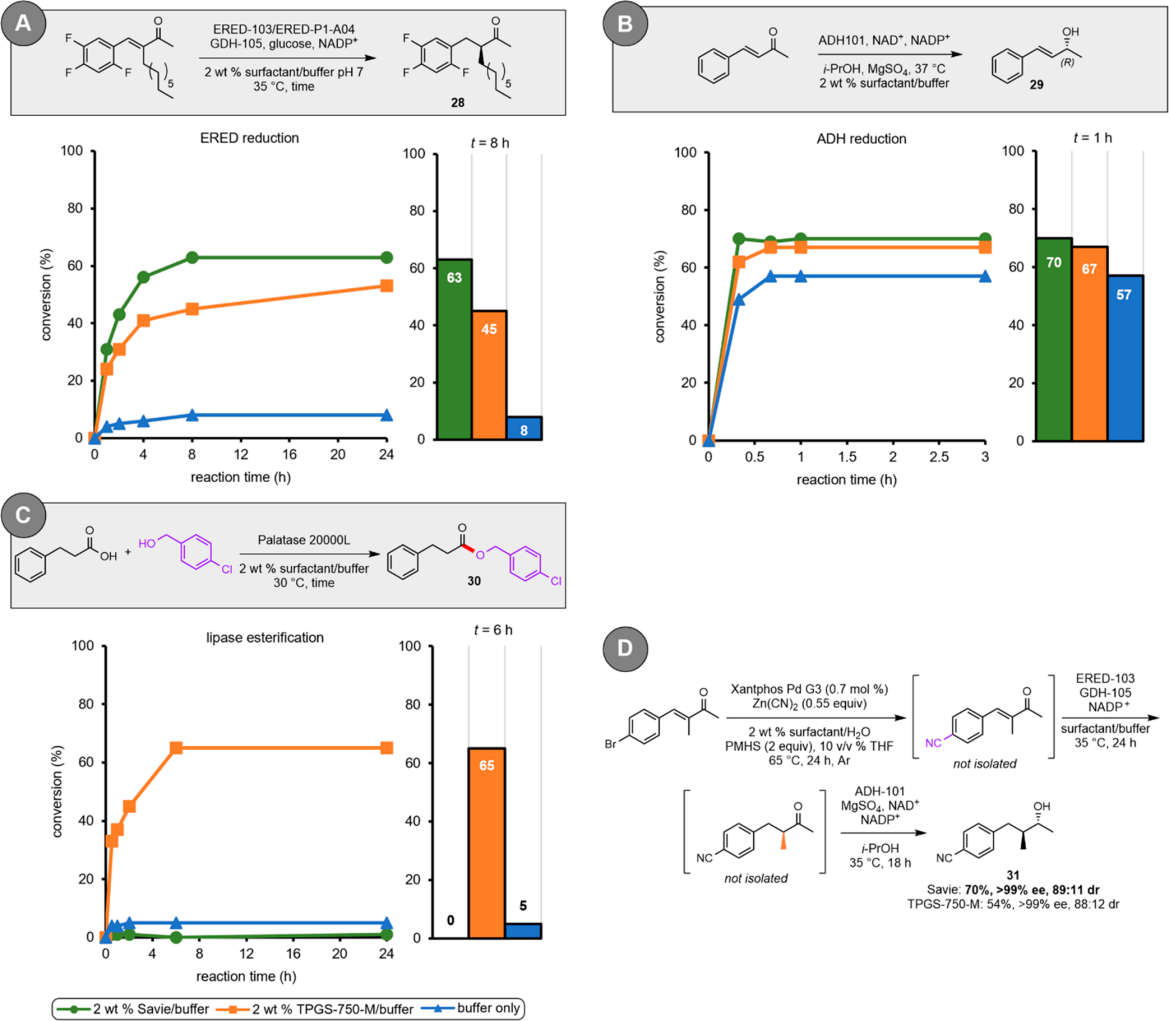
Comparison of various media in the (A) ERED-catalyzed reduction of an α,β-unsaturated ketone; (B) ADH-catalyzed ketone reduction; (C) lipase-catalyzed esterification; and (D) three-step, one-pot chemoenzymatic sequence: Pd-catalyzed cyanation/ERED reduction/ADH reduction in aqueous surfactant media.

Synthetic reactions involving biocatalysis have emerged as a powerful tool for bond connections and selective functional group transformations that can be done very efficiently in an aqueous medium.^64^ Inclusion of a surfactant in the water has been shown, on occasion, to dramatically increase conversions in several biocatalyzed reactions owing to the nanomicelles’ ability to house products, thereby preventing their eventual saturation of the enzyme’s active site (i.e., the “reservoir effect”).^58,65^ The ability of Savie to facilitate biocatalyzed reactions and mitigate product inhibition was assessed in a series of reactions involving ERED-103 (an ene-reductase that asymmetrically reduces the olefinic portions of α,β-unsaturated ketones; Figure 2A),^66^ ADH101 (a keto-reductase that asymmetrically reduces ketones to alcohols; Figure 2B),^65^ and palatase 20000L (a lipase that forms ester bonds; Figure 2C).^67^ In the case of ERED-103, Savie led to an increase in conversion vs that observed in both TPGS-750-M and pure aqueous buffer, although both surfactants showed a pronounced reservoir effect. In the case of ADH101, both surfactants showed a similar improvement over buffer. In the case of palatase 20000L, however, TPGS-750-M performed significantly better than did the reaction in pure buffer, while Savie appeared to completely inhibit this enzymatic reaction. Circular dichroism (CD) spectra taken of each enzyme in the presence of pure buffer, as well as solutions of TPGS-750-M and Savie in buffer, showed no evidence of denaturation (see SI, Section 6). The absence of denaturation suggests that Savie interferes with palatase 20000L’s ability to process substrates without affecting the secondary structure of the enzyme. While further investigation is needed, we posit that the relatively polar (compared to PEG) PSar polyamide may prevent the helical lid of palatase 20000L from opening, an important step in lipase-catalyzed reactions which benefit from a more *hydrophobic* environment.^68^

### Three-Step, One-Pot Chemoenzymatic Sequence

As the toolbox of established reactions that can now be run in the same aqueous reaction medium continues to expand, so do opportunities to telescope reactions that can now be run in a single pot without reliance on wasteful intermediate workup and purification steps. Of particular note in this regard is the inclusion of biocatalytic processes that, likewise, take place in water. Thus, performing chemo- and biocatalyzed reactions in a variety of one-pot sequences in the same aqueous medium represents an exciting area of synthesis oftentimes referred to as chemoenzymatic catalysis.^69^ To demonstrate this capability, a tandem sequence was performed involving an initial Pd-catalyzed cyanation, followed by asymmetric reduction of the olefin by an ene-reductase (ERED-103), and final asymmetric ketone reduction by a keto-reductase (ADH101; Figure 2D). The reaction in Savie achieved a 70% overall isolated yield of **31**, whereas the sequence in TPGS-750-M led to a 54% overall isolated yield. In both cases, **31** was isolated in >99% ee, while the dr was essentially unchanged for both. The improved yield in Savie is likely attributable to a greater extent of conversion using this medium during the ERED-catalyzed reaction.

### Purification-Free Amide Bond Formation

**Scheme 5:**
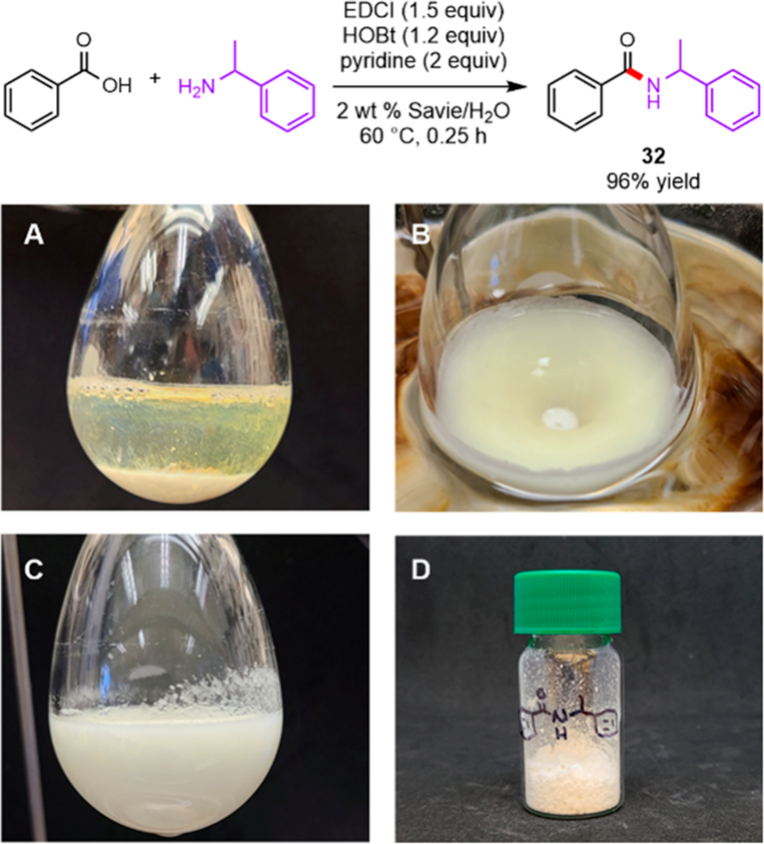
Gram-Scale Amide Bond Formation in 2 wt % Savie/H_2_O with Accompanying Images of the Reaction Mixture (A) before Stirring and (B) during and (C) after Reaction; (D) Product **32** Isolated via Filtration

In order to exemplify the facile processability of reactions performed in a medium containing aqueous Savie, a gram-scale amide bond formation was performed, adapted from a similar protocol reported by the Handa group (Scheme 5).^70^ This methodology required only filtration and washing with water to obtain pure product without the need for recrystallization or column chromatography, leading to **32** in 96% yield.

### Recycling of the Aqueous Medium for Use in a Ni-Catalyzed Migita C–S Bond Formation

**Figure 3:**
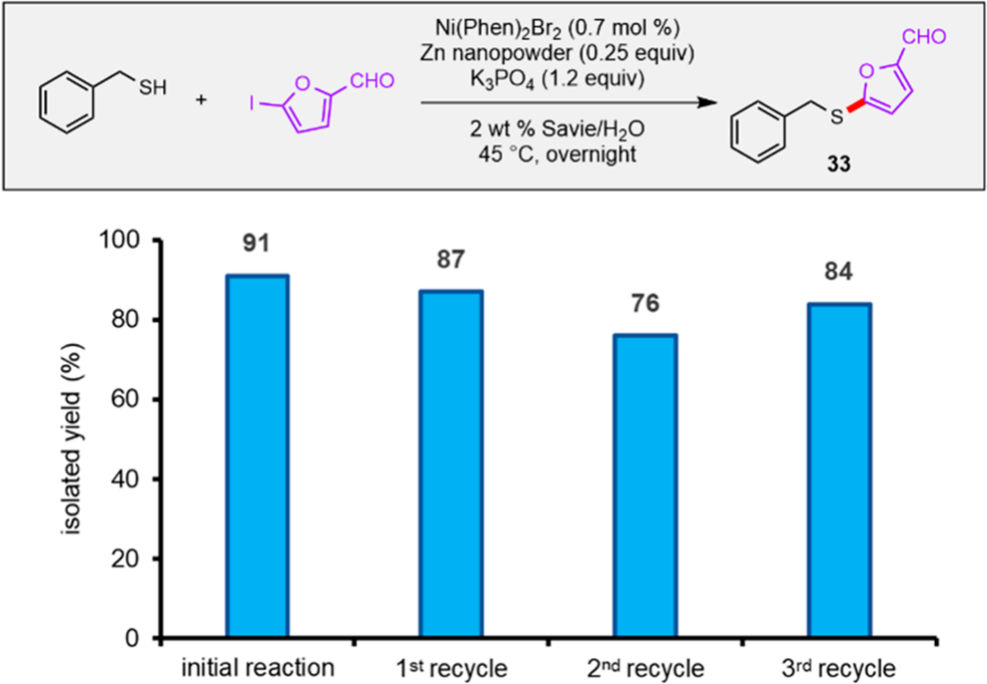
Recycling study of the Ni-catalyzed Migita-like reaction to afford **33**. Recycling involved reuse of the aqueous medium only (i.e., fresh catalyst and reactants were added for each recycle).

To enhance the greenness of chemistry in water, recycling of an aqueous micellar medium is an important consideration that can dramatically reduce the overall environmental impact relative to use of organic solvents. To demonstrate the facility with which aqueous solutions of Savie can be recycled, a Ni-catalyzed Migita-like C–S bond formation^71^ leading to **33** was performed in Savie-derived nanomicelles, after which the aqueous medium was reused three times (i.e., a total of four reactions were performed in the same medium; Figure 3). Little change in reaction outcomes was observed in successive recycles, although the study had to be halted after the third usage due to the buildup of salts in the aqueous medium, inhibiting stirring of the resultant slurry.

### Drawbacks and Limitations of Savie vs TPGS-750-M

Perhaps most notably, polysarcosine is currently more expensive than commercially available MPEGs, although it is also worth noting that this should add relatively little cost to a process even at scale, as only 2 wt % surfactant is used in a typical micellar medium. Moreover, any additional cost might well be made up by improved yields, elimination of cosolvent usage, and simplified downstream wastewater treatment. It has also been observed that certain silanes (e.g., triethoxysilane) induce gelation of the 2 wt % Savie/H_2_O medium and thus impede mixing and mass transfer. This is not the case for polysiloxanes (e.g., PMHS), as exemplified by the syntheses of substrates **24** and **31** (*vide supra*). Finally, Savie’s greater emulsifying capacity compared to PEGylated surfactants can also affect the solvents used to extract products on workup, leading to potential complications on separation (e.g., a need for centrifugation). In general, this is avoided by simply employing gentle mixing during extraction. However, in cases where it is not avoidable, it can typically be addressed by adding NaCl to the mixture and/or gentle heating to separate the two phases.

## Summary

This report discloses a newly designed, “drop in” replacement surfactant, Savie, for what has been the workhorse amphiphile, TPGS-750-M, that has enabled chemistry in water for well over a decade. The preparation of Savie relies on readily available starting materials, utilizing an efficient one-pot process. Unlike all other known surfactants that participate in micellar catalysis, Savie offers the synthetic community a multitude of especially timely and environmentally responsible features. These key attributes are likely to play increasingly important roles in looking toward a future of organic synthesis that relies on nature’s chosen reaction medium, water. These include the following:

- replacement of the typical hydrophilic PEG portion with a polypeptoid derived from *N*-methylglycine, thus arriving at a fully biodegradable amphiphile that minimizes downstream wastewater processing
- avoidance of organic cosolvents that are commonly used to ensure that well-behaved emulsions are present throughout reactions, especially when run at scale
- facile dissolution in water given the powdery consistency of Savie, thus eliminating an investment of time normally associated with the preparation of fresh solutions of waxy TPGS-750-M
- enhanced reaction rates and levels of conversion for a multitude of reaction types, thereby leading to higher isolated yields
- better enabling properties for aqueous micellar catalysis applied to multistep chemoenzymatic catalysis, leading to “clean chemistry in dirty water”,^72^ along with concomitant savings due to time, pot, and even metal economies

## Supporting Information Available

Experimental procedures, optimization details, and analytical data of isolated materials (NMR, HPLC, HRMS, MALDI TOF, FTIR, melting points) (PDF)
Annotated time-lapse video of Savie polymerization (MP4)
