# Supplementary material for: Introducing Savie: A Biodegradable Surfactant Enabling Chemo- and Biocatalysis and Related Reactions in Recyclable Water
Source: J Am Chem Soc. 2023 Feb 8;145(7):4266–78. doi: 10.1021/jacs.2c13444 (PMC9951251; doi:10.1021/jacs.2c13444)
Supplement: Supplementary file 1 — ja2c13444_si_001.pdf [file ja2c13444_si_001.pdf]

Introducing Savie: A Biodegradable Surfactant Enabling Chemo- and Biocatalysis and Related Reactions in Recyclable Water

Joseph R. A. Kincaid,[1] Madison J. Wong,[1] Nnamdi Akporji,[1] Fabrice Gallou,[2] David M. Fialho,[1] and Bruce H. Lipshutz*[1]

[1]Department of Chemistry and Biochemistry, University of California, Santa Barbara, CA 93106 USA
[2]Novartis Pharma, Basel, Switzerland

Phone : 805-893-2521
Fax : 805-893-8265
Email: lipshutz@chem.ucsb.edu
Website: https://lipshutz.chem.ucsb.edu/

Supporting Information

Table of Contents

1. General Information S2
2. Synthesis and Characterization of Savie S4
3. Polysarcosine (PSar) Polymer Length Optimization S14
4. Dynamic Light Scattering (DLS) Spectra of Savie with Varying PSar Lengths S18
5. Temperature and Salt Stability Comparison Between Aqueous Savie and TPGS-750-M S18
6. Enzyme Denaturation Experiments Using Circular Dichroism (CD) S19
7. Procedures for Reactions Performed in Aqueous Savie vs. TPGS-750-M S24
7.1 Ppm Pd-Catalyzed Suzuki-Miyaura Coupling (SMC) Reactions S24
7.2 Ppm Pd-Catalyzed Amination Reactions S27
7.3 Amide Bond Formation Reactions S30
7.4 CIP-Mediated Nitro Group Reductions S30
7.5 S_NAr Reactions S32
7.6 Ppm Au-Catalyzed Alkyne Hydration S33
7.7 Ru-Catalyzed Olefin Metathesis S34
7.8 Ni-Catalyzed *Gem*-Dibromocyclopropane Reduction S35
7.9 CuH-Catalyzed Asymmetric Ketone Reduction S37
7.10 Fe/ppm Pd Nanoparticle-Catalyzed Suzuki-Miyaura Couplings S38
7.11 Fe/ppm Cu Nanoparticle-Catalyzed CuAAC Reaction S40
7.12 Biocatalytic Reactions S41
7.13 1-Pot Pd-Catalyzed Cyanation, then ERED Reduction, then ADH Reduction S43

7.14 Gram-Scale Amide Bond Formation S45
7.15 Ni-Catalyzed Migita-Like C-S Bond Formation and Recycling S45
8. References S46
9. Experimental Data S49
10. ^{1}H, ^{13}C, and ^{19}F NMR Spectra of Synthesized Products S72

1. General Information

Safety Statement:

No unexpected or unusually high safety hazards were encountered. However, areas where extra safety precautions were taken have been explicitly noted.

Reagents and Biocatalysts:

All commercially available reagents were used without further purification with the exception of *N*-Boc-Sar (Sar = sarcosine) which was purified by hot filtration with *i*PrOAc followed by recrystallization from hot *i*PrOAc. Enzyme ERED-103 is commercially available from the Codex® Ene Reductase Screening kit from Codexis. Enzyme ADH101 is commercially available from the enzyme kit EZK-001 from Johnson Matthey. Enzyme Palatase20000L was purchased from Strem Chemicals Inc. (cat. no. 06-3118). Reagents were purchased from Sigma-Aldrich, Combi-Blocks, Alfa Aesar, or Acros Organics.

Surfactant Solution Preparation:

A 2 wt % TPGS-750-M/H_2O solution was prepared by dissolving TPGS-750-M in degassed HPLC grade water; likewise, 2 wt % aqueous solutions of Savie, Coolade, and Brij 30 were prepared in the same manner. TPGS-750-M was made as described previously[1] and is also commercially available from SigmaAldrich (catalog #733857 (solution) or #763896 (wax)). Savie was prepared as described below, and will soon be available from SigmaAldrich (catalog #926981) and in larger quantities from PHT International. Coolade was prepared according to a literature procedure.[2] Brij 30 was purchased from Acros Organics. HPLC-grade water was obtained from Fischer Scientific and was purged with argon before use.

Chromatography:

Silica gel TLC plates (UV 254 indicator, thickness 200 mm standard grade, glass backed and 230-400 mesh from Merck) were used. The developed TLC plate was analyzed by a UV lamp (254 nm). The plates were further analyzed with the use of an aqueous ceric ammonium molybdate stain or ethanolic vanillin and developed with a heat gun. Flash chromatography was performed using Silicycle Silicaflash® P60 unbonded grade silica.

Nuclear Magnetic Resonance Spectroscopy (NMR):

^{1}H, ^{13}C, and ^{19}F NMR were recorded at 25 ºC on an Agilent Technologies 400 MHz, a Bruker Avance III HD 400 MHz, a Bruker Avance NEO 500 MHz, a Varian Unity Inova 500 MHz, or a Varian Unity Inova 600 MHz spectrometer in $CDCl_3$ or DMSO-*d6* with residual $CHCl_3$ (^{1}H = 7.26 ppm, ^{13}C = 77.16 ppm) or DMSO (^{1}H = 2.54 ppm, ^{13}C = 40.45 ppm) as the internal standard. Deuterated solvents were purchased from Cambridge Isotope Laboratories. Chemical shifts are reported in parts per million (ppm). The data presented will be reported as follows; chemical shift, multiplicity (s = singlet, bs = broad singlet, d = doublet, dd = doublet of doublet, t = triplet, q = quartet, quin = quintet, m = multiplet), coupling constant (if applicable), and integration.

Mass Spectrometry (MS):

HRMS analysis (ESI-MS or CI-MS) was performed by the UC Santa Barbara mass spectrometry facility and UC Irvine mass spectrometry facility. ESI-MS analysis was performed on a Waters LCT Premier mass spectrometer equipped with an Alliance 2695 Separations module. EI-MS analysis was performed on a Waters GCT Premier mass spectrometer equipped with an Agilent 7890A GC oven and J&W Scientific DB-5ms+DG narrow bore column using helium carrier gas. MALDI TOF spectra were recorded using a Bruker Microflex LRF MALDI TOF instrument equipped with a 60 Hz nitrogen laser at 337 nm using a dithranol matrix and, in the case of the capped Savie polymer, NaTFA ionizing agent. Samples were measured in reflectron mode.

High-Performance Liquid Chromatography (HPLC):

HPLC analysis was performed on an Agilent 1220 series HPLC with columns indicated for each compound. HPLC-grade solvents were obtained from Fischer Scientific. Column conditions are specified for each relevant substrate (see SI Section 9).

Dynamic Light Scattering (DLS):

DLS data were collected using a Malvern Zetasizer Nano ZS equipped with a He-Ne, 4 mW, 633 nm red laser.

Fourier Transform Infrared Spectroscopy (FTIR):

FTIR spectra were obtained using a Perkin-Elmer Spectrum Two spectrometer equipped with a DTGS detector and diamond windows in the range of 4000-450 cm^{-1} at a resolution of 4 cm^{-1}.

Circular Dichroism (CD):

CD spectra were collected using a JASCO J-1500 spectropolarimeter (JASCO corporation, Tokyo, Japan). Details of individual experiments are outlined in SI Section 6.

Transmission Electron Microscopy (TEM):

TEM experiments were conducted at the Characterization Facility, University of Minnesota. Briefly, a 3 μl sample was applied to a 400-mesh ultra-thin carbon grid and incubated for 1

minute. The grid was subsequently blotted dry by a piece of filter paper. The TEM grid was imaged in an FEI Tecnai 300kV Field emission gun TEM on a Gatan Summit K2 direct electron detector camera.

2. Synthesis and Characterization of Savie

Monomer Synthesis:

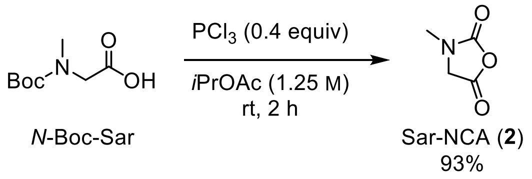

Supplementary Scheme S1: Synthesis of Sar-NCA (**2**)

The following protocol involving the cyclization of *N*-Boc-Sar is the preferred method on small scale (<100 mmol) from the standpoint of safety. On larger scales, where equipment permits, the method outlined by Barz and colleagues[3] involving the direct phosgenation of sarcosine may be favored from an atom economy standpoint.

To an oven-dried 100 mL round-bottom flask equipped with a PTFE-coated magnetic stir bar was added *N*-Boc-Sar (1 equiv, 40 mmol, 7.57 g) and *i*PrOAc (32 mL), then the solution was stirred until the substrate completely dissolved. To the flask was added PCl_3 (0.4 equiv, 16 mmol, 1.4 mL) and the reaction was allowed to stir at rt for 3 h. Upon completion, a solid residue of phosphorous acid had adhered to the walls of the flask, and the solution containing the product was decanted and filtered through Celite, then concentrated *in vacuo* to afford Sar-NCA (**2**) as a white crystalline solid (4.295 g, 93% yield). Solvent can be recovered at this stage. It is sometimes necessary to redissolve the monomer in *i*PrOAc and repeat the Celite filtration to remove any remaining phosphorous acid. Washing the product with hydrocarbons such as pentane, hexane, or heptane, followed by drying under high vacuum can hasten the removal of residual *i*PrOAc. Product was used within one hour or stored under argon and repurified before later use.

Surfactant Synthesis:

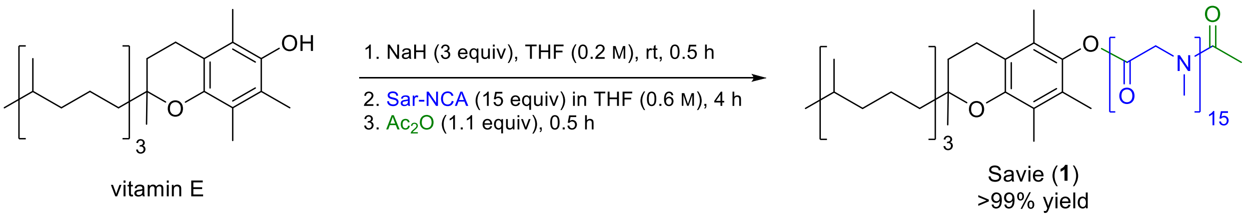

Supplementary Scheme S2: Synthesis of Savie (**1**)

Polymerization:

A time-lapse video of this polymerization process is included in Supporting Information 2.

To a flame-dried 100 mL flask equipped with a magnetic stir bar was added 60 wt % NaH in mineral oil (3 equiv, 2.4 mmol, 96 mg) in a glove box. The flask was sealed with a rubber septum and removed from the glove box, then placed on a stir plate and adapted to an argon/vacuum manifold. In a dry 5 mL volumetric flask capped with a rubber septum was added, though the septum, tocopherol (vitamin E; 1 mmol, 430.7 mg), then absolute THF was added up to the calibration line. The flask was shaken to dissolve the tocopherol, then 4 mL of this solution was withdrawn via syringe and added to the flask containing NaH to deliver 1 equiv (0.8 mmol) of tocopherol, at which time evolution of hydrogen gas was observed. The reaction was stirred gently for 30 minutes under positive pressure of argon. Meanwhile, to a dry 25 mL volumetric flask was added freshly-prepared Sar-NCA (15 mmol, 1.726 g), then the flask was capped with a rubber septum and the headspace was evacuated then backfilled with argon three times. Through the septum was added absolute THF up to the calibration line, then the flask was shaken to completely dissolve the monomer. At this point the monomer solution was completely clear; if cloudy, the monomer needs to be repurified prior to use. Once the tocopherol solution had stirred for 30 min, the monomer solution was withdrawn into a syringe which was then fitted to a syringe pump. The monomer solution was delivered at a rate such that 15 equiv of monomer (in this case, 20 mL) was delivered over the course of 2 h. Speed of stirring was gradually increased over the course of the addition to account for the increase in reaction volume while being careful not to allow the mixture to splash. Once addition of the monomer was completed, the reaction was allowed to stir for an additional 2 h, at which point a sample of the reaction solution was withdrawn, the solvent removed *in vacuo*, and subjected to FT-IR analysis to ensure full consumption of the monomer (disappearance of peaks at 1848 cm^{-1} and 1760 cm^{-1} corresponding to the anhydride carbonyls of Sar-NCA, and appearance of a peak at 1645 cm^{-1} corresponding to the amide carbonyls of the polysarcosine product, see Figs. S1 and S2). MALDI TOF analysis was then used to ensure correct polymer length and dispersity (the mode of the distribution should be 1496 Da, corresponding to tocopherol–$PSar_{15}$–H, i.e., uncapped Savie. Another distribution corresponding to $[M+Na]^+$ may also be present, and should have a mode of 1519 Da, see Figs. S3-S6 for MALDI TOF data of uncapped and capped Savie, and TPGS-750-M).

Capping:

To the polymerization solution was added Ac_2O (1.1 equiv, 0.88 mmol, 83 μL) and the reaction was stirred for 30 min. A small sample of the reaction was removed, from which the solvent was evaporated *in vacuo*, and the residue subjected to MALDI TOF analysis to ensure complete capping (mode of the distribution should be 1561 Da, corresponding to tocopherol–$PSar_{15}$–Ac + Na^+). If complete capping was not achieved, additional Ac_2O can be added and allowed to react for 30 min. The reaction solution was filtered to remove unreacted NaH and undissolved salts, then the filtrate was concentrated *in vacuo* to recover ca. 85% of the THF. Alternatively, 3 equiv AcOH

may be added and stirred for 1 h to quench NaH prior to filtration. The remaining solution was precipitated using heptane (2 mL), then either filtered or centrifuged to isolate the product which was then dried under high vacuum to obtain the product as a white amorphous solid (1.231 g, >99%). The E Factor for the synthesis of Savie was calculated to be E = 3 (*vide infra*).

Additional Information:

- Sodium hydride can be used without removal of the mineral oil
- The THF should be very dry, i.e., from a solvent purification system or ketyl still
- It is not necessary to pre-dry the *i*PrOAc or heptane
- *N*-Boc-Sar from chemical vendors is often impure and requires hot filtration with *i*PrOAc and subsequent recrystallization (from *i*PrOAc) to purify
- *i*PrOAc is interchangeable with EtOAc in terms of *N*-Boc-Sar purification and monomer synthesis, but *i*PrOAc was chosen as it is preferable from a green chemistry standpoint[4]
- The Sar-NCA should be used within one hour of purification
    - The monomer can be stored for up to 5 months under dry Ar at rt without significant degradation, but should be purified by dissolving in *i*PrOAc and filtering through Celite prior to use
- Volumetric flasks are not required; it is the ratio of initiator (α-tocopherol) to monomer (Sar-NCA) that determines the length of the polymer. However, when using a syringe pump, it is crucial to know the exact concentration of monomer so that exactly 15 equivalents can be delivered. On scales exceeding 1 mmol, a flame-dried addition funnel can be used and 15 equivalents of Sar-NCA can be added and dissolved in THF directly in the funnel
- Different polymer lengths can be accessed by adjusting the ratio of initiator to monomer. Polymer lengths >20 sarcosine repeat units are more difficult to access via this initiation method as the number of equivalents does not tend to correlate well to the polymer length beyond ca. 20 repeat units
- Good stirring is crucial during the reaction. After ca. 1.5-2 equiv of monomer have been added, the solution becomes a slurry. After ca. 4-6 equiv have been added, it forms a homogeneous solution again, at which point mixing is not as much of a concern. Reactions in excess of 4 mmol (w.r.t. tocopherol) are best performed with mechanical stirring
- An annotated time-lapse video of the polymerization process has been included as a separate portion of the SI to highlight different stages of the reaction (namely, the beginning homogeneous stage, intermediate slurry stage, and final homogeneous stage)
- It is crucial that monomer is added slowly (2 h addition time is sufficient), otherwise the reaction will not work. When monomer is added too quickly, an intractable organogel consisting of unusable polymerized material forms along with a sudden, uncontrolled release of CO_2
- The reaction evolves CO_2, so pressure release is crucial. Slow addition of the monomer ensures gentle gas evolution

- Polymers of Savie with an average n = 15 ± 1.5 sarcosine repeat units show equivalent yields in organic reactions to those with an exact 15 repeat unit average, and are therefore used in the present work without differentiating between, e.g., n = 14 and n = 15
- Savie is stable at rt indefinitely in its solid form, and for more than 3 months in aqueous solution. Over time, the ester linkage can be expected to hydrolyze in aqueous solution, but no change in reaction outcomes was observed when using 3-month-old 2 wt % solution
- Savie is somewhat hygroscopic so exposure of the solid material to humid air should be minimized
- Optionally, trituration with heptane or Et_2O can be employed to remove any remaining mineral oil (from the NaH) or unincorporated tocopherol from the capped polymer
- Average polymer length can also be determined using ^1H NMR (by comparing the multiplet from 4.47-3.84 ppm, corresponding to the PSar methylenes, to the tocopherol Ar-CH_3 peak at 1.95 ppm), however this is not preferred as it gives no indication of polydispersity
- Gel permeation chromatography (GPC) is commonly used to characterize polymers, however PSar polymers can only be characterized by GPC when 1,1,1,3,3,3-hexafluoroisopropanol (HFIP) is used as the mobile phase. As we do not have access to such an instrument, this method of characterization was not pursued for Savie. The combination of MALDI TOF and ^1H NMR spectroscopy was sufficient to characterize this molecule, and GPC was not anticipated to provide further insight

Additional Notes Regarding MALDI TOF Analysis:

- See SI pg. S10 for MALDI TOF procedures.
- The uncapped polymer ionizes readily and does not require an additive (e.g., NaTFA). The capped polymer, on the other hand, does require addition of NaTFA.
    - Higher molecular weight polymers are especially affected by low sodium concentrations, and thus if not enough Na^+ is present, the distribution will be biased toward the low molecular weight end. This can lead to confusion when, e.g., 15 average repeat units are observed for the uncapped polymer, but, e.g., 12 are seen with the capped polymer. The remedy is to add more NaTFA to enable full ionization of the capped polymer (we frequently add 1.5 μL of a 10 mg/mL solution of NaTFA in THF to a 20 μL solution of our sample containing polymer and dithranol matrix).
    - A very small amount of uncapped polymer is always present following the capping procedure, and because the uncapped material is readily ionized, MALDI samples that do not contain enough Na^+ to fully ionize the capped material will appear to contain a significant amount of uncapped material. However, when more Na^+ is added, enough capped polymer will be ionized to demonstrate that only a nearly imperceptible amount of the uncapped material (with respect to capped polymer) is truly present in the sample.

FT-IR Spectra of Sar-NCA and Savie:

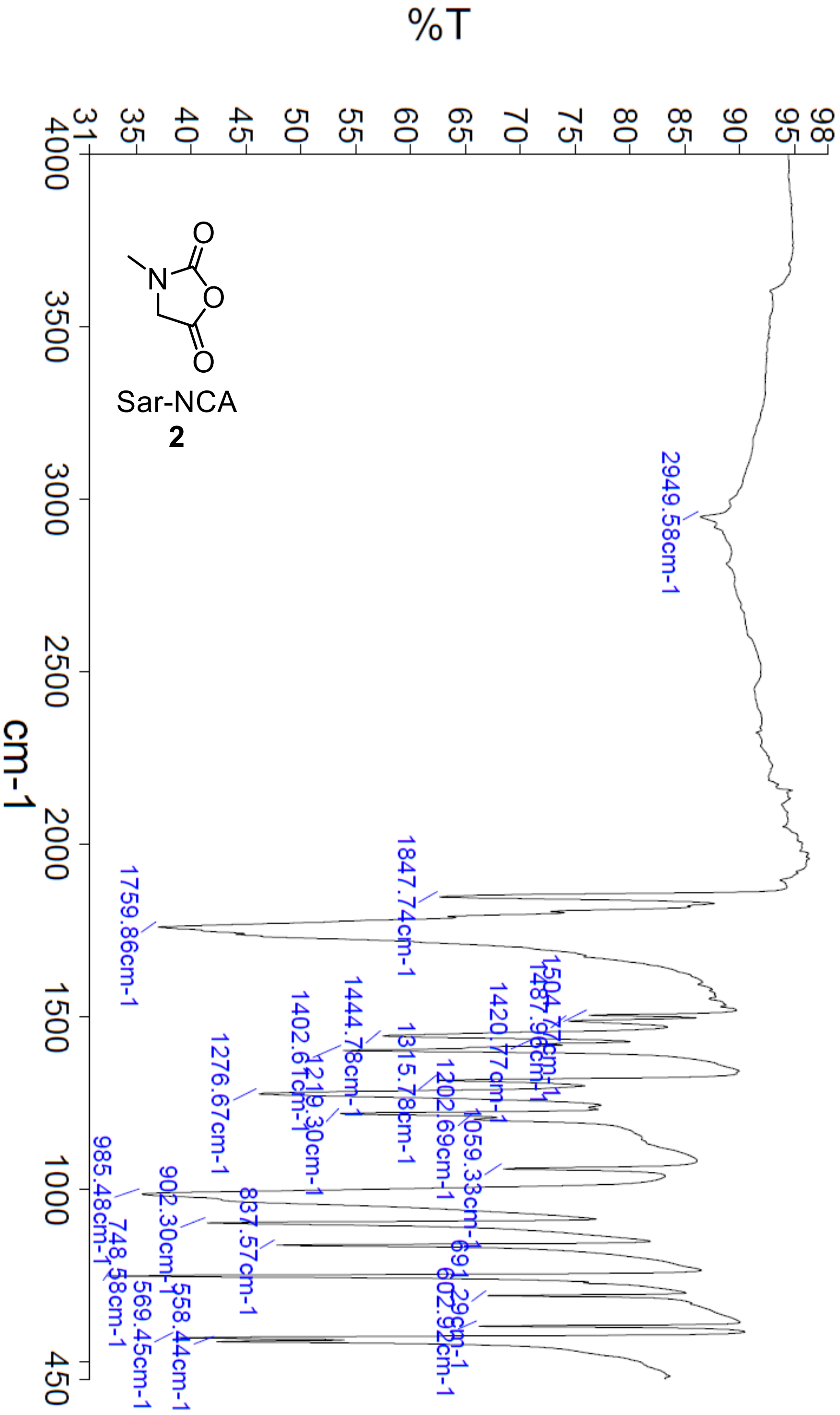

Supplementary Figure S1: IR spectrum of Sar-NCA (2). This spectrum shows the characteristic anhydride carbonyl peak at 1760 cm^{-1}

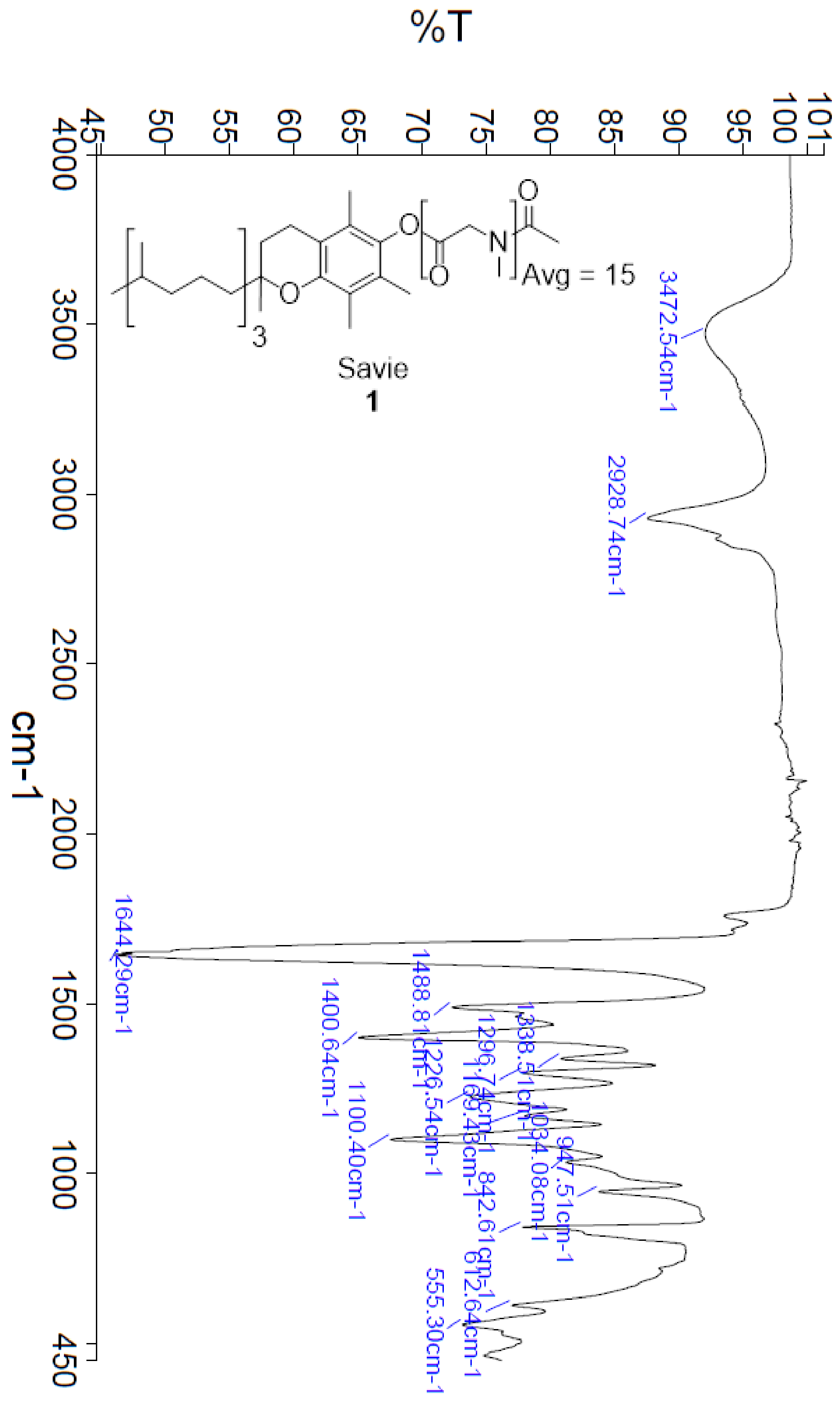

Supplementary Figure S2: IR spectrum of Savie (1). This spectrum shows the characteristic amide carbonyl peak at 1644 cm^{-1}

MALDI TOF Spectra and Polydispersity of Uncapped and Capped Savie and TPGS-750-M:

Sample Preparation:

Uncapped Savie: 5 mg of sample was dissolved in 100 μL of $CHCl_3$, then diluted 40:1 with dithranol/$CHCl_3$

Capped Savie and TPGS-750-M: 5 mg of sample was dissolved in 100 μL of THF, then diluted 40:1 with dithranol/THF containing NaTFA ionizing agent

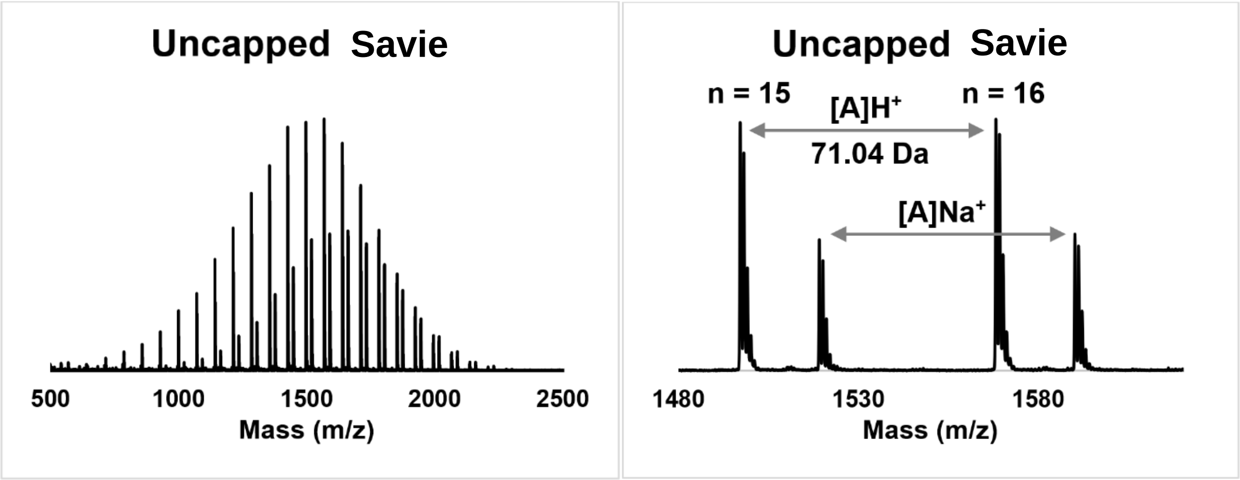

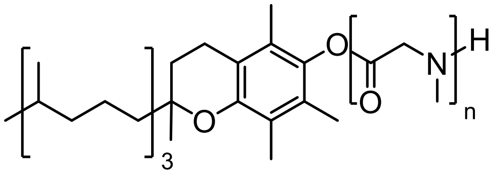

m/z = 429.37+(71.04)15+1.01+1.01 = 1496.99 **$[A]H^+$**

m/z = 429.37+(71.04)15+1.01+22.99 = 1518.97 **$[A]Na^+$**

Supplementary Figure S3: MALDI TOF spectrum of uncapped Savie showing the characteristic set of ions

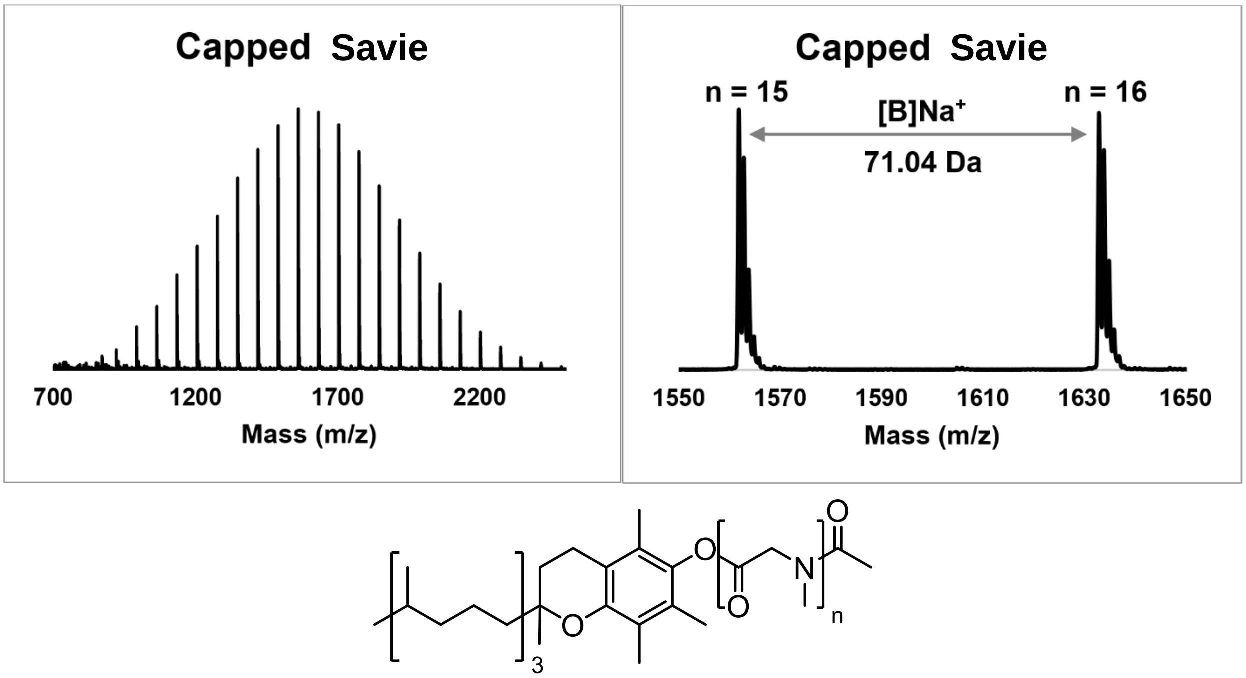

m/z = 429.37+(71.04)15+43.02+22.99 = 1560.98 **[B]Na⁺**

Supplementary Figure S4: MALDI TOF spectrum of capped Savie showing the characteristic set of ions

Polydispersity of Capped Savie:

M_n = 1634.21 Da

$Đ$ = 1.03

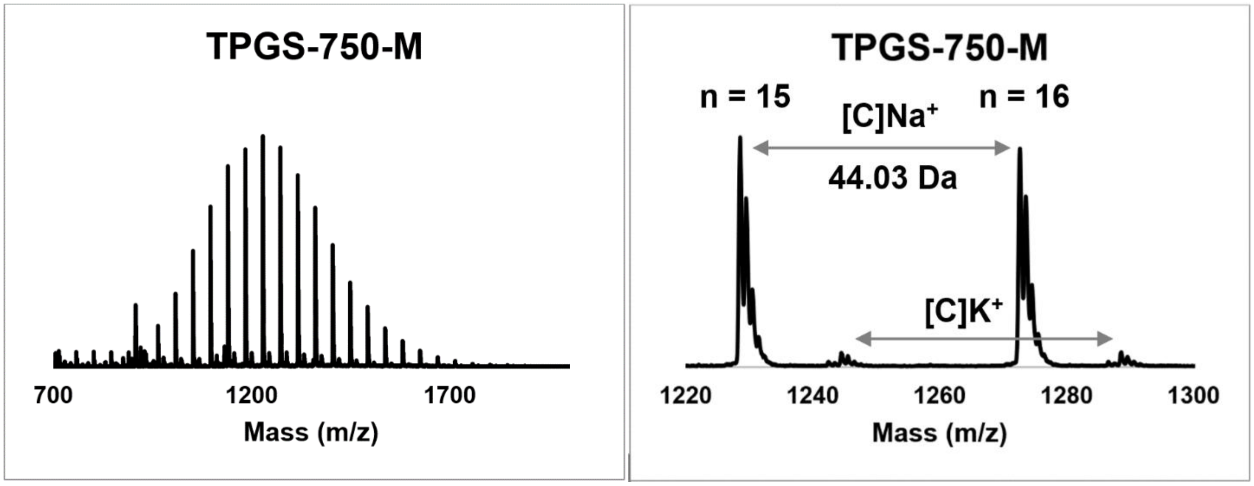

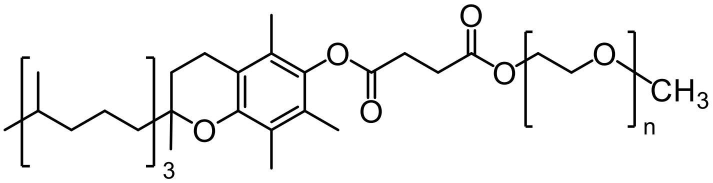

m/z = 429.37+100.02+(44.03)15+15.02+22.99 = 1227.85 **$[C]Na^+$**

m/z = 429.37+100.02+(44.03)15+15.02+38.96 = 1243.82 **$[C]K^+$**

Supplementary Figure S5: MALDI TOF spectrum of TPGS-750-M showing the characteristic set of ions

Polydispersity of TPGS-750-M:

M_n = 1204.78 Da

$Đ$ = 1.06

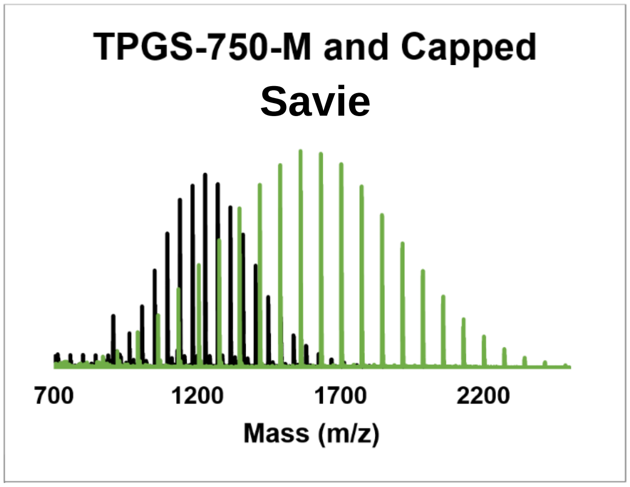

Supplementary Figure S6: Combined MALDI TOF spectra of TPGS-750-M (shown in black) and Savie (shown in green)

E Factor Calculations:

Savie

Scale = 1 mmol

Yield = 1.54 g

Waste:

THF (assumes 85% recovery) = 4.35 mL = 3.8628 g

NaH (including mineral oil) = 0.096 g

H_2 = 0.002 g

CO_2 = 0.6602 g

Unincorporated Ac_2O = 0.0102 g

Acetate = 0.0591 g

Total = 4.6903 g

E Factor = total waste/total product = 4.6903/1.54 = 3

TPGS-750-M[1]

Scale = 154.1 mmol

Yield = 174.11 g

Waste:

Tocopherol succinate synthesis:

Unincorporated tocopherol = 2.0575 g

Excess + unincorporated succinic anhydride = 8.1734 g

CH_2Cl_2 = 798 g

DMAP = 9.4 g

Et_3N = 15.609 g

1 M aqueous HCl = 480 g

H_2O = 200 g

Saturate brine = 300.5 g

TPGS-750-M synthesis:

Unincorporated tocopherol succinate = 1.4205 g

Excess tocopherol succinate = 0.3291 g

Unincorporated MPEG-750-M = 1.971 g

Toluene = 485.52 g

p-toluenesulfonic acid = 3.01 g

H_2O = 2.3361 g

Total = 2308.3266 g

E Factor = total waste/total product = 2308.3266/174.11 = 13

3. Polysarcosine (PSar) Polymer Length Optimization

The optimal length of the polysarcosine (PSar) portion of Savie was determined to be 15 sarcosine repeat units by performing a series of reactions using tocopheryl polysarcosinate (ToPSar) polymers with average PSar lengths of n = 5, 10, 15 (i.e., Savie), and 20 sarcosine repeat units. These polymers were synthesized by varying the number of equivalents of Sar-NCA used during the polymerization (*vide supra*). Comparisons were made to pure water as well as 2 wt % aqueous solutions of several other surfactants, including TPGS-750-M.

Supplementary Table S1: S_NAr reaction in various media

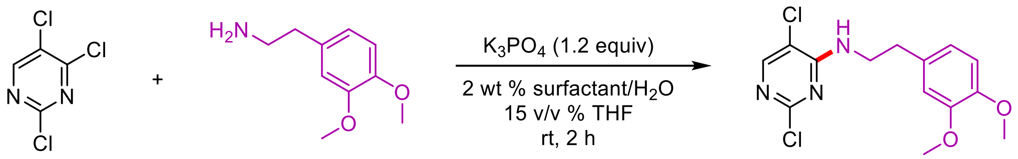

entry	surfactant	isolated yield (%)
1	none (DI H_2O)	79
2	TPGS-750-M	82
3	Brij 30	82
4	Coolade	75
5	ToPSar, n = 5	75
6	ToPSar, n = 10	79
**7**	**Savie (n = 15)**	**85**
8	ToPSar, n = 20	82

Procedure: To a 1 dr vial equipped with a PTFE-coated magnetic stir bar was added K_3PO_4 (1.2 equiv, 0.24 mmol, 51 mg), then the vial was sealed with a rubber septum and evacuated and backfilled with argon three times using an argon/vacuum manifold. A 2 wt % aqueous solution of the respective surfactant (0.34 mL) was added under a flow of argon followed by 2,4,5-trichloropyrimidine (1 equiv, 0.2 mmol, 23 μL), THF (15 v/v %, 60 μL), and 2-(3,4-dimethoxyphenyl)ethan-1-amine (1 equiv, 0.2 mmol, 34 μL). The vial was sealed and allowed to stir at rt for 2 h. Upon completion, the reaction mixture was extracted with EtOAc (3 x 1 mL), the organic layers were combined, washed with brine, dried over anhydrous $MgSO_4$, and concentrated *in vacuo*. Samples were then purified by flash chromatography (25% EtOAc/hexanes).

Of the polysarcosine surfactants, n = 15 (i.e., Savie) performed best, and in fact it was the only reaction that performed better than pure water (outside of an expected experimental error of ± 5% yield).

Supplementary Table S2: Amide bond formation in various media

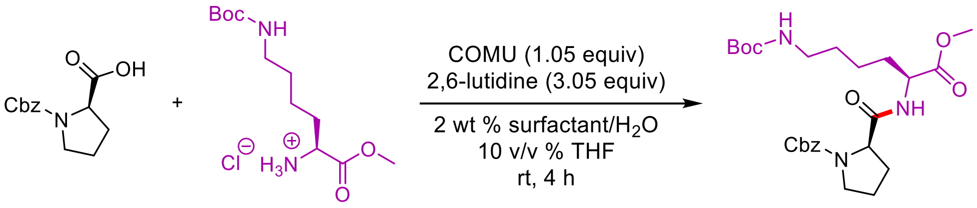

entry	surfactant	isolated yield (%)
1	none (DI H_2O)	70
2	TPGS-750-M	73
3	Brij 30	79
4	Coolade	83
5	ToPSar, n = 5	81
6	ToPSar, n = 10	73
**7**	**Savie (n = 15)**	**82**
8	ToPSar, n = 20	80

Procedure: To a 1 dr vial equipped with a PTFE-coated magnetic stir bar was added Z-Pro-OH (1 equiv, 0.5 mmol, 125 mg), HCl•H-Lys(Boc)-OMe (1 equiv, 0.5 mmol, 148 mg), a 2 wt % aqueous solution of the respective surfactant (0.9 mL), THF (10 v/v %, 100 µL), 2,6-lutidine (3.05 equiv, 1.525 mmol, 177 µL), and COMU (1.05 equiv, 0.525 mmol, 225 mg). The vial was sealed and allowed to stir at rt for 4 h. Upon completion of the reaction, the mixture was extracted with EtOAc (3 x 1 mL) and the organic layers were combined, then washed with 1 M aqueous HCl (3 x 3 mL), then washed with a 1:1 mixture of sat. aqueous $NaHCO_3$ and water (until the aqueous layer stopped turning yellow), followed by washing with brine, drying over anhydrous $MgSO_4$, and concentrating *in vacuo*. Samples were then purified by flash chromatography (80% EtOAc/hexanes).

The PSar surfactant with n = 15 (i.e., Savie) performed significantly better than pure water as well as TPGS-750-M. It performed comparably to PSar surfactants with n = 5 and n = 20 repeat units, but surprisingly outperformed that with n = 10.

Supplementary Table S3: Pd-catalyzed Suzuki-Miyaura cross-coupling in various media

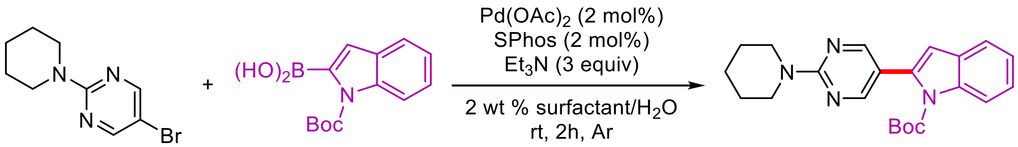

entry	surfactant	isolated yield (%)
1	pure H_2O	37
2	TPGS-750-M	77
3	Brij 30	76
4	Coolade	68
5	ToPSar, n = 5	59
6	ToPSar, n = 10	61
**7**	**Savie (n = 15)**	**74**
8	ToPSar, n = 20	65

Procedure: To a 1 dr vial equipped with a PTFE-coated magnetic stir bar was added $Pd(OAc)_2$ (2 mol %, 0.004 mmol, 0.9 mg), SPhos (2 mol %, 0.004 mmol, 1.6 mg), 5-bromo-2-(piperidin-1-yl)pyrimidine (1 equiv, 0.2 mmol, 48 mg), and (1-(*t*-butoxycarbonyl)-1*H*-indol-2-yl)boronic acid (1 equiv, 0.2 mmol, 52 mg). The vial was sealed with a rubber septum and evacuated and backfilled with argon three times using an argon/vacuum manifold. A 2 wt % aqueous solution of the respective surfactant (0.4 mL) was added under a flow of argon followed by Et_3N (3 equiv, 0.6 mmol, 84 μL) and the reaction was allowed to stir at rt for 2 h. Upon completion of the reaction, the mixture was extracted with EtOAc (3 x 1 mL) and the organic layers were combined and washed with brine, dried over anhydrous $MgSO_4$, and concentrated *in vacuo*. Samples were then purified by flash chromatography (15% EtOAc/hexanes).

All reactions containing surfactant significantly outperformed the reaction using pure water owing to the reactants forming a clump on the stir bar in the latter, and homogeneous emulsions in the former. Among the surfactants, the PSar surfactant with n = 15 (i.e., Savie), TPGS-750-M, and Brij 30 performed the best with all other surfactants leading to significantly lower yields.

4. Dynamic Light Scattering (DLS) Spectra of Savie with Varying PSar Lengths

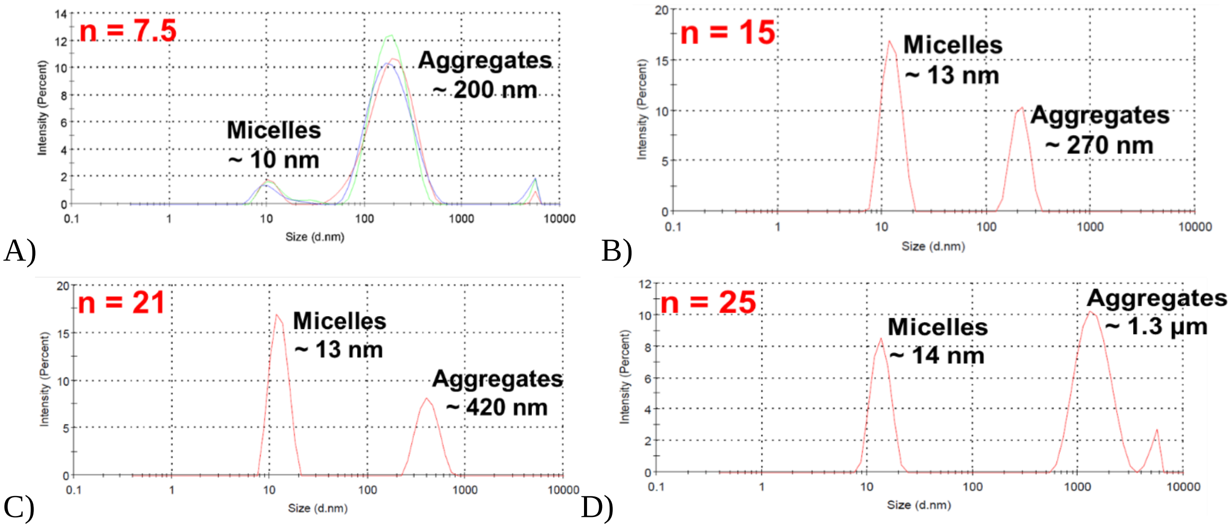

Supplementary Figure S7: DLS spectra of 2 wt % solutions of polysarcosine surfactants with PSar monomer repeat units equal to A) 7.5: this spectrum shows a small proportion of individual micelles with an average diameter of ca. 10 nm and a much larger proportion of micelles in the form of ca. 200 nm aggregates. B) 15 (i.e., Savie): this amphiphile demonstrates a greater proportion of micelles as individual particles with ca. 13 nm diameter, and aggregates with ca. 270 nm diameter. C) 21: individual micelles are present in roughly the same proportion and size as with n = 15, however the micelle aggregates are larger at ca. 420 nm. D) 25: at this size, aggregates again become the dominant species, but are much larger at ca. 1.3 μm, while individual micelles remain roughly the same size as with shorter length polymers.

5. Temperature and Salt Stability Comparison Between Aqueous Savie and TPGS-750-M

Salt Stability:

The stability, i.e., ability to resist flocculation, of aqueous solutions of 2 wt % TPGS-750-M/H_2O and 2 wt % Savie/H_2O were evaluated by subjecting them to increasing concentrations of K_3PO_4 at rt. To 1 dr vials was added the respective quantity of base, followed by addition of 0.5 mL surfactant solution and the mixtures were vortexed for 15 seconds each.

Supplementary Table S4: Salt stability experiments show that 2 wt % Savie/H_2O does not flocculate until the K_3PO_4 concentration exceeds 0.45 M. 2 wt % TPGS-750-M/H_2O begins flocculating around 0.35 M

K_3PO_4 conc. (M)	Appearance of 2 wt % TPGS-750-M/H_2O solution	Appearance of 2 wt % Savie/H_2O solution
0.25	clear gel, no flocculation	clear solution, no flocculation
0.30	clear gel, no flocculation	clear solution, no flocculation
0.35	turbid gel, slight flocculation	clear solution, no flocculation
0.40	turbid gel, slight flocculation	clear solution, no flocculation
0.45	turbid solution, mostly flocculated	slightly turbid, no flocculation
0.50	clear solution, fully flocculated	turbid solution, slight flocculation

Temperature Stability/Cloud Point:

The cloud point of aqueous 2 wt % TPGS-750-M and Savie solutions were tested by adding 1 mL of the respective surfactant to a 1 dr vial and heating to various temperatures in an aluminum heating block mounted on a heating stir plate.

A) 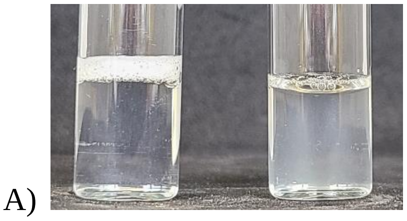 B) 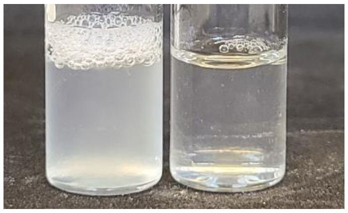 C) 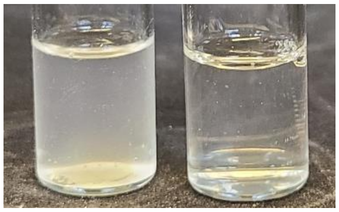

Supplementary Figure S8: Aqueous 2 wt % solutions of TPGS-750-M (left in each image) and Savie (right in each image) at varying temperatures demonstrating a cloud point effect in TPGS-750-M, and lack thereof in Savie. A) 21 °C; B) 75 °C, note cloudy TPGS-750-M solution; C) 100 °C, note all TPGS-750-M has fallen out of solution and coalesced as an oil at the bottom of the vial.

It is worth noting that the 2 wt % Savie/H_2O (unlike the aqueous solution of TPGS-750-M) still foamed when shaken at 100 °C, indicating that it retained its surfactant properties well above the temperatures typically accessible by PEGylated surfactants.

6. Enzyme Denaturation Experiments Using Circular Dichroism (CD)

Preparation of buffer and surfactant/buffer solutions:

Aqueous 1 M stock solutions of potassium phosphate monobasic (**A**) and potassium phosphate dibasic (**B**) were prepared. A pH 7 phosphate buffer solution was then prepared by mixing 38.5

mL of solution **A** with 61.5 mL of solution **B**. The pH was controlled and adjusted, if needed, with a 1 M aqueous solution of NaOH or HCl. The buffer solution was diluted with HPLC grade water to 0.1 M for ERED and ADH, and 0.01 M for lipase. 0.03 wt % of solid surfactant was dissolved to make surfactant/buffer solutions (lower wt % was used for CD spectra than in biocatalytic reactions as a strong absorbance was observed in the far UV for TPGS-750-M, affecting the signal of the helix portion of the protein).

Sample Preparation:

ERED:

- Sample 1: buffer solution (for blank)
- Sample 2: 0.03 wt % TPGS-750-M/buffer solution (for blank)
- Sample 3: 0.03 wt % Savie/buffer solution (for blank)
- Sample 4: 4.2 mg ERED-103 in 1 mL of the buffer solution
- Sample 5: 4.2 mg ERED-103 in 1 mL of the 0.03 wt % TPGS-750-M/buffer solution
- Sample 6: 4.2 mg ERED-103 in 1 mL of the 0.03 wt % Savie/buffer solution

ADH:

- Sample 1: buffer solution (for blank)
- Sample 2: 0.03 wt % TPGS-750-M/buffer solution (for blank)
- Sample 3: 0.03 wt % Savie/buffer solution (for blank)
- Sample 4: 2.6 mg ADH101 in 1 mL of the buffer solution
- Sample 5: 2.6 mg ADH101 in 1 mL of the 0.03 wt % TPGS-750-M/buffer solution
- Sample 6: 2.6 mg ADH101 in 1 mL of the 0.03 wt % Savie/buffer solution

Lipase:

- Sample 1: buffer solution (for blank)
- Sample 2: 0.03 wt % TPGS-750-M/buffer solution (for blank)
- Sample 3: 0.03 wt % Savie/buffer solution (for blank)
- Sample 4: 10 µL palatase20000L in 1 mL of the buffer solution
- Sample 5: 10 µL palatase20000L in 1 mL of the 0.03 wt % TPGS-750-M/buffer solution
- Sample 6: 10 µL palatase20000L in 1 mL of the 0.03 wt % Savie/buffer solution

The analysis required a lower concentration of enzymes than in the reaction. The spectra are presented in Figs. S9-S11.

CD Parameters:

Spectra were collected using a JASCO J-1500 spectropolarimeter (JASCO corporation, Tokyo, Japan) with a 0.1 mm pathlength, U-shaped quartz cuvette. The following conditions were employed: scanning speed 50 nm/min, band width 1 nm, and 3 accumulations per sample. CD protein spectra were corrected for the corresponding buffer or surfactant/buffer signal.

CD Spectra:

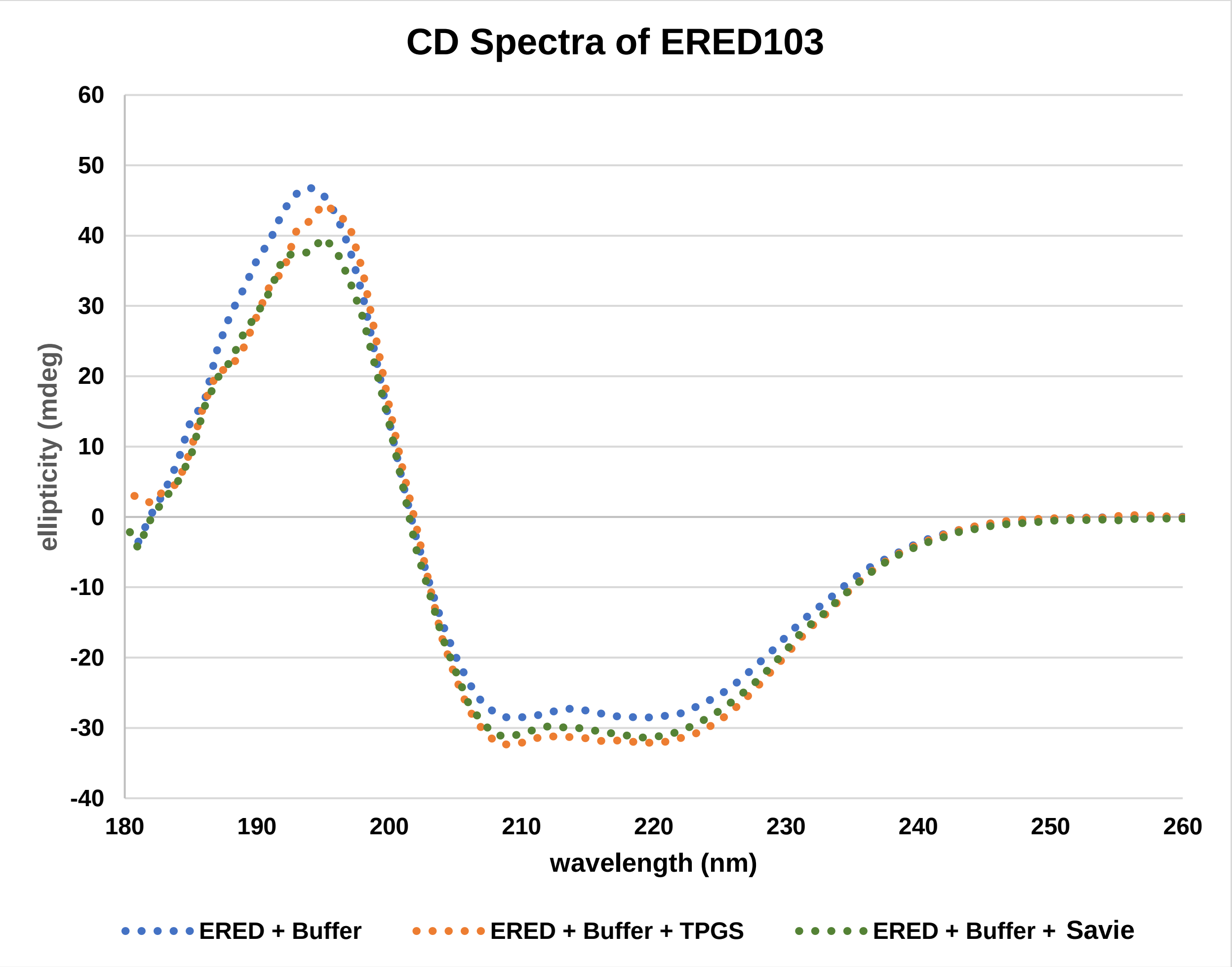

Supplementary Figure S9: CD spectra of ERED-103. This spectrum shows the secondary structure of ERED-103 in aqueous buffer solution (blue line), 0.03 wt % TPGS-750-M/buffer (orange line), and 0.03 wt % Savie/buffer (green line). All spectra are characteristic of proteins with both α-helical and β-sheet components, indicating the secondary structure has been conserved in the presence of both surfactants.

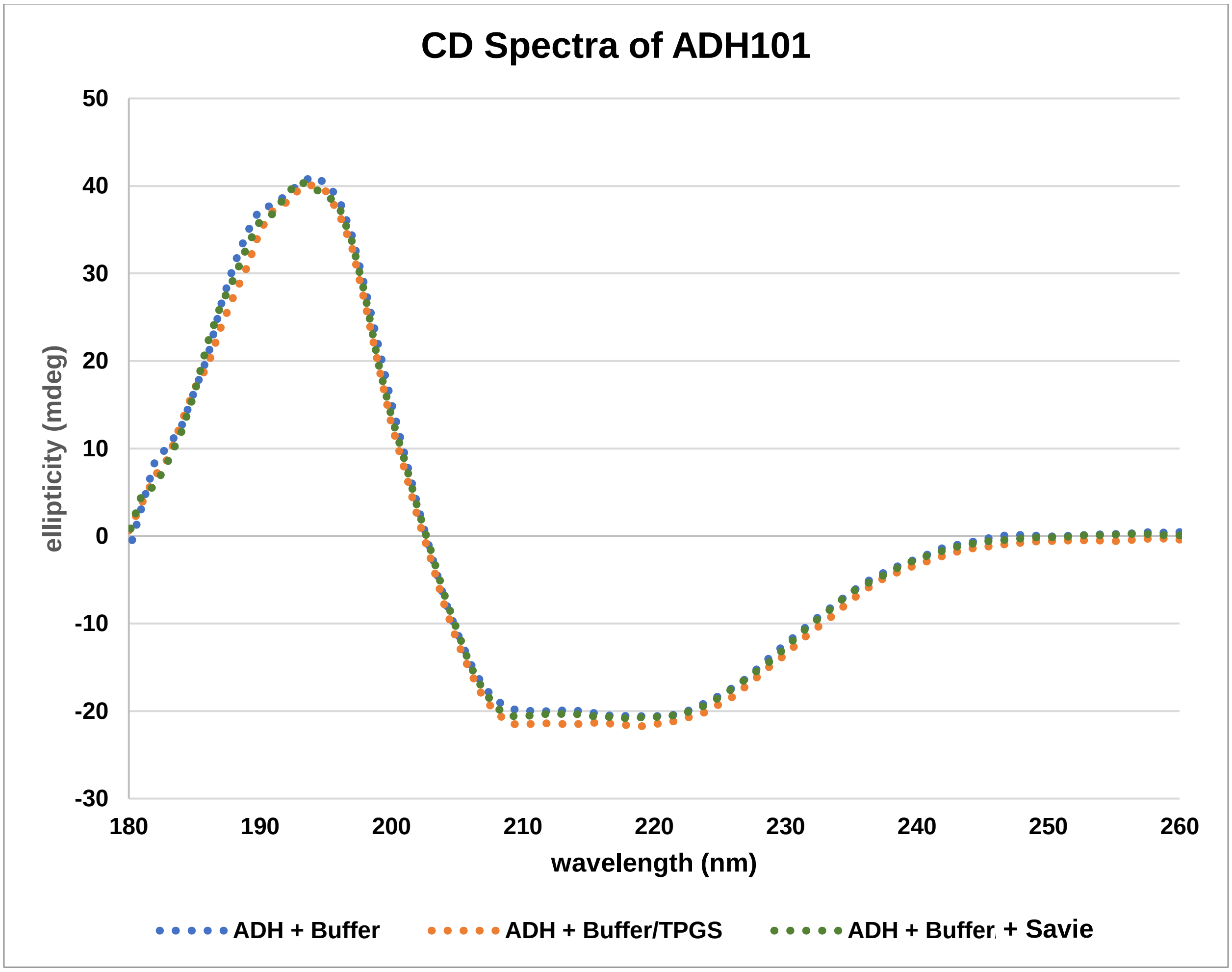

Supplementary Figure S10: CD spectra of ADH101. This spectrum shows the secondary structure of ADH101 in aqueous buffer solution (blue line), 0.03 wt % TPGS-750-M/buffer (orange line), and 0.03 wt % Savie/buffer (green line). All spectra are characteristic of proteins with both α-helical and β-sheet components, indicating the secondary structure has been conserved in the presence of both surfactants.

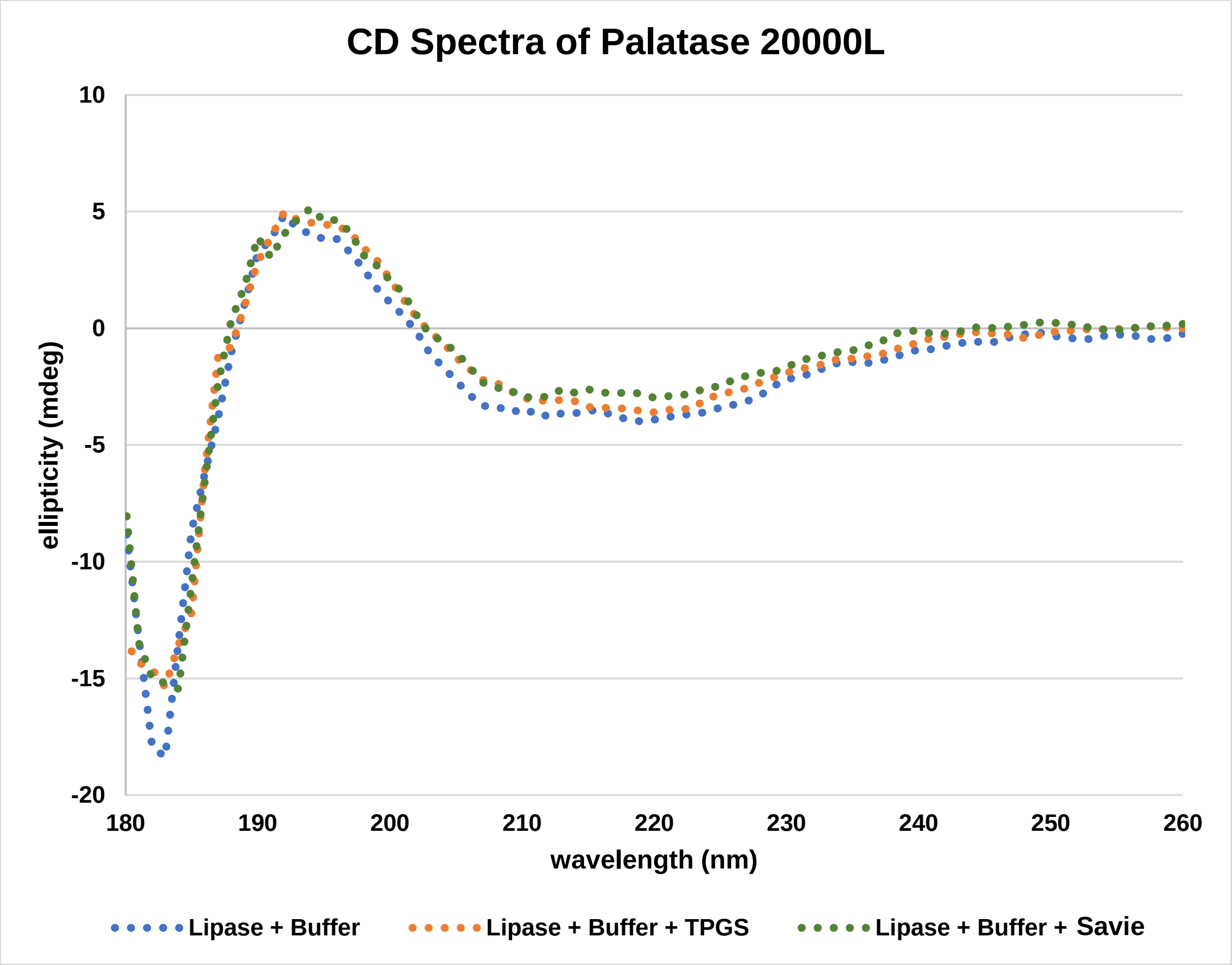

Supplementary Figure S11: CD spectra of palatase 20000L. This spectrum shows the secondary structure of palatase 20000L in aqueous buffer solution (blue line), 0.03 wt % TPGS-750-M/buffer (orange line), and 0.03 wt % Savie/buffer (green line). All spectra are characteristic of proteins with both α-helical and β-sheet components, indicating the secondary structure has been conserved in the presence of both surfactants.

7. Procedures for Reactions Performed in Aqueous Savie vs. TPGS-750-M

7.1 Ppm Pd-Catalyzed Suzuki-Miyaura Coupling (SMC) Reactions

Catalyst Stock Solution Preparation (N_2Phos):

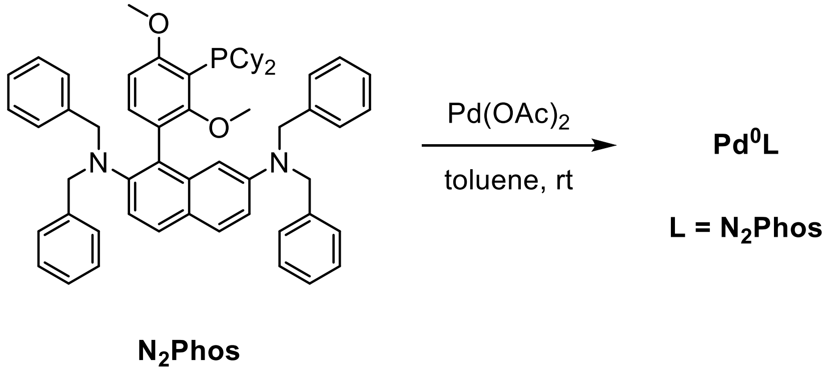

Supplementary Scheme S3: Preparation of Pd(N_2Phos) catalyst

To an oven-dried 1 dr vial equipped with a PTFE-coated magnetic stir bar was added $Pd(OAc)_2$ (0.01 mmol, 2.3 mg) and N_2Phos (0.018 mmol, 14.8 mg). The vial was sealed with a rubber septum, then evacuated and backfilled with argon three times using an argon/vacuum manifold. Anhydrous degassed toluene (1 mL for 1000 ppm reactions [i.e., those employing aryl bromides], 0.8 mL for 2500 ppm reactions [i.e., those employing aryl chlorides]) was added and the mixture was allowed to stir for 15 minutes. At this point the catalyst was ready and could be added to the reaction mixture.

General procedure for SMC reactions in aqueous Savie or TPGS-750-M (N_2Phos):

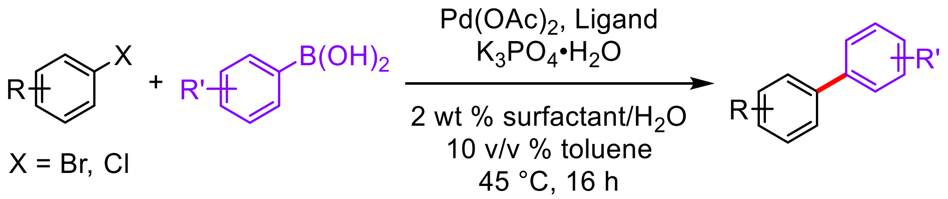

Supplementary Scheme S4: General procedure for SMC reactions using Pd(N_2Phos) in surfactant media

For reactions containing co-solvent: To an oven-dried 1 dr vial equipped with a PTFE-coated magnetic stir bar was added aryl halide (1 equiv, 0.5 mmol), organoboron (1.5 equiv, 0.75 mmol), and potassium phosphate (1.5 equiv, 0.75 mmol). The vial was capped with a rubber septum and

sealed with PTFE tape. The reaction vial was evacuated and backfilled with argon three times using a vacuum/argon manifold. A 2 wt % aqueous solution of surfactant (0.9 mL) was added, followed by the catalyst solution (0.1 mL), both via syringe. The reactions were allowed to stir in an aluminum heating block over a stir plate with stir rate set to >1000 rpm with a thermocouple probe in the aluminum block set to 48 °C (this gave a temperature of 45 °C in the reaction vial). The reactions were monitored by TLC analysis. Upon completion of the reaction, the vial was cooled to rt and the mixture was extracted with MTBE, then the organic layer was washed with brine three times and dried over anhydrous Na_2SO_4. The mixture was concentrated *in vacuo* and purified by flash chromatography (see SI Section 9 for column conditions and yields of individual compounds).

For co-solventless reactions: An oven-dried 1 dr vial equipped with a PTFE-coated magnetic stir bar was capped with a rubber septum. The vial was evacuated and backfilled with argon three times using a vacuum/argon manifold, then the catalyst solution (0.1 mL) was added via syringe. Toluene was removed under vacuum, then aryl chloride (1 equiv, 0.5 mmol), organoboron (1.5 equiv, 0.75 mmol), and potassium phosphate (1.5 equiv, 0.75 mmol) were added quickly under a constant stream of argon. The septum was replaced, and the vial was sealed with PTFE tape and once again evacuated and backfilled three times. A 2 wt % aqueous solution of surfactant (1 mL) was then added via syringe and the reactions were allowed to stir in an aluminum heating block over a stir plate with stir rate set to >1000 rpm with a thermocouple probe in the aluminum block set to 48 °C (this gave a temperature of 45 °C in the reaction vial). The reactions were monitored by TLC analysis. Upon completion of the reaction, the vial was cooled to rt and the mixture was extracted with MTBE, then the organic layer was washed with brine three times and dried over anhydrous Na_2SO_4. The mixture was concentrated *in vacuo* and purified by flash chromatography (see SI Section 9 for column conditions and yields of individual compounds).

Procedure for SMC reaction in aqueous Savie and TPGS-750-M (Arylex Precursor **5**):

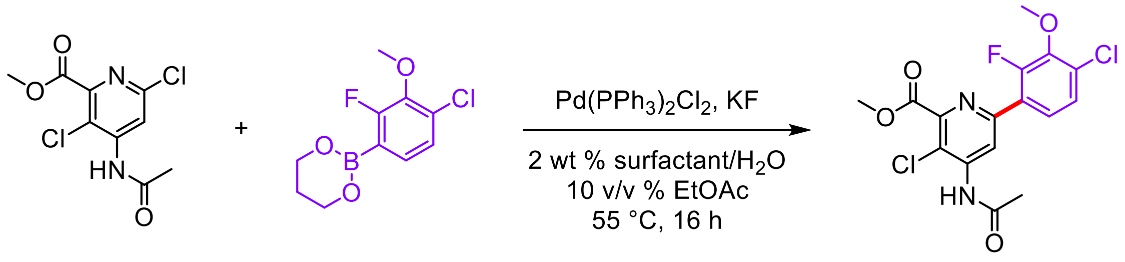

5
Savie: **94%**
TPGS-750-M: 82%

Supplementary Scheme S5: SMC reaction in micellar media to afford Arylex precursor **5**

To a 1 dram vial equipped with a PTFE-coated magnetic stir bar was added the aryl chloride (1 equiv, 0.5 mmol, 131 mg), the boronic ester (1.2 equiv, 0.6 mmol, 147 mg), KF (2 equiv, 1.0 mmol, 58 mg), and $Pd(PPh_3)_2Cl_2$ (2500 ppm, 0.9 mg). The vial was sealed with a rubber septum and evacuated and backfilled with argon three times using an argon/vacuum manifold. Under a positive flow of argon, a 2 wt % aqueous solution of surfactant (0.9 mL) and EtOAc (0.1 mL) were added via syringe, and the vial was stirred at 55 °C in an aluminum heating block mounted on a stir plate until completion (as monitored by TLC). The products were separated by filtration and purified by flash chromatography (gradient from 10-60% EtOAc/hexanes) to afford **5** as a white solid (Savie: 182 mg, 94% yield; TPGS-750-M: 158 mg, 82% yield).

Procedure for the gram-scale SMC reaction in aqueous Savie and TPGS-750-M (sonidegib precursor **6**):

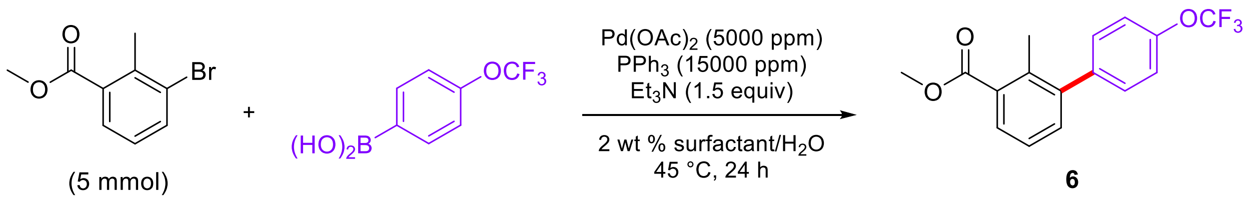

6
Savie: **95%**
TPGS-750-M: 85%

Supplementary Scheme S6: Gram-scale SMC to afford sonidegib precursor **6**

In an argon-filled glovebox, to a flame-dried 25 mL round-bottom flask equipped with a PTFE-coated magnetic stir bar was added $Pd(OAc)_2$ (0.5 mol %, 5.6 mg) and PPh_3 (1.5 mol %, 19.7 mg), then the flask was sealed with a rubber septum and removed from the glovebox. Through the septum, via syringe, was added degassed anhydrous THF (2 mL) and the mixture was allowed to stir gently for 5 min to ligate the Pd. The THF was then carefully remove under vacuum. The septum was removed and then, under a constant flow of argon, the aryl bromide (1 equiv, 5 mmol, 1.1454 g), aryl boronic acid (1.5 equiv, 7.5 mmol, 1.5445 g), and $K_3PO_4 \cdot H_2O$ (1.5 equiv, 7.5 mmol, 1.7271 g) were added, then the septum was replaced, and the headspace was evacuated and refilled with argon three times. With the flask still adapted to the Schlenk manifold under argon pressure, a 2 wt % aqueous surfactant solution was added via syringe (10 mL), then the reaction was allowed to stir vigorously in an oil bath at 45 °C for 24 h. Upon completion, the reaction mixture was loaded onto a Celite plug (ca. 8 cm) and eluted with 10% EtOAc/hexanes, then the solvent was removed *in vacuo*. The product was isolated by column chromatography (0-2% EtOAc/hexanes) and **6** was obtained as a colorless oil (Savie: 1.4734 g, 95%; TPGS-750-M: 1.3204 g, 85%).

Synthesis of relevant starting materials:

Indomethacin isobutyl ester:

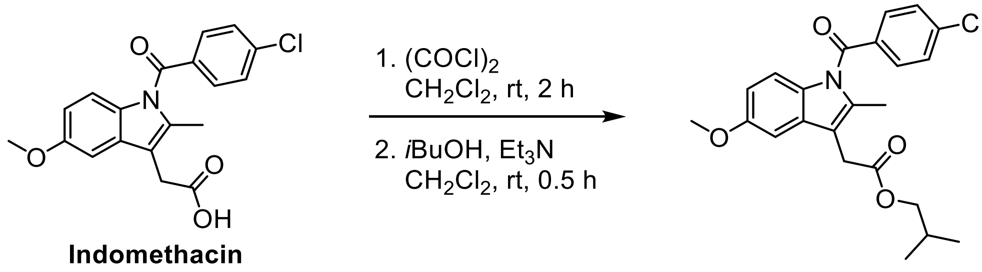

Supplementary Scheme S7: Esterification to afford indomethacin isobutyl ester

Acyl chloride formation: To a flame-dried 100 mL round bottom flask equipped with a PTFE-coated magnetic stir bar was added indomethacin (1 equiv, 6 mmol, 2.1467 g) and CH_2Cl_2 (30 mL). To the solution was added oxalyl chloride (1.2 equiv, 7.2 mmol, 0.69 mL) dropwise at rt, then the reaction was capped with a rubber septum and allowed to stir at rt for an additional 2 h. Upon completion, the solvent and excess oxalyl chloride were removed *in vacuo,* then re-dissolved in 15 mL of CH_2Cl_2.

Esterification: In a separate 20 mL vial, *i*BuOH (1.2 equiv, 7.2 mmol, 0.67 mL) and Et_3N (1.2 equiv, 7.2 mmol, 1.0 mL) were dissolved in dichloromethane (15 mL), then this solution was slowly added to the acyl chloride solution through the septum via syringe with the use of a vent needle to allow for evolution of HCl gas, then the reaction was allowed to stir at rt for 30 minutes. Upon completion, the reaction was transferred to a separatory funnel and washed with DI water (3 x 50 mL), then brine (1 x 25 mL), then the organic layer was dried over anhydrous $MgSO_4$, filtered, and concentrated *in vacuo*. The crude material was purified via flash chromatography (15 % EtOAc/hexanes) to yield indomethacin isobutyl ester as a yellow solid (1.1161 g, 45%).

7.2 Ppm Pd-Catalyzed Amination Reactions

Catalyst stock solution preparation:

To a 1 dr vial in an argon-filled glove box was added tBuXPhosPd(allyl)OTf (9 mg) and the vial was capped with a rubber septum. The vial was removed from the glove box and degassed anhydrous THF (1 mL) was added though the septum via syringe to make a 12.3 µmol/mL stock solution.

General procedure for Pd-catalyzed amination reactions:

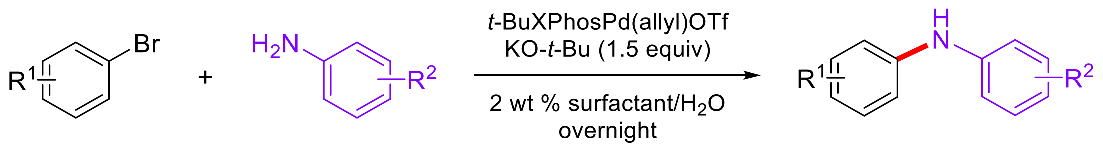

Supplementary Scheme S8: General procedure for amination reactions in surfactant media

To a 1 dr vial equipped with a PTFE-coated magnetic stir bar was added stock solution (40 µL to deliver 1000 ppm Pd, or 60 µL to deliver 1500 ppm Pd), then the solvent was removed *in vacuo* and the atmosphere was replaced with argon. The vial was taken into an argon-filled glove box where aryl bromide (1 equiv, 0.5 mmol), amine (1.25 equiv, 0.625 mmol), and KO*t*Bu (1.5 equiv, 0.75 mmol, 84 mg) were added. The vial was re-capped with the septum and removed from the glove box. A 2 wt % surfactant/H_2O solution (1 mL) was added through the septum via syringe and the mixture was stirred at the specified reaction temperature overnight. Upon completion, as monitored by TLC, the reaction was extracted with EtOAc (4 x 1 mL). The combined extracts were dried over anhydrous $MgSO_4$, filtered, and concentrated *in vacuo*. Products were purified by flash chromatography (see SI Section 9 for column conditions and yields of individual compounds).

Procedure for the 2-Step, 1-Pot amination/cyclization to afford acridine **11**:

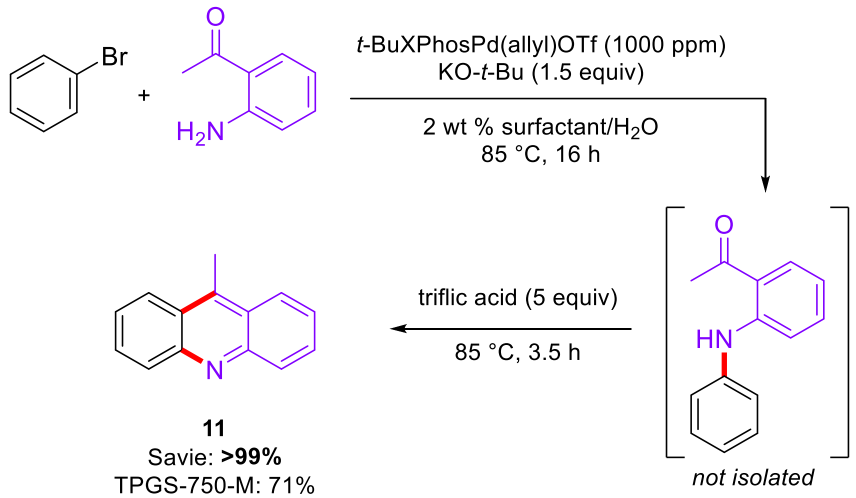

Supplementary Scheme S9: 2-Step, 1-pot acridine synthesis in surfactant media

To a 1 dr vial was added *t*-BuXPhosPd(allyl)OTf (4.5 mg). THF was added to make a stock solution at a concentration of 12.5 µmol/mL (i.e., 1.25 µmol /100 µL). A microliter syringe was then used to add the stock pre-catalyst solution to a 1 dr vial (e.g., for a 0.25 mmol scale reaction, 20 µL of the aforementioned stock solution would contain 0.25 µmol catalyst, thus corresponding

to 1000 ppm of the ligated Pd pre-catalyst). THF was then removed under reduced pressure and the atmosphere was replaced by argon using a Schlenk line.

To the 1 dr vial containing 1000 ppm pre-catalyst, a PTFE-coated magnetic stir bar and KO*t*Bu (84.2 mg, 0.75 mmol, 1.5 equiv) were added. A rubber septum was used to cap the reaction vial. The atmosphere of the reaction vial was switched to argon using a Schlenk line. A solution of 2 wt % surfactant/H_2O (1 mL) was added to the reaction mixture, which was then briefly stirred for 1 min at 85 °C. Then, bromobenzene (52.3 µL, 0.5 mmol, 1.0 equiv) and 2-aminoacetophenone (60.8 µL, 0.5 mmol, 1.0 equiv) were added via syringe under Ar. The vial was closed and parafilm was used to seal the vial. The reaction mixture was then stirred vigorously (~600–800 rpm) at 85 °C in an isotherm aluminum reaction block on an IKA hot plate for 16 h.

Upon completion of the first step (by TLC), the reaction mixture was neutralized with 1 M aqueous HCl. Then, triflic acid (0.22 mL, 2.5 mmol, 5 equiv) was added via syringe. The vial was closed and parafilm was used to seal the vial. The reaction mixture was then stirred vigorously (~600–800 rpm) at 85 °C in an isotherm aluminum reaction block on an IKA hot plate for 3.5 h.

After 3.5 h, the pH of the reaction mixture was adjusted to 13 by adding 5 M aqueous NaOH and allowed to stir at 85 °C for 2 min. The vial was then cooled to rt and placed in the fridge for 5 min. The vial was centrifuged, and the supernatant was then decanted off. The resulting solid was washed with water, centrifuged, and decanted (3 x 2 mL). The water was removed by washing with toluene and the resulting solid was further dried on high vac. The resulting yellow solid was purified via flash chromatography (20% EtOAc/hexanes) and the product was collected as a pale yellow solid (Savie: 97.0 mg, >99%; TPGS-750-M: 68.5 mg, 71%).

Synthesis of relevant starting materials:

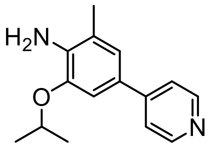

2-isopropoxy-6-methyl-4-(pyridin-4-yl)aniline was prepared according to a literature protocol.[5]

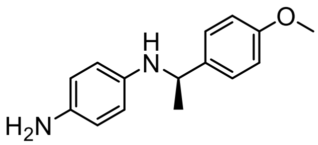

(*R*)-*N*1-(1-(4-methoxyphenyl)ethyl)benzene-1,4-diamine was prepared according to a literature protocol.[5]

7.3 Amide Bond Formation Reactions

General procedure for amide bond formation reactions:

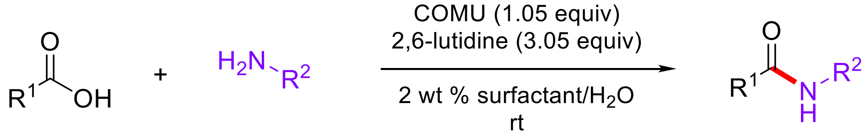

Supplementary Scheme S10: General procedure for amide bond formations in surfactant media

To a 1 dr vial equipped with a PTFE-coated magnetic stir bar was added carboxylic acid (1 equiv, 0.5 mmol), amine hydrochloride salt (1 equiv, 0.5 mmol), a 2 wt % aqueous solution of surfactant (1 mL), 2,6-lutidine (3.05 equiv, 1.525 mmol), and (1-cyano-2-ethoxy-2-oxoethyliden-aminooxy)dimethylamino-morpholinocarbenium hexafluorophosphate (COMU; 1.05 equiv, 0.525 mmol). The vial was sealed and allowed to stir at rt and the reaction was monitored by TLC analysis. Upon completion of the reaction, the reaction mixture was extracted with EtOAc (3 x 1 mL) and the organic layers were combined, then washed with 1 M aqueous HCl (3 x 3 mL), then washed with a 1:1 mixture of sat. aqueous $NaHCO_3$ and water (until the aqueous layer stopped turning yellow), followed by washing with brine, drying over anhydrous $MgSO_4$, and concentrating *in vacuo*. Samples were then purified by flash chromatography (80% EtOAc/hexanes; see SI Section 9 for yields of individual compounds).

7.4 CIP-Mediated Nitro Group Reductions

General procedure for CIP-mediated nitro group reductions:

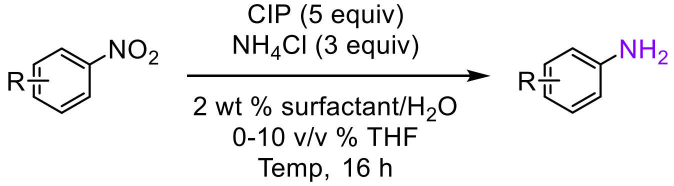

Supplementary Scheme S11: General procedure for nitro group reductions in surfactant media

For reactions containing co-solvent: To a 1 dr vial equipped with a PTFE-coated magnetic stir bar was added the nitro compound (1 equiv, 0.2 mmol), carbonyl iron powder (CIP; 5 equiv, 1 mmol), ammonium chloride (3 equiv, 0.6 mmol), and THF (10 v/v %, 40 µL), then the vial was capped and set to stir in an aluminum heating block on a heating stir plate set to 48 °C (this gave a temperature of 45 °C in the reaction vial) for 2 min. The vial was then removed from heating and a 2 wt % aqueous solution of surfactant (0.36 mL) was added and the vial was capped and returned

to the hot plate for 16 h. The reaction was monitored by TLC analysis. Upon completion, the vial was cooled to rt and extracted with EtOAc (3 x 1 mL) and the organic layers were combined and washed with brine, then dried over anhydrous $MgSO_4$ and concentrated *in vacuo*. Products were purified by flash chromatography (see SI Section 9 for column conditions and yields of individual compounds).

For co-solventless reactions: To a 1 dr vial equipped with a PTFE-coated magnetic stir bar was added the nitro compound (1 equiv, 0.2 mmol), carbonyl iron powder (CIP; 5 equiv, 1 mmol), ammonium chloride (3 equiv, 0.6 mmol), and a 2 wt % aqueous solution of surfactant (0.4 mL). The vial was capped and set to stir in an aluminum heating block on a heating stir plate set to 58 °C (this gave a temperature of 55 °C in the reaction vial) for 16 h. The reaction was monitored by TLC analysis. Upon completion, the vial was cooled to rt and extracted with EtOAc (3 x 1 mL) and the organic layers were combined and washed with brine, then dried over anhydrous $MgSO_4$ and concentrated *in vacuo*. Products were purified by flash chromatography (see SI Section 9 for column conditions and yields of individual compounds).

Synthesis of relevant starting materials:

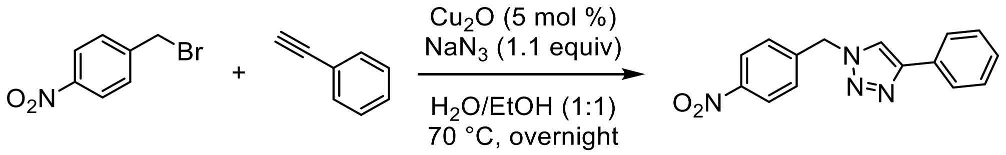

Supplementary Scheme S12: Synthesis of 1-(4-nitrobenzyl)-4-phenyl-1*H*-1,2,3-triazole

1-(4-nitrobenzyl)-4-phenyl-1*H*-1,2,3-triazole: To a 100 mL round-bottom flask equipped with a PTFE-coated magnetic stir bar was added Cu_2O (5 mol %, 1 mmol, 150 mg), 4-nitrobenzyl bromide (1.1 equiv, 22 mmol, 4.7527 g), NaN_3 (1.1 equiv, 22 mmol, 1.4302 g), EtOH (20 mL), H_2O (20 mL), and phenylacetylene (1 equiv, 20 mmol, 2.2 mL), then the mixture was heated to 70 °C in an oil bath overnight with stirring. Upon completion, the product precipitated and was collected via filtration, washed with water, then dried on vacuum. The crude material was then dissolved in EtOAc and filtered through a short (ca. 4 cm) silica plug, eluting with 40% EtOAc/hexanes. The solvent was removed *in vacuo* and product was recrystallized from EtOAc to afford the product as a white crystalline solid.

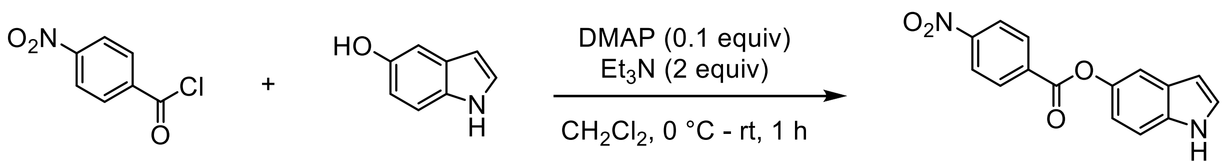

Supplementary Scheme S13: Synthesis of 1*H*-indol-5-yl 4-nitrobenzoate

1*H*-indol-5-yl 4-nitrobenzoate: To a 100 mL round bottom flask equipped with a PTFE-coated magnetic stir bar was added 5-hydroxyindole (1 equiv, 17.5 mmol, 2.33 g), DMAP (0.1 equiv, 1.75 mmol, 213 mg), CH_2Cl_2 (35 mL), and Et_3N (2 equiv, 35 mmol, 4.86 mL), then the flask was sealed with a rubber septum and chilled in an ice bath. Once chilled, *p*-nitrobenzoyl chloride (1.5 equiv, 26.3 mmol, 4.88 g) was added slowly with stirring, and a yellow color formed immediately. The reaction was allowed to warm to rt and stir for an additional hour and reaction progress was monitored via TLC. Upon complete consumption of 5-hydroxyindole, product was collected via filtration, washed with 1 M aq. HCl (50 mL), then washed with DI water (3 x 25 mL), and finally washed with hexanes (3 x 10 mL), then allowed to dry under vacuum to afford the product as a yellow solid.

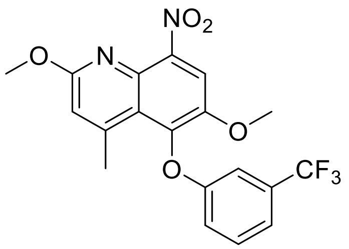

2,6-dimethoxy-4-methyl-8-nitro-5-(3-(trifluoromethyl)phenoxy)quinoline was prepared according to a literature protocol.[6]

7.5 S$_N$Ar Reactions

General procedure for S$_N$Ar reactions:

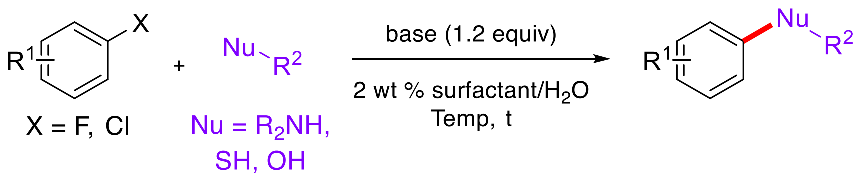

Supplementary Scheme S14: General procedure for S$_N$Ar reactions in surfactant media

To a 1 dr vial equipped with a PTFE-coated magnetic stir bar was added the aryl halide (1 equiv, 0.2 mmol), nucleophile (1 equiv, 0.2 mmol), base (if K_3PO_4 - 1.2 equiv, 0.24 mmol), a 2 wt % aqueous solution of surfactant (0.4 mL), and base (if Et_3N - 1.2 equiv, 0.24 mmol). The vial was capped and set to stir in an aluminum heating block on a heating stir plate set to the specified temperature. Reactions were monitored by TLC analysis. Upon completion, reactions were extracted with EtOAc (3 x 1 mL) and the organic layers were combined and washed with brine, then dried over anhydrous $MgSO_4$ and concentrated *in vacuo*. Products were purified by flash chromatography (see SI Section 9 for column conditions and yields of individual compounds).

7.6 Ppm Au-Catalyzed Alkyne Hydration

Procedure for Au-catalyzed alkyne hydration of pleconaril precursor **21**:

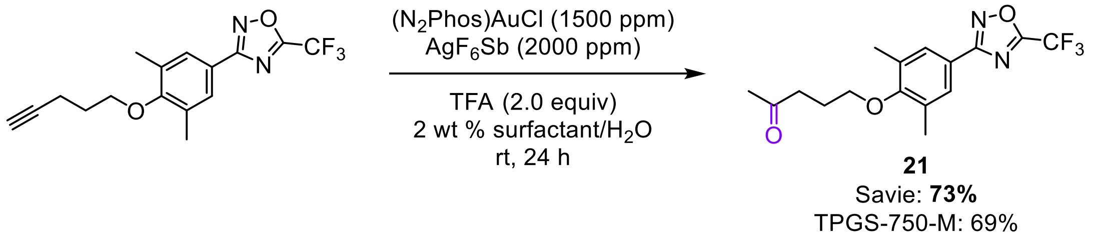

Supplementary Scheme S15: Au-catalyzed alkyne hydration to afford **21**

Preparation of gold pre-catalyst:

(Tetrahydrothiophene)AuCl: To a 5 mL round-bottom flask equipped with a PTFE-coated magnetic stir bar was added $HAuCl_4$ (1 mmol, 394 mg), EtOH (3.3 mL), and DI water (0.7 mL) followed by dropwise addition of tetrahydrothiophene (2.1 mmol, 0.19 mL). The reaction mixture was stirred for 30 min at rt until the yellow precipitate was transformed into a white solid. The resulting white solid was filtered, washed with EtOH, and dried under vacuum.

(N_2Phos)Au(I) Chloride: A 10 mL round-bottom flask equipped with a PTFE-coated magnetic stir bar and septum was charged with N_2Phos (0.05 mmol, 42.6 mg) and (tetrahydrothiophene)-gold(I) chloride (0.05 mmol, 16.0 mg). A rubber septum was added to the flask, which was evacuated and backfilled with argon three times. The flask was covered with aluminum foil to protect it from light. Anhydrous CH_2Cl_2 (2 mL) was added via syringe and the reaction was stirred at rt for 2 h. After, the solvent was removed *in vacuo*, then the product, a white solid, was placed under high vacuum overnight to remove trace amounts of solvent and tetrahydrothiophene.

Gold Pre-catalyst: To a 2 dr vial covered with aluminum foil and equipped with a PTFE-coated magnetic stir bar was added (N_2Phos)Au(I) chloride (0.003 mmol, 3.3 mg) and silver(I) hexafluoroantimonate (0.006 mmol, 2.1 mg) in an argon-filled glove box. The vial was capped with a rubber septum and removed from the glove box, then anhydrous CH_2Cl_2 (3 mL) was added via syringe and the solution was stirred for 15-20 minutes prior to use.

Alkyne hydration:

To a 2 dr vial equipped with a PTFE-coated magnetic stir bar and capped with a rubber septum and purged with argon was added gold pre-catalyst solution (0.5 mL), then the solvent was carefully removed *in vacuo*. Then, the alkyne (1 equiv, 0.5 mmol, 162 mg) was added, and the vial was resealed with the septum and evacuated and backfilled with argon three times. A 2 wt % solution of surfactant/H_2O (1 mL), followed by trifluoroacetic acid (TFA; 2 equiv, 1 mmol, 77 µL) were added through the septum via syringe and the mixture was stirred under argon at rt for 24 h. Upon completion, as monitored by TLC, the reaction mixture was extracted with EtOAc (4 x 1 mL). The combined extracts were dried over anhydrous $MgSO_4$, filtered, and concentrated *in vacuo*. The product was purified by flash chromatography (gradient 0-30% EtOAc/hexanes) to afford **21** as a white solid (Savie: 125.1 mg, 73%; TPGS-750-M: 118.1 mg, 69%).

Synthesis of relevant starting materials:

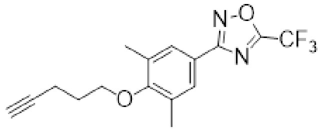

5-(2,6-dimethyl-4-(5-(trifluoromethyl)-1,2,4-oxadiazol-3-yl)phenoxy)pentan-2-one-3-(3,5-dimethyl-4-(pent-4-yn-1-yloxy)phenyl)-5-(trifluoromethyl)-1,2,4-oxadiazole was prepared according to a literature procedure.[7]

7.7 Ru-Catalyzed Olefin Metathesis

Procedure for Ru-catalyzed olefin metathesis of *O*-benzyleugenol to afford **22**:

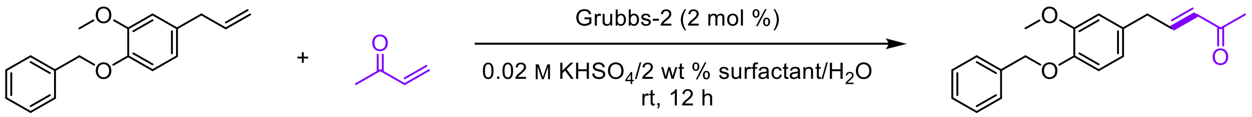

22
Savie: **87%**
TPGS-750-M: 87%

Supplementary Scheme S16: Ru-catalyzed olefin metathesis to afford **22**

To a 1 dr vial equipped with a PTFE-coated magnetic stir bar was added $KHSO_4$ (1.4 mg), then the vial was brought into an argon-filled glove box where Grubbs-2 catalyst (2 mol %, 0.01 mmol, 8.3 mg) was added. The vial was sealed with a rubber septum and removed from the glove box, then a 2 wt % aqueous solution of surfactant (1 mL) was added via syringe under a positive flow of argon, followed by *O*-benzyleugenol (1 equiv, 0.5 mmol, 120 µL) and methyl vinyl ketone (3 equiv, 1.5 mmol, 130 µL). The vial was set to stir at rt and the reaction was monitored by TLC analysis. Upon completion, the reaction mixture was loaded onto a 5 cm plug of diatomaceous earth and eluted with EtOAc, then the eluent was concentrated *in vacuo*. The product was purified by flash chromatography (gradient 0-25% EtOAc/hexanes) to afford **22** as a white solid (Savie: 122.4 mg, 87%; TPGS-750-M: 122.3 mg, 87%).

Synthesis of relevant starting materials:

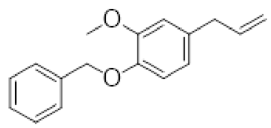

***O*-benzyleugenol** was prepared according to a literature protocol.[8]

7.8 Ni-Catalyzed *Gem*-Dibromocyclopropane Reduction

Procedure for Ni-catalyzed *gem*-dibromocyclopropane reduction to afford **23**:

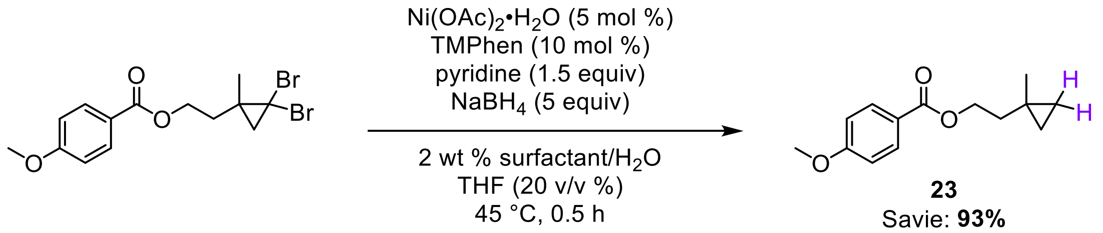

Supplementary Scheme S17: Ni-catalyzed cyclopropane dehalogenation to afford **23**

To a 5 mL round-bottom flask equipped with a PTFE-coated magnetic stir bar was added $Ni(OAc)_2 \cdot 4H_2O$ (5 mol %, 0.01 mmol, 2.5 mg), and 3,4,7,8-tetramethyl-1,10-phenanthroline (TMPhen; 10 mol %, 4.7 mg). The flask was sealed with a rubber septum and purged with argon with the use of a purge needle. A 2 wt % aqueous solution of surfactant (0.32 mL) was added and allowed to stir at rt for 10 min. Pyridine (1.5 equiv, 0.3 mmol, 24 µL) was added via syringe and allowed to stir for 5 min. $NaBH_4$ (5 equiv, 1 mmol, 38 mg) was added in a single portion and the flask was again capped with a rubber septum and sealed to prevent leaking. The *gem*-

dibromocyclopropane substrate (1 equiv, 0.2 mmol, 78.4 mg) was dissolved in THF (20 v/v %, 80 µL) and added as a solution to the flask through the septum. A 6 mL syringe (containing 0.1 mL of THF) was added through the septum to accept evolving hydrogen gas. The reaction was stirred for 30 min at 45 °C in an oil bath. If the volume of gas generated exceeds the volume of the syringe, the syringe must be emptied outside of the flask and the ballast adapted again. After completion, the reaction was extracted with EtOAc (3 mL) and filtered through a pad of silica. The pad was rinsed with EtOAc (3 x 3 mL). The organic layer was dried over anhydrous $MgSO_4$, filtered, and concentrated *in vacuo*. Products were purified by flash chromatography (5 % EtOAc/hexanes) to afford **23** as a tan oil (Savie: 43.6 mg, 93%; TPGS-750-M: 42.6 mg, 91%).

Synthesis of relevant starting materials:

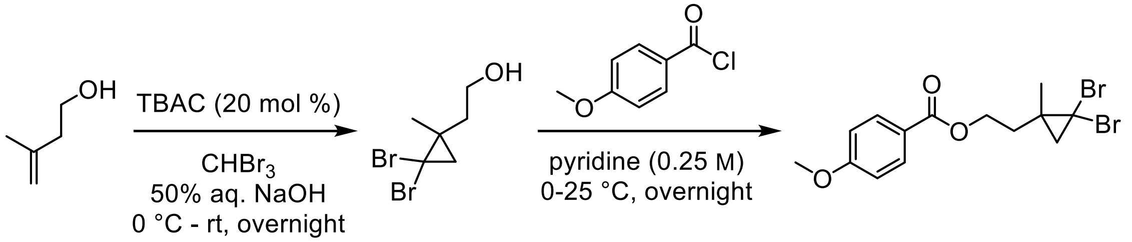

Supplementary Scheme S18: Synthesis of 2-(2,2-dibromo-1-methylcyclopropyl)ethyl 4-methoxybenzoate

2-(2,2-dibromo-1-methylcyclopropyl)ethyl 4-methoxybenzoate:

Cyclopropanation: To a 25 mL round-bottom flask equipped with a PTFE-coated magnetic stir bar was added 3-methylbut-3-en-1-ol (1 equiv, 10 mmol, 1.0 mL), tetrabutylammonium chloride (TBAC; 20 mol %, 455.6 mg), and bromoform (3.5 mL), then the mixture was allowed to cool in an ice bath before a 50 w/w % aqueous solution of NaOH (3.5 mL) was added. The flask was then sealed with a rubber septum and wrapped tightly with electrical tape to prevent the septum from popping off as dibromocarbene gas is generated. The mixture was allowed to warm to rt and stir overnight. Upon completion, the mixture was diluted with 50 mL of ice water, neutralized with 1 M aqueous HCl, and extracted with EtOAc (3 x 20 mL). The combined organic extracts were washed with brine, then dried over anhydrous $MgSO_4$ and solvent was removed *in vacuo*. Product 2-(2,2-dibromo-1-methylcyclopropyl)ethan-1-ol was purified by flash chromatography (20% EtOAc/hexanes) and isolated as a brown oil (748 mg, 29%)

NOTE: Since this reaction involves evolution of gas in a sealed container, glassware should be checked for fractures prior to use. It is advisable to set up a blast shield as an added precaution.

Esterification: To a 50 mL round-bottom flask equipped with a PTFE-coated magnetic stir bar was added 2-(2,2-dibromo-1-methylcyclopropyl)ethan-1-ol (1 equiv, 3.99 mmol, 1.0284 g) and

pyridine (16 mL), then the mixture was allowed to cool in an ice bath. To the mixture was added 4-methoxybenzoyl chloride (1.1 equiv, 4.39 mmol, 748.1 mg), then the reaction was capped with a rubber septum, through which a vent needle was pierced. The mixture was allowed to warm to rt and stir overnight. The mixture was then poured into 100 mL water, extracted with Et_2O (3 x 25 mL), then the combined organic extracts were washed with water (3 x 20 mL), then brine (20 mL), then dried over anhydrous $MgSO_4$. Product 2-(2,2-dibromo-1-methylcyclopropyl)ethyl 4-methoxybenzoate was isolated by flash chromatography (20% EtOAc/hexanes) and afforded a tan oil that solidified to a wax on prolonged standing (597.5 mg, 62%)

7.9 CuH-Catalyzed Asymmetric Ketone Reduction

Procedure for CuH-catalyzed asymmetric ketone reduction to afford **24**:

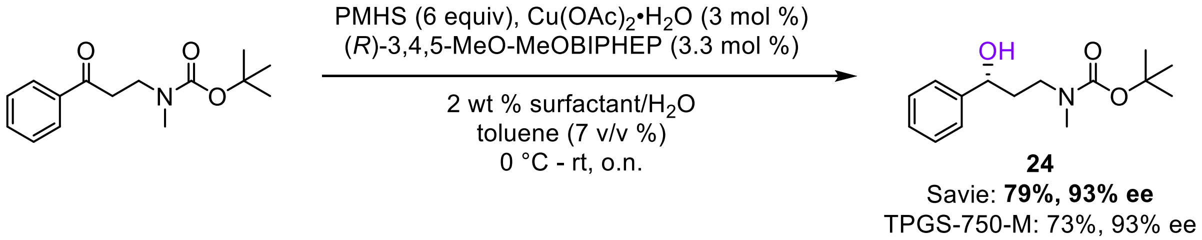

Supplementary Scheme S19: CuH-catalyzed asymmetric ketone reduction to afford **24**

To a 2 dr vial equipped with a PTFE-coated magnetic stir bar was added the ketone (1 equiv, 0.4 mmol, 105.3 mg) and $Cu(OAc)_2 \cdot H_2O$ (3 mol %, 2.4 mg). The vial was purged with argon and brought into an argon-filled glovebox where (*R*)-3,4,5-MeO-MeOBIPHEP (3.3 mol %, 12.4 mg) was added. The vial was capped with a rubber septum and removed from the glovebox, then adapted to an argon line. Through the septum, via syringe, degassed toluene (140 μL) was added, followed by the aqueous 2 wt % surfactant solution with vigorous stirring. The mixture was allowed to stir for 1 h to ensure the copper catalyst was fully ligated, as indicated by the emulsion transforming from a faint turquoise color to faint yellow. At this time, the vial was allowed to stir in an ice bath to cool, then polymethylhydrosiloxane (PMHS; 6 equiv, 2.4 mmol, 144 μL) was added via syringe in five ca. 30 μL portions every 0.5 h. During the addition, ice was replaced to keep the solution at ca. 0 °C. Upon the final addition, the septum was replaced with a screw-on cap, then the reaction was allowed to warm to rt and stir overnight. On completion, the vial was opened to release gas, then the aqueous phase was extracted with EtOAc (5 x 2 mL), and the extracts were dried over anhydrous $NaSO_4$, solvent was removed *in vacuo*, and product was isolated by flash chromatography (25% EtOAc/hexanes) to afford **24** as a white solid (Savie: 83.5 mg, 79%; TPGS-750-M: 77.5 mg, 73%). The ee of the products were determined by chiral HPLC (see SI Section 9).

Synthesis of relevant starting materials:

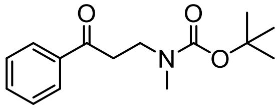

***tert*-butyl methyl(3-oxo-3-phenylpropyl)carbamate** was prepared according to a literature procedure.[9]

7.10 Fe/ppm Pd Nanoparticle-Catalyzed Suzuki-Miyaura Couplings

Grignard titration:

To a 25 ml flamed-dried round-bottom flask equipped with a PTFE-coated magnetic stir bar was added menthol (1 mmol, 156 mg), 1,10-phenanthroline (ca. 1 mg), and anhydrous THF (5 ml), then the flask was placed under an argon atmosphere. The Grignard reagent was then added dropwise (1 drop every 2-5 seconds), watching for bubbling to cease before adding the next drop. Addition of the Grignard was continued until a pink color formed and was maintained for over 5 minutes.

NOTE: The end-point is very faint, so titrating with a white background is recommended. Over-titrating will result in a vibrant pink color.

Preparation of Fe/ppm Pd nanoparticles:

To a flame-dried, two-neck, 50 mL round-bottom flask equipped with a PTFE-coated magnetic stir bar inside an argon-filled glove box was added anhydrous $FeCl_3$ (3.09 mmol, 500 mg), SPhos (2.47 mmol, 1015 mg), and $Pd(OAc)_2$ (0.027 mmol, 6.1 mg). The flask was closed with a septum and dry THF (8 mL) was added. The reaction mixture was stirred for 20 min at rt. While maintaining a dry atmosphere at rt, a 0.5 M solution of MeMgCl in THF (12.4 mL) was added very slowly (1 drop every two seconds) to the reaction mixture. After complete addition of the Grignard reagent, the reaction mixture was stirred for an additional 10 min at rt. The appearance of a black coloration was indicative of generation of the desired nanomaterial. After 20 min, the mixture was quenched with a single drop of degassed water, and THF was evaporated under reduced pressure at rt followed by triturating the mixture with dry pentane to provide a black nano-powder. The nanomaterial was dried under reduced pressure for 1 hr and could be used as such for coupling reactions under micellar conditions.

General procedure for Fe/ppm Pd-catalyzed SMC reactions:

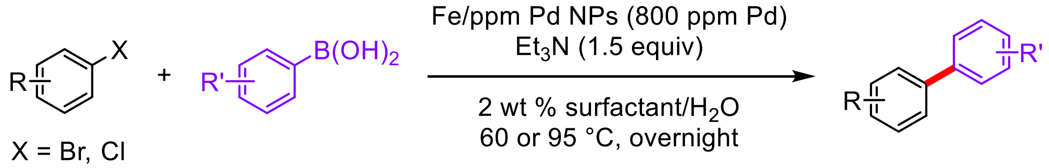

Supplementary Scheme S20: General procedure for Fe/ppm Pd NP-catalyzed SMC reactions in surfactant media

To a 1 dr vial equipped with a PTFE-coated magnetic stir bar was added Fe/ppm Pd NPs (20 mg, 800 ppm Pd), aryl halide (1 equiv, 0.5 mmol) and organoboron (1.5 equiv, 0.75 mmol). The vial was then evacuated and backfilled with argon three times. Under positive flow of argon, an aqueous 2 wt % surfactant solution (1 mL) and Et_3N (1.5 equiv, 0.75 mmol, 104 µL) were added. The vial was placed in an aluminum heating block over a stir plate with the stir rate set to >1000 rpm with a thermocouple probe in the aluminum block set to 65 °C (gives a temperature of 60 °C inside the reaction vial) or 98 °C (for 95 °C reaction). The reactions were monitored by TLC analysis. Upon completion of the reaction, the vial was cooled to rt. The mixture was extracted with MTBE (2 mL) and the organic layer was washed with brine three times. The layers were then separated, and the organic layer was dried over anhydrous Na_2SO_4. The mixture was filtered and concentrated *in vacuo*, then the crude material was purified via flash chromatography (see SI Section 9 for column conditions and yields of individual compounds).

Synthesis of relevant starting materials:

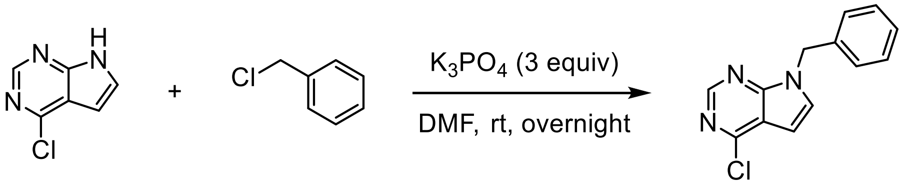

Supplementary Scheme S21: Synthesis of 7-Benzyl-4-chloro-7*H*-pyrrolo[2,3-*d*]pyrimidine

7-benzyl-4-chloro-7*H*-pyrrolo[2,3-*d*]pyrimidine: To a 50 mL round-bottom flask equipped with a PTFE-coated magnetic stir bar added 4-chloro-7*H*-pyrrolo[2,3-*d*]pyrimidine (1 equiv, 8.253 mmol, 1.2682 g) and DMF (15 mL), then stirred to dissolve. To the mixture, added K_3PO_4 (3 equiv, 24.77 mmol, 3.424 g) and stirred for 15 min at rt, then added benzyl chloride (1.5 equiv, 12.39 mmol, 1.425 mL) and stirred the mixture at rt overnight. Upon completion, the mixture was poured into 100 mL H_2O in a separatory funnel, then extracted with CH_2Cl_2 (3 x 20 mL). The combined extracts were washed with H_2O (2 x 25 mL), then brine (20 mL), then dried over anhydrous $MgSO_4$. The solvent was removed *in vacuo* and product was isolated by flash chromatography (10% EtOAc/hexanes) to obtain the title compound as a white solid (1.8082 g, 90%)

7.11 Fe/ppm Cu Nanoparticle-Catalyzed CuAAC Reaction

Preparation of Fe/ppm Cu nanoparticles:

To a flame-dried, two-neck, 50 mL round-bottom flask equipped with a PTFE-coated magnetic stir bar inside an argon-filled glove box was added anhydrous $FeCl_3$ (3.09 mmol, 500 mg). The flask was closed with a septum and dry THF (8 mL) was added. While maintaining a dry atmosphere at rt, a 0.5 M solution of MeMgCl in THF (titrated prior to use as outlined in SI Section 7.10; 12.4 mL) was added very slowly (1 drop every two seconds) to the reaction mixture. After complete addition of the Grignard reagent, the reaction mixture was stirred for an additional 10 min at rt. The appearance of a black coloration was indicative of generation of the desired nanomaterial. After 20 min, the mixture was quenched with a single drop of degassed water, and THF was evaporated under reduced pressure at rt followed by triturating the mixture with dry pentane to provide a black iron nano-powder. The nanomaterial was then dried under reduced pressure for 1 hr.

To a separate oven-dried 2 dr vial equipped with a PTFE-coated magnetic stir bar in an argon-filled glovebox was added $Cu(CH_3CN)_4(PF_6)$ (2.8 mg), then the vial was capped with a rubber septum and removed from the glovebox. Through the septum, via syringe, was added anhydrous, degassed THF (2.5 mL) and the mixture was allowed to stir until all catalyst dissolved.

To a separate oven-dried 1 dr vial (to be used for the subsequent CuAAC reaction) was added 20 mg of the iron nano-powder, then the vial was capped with a rubber septum and evacuated and backfilled with argon three times using a Schlenk line. To the mixture was added 0.5 mL of the catalyst stock solution, then the mixture was allowed to stir at rt for 15 min, at which time THF was removed carefully *in vacuo*.

NOTE: This procedure differs from that outlined in SI Section 7.10 insofar as the iron NPs are prepared in the absence of the catalyst, then they are subsequently doped with the catalyst.

Procedure for Fe/ppm Cu-catalyzed CuAAC reaction to afford **27**:

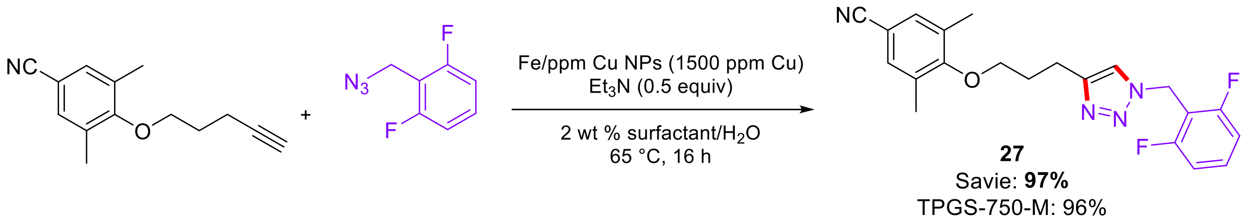

Supplementary Scheme S22: Fe/ppm Cu CuAAC reaction in surfactant media

To the 1 dr vial containing the Cu-doped NPs was added the alkyne (1 equiv, 0.5 mmol, 106.6 mg) under a constant flow of argon, then the septum was replaced and the vial was evacuated and

backfilled with argon three times using a Schlenk line. Through the septum, via syringe, was added the azide (1.2 equiv, 0.6 mmol, 101.5 mg), a 2 wt % aqueous solution of the respective surfactant (1 mL), and Et_3N (0.5 equiv, 0.25 mmol, 35 µL), then the mixture was allowed to stir at 65 °C overnight. The reaction mixture was poured onto a ca. 4 cm plug of Celite and eluted with EtOAc, then the solvent was removed *in vacuo* and product **27** was purified by flash chromatography (40% EtOAc/hexanes) and isolated as a white solid (Savie: 185.9 mg, 97%; TPGS-750-M: 182.6 mg, 96%).

Synthesis of relevant starting materials:

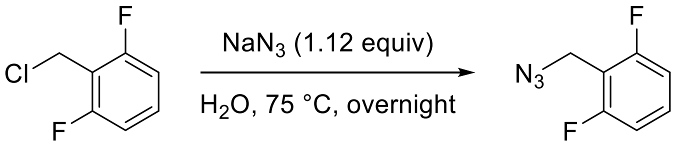

Supplementary Scheme S23: Synthesis of 2-(azidomethyl)-1,3-difluorobenzene

2-(azidomethyl)-1,3-difluorobenzene: To a 25 mL round-bottom flask was added 2,6-difluoro-benzylchloride (1 equiv, 10 mmol, 1.6256 g), H_2O (10 mL), and NaN_3 (1.12 equiv, 11.2 mmol, 728 mg), then the flask was sealed with a rubber septum and allowed to stir overnight in a 75 °C oil bath. Upon completion, the product azide collected as a colorless oil at the bottom of the flask. The oil was removed via pipette and dried over $MgSO_4$ (1.1444 g, 68%).

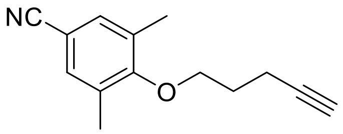

3,5-dimethyl-4-(pent-4-yn-1-yloxy)benzonitrile was prepared according to a literature procedure.[7]

7.12 Biocatalytic Reactions

NOTE: No products were isolated for the purposes of this study as the intention was to compare conversions only. ^{1}H NMR spectra of crude materials were used both to determine conversion, and to confirm the identity of the product compared to literature references (ERED[10], ADH[11], and lipase[12]).

Preparation of surfactant/buffer solution:

Aqueous 1 M stock solutions of potassium phosphate monobasic (**A**) and potassium phosphate dibasic (**B**) were prepared. A pH 7 phosphate buffer solution was then prepared by mixing 38.5 mL of solution **A** with 61.5 mL of solution **B**. The pH was controlled and adjusted, if needed, with a 1 M aqueous solution of NaOH or HCl. The buffer solution was diluted with HPLC grade water to 0.1 M for ERED and ADH, and 0.01 M for lipase. 2 wt % of solid surfactant was dissolved in the respective buffer solution, and used as the medium for the reactions.

Procedure for ERED-catalyzed asymmetric reduction of α,β-unsaturated ketone to afford **28**:

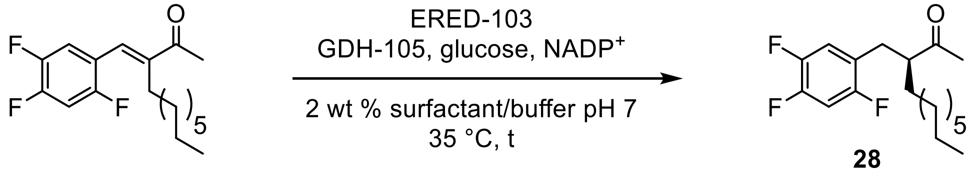

Supplementary Scheme S24: ERED-catalyzed asymmetric α,β-unsaturated ketone reduction to afford **28**

To a 1 dr vial equipped with a PTFE-coated magnetic stir bar was added the α,β-unsaturated ketone (1 equiv, 0.016 mmol, 5 mg), glucose (2 equiv, 0.032 mmol, 5.8 mg), $NADP^+$ (0.5 mg), GDH-105 (2 mg), and ERED-103 (10 mg) followed by 2 wt % surfactant/buffer (1 mL), then the vial was capped and allowed to stir at 35 °C for the specified time. Each time point was a separate reaction. After the specified time had elapsed, the reaction was extracted with MTBE (5 x 0.5 mL), the organic layers were combined and dried over anhydrous $MgSO_4$, filtered, and concentrated *in vacuo*. Conversion was determined by ^1H NMR analysis of the crude material (singlet at 7.34 ppm (1H) → multiplet at 2.68 ppm (1H)).

Procedure for ADH-catalyzed asymmetric reduction of ketone to afford **29**:

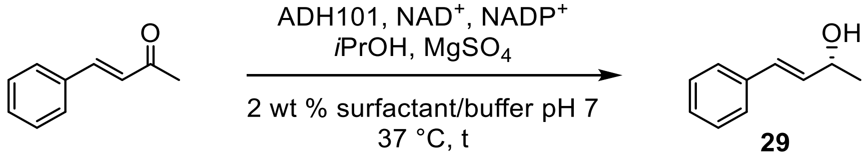

Supplementary Scheme S25: ADH-catalyzed asymmetric ketone reduction to afford **29**

To a 2 dr vial equipped with a PTFE-coated magnetic stir bar was added the ketone (1 equiv, 0.2 mmol, 29.2 mg), $MgSO_4$ (0.8 mg), NAD^+ (2.6 mg), $NADP^+$ (2.4 mg), *i*PrOH (0.4 mL), 2 wt % surfactant/buffer (3.2 mL), and ADH101 (20 mg), then the vial was capped and allowed to stir at 37 °C for the specified time. Each time point was a separate reaction. After the specified time had

elapsed, the reaction was extracted with MTBE (5 x 0.5 mL), then the organic layers were combined, dried over anhydrous $MgSO_4$, filtered, and concentrated *in vacuo*. Conversion was determined by ^{1}H NMR analysis of the crude material (singlet at 2.39 ppm (3H) → doublet at 1.38 ppm (3H)).

Procedure for lipase-catalyzed esterification to afford **30**:

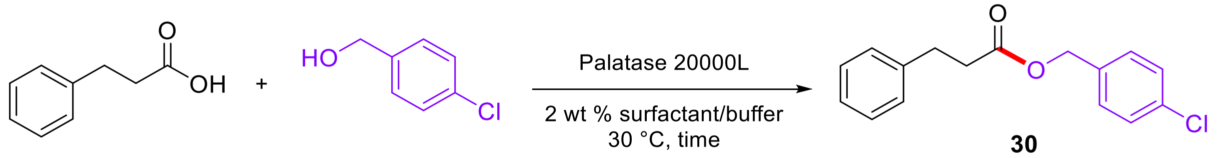

Supplementary Scheme S26: Lipase-catalyzed esterification to afford **30**

To a 1 dr vial equipped with a PTFE-coated magnetic stir bar was added 3-phenylpropanoic acid (1 equiv, 0.5 mmol, 75.1 mg), 4-chlorobenzyl alcohol (1 equiv, 0.5 mmol, 71.3 mg), and 2 wt % surfactant/buffer and the mixture was stirred vigorously to emulsify the reagents. Then, palatase 20000L was added (25 µL) and the vial was capped and allowed to stir at 30 °C for the specified time. Each time point was a separate reaction. After the specified time had elapsed, the reaction was extracted with MTBE (5 x 0.5 mL), the organic layers were combined and dried over anhydrous $MgSO_4$, filtered, and concentrated *in vacuo*. Conversion was determined using ^{1}H NMR analysis of the crude material (singlet at 4.66 (2H) → singlet at 5.07 (2H)).

7.13 1-Pot Pd-Catalyzed Cyanation, then ERED Reduction, then ADH Reduction

Procedure for 1-Pot Pd-catalyzed cynation, then ERED reduction, then ADH reduction:

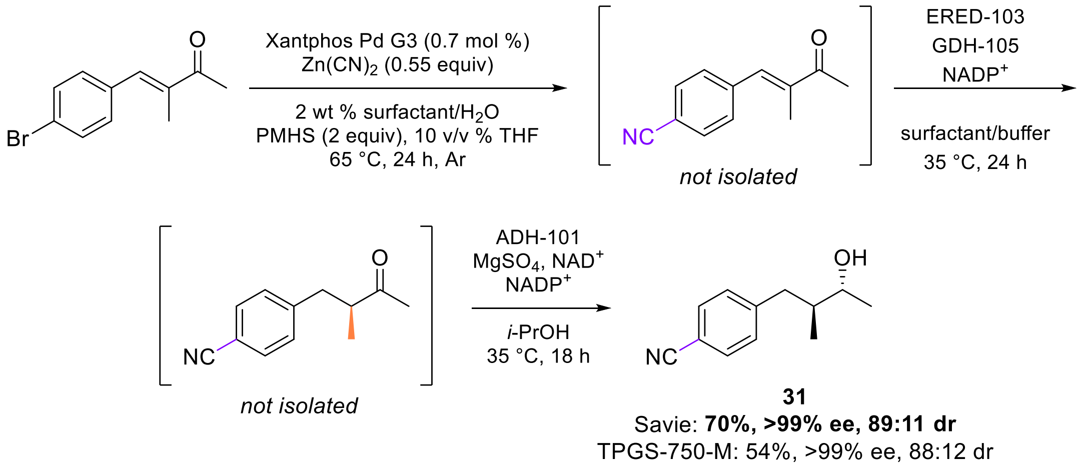

Supplementary Scheme S27: 3-Step, 1-pot chemoenzymatic sequence to afford **31**

Step 1 - Pd-catalyzed cyanation:

To a 1 dr vial equipped with a PTFE-coated magnetic stir bar was added (*E*)-4-(4-bromophenyl)-3-methylbut-3-en-2-one (1 equiv, 0.2 mmol, 48 mg), $Zn(CN)_2$ (0.55 equiv, 0.11 mmol, 13 mg), xantphos Pd G3 (0.7 mol %, 1.7 mg), and the vial was capped with a rubber septum and evacuated and backfilled with argon three times. Through the septum was added polymethylhydroxysilane (PMHS; 2 equiv, 0.4 mmol, 26 µL), THF (10 v/v %, 40 µL), and 2 wt % surfactant/H_2O (360 µL). The vial was allowed to stir at 65 °C for 24 h.

Step 2 - ERED reduction:

The buffer solution was prepared as in the ERED example described in SI Section 7.12. The reaction mixture from Step 1 (excepting the stir bar) was transferred to a 5 dr vial equipped with a larger football-shaped PTFE-coated magnetic stir bar. 2 wt % surfactant/buffer was used to wash the original 1 dr vial and then transferred to the 5 dr vial (a total of 5.6 mL 2 wt % surfactant/buffer solution was added). To the vial was added ERED-103 (70 mg), GDH-105 (20 mg), $NADP^+$ (5 mg), and glucose (2 equiv, 0.4 mmol, 72 mg). The vial was capped and stirred gently at 35 °C for 24 h.

Step 3 – ADH reduction:

To the 5 dr vial was added anhydrous $MgSO_4$ (0.8 mg), NAD^+ (2.6 mg), $NADP^+$ (2.4 mg), ADH101 (20 mg), and *i*PrOH (0.6 mL). The reaction vial was capped and allowed to react at 35 °C for 18 h. Upon completion, the reaction mixture was extracted with EtOAc (3 x 2 mL). If the extraction solvent became emulsified, the mixture was centrifuged to resolve the layers. The organic layers were combined and dried over anhydrous $MgSO_4$, filtered, and concentrated *in vacuo*. The crude mixture was purified by flash chromatography (gradient 0-25% EtOAc/hexanes) to afford **31** as a colorless oil (Savie: 26.4 mg, 70%; TPGS-750-M: 20.4 mg, 54%). The ee and dr of products were determined by chiral HPLC (see SI Section 9).

Synthesis of relevant starting materials:

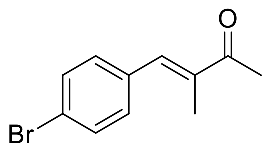

(*E*)-4-(4-bromophenyl)-3-methylbut-3-en-2-one was prepared according to a literature protocol.[13]

7.14 Gram-Scale Amide Bond Formation

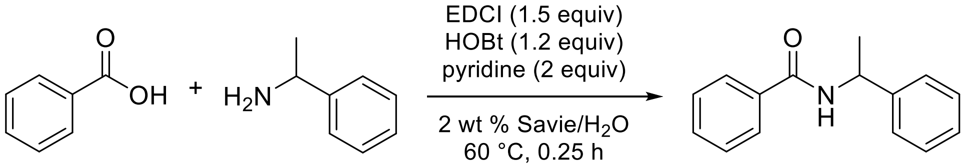

32
96% yield

Supplementary Scheme S28: Gram-scale amide bond formation in aqueous Savie

To a 25 mL pear-shaped round-bottom flask equipped with a PTFE-coated magnetic stir bar was added benzoic acid (1 equiv, 5 mmol, 610 mg), HOBt hydrate (1.2 equiv, 6 mmol, 919 mg), 2 wt % Savie/H_2O (10 mL), pyridine (2 equiv, 10 mmol, 0.8 mL), racemic 1-phenylethan-1-amine (1 equiv, 5 mmol, 645 μL), and EDCI (1.5 equiv, 7.5 mmol, 1.4378 g), then the flask was sealed with a rubber septum and the reaction was vigorously stirred in an oil bath at 60 °C for 15 min. Upon completion, the precipitated product was collected via filtration and washed with copious DI water to afford **32** as a fluffy white solid (1.0814 g, 96%).

7.15 Ni-Catalyzed Migita-Like C-S Bond Formation and Recycling

Preparation of $Ni(Phen)_2Br_2$ pre-catalyst:

To a 1 dr vial equipped with a PTFE-coated magnetic stir bar was added $NiBr_2$ (0.5 mmol, 109.3 mg), phenanthroline (Phen; 1.0 mmol, 180.2 mg) and acetonitrile (1 mL). The vial was placed in a 45 °C aluminum heating block, resulting in a rapid color change to pink, then to green. It was allowed to stir overnight. The vial was centrifuged, and the supernatant was removed. The pellet was washed by EtOAc (1 x 1 mL) and then pentane (2 x 1 mL). The green solid was dried under vacuum for 24 h.

Procedure for Migita-like cross-coupling to afford **33**, and recycling study:

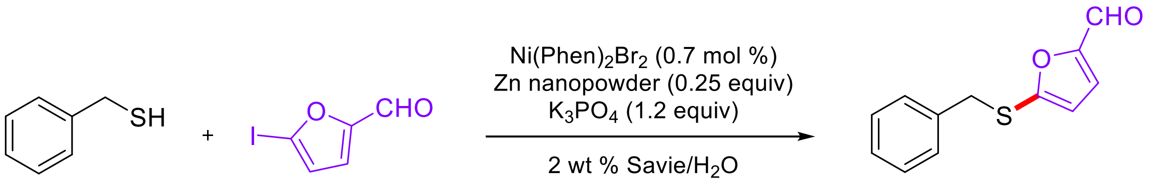

33

Supplementary Scheme S29: Ni-catalyzed Migita-like C-S bond formation in 2 wt % aqueous Savie

To a 1 dr vial equipped with a PTFE-coated magnetic stir bar was added Ni(Phen)$_2$Br$_2$ (0.7 mol %, 1.0 mg), Zn nanopowder (0.25 equiv, 0.063 mmol, 4.1 mg), K_3PO_4 (1.2 equiv, 0.3 mmol, 63.7 mg), and 5-iodofuran-2-carbaldehyde (1 equiv, 0.25 mmol, 55.5 mg). The vial was capped with a rubber septum, then evacuated and backfilled with argon three times. Next, benzyl mercaptan (1.05 equiv, 0.263 mmol, 77.6 mg) and 2 wt % Savie/H_2O (0.5 mL) was added via a syringe through the septum. The solution was heated at 45 °C and stirred vigorously for 20 h.

After the reaction, to the vial was added ca. 1 mL of EtOAc (3x) and the organic phase was removed via syringe, while keeping the mixture under Ar. The organic extracts were concentrated under reduced pressure to obtain a crude oil, which was then subjected to flash chromatography (10% EtOAc/hexanes) to afford **33** as a brown oil.

Recycle:

To a separate 1 dr vial equipped with a PTFE-coated magnetic stir bar was added Ni(Phen)$_2$Br$_2$ (0.7 mol %, 1.0 mg), Zn nanopowder (0.25 equiv, 0.063 mmol, 4.1 mg), K_3PO_4 (1.2 equiv, 0.3 mmol, 63.7 mg), and 5-iodofuran-2-carbaldehyde (1 equiv, 0.25 mmol, 55.5 mg). The vial was capped with a rubber septum and then evacuated and backfilled with argon three times. Next, benzyl mercaptan (1.05 equiv, 0.263 mmol, 77.6 mg) and the previously used 0.5 mL 2 wt % Savie/H_2O from the initial reaction was added via a syringe through the septum. The solution was heated at 45 °C and stirred vigorously for 20 h and the same workup and purification as used in the initial reaction was repeated for this and the other additional reaction cycles.

Initial Reaction: 49.6 mg (91%)
Recycle 1: 47.4 mg (87%)
Recycle 2: 41.3 mg (76%)
Recycle 3: 45.7 mg (84%)

8. References

1. Lipshutz, B. H.; Ghorai, S.; Abela, A. R.; Moser, R.; Nishikata, T.; Duplais, C.; Krasovskiy, A.; Gaston, R. D.; Gadwood, R. C. TPGS-750-M: A Second-Generation Amphiphile for Metal-Catalyzed Cross-Couplings in Water at Room Temperature *J. Org. Chem.* **2011**, *76*, 4379–4391.
2. Lee, N. R.; Cortes-Clerget, M.; Wood, A. B.; Lippincott, D. J.; Pang, H.; Moghadam, F. A.; Gallou, F.; Lipshutz, B. H. Coolade. A Low-Foaming Surfactant for Organic Synthesis in Water *ChemSusChem*, **2019**, *12*, 3159-3165.
3. Heller, P.; Mohr, N.; Birke, A.; Weber, B.; Reske-Kunz, A.; Bros, M.; Barz, M. Directed interactions of block copolypept(o)ides with mannose-binding receptors:

PeptoMicelles targeted to cells of the innate immune system *Macromol. Biosci.* **2015**, *15*, 63-73.
4. Byrne, F. P.; Jin, S.; Paggiola, G.; Petchey, T. H. M.; Clark, J. H.; Farmer, T. J.; Hunt, A. J.; McElroy, C. R.; Sherwood, J. Tools and Techniques for Solvent Selection: Green Solvent Selection Guides. *Sustain. Chem. Process* **2016**, *4*, 7.
5. Lee, N. R.; Bikovtseva, A. A.; Cortes-Clerget, M.; Gallou, F.; Lipshutz, B. H. Carbonyl Iron Powder: A Reagent for Nitro Group Reductions under Aqueous Micellar Catalysis Conditions *Org. Lett.* **2017**, *19*, 6518-6521.
6. Kavthe, R. D.; Kincaid, J. R. A.; Lipshutz, B. H. An Efficient & Sustainable Synthesis of the Antimalarial Drug Tafenoquine *ACS Sustain. Chem. Eng. in press*.
7. Egorova, A.; Kazakova, E.; Jahn, B.; Ekins, S.; Makarov, V.; Schmidtke, M. Novel Pleconaril Derivatives: Influence of Substituents in the Isoxazole and Phenyl Rings on the Antiviral Activity against Enteroviruses. *Euro. J. Med. Chem.* **2020**, *188*, 112007.
8. Sun, B.-F.; Hong, R.; Kang, Y.-B.; Deng, L. Asymmetric Total Synthesis of (−)-Plicatic Acid via a Highly Enantioselective and Diastereoselective Nucleophilic Epoxidation of Acyclic Trisubstitued Olefins. *J. Am. Chem. Soc.* **2009**, *131*, 10384–10385.
9. Buitrago, E.; Lundberg, H.; Andersson, H.; Ryberg, P.; Adolfsson, H. High Throughput Screening of a Catalyst Library for the Asymmetric Transfer Hydrogenation of Heteroaromatic Ketones: Formal Syntheses of (*R*)-Fluoxetine and (*S*)-Duloxetine. *ChemCatChem* **2012**, *4*, 2082–2089.
10. Akporji, N.; Singhania, V.; Dussart-Gautheret, J.; Gallou, F.; Lipshutz, B. H. Nanomicelle-Enhanced, Asymmetric ERED-Catalyzed Reductions of Activated Olefins. Applications to 1-Pot Chemo- and Bio-Catalysis Sequences in Water. *Chem. Commun.* **2021**, *57*, 11847–11850.
11. Cortes-Clerget, M.; Akporji, N.; Zhou, J.; Gao, F.; Guo, P.; Parmentier, M.; Gallou, F.; Berthon, J.-Y.; Lipshutz, B. H. Bridging the Gap between Transition Metal- and Bio-Catalysis via Aqueous Micellar Catalysis. *Nat. Commun.* **2019**, *10*, 2169.
12. Singhania, V.; Cortes-Clerget, M.; Dussart-Gautheret, J.; Akkachairin, B.; Yu, J.; Akporji, N.; Gallou, F.; Lipshutz, B. H. Lipase-Catalyzed Esterification in Water Enabled by Nanomicelles. Applications to 1-Pot Multi-Step Sequences. *Chem. Sci.* **2022**, *13*, 1440–1445.
13. Silva, R. M.; Okano, L. T.; Rodrigues, J. A. R.; Clososki, G. C. Extractive Biocatalysis in the Asymmetric Reduction of α-Alkyl, β-Aryl Enones by Baker's Yeast. *Tetrahedron: Asymmetry* **2017**, *28*, 939–944.
14. Secker, C.; Völkel, A.; Tiersch, B.; Koetz, J.; Schlaad, H. Thermo-Induced Aggregation and Crystallization of Block Copolypeptoids in Water. *Macromolecules* **2016**, *49*, 979–985.
15. Akporji, N.; Thakore, R. R.; Cortes-Clerget, M.; Andersen, J.; Landstrom, E.; Aue, D. H.; Gallou, F.; Lipshutz, B. H. N_2 Phos – an Easily Made, Highly Effective Ligand Designed for Ppm Level Pd-Catalyzed Suzuki–Miyaura Cross Couplings in Water. *Chem. Sci.* **2020**, *11* (20), 5205–5212.
16. Epp, J. B.; Alexander, A. L.; Balko, T. W.; Buysse, A. M.; Brewster, W. K.; Bryan, K.; Daeuble, J. F.; Fields, S. C.; Gast, R. E.; Green, R. A.; Irvine, N. M.; Lo, W. C.;

Lowe, C. T.; Renga, J. M.; Richburg, J. S.; Ruiz, J. M.; Satchivi, N. M.; Schmitzer, P. R.; Siddall, T. L.; Webster, J. D.; Weimer, M. R.; Whiteker, G. T.; Yerkes, C. N. The Discovery of Arylex™ Active and Rinskor™ Active: Two Novel Auxin Herbicides. *Bioorg. Med. Chem.* **2016**, *24*, 362–371.

17. Bradner, J.; Erb, M.; Qi, J. Compounds and Methods for Treating Cancer. U.S. Patent WO2016196879A1, December 8, 2016.
18. Zhang, Y.; Takale, B. S.; Gallou, F.; Reilly, J.; Lipshutz, B. H. Sustainable Ppm Level Palladium-Catalyzed Aminations in Nanoreactors under Mild, Aqueous Conditions. *Chem. Sci.* **2019**, *10*, 10556–10561.
19. Zhang, W.; Liu, Y.; Zhang, Y.; Wang, L.-J.; Wu, Y.-C.; Li, H.-J. 9-Substituted Acridines as Effective Corrosion Inhibitors for Mild Steel: Electrochemical, Surface Morphology, and Computational Studies. *New J. Chem.* **2020**, *44*, 6464–6474.
20. Dunny, E.; Doherty, W.; Evans, P.; Malthouse, J. P. G.; Nolan, D.; Knox, A. J. S. Vinyl Sulfone-Based Peptidomimetics as Anti-Trypanosomal Agents: Design, Synthesis, Biological and Computational Evaluation. *J. Med. Chem.* **2013**, *56*, 6638–6650.
21. Isley, N. A.; Linstadt, R. T. H.; Kelly, S. M.; Gallou, F.; Lipshutz, B. H. Nucleophilic Aromatic Substitution Reactions in Water Enabled by Micellar Catalysis. *Org. Lett.* **2015**, *17*, 4734–4737.
22. Wood, A. B.; Cortes-Clerget, M.; Kincaid, J. R. A.; Akkachairin, B.; Singhania, V.; Gallou, F.; Lipshutz, B. H. Nickel Nanoparticle Catalyzed Mono- and Di-Reductions of *Gem* -Dibromocyclopropanes Under Mild, Aqueous Micellar Conditions. *Angew. Chem., Int. Ed.* **2020**, *59*, 17587–17593.
23. Fialho, D. M.; Etemadi-Davan, E.; Langner, O. C.; Takale, B. S.; Gadakh, A.; Sambasivam, G.; Lipshutz, B. H. Copper-Catalyzed Asymmetric Reductions of Aryl/Heteroaryl Ketones under Mild Aqueous Micellar Conditions. *Org. Lett.* **2021**, *23*, 3282–3286.
24. Wood, A. B.; Nandiwale, K. Y.; Mo, Y.; Jin, B.; Pomberger, A.; Schultz, V. L.; Gallou, F.; Jensen, K. F.; Lipshutz, B. H. Continuous Flow Suzuki–Miyaura Couplings in Water under Micellar Conditions in a CSTR Cascade Catalyzed by Fe/Ppm Pd Nanoparticles. *Green Chem.* **2020**, *22*, 3441–3444.
25. Bhat, M. Y.; Ahmed, S.; Ahmed, Q. N. Tf_2O- and $Cu(OTf)_2$-Assisted Acylamination Reaction of Unactivated Alcohols with Nitriles: A One-Pot P(IV) Activation, Stereoretention in Cycloalkanols and Deprotection Approach. *J. Org. Chem.* **2022**, *87*, 11608–11624.
26. Yu, T.; Pang, H.; Cao, Y.; Gallou, F.; Lipshutz, B. H. Safe, Scalable, Inexpensive, and Mild Nickel-Catalyzed Migita-Like C−S Cross-Couplings in Recyclable Water. *Angew. Chem., Int. Ed.* **2021**, *60*, 3708–3713.

9. Experimental Data

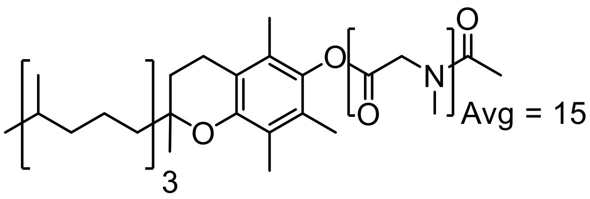

Savie (1):

^{1}H NMR (400 MHz, $CDCl_3$) δ(ppm) 4.50-3.80 (m, 36.6H, R-N(CH_3)-C**H_2**-C(O)-R), 3.27-2.79 (m, 54.9H, R–N(C**H_3**)–CH_2–C(O)–R), 2.57 (t, J = 6.8 Hz, 2H, Ar–C**H_2**–CH_2–R), 2.07 (m, 3H, R–N–C(O)–C**H_3**), 2.05 (s, 3H Ar–C**H_3**), 2.00 (s, 3H, Ar–C**H_3**), 1.96 (s, 3H, Ar–C**H_3**) 1.85-1.69 (m, 2H, Ar–CH_2–C**H_2**–R), 1.64-0.95 (m, 21H, R–C**H**(CH_3) –C**H_2**–R), 0.93-0.75 (m, 12H, R–CH(C**H_3**) –CH_2–R).

Yield: >99% (1.231 g). 0.8 mmol scale.

Physical appearance: Finely-divided, amorphous white powder.

NOTE: The ^{13}C NMR spectrum of this polydisperse oligomer is very complex, and thus not amenable to peak picking. The spectrum is provided in SI Section 10. See SI Fig. S4 for the MALDI TOF spectrum of Savie.

NOTE: We define the average polymer length to be the mode of the distribution as determined by MALDI TOF analysis. However, this differs from the mean polymer length, and this is reflected in the seemingly inflated ^{1}H NMR integrations which indicate a mean of ca. 18 repeating units.

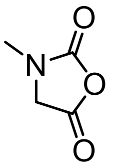

Sar-NCA (2):

^{1}H NMR (600 MHz, $CDCl_3$) δ(ppm) 4.13 (s, 2H), 3.05 (2, 3H).

^{13}C NMR (126 MHz, $CDCl_3$) δ(ppm) 165.5, 152.5, 51.1, 30.5.

Yield: 93% (4.295 g). 40 mmol scale.

Physical appearance: White crystalline solid.

Spectral data matched those previously reported.[14]

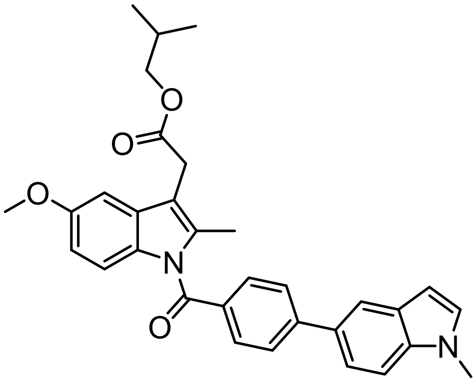

Isobutyl 2-(5-methoxy-2-methyl-1-(4-(1-methyl-1*H*-indol-5-yl)benzoyl)-1*H*-indol-3-yl) acetate (3):

^{1}H NMR (600 MHz, $CDCl_3$) δ(ppm) 7.93 (d, *J* = 1.8 Hz, 1H), 7.83 – 7.73 (m, 4H), 7.55 (dd, *J* = 8.5, 1.8 Hz, 1H), 7.42 (d, *J* = 8.5 Hz, 1H), 7.12 (d, *J* = 3.0 Hz, 1H), 7.01 (d, *J* = 9.0 Hz, 1H), 7.00 (d, *J* = 2.5 Hz, 1H), 6.68 (dd, *J* = 9.0, 2.6 Hz, 1H), 6.57 (dd, *J* = 3.1, 0.8 Hz, 1H), 3.90 (d, *J* = 6.6 Hz, 2H), 3.85 (d, *J* = 2.2 Hz, 6H), 3.70 (s, 2H), 2.44 (s, 3H), 1.92 (hept, *J* = 6.7 Hz, 1H), 0.91 (s, 3H), 0.90 (s, 3H).

^{13}C NMR (126 MHz, $CDCl_3$) δ(ppm) 171.3, 169.7, 156.0, 147.4, 137.0, 136.2, 133.2, 131.3, 131.3, 130.7, 130.1, 129.3, 127.5, 121.4, 120.1, 115.3, 112.3, 111.8, 110.0, 101.8, 101.2, 71.3, 55.9, 33.2, 30.7, 27.9, 19.3, 13.5.

mp: 122-123 °C**.**

Yield: Savie: 91% (115 mg) with co-solvent, 89% (113 mg) without co-solvent; TPGS-750-M: 96% (122 mg) with co-solvent, 67% (85 mg) without co-solvent. 0.25 mmol scale.

Physical appearance: Yellow solid.

R_f: 0.32 (25% EtOAc/hexanes).

Column conditions: Gradient 15-25% EtOAc/hexanes.

Spectral data matched those previously reported.[15]

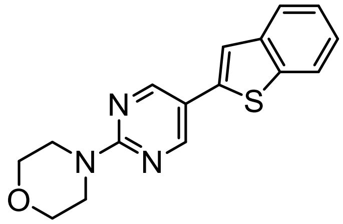

4-(5-(benzo[*b*]thiophen-2-yl)pyrimidin-2-yl)morpholine (4):

^{1}H NMR (600 MHz, CDCl$_3$) δ(ppm) 8.66 (s, 2H), 7.82 (d, *J* = 7.9 Hz, 1H), 7.76 (dd, *J* = 7.6, 1.2 Hz, 1H), 7.40 (s, 1H), 7.36 (ddd, *J* = 7.9, 7.1, 1.3 Hz, 1H), 7.31 (ddd, *J* = 8.4, 7.3, 1.4 Hz, 1H), 3.89 (t, *J* = 4.9 Hz, 4H), 3.84 – 3.76 (m, 4H).

^{13}C NMR (126 MHz, CDCl$_3$) δ(ppm) 161.1, 155.6, 140.8, 139.2, 138.5, 125.0, 124.5, 123.5, 122.4, 118.5, 117.9, 67.5, 67.0, 44.6.

mp: 196-198 °C.

HRMS (ESI): m/z calculated for $C_{16}H_{15}N_3OS+H^+$: 298.1014; $[M+H]^+$ found: 298.1015.

Yield: Savie: 83% (124 mg) with co-solvent, 82% (122 mg) without co-solvent; TPGS-750-M: 88% (131 mg) with co-solvent, 74% (110 mg) without co-solvent. 0.5 mmol scale.

Physical appearance: White solid.

R$_f$: 0.23 (15% EtOAc/hexanes).

Column conditions: Gradient 15-20% EtOAc/hexanes.

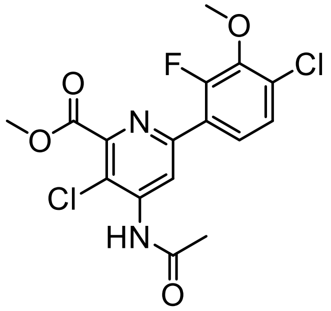

methyl 4-acetamido-3-chloro-6-(4-chloro-2-fluoro-3-methoxyphenyl)picolinate (5):

^{1}H NMR (600 MHz, CDCl$_3$) δ(ppm) 9.01 (s, 1H), 7.98 (s, 1H), 7.64 – 7.56 (m, 1H), 7.24 (dd, *J* = 8.6, 1.7 Hz, 1H), 4.01 (s, 3H), 3.99 (s, 3H), 2.33 (s, 3H).

^{13}C NMR (126 MHz, CDCl$_3$) δ(ppm) 169.0, 165.1, 155.6, 153.6, 151.7 (d, *J* = 1.8 Hz), 148.3, 145.1 (d, *J* = 14.2 Hz), 143.0, 130.0 (d, *J* = 3.2 Hz), 126.4 (d, *J* = 10.1 Hz), 125.5 (d, *J* = 3.7 Hz), 125.3 (d, *J* = 2.8 Hz), 116.9, 116.3 (d, *J* = 10.1 Hz), 61.9 (d, *J* = 4.6 Hz), 53.3, 25.3.

^{19}F NMR (471 MHz, $CDCl_3$) δ(ppm) -131.24 (d, *J* = 7.7 Hz).

mp: 131-132 °C.

Yield: Savie: 94% (182 mg); TPGS-750-M: 82% (158 mg). 0.5 mmol scale.

Physical appearance: White solid.

R_f: 0.23 (33% EtOAc/hexanes).

Column conditions: Gradient 10-60% EtOAc/hexanes.

Spectral data matched those previously reported.[16]

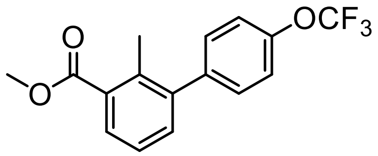

methyl 2-methyl-4'-(trifluoromethoxy)-[1,1'-biphenyl]-3-carboxylate (6):

^{1}H NMR (600 MHz, $CDCl_3$) δ(ppm) 7.79 (dd, *J* = 7.7, 1.7 Hz, 1H), 7.32 – 7.20 (m, 6H), 3.88 (s, 3H), 2.36 (s, 3H).

^{13}C NMR (126 MHz, $CDCl_3$) δ(ppm) 13C NMR (126 MHz, CDCl3) δ 168.9, 148.6 (q, *J* = 1.8 Hz), 142.5, 140.4, 136.8, 133.4, 131.7, 130.9, 129.8, 125.6, 120.9, 120.7 (q, *J* = 257.4 Hz), 52.3, 18.6.

^{19}F NMR (471 MHz, $CDCl_3$) δ(ppm) -57.79.

Yield: Savie: 95% (1.4734 g); TPGS-750-M: 85% (1.3204 g). 5 mmol scale.

Physical appearance: Colorless oil.

R_f: 0.29 (3% EtOAc/hexanes).

Column conditions: Gradient 0-2% EtOAc/hexanes.

Spectral data matched those previously reported.[17]

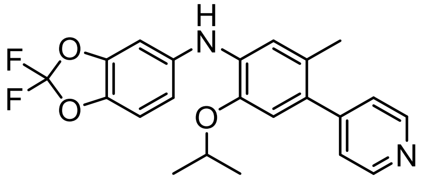

2,2-difluoro-*N*-(2-isopropoxy-5-methyl-4-(pyridin-4-yl)phenyl)benzo[*d*][1,3]dioxol-5-amine (7):

^{1}H NMR (400 MHz, $CDCl_3$) δ(ppm) 8.62 (dd, *J* = 4.4, 1.7 Hz, 2H), 7.34 – 7.20 (m, 2H), 7.07 – 6.94 (m, 3H), 6.86 (dd, *J* = 8.6, 2.2 Hz, 1H), 6.77 (s, 1H), 6.16 (s, 1H), 4.57 (hept, *J* = 6.1 Hz, 1H), 2.20 (s, 3H), 1.38 (d, *J* = 6.1 Hz, 6H).

^{13}C NMR (126 MHz, $CDCl_3$) δ(ppm) 150.0, 149.8, 144.6, 144.4, 139.2, 138.8, 134.7, 132.0 (t, *J* = 254.6 Hz), 130.3, 127.9, 124.6, 115.9, 115.4, 114.9, 110.0, 103.0, 71.7, 22.5, 20.1.

^{19}F NMR (376 MHz, $CDCl_3$) δ(ppm) -50.11.

mp: 107-109 °C.

HRMS (ESI): m/z calculated for $C_{22}H_{20}F_2N_2O_3+H^+$: 399.1515; $[M+H]^+$ found: 399.1520.

Yield: Savie: 95% (94.3 mg); TPGS-750-M: 95% (91.1 mg). 0.25 mmol scale.

Physical appearance: Orange solid.

R_f: 0.55 (5% MeOH/CH_2Cl_2).

Column conditions: Gradient 0-5% MeOH/CH_2Cl_2

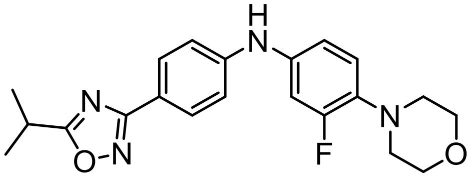

3-fluoro-*N*-(4-(5-isopropyl-1,2,4-oxadiazol-3-yl)phenyl)-4-morpholinoaniline (8):

^{1}H NMR (500 MHz, $CDCl_3$) δ(ppm) 7.91 (d, *J* = 8.7 Hz, 2H), 7.02 (d, *J* = 8.7 Hz, 2H), 6.96 – 6.83 (m, 3H), 6.16 (s, 1H), 3.90 (t, *J* = 4.7 Hz, 4H), 3.27 (hept, *J* = 7.0 Hz, 1H), 3.04 (bs, 4H), 1.43 (d, *J* = 7.0 Hz, 6H).

^{13}C NMR (126 MHz, $CDCl_3$) δ(ppm) 183.7, 168.1, 157.3, 155.3, 146.4, 137.1 (d, *J* = 10.1 Hz), 135.4 (d, *J* = 9.4 Hz), 129.1, 119.8 (d, *J* = 4.6 Hz), 118.7, 116.3 (d, *J* = 3.2 Hz), 115.8, 109.0 (d, *J* = 23.4 Hz), 67.3, 51.5 (d, *J* = 2.8 Hz), 27.72, 20.42.

^{19}F NMR (376 MHz, $CDCl_3$) δ(ppm) -121.15 (dd, *J* = 13.3, 8.5 Hz)

mp: 85-87 °C.

HRMS (ESI): m/z calculated for $C_{21}H_{23}FN_4O_2+H^+$: 383.1883; $[M+H]^+$ found: 383.1878.

Yield: Savie: 77% (73.5 mg); TPGS-750-M: 82% (93.3 mg). 0.25 mmol scale.

Physical appearance: Brown solid.

R_f: 0.30 (30% EtOAc/hexanes).

Column conditions: Gradient 10-30% EtOAc/hexanes.

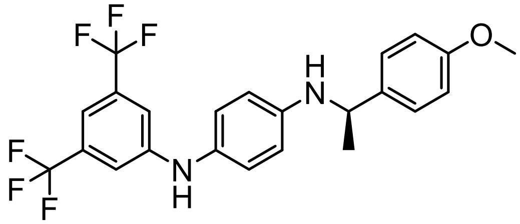

(*R*)-*N*[1]-(3,5-bis(trifluoromethyl)phenyl)-*N*[4]-(1-(4-methoxyphenyl)ethyl)benzene-1,4-diamine (9):

^{1}H NMR (500 MHz, $CDCl_3$) δ(ppm) 7.43 (d, *J* = 8.9 Hz, 2H), 7.26 (s, 1H), 7.12 (s, 2H), 7.07 – 6.93 (m, 4H), 6.62 (d, *J* = 8.9 Hz, 2H), 5.80 (s, 1H), 4.53 (q, *J* = 6.8 Hz, 1H), 4.30 (bs, 1H), 3.87 (s, 3H), 1.59 (d, *J* = 6.7 Hz, 3H).

^{13}C NMR (126 MHz, $CDCl_3$) δ(ppm) 158.8, 148.1, 145.5, 137.3, 132.5 (q, *J* = 32.6 Hz), 129.5, 127.1, 125.6, 123.8 (q, *J* = 272.5 Hz), 114.4, 114.3, 113.2 (q, *J* = 4.1 Hz), 111.0 (p, *J* = 3.9 Hz), 55.3, 53.3, 25.1.

^{19}F NMR (376 MHz, $CDCl_3$) δ(ppm) -63.0.

Yield: Savie: 97% (110.1 mg); TPGS-750-M: 82% (93.3 mg). 0.25 mmol scale.

Physical appearance: Brown oil.

R_f: 0.25 (20% EtOAc/hexanes).

Column conditions: Gradient 0-20% EtOAc/hexanes.

Spectral data matched those previously reported.[18]

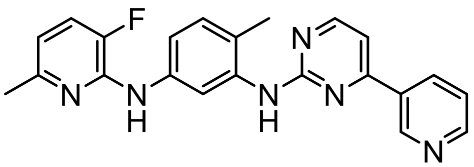

N^1-(3-fluoro-6-methylpyridin-2-yl)-4-methyl-N^3-(4-(pyridin-3-yl)pyrimidin-2-yl)benzene-1,3-diamine (10):

^{1}H NMR (400 MHz, $CDCl_3$) δ(ppm) 9.22 (dd, *J* = 2.3, 0.7 Hz, 1H), 8.68 (dd, *J* = 4.8, 1.8 Hz, 1H), 8.60 (d, *J* = 2.3 Hz, 1H), 8.51 (d, *J* = 5.3 Hz, 1H), 8.35 (dt, *J* = 7.9, 2.0 Hz, 1H), 7.38 (dd, *J* = 8.2, 2.3 Hz, 1H), 7.32 (ddd, *J* = 8.1, 4.8, 0.9 Hz, 1H), 7.21 – 7.03 (m, 4H), 6.64 (d, *J* = 3.8 Hz, 1H), 6.48 (dd, *J* = 7.9, 3.1 Hz, 1H), 2.31 (s, 6H).

^{13}C NMR (126 MHz, $CDCl_3$) δ(ppm) 162.7, 160.9, 159.2, 151.6 (d, *J* = 5.0 Hz), 151.6, 148.7, 146.4, 144.4 (d, *J* = 2.8 Hz), 144.4, 138.9, 137.8, 134.8, 132.9, 130.8, 123.7, 121.9, 121.2 (d, *J* = 16.1 Hz), 114.2, 113.2 (d, *J* = 2.3 Hz), 112.0, 108.2, 23.9, 17.7.

^{19}F NMR (376 MHz, $CDCl_3$) δ(ppm) -144.68 (d, *J* = 11.6 Hz).

mp: 161-163 °C.

HRMS (ESI): m/z calculated for $C_{22}H_{19}FN_6+H^+$: 387.1733; $[M+H]^+$ found: 387.1743.

Yield: Savie: 86% (83.1 mg); TPGS-750-M: 72% (69.7 mg). 0.25 mmol scale.

Physical appearance: Off-white solid.

R_f: 0.26 (70% EtOAc/hexanes).

Column conditions: Gradient 30-70% EtOAc/hexanes.

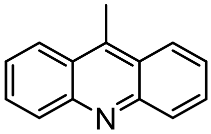

9-methylacridine (11):

^{1}H NMR (400 MHz, $CDCl_3$) δ(ppm) 8.30 – 8.14 (m, 4H), 7.76 (ddd, *J* = 8.8, 6.5, 1.4 Hz, 2H), 7.55 (ddd, *J* = 8.8, 6.6, 1.3 Hz, 2H), 3.12 (s, 3H).

^{13}C NMR (126 MHz, $CDCl_3$) δ(ppm) 148.6, 142.4, 130.4, 129.9, 125.7, 125.6, 124.7, 13.8.

mp: 118-119 °C.

Yield: Savie: >99% (97.0 mg); TPGS-750-M: 71% (68.5 mg). 0.5 mmol scale.

Physical appearance: Yellow solid.

R$_f$: 0.30 (30% EtOAc/hexanes).

Column conditions: 20% EtOAc/hexanes.

Spectral data matched those previously reported.[19]

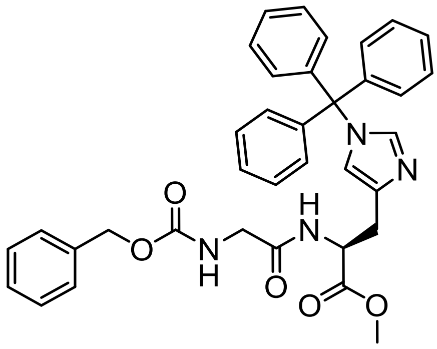

Methyl N^{α}-(((benzyloxy)carbonyl)glycyl)-N^{τ}-trityl-*L*-histidinate (12):

^{1}H NMR (600 MHz, CDCl$_3$) δ(ppm) 8.06-7.66 (d, *J* = 7.8 Hz, 1H), 7.53 – 7.16 (m, 15H), 7.15-7.02 (m, 6H), 6.54 (s, 1H), 5.51 (t, *J* = 5.6 Hz, 1H), 5.08 (s, 2H), 4.80 (dt, *J* = 7.8, 4.8 Hz, 1H), 4.06 – 3.79 (m, 2H), 3.59 (s, 3H), 3.04 (dd, *J* = 14.7, 5.0 Hz, 1H), 2.98 (dd, *J* = 14.7, 4.7 Hz, 1H).

^{13}C NMR (126 MHz, CDCl$_3$) δ(ppm) 171.6, 168.8, 156.4, 142.3, 138.8, 136.5, 136.5, 129.8, 128.6, 128.2, 128.2, 128.1, 128.0, 119.7, 75.4, 67.0, 52.7, 52.3, 44.4, 29.7.

HRMS (ESI): m/z calculated for $C_{36}H_{34}N_4O_5$+H^+: 603.2607; $[M+H]^+$ found: 603.2583.

Yield: Savie: 71% (214.2 mg); TPGS-750-M: 54% (163.4 mg); MC-1: 86% (260.2 mg). 0.5 mmol scale.

Physical appearance: White solid.

R$_f$: 0.19 (70% EtOAc/hexanes, CAM stain).

Column conditions: Gradient 40-85% EtOAc/hexanes.

NOTE: NMR spectra are messy as a result of a mixture of rotamers.

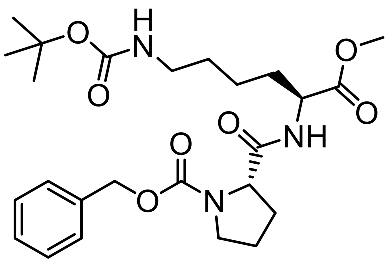

Benzyl (*S*)-2-(((*S*)-6-((*tert*-butoxycarbonyl)amino)-1-methoxy-1-oxohexan-2-yl)carbamoyl) pyrrolidine-1-carboxylate (13):

^{1}H NMR (400 MHz, CDCl$_3$) δ(ppm) 7.57-7.27 (m, 5H), 7.03 (s, 0.7H), 6.44 (s, 0.4H), 5.17 (s, 2H), 4.77 (s, 0.7H), 4.63-4.40 (m, 1.4H), 4.40 – 4.25 (m, 1H), 3.87 – 3.33 (m, 5H), 3.22-2.91 (m, 2H), 2.39 – 1.53 (m, 6H), 1.53-1.06 (m, 13H).

^{13}C NMR (126 MHz, CDCl$_3$) δ(ppm) 172.8, 171.7, 156.2, 136.6, 128.6, 128.2, 127.9, 79.1, 67.4, 60.5, 52.5, 52.2, 47.1, 40.2, 32.0, 31.2, 29.4, 28.7, 28.5, 24.7, 22.4.

Yield: Savie: 82% (201.4 mg); TPGS-750-M: 73% (178.7 mg); MC-1: 65% (160.2 mg). 0.5 mmol scale.

Physical appearance: Colorless viscous oil.

R_f: 0.39 (70% EtOAc/hexanes, CAM stain).

Column conditions: Gradient 40-80% EtOAc/hexanes.

Spectral data matched those previously reported.[20]

NOTE: NMR spectra are messy as a result of a mixture of rotamers.

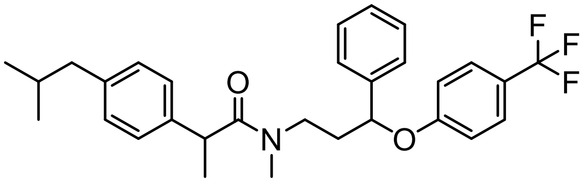

2-(4-isobutylphenyl)-*N*-methyl-*N*-(3-phenyl-3-(4-(trifluoromethyl)phenoxy)propyl) propenamide (14):

^{1}H NMR (400 MHz, CDCl$_3$) δ(ppm) 7.56 – 6.95 (m, 11H), 6.95 – 6.68 (m, 2H), 5.20 – 4.93 (m, 1H), 3.87 – 3.16 (m, 3H), 2.98 – 2.76 (m, 3H), 2.54-2.32 (m, 2H), 2.27 – 1.66 (m, 3H), 1.49 – 1.32 (m, 2H), 1.31 – 1.16 (m, 1H), 0.89 (d, J = 6.7 Hz, 6H).

^{13}C NMR (126 MHz, CDCl$_3$) δ(ppm) 174.2, 174.1, 174.1, 173.8, 160.6, 160.4, 160.3, 160.2, 141.1, 140.9, 140.5, 140.4, 140.4, 140.4, 140.2, 140.1, 139.6, 139.5, 139.3, 139.2, 131.2, 129.8, 129.8, 129.2, 129.2, 129.0, 129.0, 128.4, 128.3, 128.1, 127.8, 127.3, 127.2, 127.2, 127.2, 127.1,

127.1, 127.1, 127.0, 127.0, 126.9, 126.9, 126.9, 125.9, 125.9, 125.8, 125.7, 125.7, 125.6, 125.6, 125.5, 123.6, 123.5, 123.5, 123.5, 123.4, 123.4, 123.3, 123.3, 123.2, 123.1, 122.9, 122.9, 122.7, 122.6, 116.0, 115.8, 115.8, 78.5, 78.2, 77.9, 46.5, 46.1, 46.0, 45.8, 45.2, 43.4, 43.4, 43.1, 42.7, 37.5, 37.1, 36.7, 36.5, 36.4, 35.9, 34.1, 33.8, 32.1, 31.1, 30.4, 30.4, 30.2, 29.9, 29.9, 29.6, 22.6, 22.6, 22.6, 21.3, 21.0, 20.9, 20.8.

^{19}F NMR (376 MHz, $CDCl_3$) δ(ppm) -61.6.

HRMS (ESI): m/z calculated for $C_{30}H_{34}F_3NO_2$+H^+: 498.2620; $[M+H]^+$ found: 498.2620.

Yield: Savie: 88% (110.0 mg); TPGS-750-M: 73% (91.1 mg). 0.25 mmol scale.

Physical appearance: Colorless oil.

R_f: 0.54 (30% EtOAc/hexanes).

Column conditions: Gradient 0-10% EtOAc/hexanes.

NOTE: NMR spectra are messy as a result of a mixture of rotamers and a 50:50 mixture of diastereomers (since both coupling partners were racemic). J-coupling values for the ^{13}C spectrum could not be obtained due to its complexity; in lieu of this, all ^{13}C peaks are listed above.

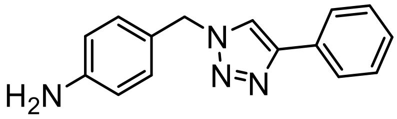

4-((4-phenyl-1*H*-1,2,3-triazol-1-yl)methyl)aniline (15):

^{1}H NMR (600 MHz, DMSO-*d6*) δ(ppm) 8.51 (s, 1H), 7.83 (dd, *J* = 8.3, 1.4 Hz, 2H), 7.46 – 7.38 (m, 2H), 7.31 (tt, *J* = 7.0, 1.4 Hz, 1H), 7.10 – 7.03 (m, 2H), 6.58 – 6.48 (m, 2H), 5.39 (s, 2H), 5.17 (s, 2H).

^{13}C NMR (126 MHz, DMSO-*d6*) δ(ppm) 148.8, 146.5, 130.8, 129.3, 128.9, 127.8, 125.1, 122.5, 120.9, 113.8, 53.1.

mp: 171-172 °C.

HRMS (ESI): m/z calculated for $C_{15}H_{14}N_4$+H^+: 273.1116; $[M+H]^+$ found: 273.1118.

Yield: Savie: >99% (62.3 mg); TPGS-750-M: 71% (44.2 mg). 0.25 mmol scale.

Physical appearance: Off-white crystalline solid.

R_f: 0.18 (40% EtOAc/hexanes).

Column conditions: 40% EtOAc/hexanes.

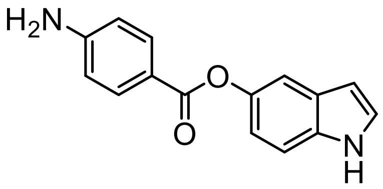

1*H*-indol-5-yl 4-aminobenzoate (16):

^{1}H NMR (400 MHz, DMSO-*d6*) δ(ppm) 11.15 (s, 1H), 7.88 – 7.73 (m, 2H), 7.39 (m, 2H), 7.29 (d, *J* = 2.3 Hz, 1H), 6.87 (dd, *J* = 8.6, 2.3 Hz, 1H), 6.69 – 6.56 (m, 2H), 6.42 (ddd, *J* = 3.1, 2.0, 1.0 Hz, 1H), 6.10 (s, 2H).

^{13}C NMR (126 MHz, DMSO-*d6*) δ(ppm) 165.4, 154.0, 144.0, 133.5, 131.7, 127.7, 126.5, 115.7, 115.2, 112.8, 112.3, 111.5, 101.2.

mp: 200-201 °C.

HRMS (ESI): m/z calculated for $C_{15}H_{12}N_2O_2+H^+$: 253.0977; $[M+H]^+$ found: 253.0983.

Yield: Savie: 80% (40.1 mg); TPGS-750-M: 61% (31.0 mg). 0.25 mmol scale.

Physical appearance: Yellow solid.

R_f: 0.26 (40% EtOAc/hexanes).

Column conditions: Gradient 0-40% EtOAc/hexanes.

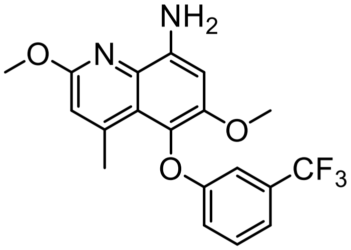

2,6-dimethoxy-4-methyl-5-(3-(trifluoromethyl)phenoxy)quinolin-8-amine (17):

^{1}H NMR (400 MHz, $CDCl_3$) δ(ppm) 7.34 (t, *J* = 7.7 Hz, 1H), 7.22 (m, 1H), 7.04 (t, *J* = 2.1 Hz, 1H), 6.93 (dd, *J* = 8.2, 2.6 Hz, 1H), 6.78 (s, 1H), 6.65 (m, 1H), 4.85 (s, 2H), 4.02 (s, 3H), 3.76 (s, 3H), 2.56 (d, *J* = 1.1 Hz, 3H).

^{13}C NMR (101 MHz, $CDCl_3$) δ(ppm) 159.8, 159.7, 148.6, 146.2, 141.5, 132.0 (q, *J* = 32.5 Hz), 131.2, 130.2, 128.5, 124.1 (q, *J* = 272.2 Hz), 120.9, 118.4, 118.1 (q, *J* = 4.0 Hz), 115.7, 112.1 (q, *J* = 4.0 Hz), 99.3, 56.6, 53.0, 23.1.

^{19}F NMR (376 MHz, $CDCl_3$) δ(ppm) -62.57.

mp: 113-115 °C.

HRMS (ESI): m/z calculated for $C_{19}H_{18}F_3N_2O_3$+H^+: 379.1270; $[M+H]^+$ found: 379.1281.

Yield: Savie: 60% (57.2 mg); TPGS-750-M: 34% (32.1 mg). 0.25 mmol scale.

Physical appearance: Yellow solid.

R_f: 0.38 (33% EtOAc/hexanes).

Column conditions: 30% EtOAc/hexanes.

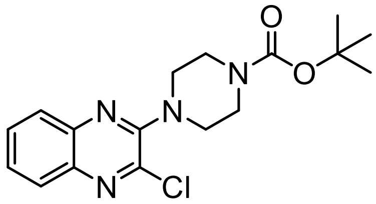

***Tert*-butyl 4-(3-chloroquinoxalin-2-yl)piperazine-1-carboxylate (18):**

^{1}H NMR (600 MHz, $CDCl_3$) δ(ppm) 7.89 (dt, *J* = 8.3, 0.8 Hz, 1H), 7.86 – 7.80 (m, 1H), 7.66 (ddt, *J* = 8.3, 7.0, 1.2 Hz, 1H), 7.56 (ddt, *J* = 8.3, 7.0, 1.2 Hz, 1H), 3.65 (dd, *J* = 6.3, 4.0 Hz, 4H), 3.57 – 3.45 (m, 4H), 1.50 (d, *J* = 0.9 Hz, 9H).

^{13}C NMR (126 MHz, $CDCl_3$) δ(ppm) 154.9, 152.6, 141.8, 140.2, 138.5, 130.3, 127.9, 127.7, 127.2, 80.1, 49.1, 28.6.

HRMS (ESI): m/z calculated for $[C_{17}H_{21}ClN_4O_2]_2$+Na^+: 719.2604; $[2M+Na]^+$ found: 719.2619.

Yield: Savie: 90% (62.5 mg); TPGS-750-M: 90% (62.6 mg). 0.25 mmol scale.

Physical appearance: Viscous yellow oil.

R_f: 0.27 (10% EtOAc/hexanes).

Column conditions: Gradient 0-5% EtOAc/hexanes.

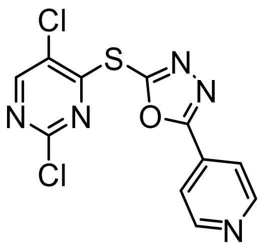

2-((2,5-dichloropyrimidin-4-yl)thio)-5-(pyridin-4-yl)-1,3,4-oxadiazole (19):

^{1}H NMR (600 MHz, $CDCl_3$) δ(ppm) 8.88 (dd, *J* = 4.4, 1.7 Hz, 2H), 8.45 (s, 1H), 8.01 – 7.95 (m, 2H).

^{13}C NMR (126 MHz, $CDCl_3$) δ(ppm) 167.3, 165.9, 158.7, 156.8, 156.7, 151.1, 130.7, 126.7, 120.8.

mp: 181-183 °C.

HRMS (ESI): m/z calculated for $[C_{11}H_5Cl_2N_5OS]_2+Na^+$: 674.9054; $[2M+Na]^+$ found: 674.9039.

Yield: Savie: 87% (143.3 mg); TPGS-750-M: 85% (140.1 mg). 0.5 mmol scale.

Physical appearance: White solid.

R_f: 0.22 (40% EtOAc/hexanes).

Column conditions: 3% $MeOH/CH_2Cl_2$

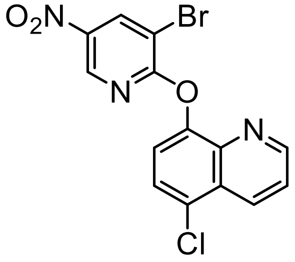

8-((3-bromo-5-nitropyridin-2-yl)oxy)-5-chloroquinoline (20):

^{1}H NMR (600 MHz, $CDCl_3$) δ(ppm) 8.79-8.76 (m, 2H), 8.71 (d, *J* = 2.4 Hz, 1H), 8.60 (dd, *J* = 8.6, 1.7 Hz, 1H), 7.70 (d, *J* = 8.1 Hz, 1H), 7.58 – 7.48 (m, 2H).

^{13}C NMR (126 MHz, $CDCl_3$) δ(ppm) 164.2, 151.1, 148.6, 142.6, 141.6, 140.5, 137.8, 133.5, 129.4, 127.9, 126.5, 122.8, 121.3, 107.0.

mp: 181-182 °C.

Yield: Savie: 93% (70.5 mg); TPGS-750-M: 88% (64.3 mg). 0.25 mmol scale.

Physical appearance: White solid.

R$_f$: 0.23 (10% EtOAc/hexanes).

Column conditions: Gradient 0-10% EtOAc/hexanes.

Spectral data matched those previously reported.[21]

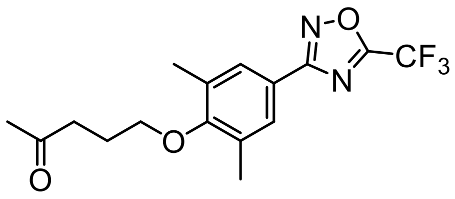

5-(2,6-dimethyl-4-(5-(trifluoromethyl)-1,2,4-oxadiazol-3-yl)phenoxy)pentan-2-one (21):

^{1}H NMR (400 MHz, CDCl$_3$) δ(ppm) 7.76 (s, 2H), 3.82 (t, *J* = 6.2 Hz, 2H), 2.74 (t, *J* = 7.2 Hz, 2H), 2.32 (s, 6H), 2.21 (s, 3H), 2.15 – 2.05 (m, 2H).

^{13}C NMR (126 MHz, CDCl$_3$) δ(ppm) 208.2, 169.1, 165.8 (q, *J* = 44.4 Hz), 159.5, 132.3, 128.6, 120.3, 116.2 (q, *J* = 273.5 Hz), 71.3, 40.0, 30.2, 24.6, 16.5.

^{19}F NMR (376 MHz, CDCl$_3$) δ(ppm) -65.4.

mp: 60-61 °C.

HRMS (CI): m/z calculated for $C_{16}H_{17}F_3N_2O_3+H^+$: 343.1270; $[M+H]^+$ found: 343.1261.

Yield: Savie: 73% (125.1 mg); TPGS-750-M: 69% (118.1 mg). 0.5 mmol scale.

Physical appearance: White solid.

R$_f$: 0.48 (30% EtOAc/hexanes).

Column conditions: Gradient 0-30% EtOAc/hexanes.

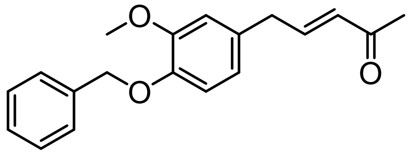

(*E*)-5-(4-(benzyloxy)-3-methoxyphenyl)pent-3-en-2-one (22):

^{1}H NMR (600 MHz, CDCl$_3$) δ(ppm) 7.46 – 7.40 (m, 2H), 7.40 – 7.33 (m, 2H), 7.32 – 7.28 (m, 1H), 6.89 (dt, *J* = 15.8, 6.8 Hz, 1H), 6.83 (d, *J* = 8.2 Hz, 1H), 6.70 (d, *J* = 2.1 Hz, 1H), 6.65 (dd, *J*

= 8.1, 2.1 Hz, 1H), 6.07 (dt, *J* = 15.8, 1.6 Hz, 1H), 5.14 (s, 2H), 3.88 (s, 3H), 3.47 (dd, *J* = 6.8, 1.7 Hz, 2H), 2.24 (s, 3H).

^{13}C NMR (126 MHz, $CDCl_3$) δ(ppm) 198.8, 150.0, 147.2, 146.8, 137.4, 132.0, 130.9, 128.7, 128.0, 127.4, 121.0, 114.6, 112.8, 71.3, 56.2, 38.6, 27.1.

mp: 53-55 °C.

HRMS (ESI): m/z calculated for $C_{19}H_{20}O_3+Na^+$: 319.1310; $[M+Na]^+$ found: 319.1298.

Yield: Savie: 87% (122.4 mg); TPGS-750-M: 87% (122.3 mg). 0.5 mmol scale.

Physical appearance: White solid.

R$_f$: 0.24 (25% EtOAc/hexanes).

Column conditions: Gradient 0-25% EtOAc/hexanes.

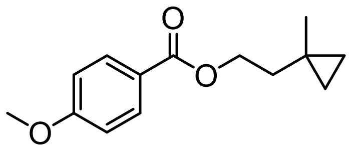

2-(1-methylcyclopropyl)ethyl 4-methoxybenzoate (23):

^{1}H NMR (600 MHz, $CDCl_3$) δ(ppm) 8.02 – 7.96 (m, 2H), 6.95 – 6.88 (m, 2H), 4.40 (t, *J* = 6.9 Hz, 2H), 3.86 (s, 3H), 1.69 (t, *J* = 6.9 Hz, 2H), 1.11 (s, 3H), 0.36 (q, *J* = 3.8 Hz, 2H), 0.29 (q, *J* = 3.8 Hz, 2H).

^{13}C NMR (126 MHz, $CDCl_3$) δ(ppm) 166.6, 163.4, 131.7, 123.2, 113.8, 63.6, 55.6, 38.2, 23.0, 13.3, 13.0.

Yield: Savie: 93% (43.6 mg); TPGS-750-M: 91% (42.6 mg). 0.25 mmol scale.

Physical appearance: Tan oil.

R$_f$: 0.31 (5% EtOAc/hexanes).

Column conditions: 5% EtOAc/hexanes.

Spectral data matched those previously reported.[22]

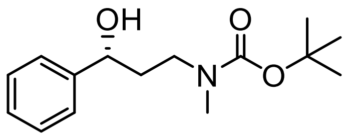

tert-butyl (R)-(3-hydroxy-3-phenylpropyl)(methyl)carbamate (24):

^{1}H NMR (500 MHz, CD_3OD) δ(ppm) 7.35 – 7.23 (m, 4H), 7.23 – 7.09 (m, 1H), 4.55 (t, J = 6.6 Hz, 1H), 3.21 (bm, 2H), 2.78 (s, 3H), 1.97 – 1.75 (bm, 2H), 1.37 (bs, 9H).

^{13}C NMR (126 MHz, CD_3OD) δ(ppm) 157.8 (b), 146.2, 129.5, 128.5, 127.1, 81.1, 72.9, 47.3 (b), 38.4 (b), 34.9 (b), 28.9.

Yield: Savie: 79% (83.5 mg); TPGS-750-M: 73% (77.5 mg). 0.4 mmol scale.

Physical appearance: White solid.

R_f: 0.18 (25% EtOAc/hexanes).

Column conditions: 25% EtOAc/hexanes.

Spectral data matched those previously reported.[23]

The ee was determined by HPLC analysis on Chiralpak OD-H column, hexane:IPA = 95/5; flow rate = 1 mL/min; UV detection at 210 nm (for TPGS-750-M samples) or 204 nm (for Savie samples); t_R(R) = 7.70 min (major), t_R(S) = 8.84 min (minor), ee = 93% for products formed in both Savie and TPGS-750-M. HPLC chromatograms are shown below for the racemic standards (first and third traces) and enantioenriched products (second [TPGS-750-M] and fourth [Savie] traces).

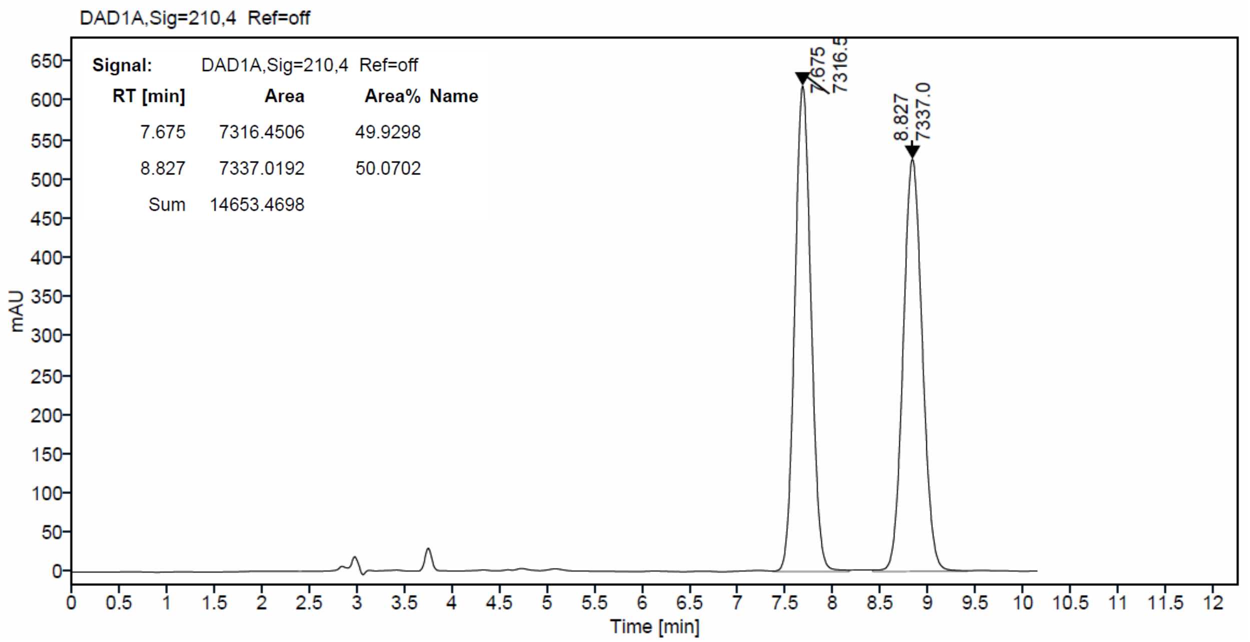

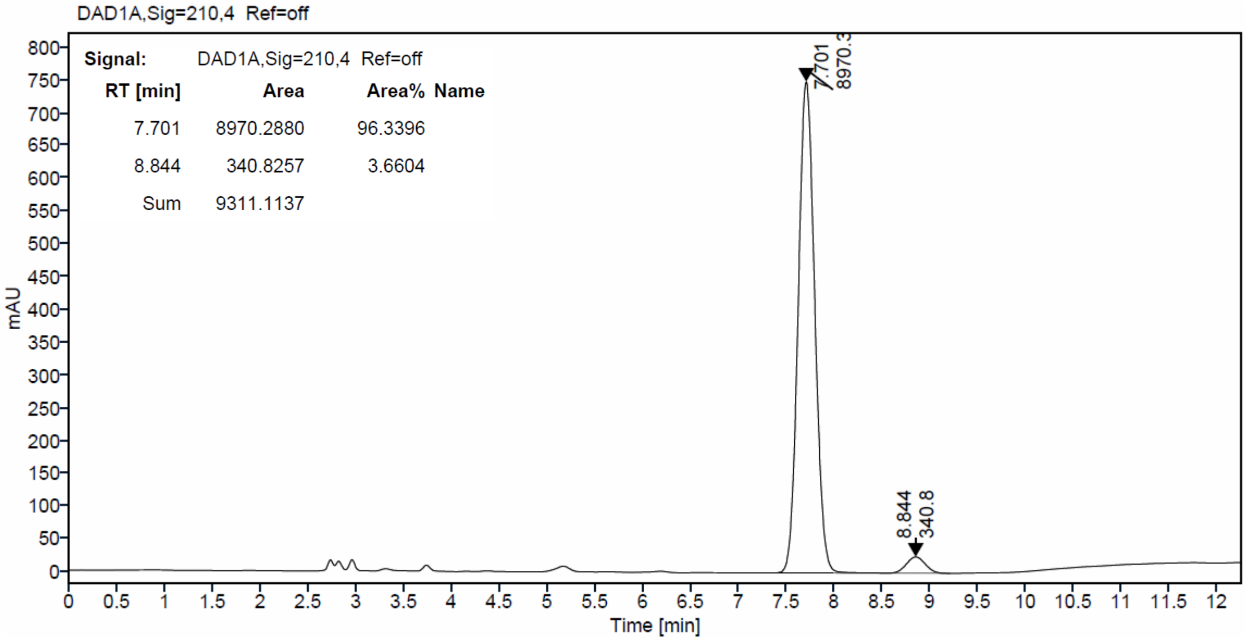
DAD1A,Sig=210,4 Ref=off
Signal: DAD1A,Sig=210,4 Ref=off
RT [min] Area Area% Name
7.701 8970.2880 96.3396
8.844 340.8257 3.6604
Sum 9311.1137
mAU
Time [min]
7.701 8970.3
8.844 340.8

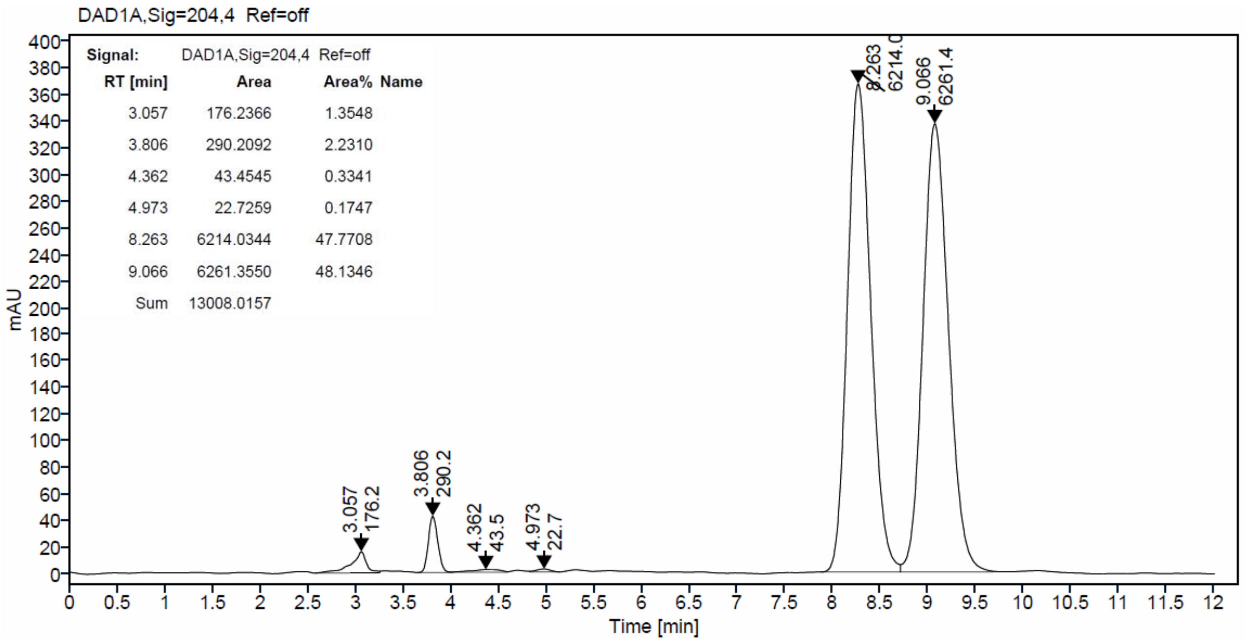
DAD1A,Sig=204,4 Ref=off
Signal: DAD1A,Sig=204,4 Ref=off
RT [min] Area Area% Name
3.057 176.2366 1.3548
3.806 290.2092 2.2310
4.362 43.4545 0.3341
4.973 22.7259 0.1747
8.263 6214.0344 47.7708
9.066 6261.3550 48.1346
Sum 13008.0157
mAU
Time [min]
3.057 176.2
3.806 290.2
4.362 43.5
4.973 22.7
9.066 6261.4

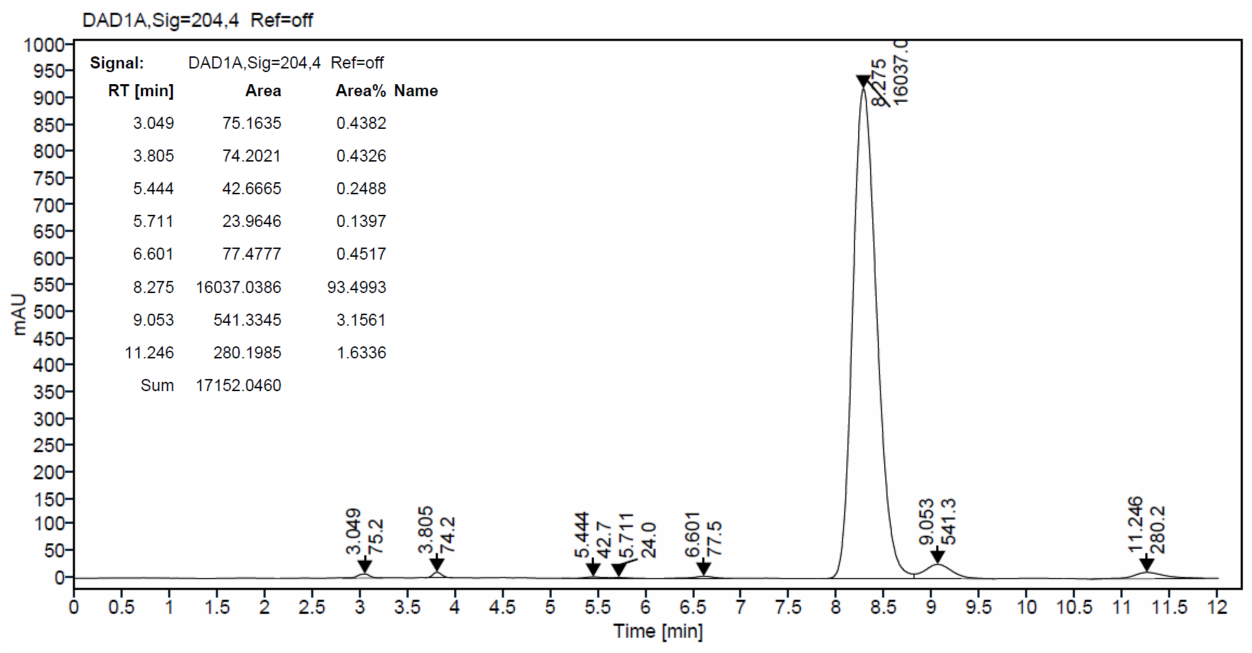

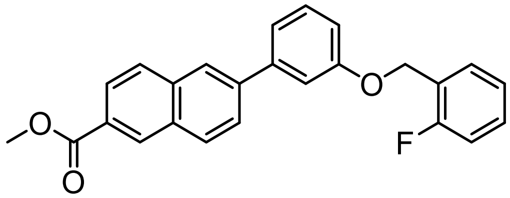

Methyl 6-(3-((2-fluorobenzyl)oxy)phenyl)-2-naphthoate (25):

^{1}H NMR (600 MHz, $CDCl_3$) δ(ppm) 8.63 (dd, *J* = 1.7, 0.7 Hz, 1H), 8.09 (dd, *J* = 8.6, 1.7 Hz, 1H), 8.08 – 8.05 (m, 1H), 8.02 (d, *J* = 8.4 Hz, 1H), 7.93 (d, *J* = 8.6 Hz, 1H), 7.80 (dd, *J* = 8.5, 1.8 Hz, 1H), 7.57 (td, *J* = 7.5, 1.9 Hz, 1H), 7.43 (t, *J* = 7.9 Hz, 1H), 7.38 – 7.30 (m, 3H), 7.19 (td, *J* = 7.5, 1.2 Hz, 1H), 7.12 (ddd, *J* = 9.7, 8.3, 1.3 Hz, 1H), 7.04 (ddd, *J* = 8.2, 2.6, 1.0 Hz, 1H), 5.24 (s, 2H), 4.00 (s, 3H).

^{13}C NMR (126 MHz, $CDCl_3$) δ(ppm) 167.4, 160.7 (d, J = 246.8 Hz), 159.2, 142.3, 140.8, 135.9, 131.9, 131.0, 130.2, 130.0, 130.0, 130.0, 130.0 (d, J = 0.8 Hz), 128.6, 127.6, 126.5, 125.9 (d, J = 8.3 Hz), 124.5 (d, J = 3.7 Hz), 124.2 (d, J = 14.2 Hz), 120.6, 115.6 (d, J = 21.1 Hz), 114.4, 114.2, 64.0 (d, J = 4.1 Hz), 52.4.

^{19}F NMR (471 MHz, $CDCl_3$) δ(ppm) -118.57.

mp: 112-113 °C.

HRMS (ESI): m/z calculated for $C_{25}H_{19}FO_3^+$: 386.1318; $[M]^+$ found: 386.1331.

Yield: Savie: 74% (71.1 mg); TPGS-750-M: 43% (41.1 mg). 0.25 mmol scale.

Physical appearance: White solid.

R_f: 0.19 (5% EtOAc/hexanes).

Column conditions: Gradient 0-4% EtOAc/hexanes.

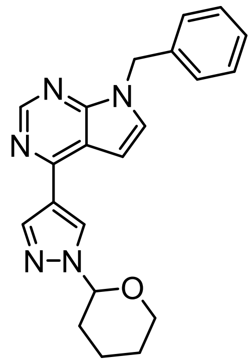

7-Benzyl-4-(1-(tetrahydro-2*H*-pyran-2-yl)-1*H*-pyrazol-4-yl)-7*H*-pyrrolo[2,3-*d*]pyrimidine (26):

^{1}H NMR (600 MHz, $CDCl_3$) δ(ppm) 8.86 (s, 1H), 8.42 (s, 1H), 8.29 (s, 1H), 7.35 – 7.24 (m, 3H), 7.24 – 7.20 (m, 2H), 7.19 (d, J = 3.6 Hz, 1H), 6.75 (d, J = 3.6 Hz, 1H), 5.53 – 5.40 (m, 3H), 4.09 (ddt, J = 11.7, 3.0, 1.7 Hz, 1H), 3.74 (td, J = 11.4, 2.9 Hz, 1H), 2.32 – 2.10 (m, 2H), 2.05 (dtq, J = 10.2, 4.3, 2.2 Hz, 1H), 1.80 – 1.56 (m, 3H).

^{13}C NMR (126 MHz, $CDCl_3$) δ(ppm) 151.8, 151.7, 151.1, 139.5, 137.0, 129.0, 128.6, 128.5, 128.1, 127.7, 122.1, 114.3, 100.3, 88.0, 67.9, 48.0, 30.7, 25.1, 22.3.

mp: 100-101 °C.

Yield: Savie: 98% (176.7 mg); TPGS-750-M: 75% (135.4 mg). 0.5 mmol scale.

Physical appearance: White solid.

R_f: 0.23 (50% EtOAc/hexanes).

Column conditions: Gradient 0-50% EtOAc/hexanes.

Spectral data matched those previously reported.[24]

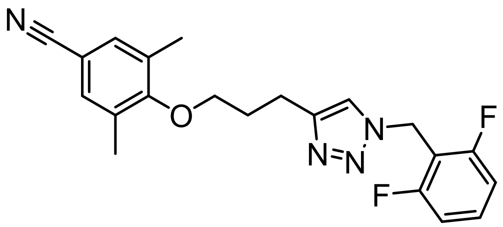

4-(3-(1-(2,6-difluorobenzyl)-1*H*-1,2,3-triazol-4-yl)propoxy)-3,5-dimethylbenzonitrile (27):

^{1}H NMR (600 MHz, $CDCl_3$) δ(ppm) 7.40 – 7.33 (m, 2H), 7.29 (s, 2H), 7.00 – 6.93 (m, 2H), 5.60 (s, 2H), 3.82 (t, *J* = 6.3 Hz, 2H), 2.93 (t, *J* = 7.6 Hz, 2H), 2.23 (s, 6H), 2.22 – 2.16 (m, 2H).

^{13}C NMR (126 MHz, $CDCl_3$) δ(ppm) 161.5 (dd, *J* = 251.2, 7.1 Hz), 159.9, 147.5, 132.8, 132.7, 131.5 (dd, apparent triplet, *J* = 10.3 Hz), 121.0, 119.2, 111.9 (dd, *J* = 20.2 Hz, 5.1 Hz), 111.1 (dd, apparent triplet, *J* = 19.1 Hz), 107.2, 71.5, 41.3 (dd, apparent triplet, *J* = 4.1 Hz), 29.9, 22.2, 16.3.

^{19}F NMR (471 MHz, $CDCl_3$) δ(ppm) -114.23.

mp: 79-80 °C.

HRMS (ESI): m/z calculated for $C_{21}H_{20}F_2N_4O+H^+$: 383.1683; $[M+H]^+$ found: 383.1689.

Yield: Savie: 97% (185.9 mg); TPGS-750-M: 96% (182.6 mg). 0.5 mmol scale.

Physical appearance: White solid.

R_f: 0.32 (40% EtOAc/hexanes).

Column conditions: 40% EtOAc/hexanes.

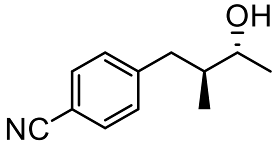

4-((2*S*,3*R*)-3-hydroxy-2-methylbutyl)benzonitrile (31):

^{1}H NMR (400 MHz, $CDCl_3$) δ(ppm) 7.61 – 7.53 (m, 2H), 7.32-7.23 (m, 2H), 3.66 (p, *J* = 6.1 Hz, 1H), 2.99 (dd, *J* = 13.3, 5.1 Hz, 1H), 2.40 (dd, *J* = 13.4, 9.6 Hz, 1H), 1.87 – 1.73 (m, 1H), 1.38 (s(br), 1H), 1.28 – 1.16 (m, 4H), 0.81 (dd, J = 6.8 Hz, 3H).

^{13}C NMR (126 MHz, $CDCl_3$) δ(ppm) 147.2, 132.3, 130.2, 119.3, 109.9, 71.5, 42.3, 39.3, 20.6, 15.0.

Yield: Savie: 70% (26.4 mg); TPGS-750-M: 54% (20.4 mg). 0.2 mmol scale.

Physical appearance: Colorless oil.

R_f: 0.37 (40% EtOAc/hexanes).

Column conditions: Gradient 0-25% EtOAc/hexanes.

Spectral data matched those previously reported.[10]

NOTE: NMR spectra are messy due to the presence of diastereomers.

The ee was determined by HPLC analysis on Chiracel OD-H column, 98:2 = hexane/IPA; flow rate = 0.7 mL/min; UV detection at 230 nm; t_1 = 52.31 min (minor), t_2 = 54.02 min (major). HPLC chromatograms are shown below for the racemic standard (top) and enantioenriched products (TPGS-750-M sample in middle, Savie sample on bottom). The HPLC data of the racemic material show both diastereomers in roughly a 1.2:1 ratio. The products (bottom two traces) show three of the four enantiomers of the two possible diastereomers. However, the first two peaks at 49.8 and 52.3 min correspond to one of the two diastereomers, while the largest peak is the only one observed for the major isomer. Hence, the ee is >99% for both product samples. The dr for the sample run in 2 wt% TPGS-750-M/H_2O is 88:12, and that of the sample run in 2 wt % Savie/H_2O is 89:11.

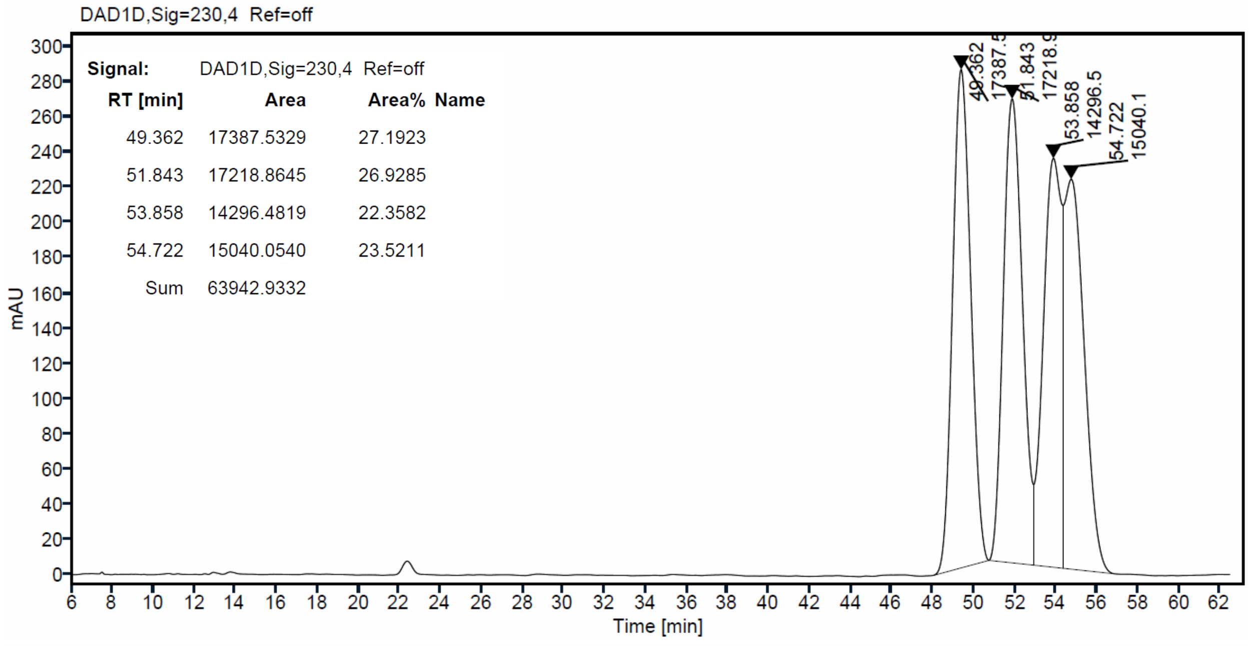

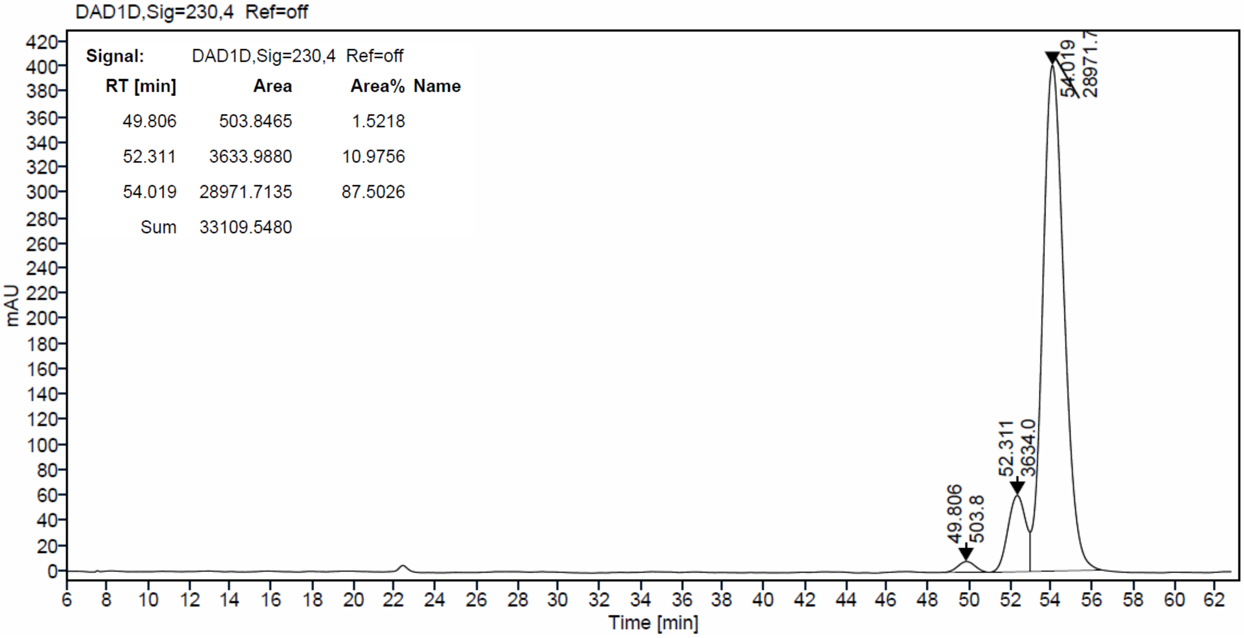
DAD1D,Sig=230,4 Ref=off
Signal: DAD1D,Sig=230,4 Ref=off
RT [min] Area Area% Name
49.806 503.8465 1.5218
52.311 3633.9880 10.9756
54.019 28971.7135 87.5026
Sum 33109.5480
49.806
503.8
52.311
3634.0
54.019
mAU
Time [min]

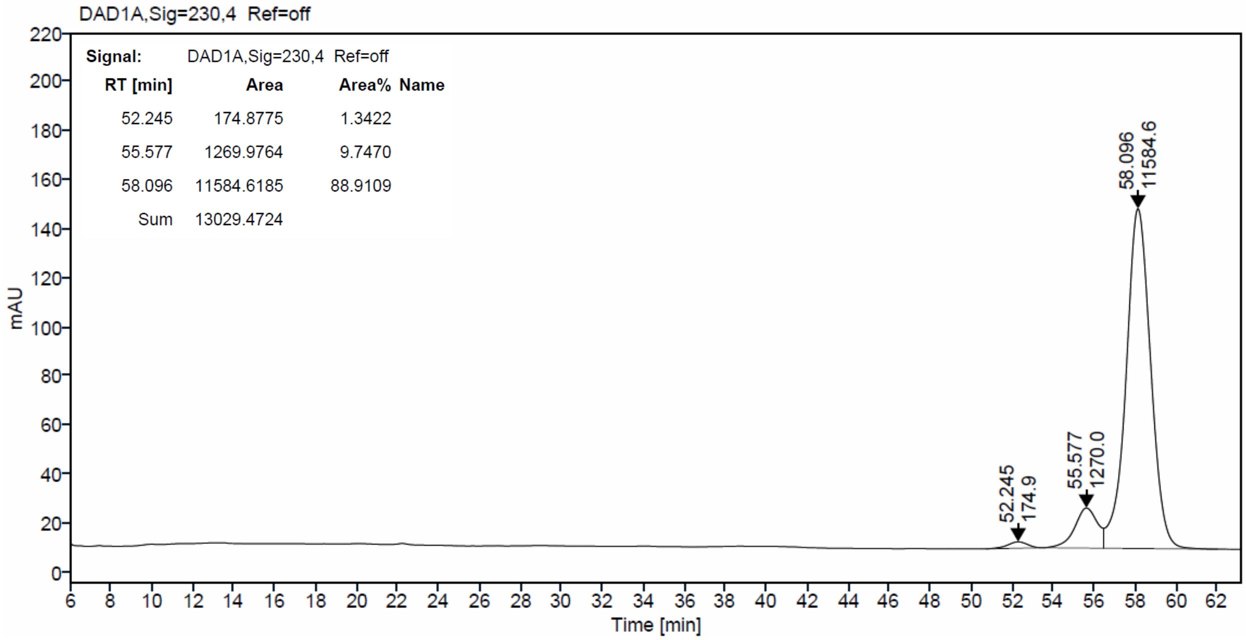
DAD1A,Sig=230,4 Ref=off
Signal: DAD1A,Sig=230,4 Ref=off
RT [min] Area Area% Name
52.245 174.8775 1.3422
55.577 1269.9764 9.7470
58.096 11584.6185 88.9109
Sum 13029.4724
52.245
174.9
55.577
1270.0
58.096
11584.6
mAU
Time [min]

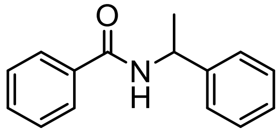

***N*-(1-phenylethyl)benzamide (32):**

^{1}H NMR (600 MHz, $CDCl_3$) δ(ppm) 7.81 – 7.74 (m, 2H), 7.53 – 7.46 (m, 1H), 7.45 – 7.38 (m, 4H), 7.38 – 7.33 (m, 2H), 7.31 – 7.26 (m, 1H), 6.42 – 6.26 (m, 1H), 5.35 (p, *J* = 7.0 Hz, 1H), 1.62 (d, *J* = 6.9 Hz, 3H).

^{13}C NMR (126 MHz, $CDCl_3$) δ(ppm) 166.8, 143.4, 134.7, 131.6, 128.9, 128.7, 127.6, 127.1, 126.4, 49.4, 21.9.

mp: 121 °C.

Yield: Savie: 96% (1.0814 g). 5 mmol scale.

Physical appearance: White solid.

R_f: 0.29 (25% EtOAc/hexanes).

Spectral data matched those previously reported.[25]

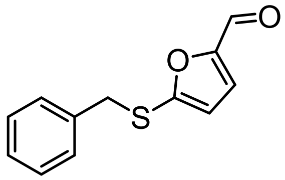

5-(Benzylthio)furan-2-carbaldehyde (33):

^{1}H NMR (400 MHz, $CDCl_3$) δ(ppm) 9.54 (s, 1H), 7.35 – 7.22 (m, 5H), 7.16 (d, *J* = 3.5 Hz, 1H), 6.38 (d, *J* = 3.7 Hz, 1H), 4.21 (s, 2H).

^{13}C NMR (126 MHz, $CDCl_3$) δ(ppm) 176.6, 155.0, 154.3, 136.6, 129.0, 128.8, 127.9, 122.9, 115.7, 38.7.

Yield: 91% (49.6 mg). 0.25 mmol scale.

Physical appearance: Brown oil.

R_f: 0.26 (10% EtOAc/hexanes).

Column conditions: 10% EtOAc/hexanes.

Spectral data matched those previously reported.[26]

10. ^{1}H, ^{13}C, and ^{19}F NMR Spectra of Synthesized Products

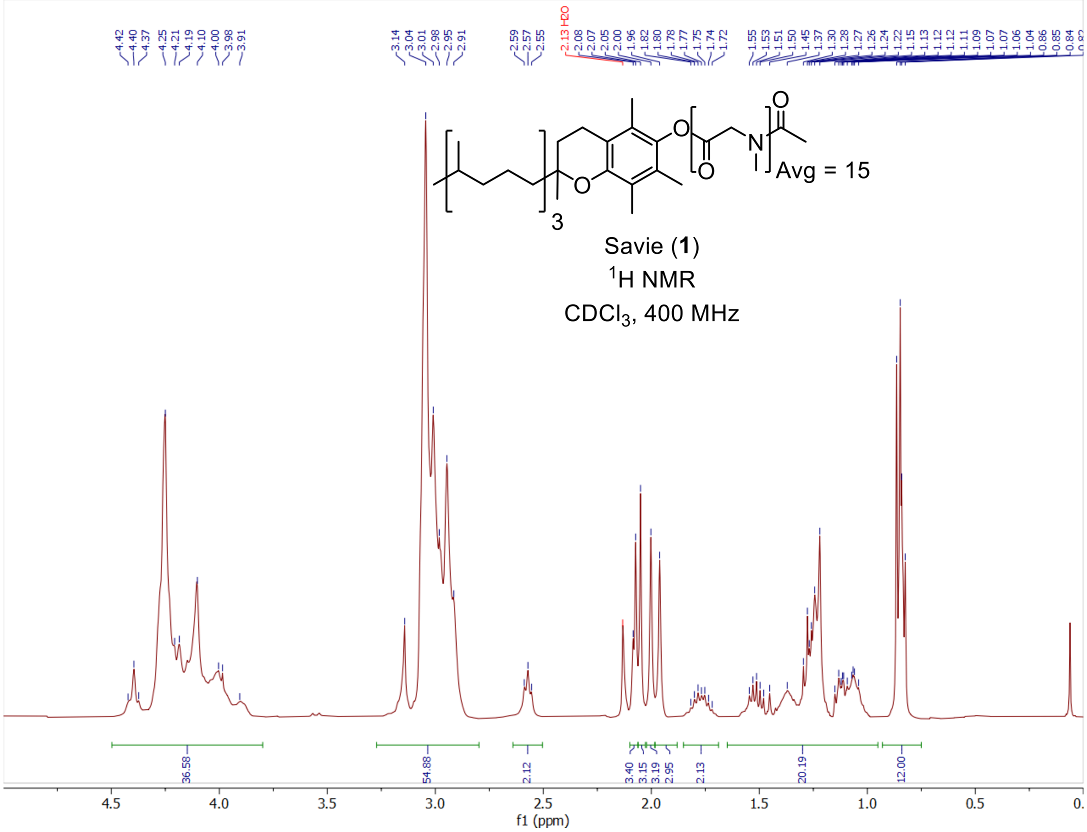

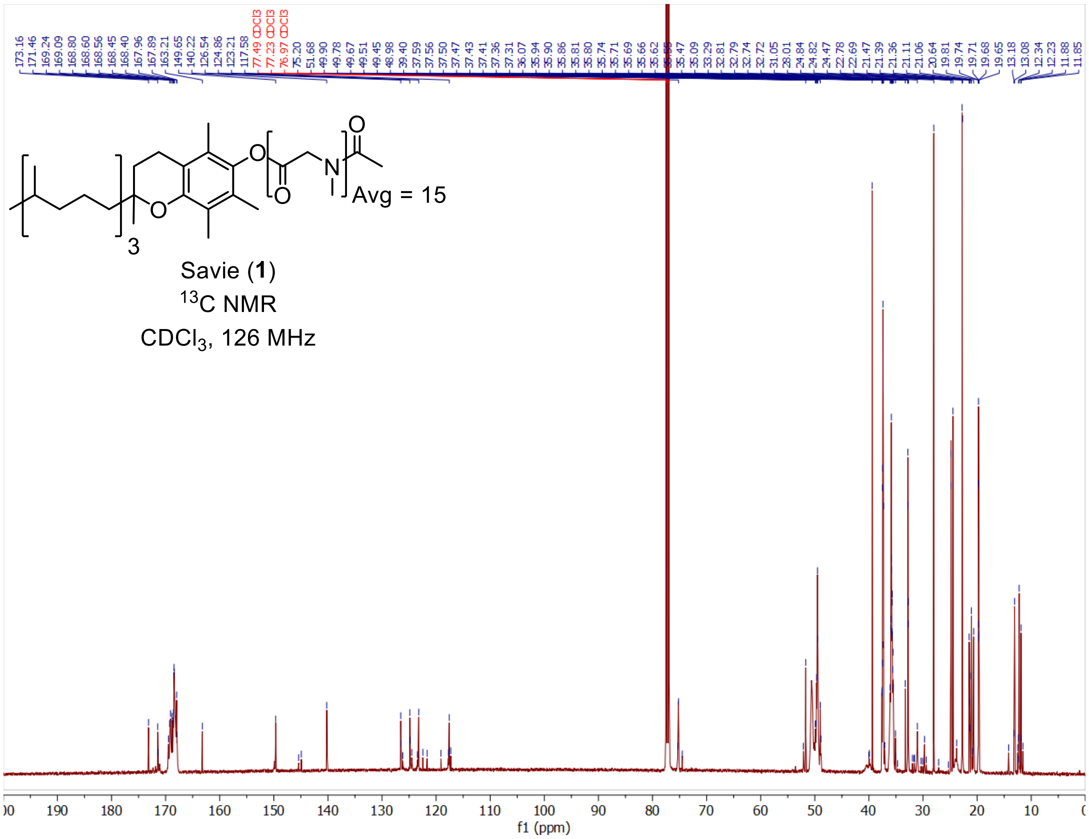

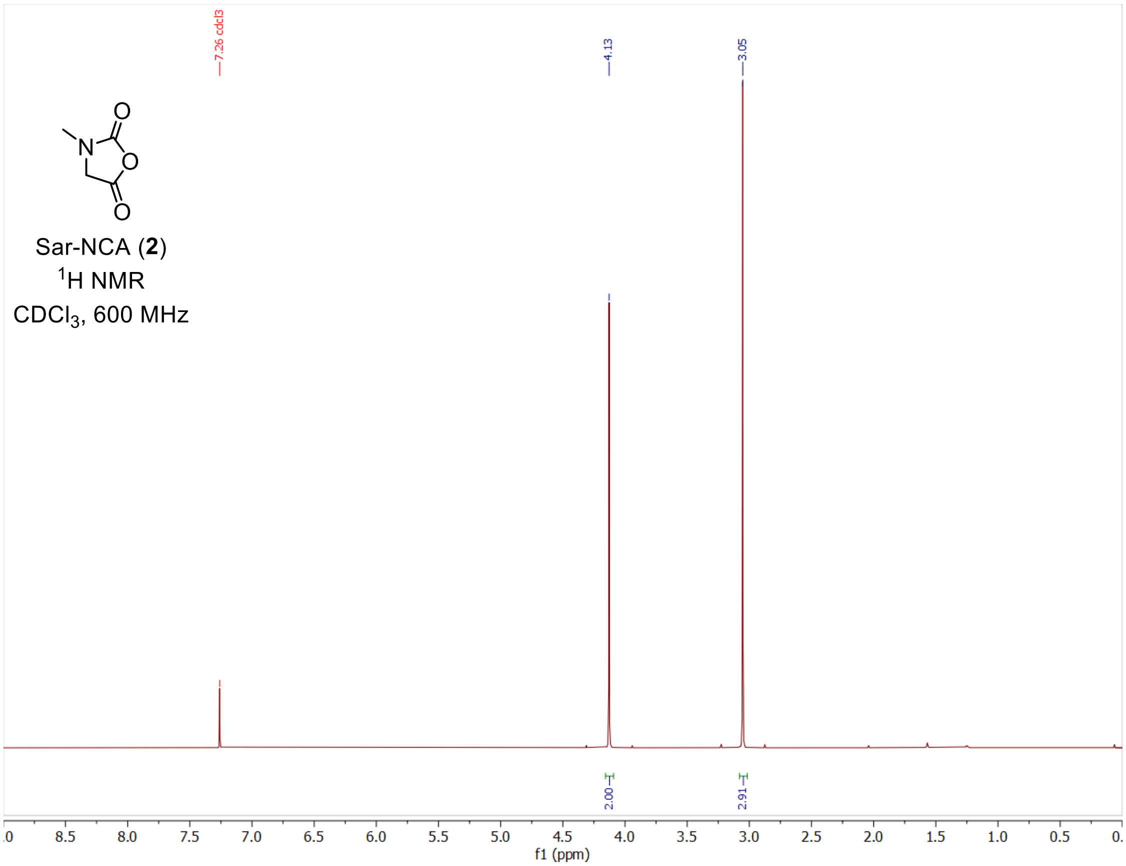

7.26 cdcl3
4.13
3.05
Sar-NCA (2)
1H NMR
CDCl3, 600 MHz
2.00
2.91
f1 (ppm)

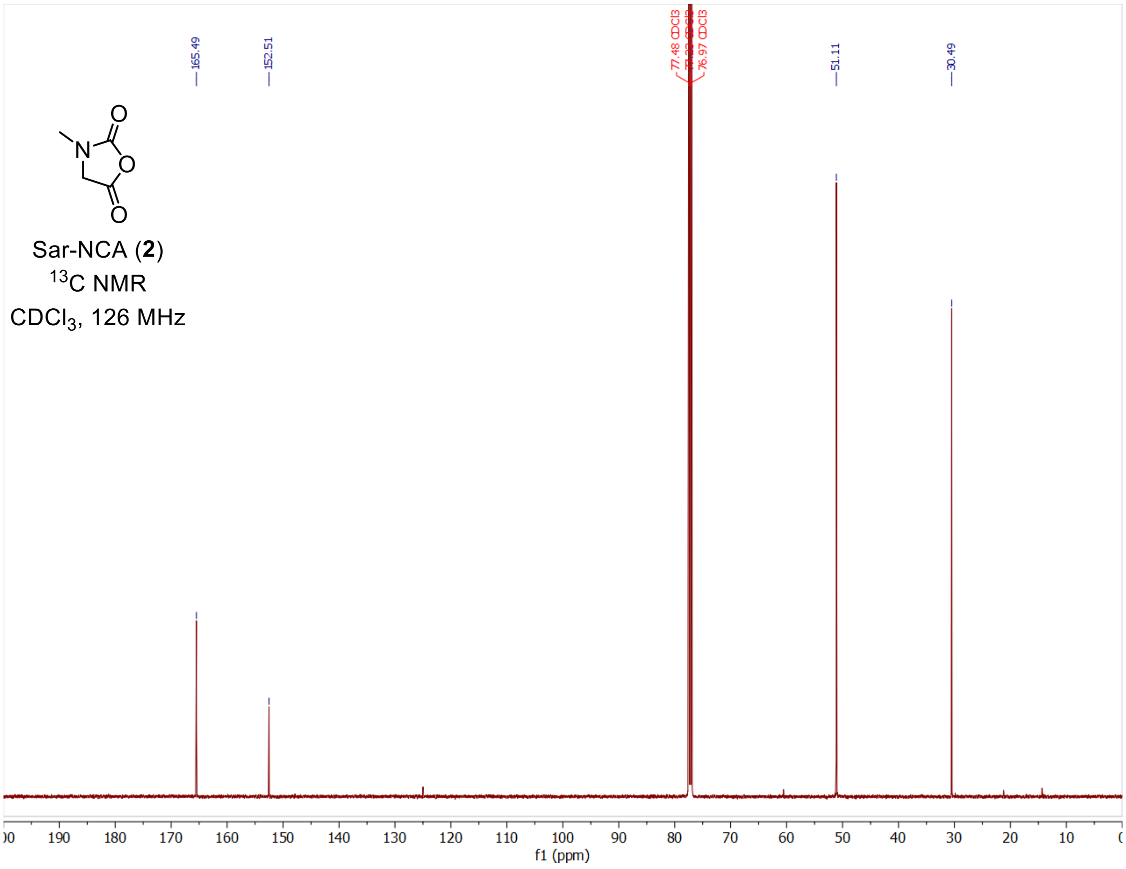

165.49
152.51
77.48 CDCl3
76.97 CDCl3
51.11
30.49
Sar-NCA (2)
13C NMR
CDCl3, 126 MHz
f1 (ppm)

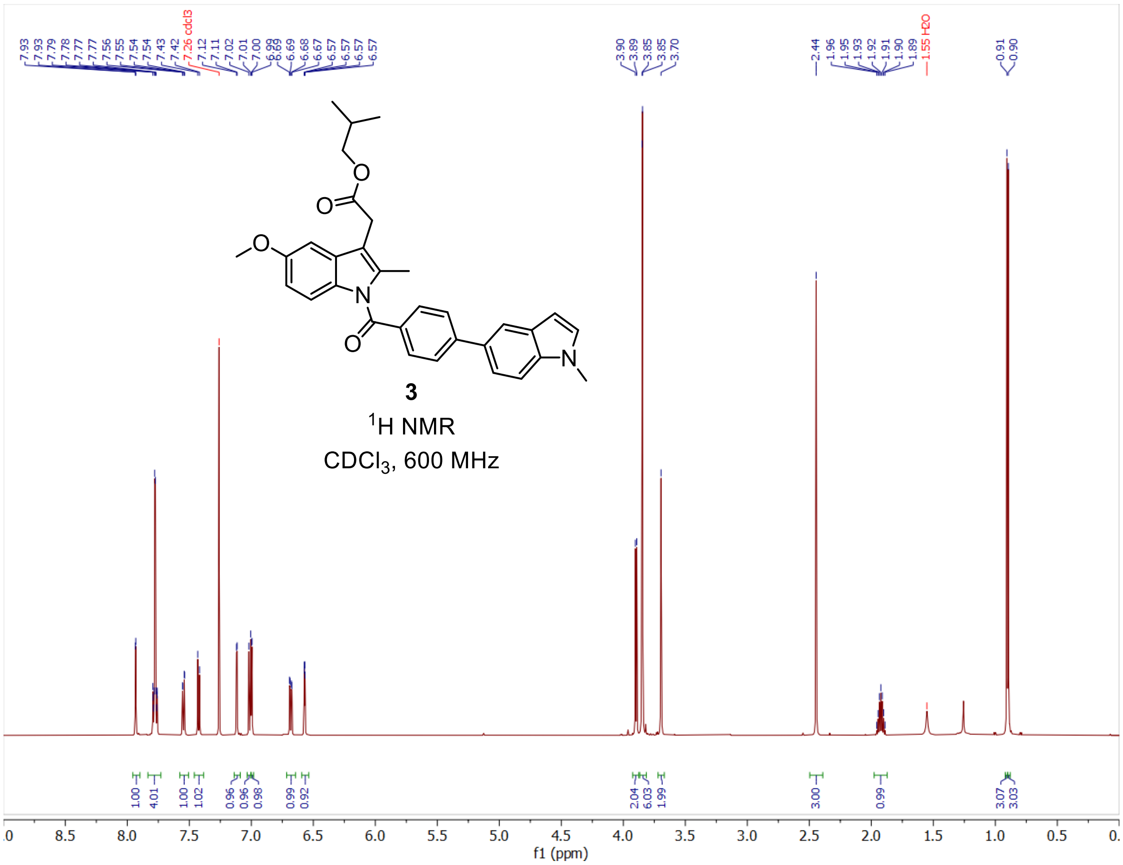
3
^{1}H NMR
$CDCl_3$, 600 MHz
f1 (ppm)

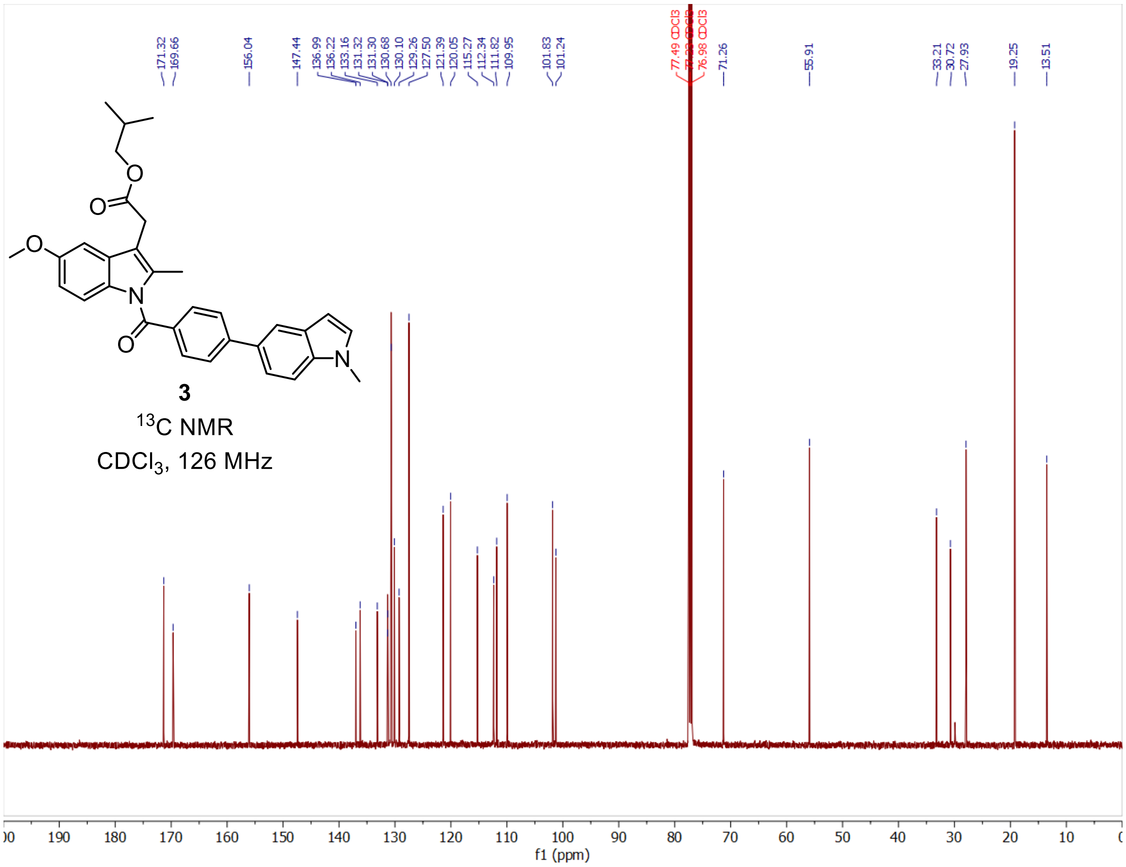
3
^{13}C NMR
$CDCl_3$, 126 MHz
f1 (ppm)

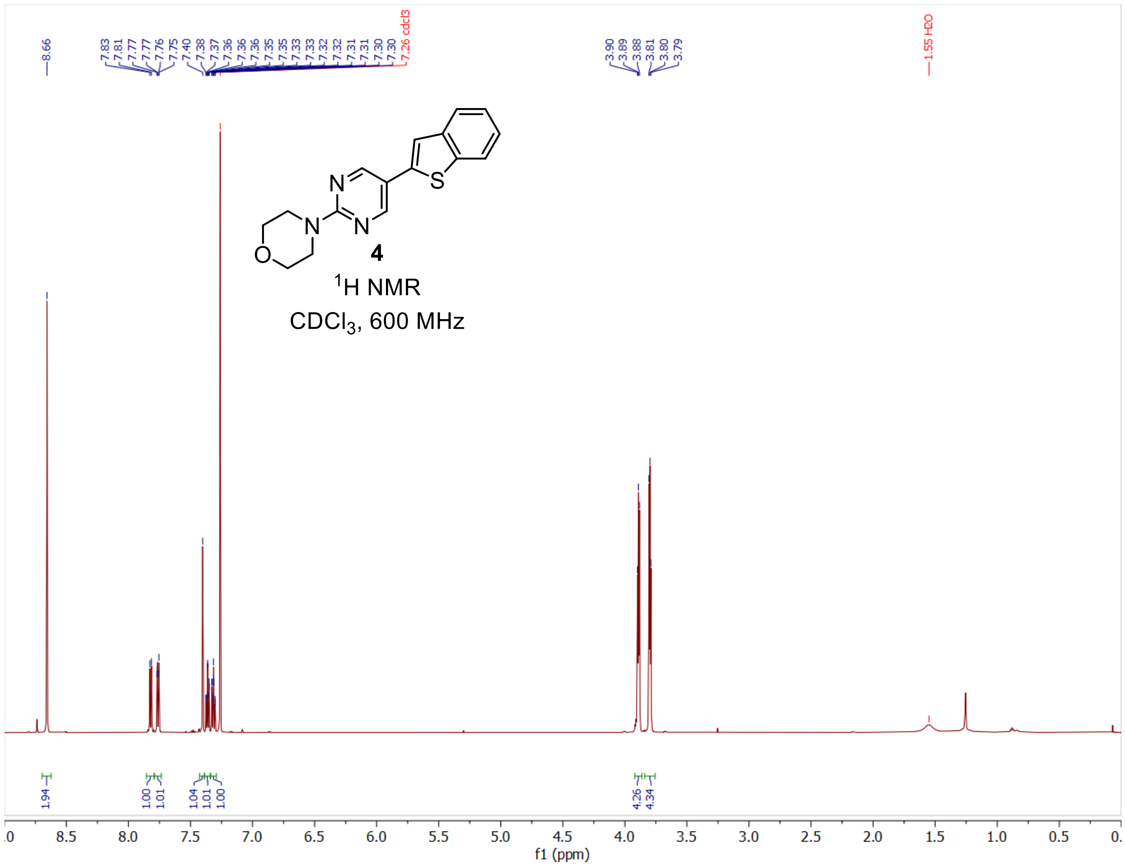
8.66
7.83
7.81
7.77
7.77
7.76
7.75
7.40
7.38
7.37
7.36
7.36
7.36
7.35
7.35
7.33
7.33
7.32
7.31
7.31
7.30
7.30
7.26 cdcl3
3.90
3.89
3.88
3.81
3.80
3.79
1.55 H2O
N
N
N
O
S
4
1H NMR
CDCl3, 600 MHz
1.94
1.00
1.01
1.04
1.01
1.00
4.26
4.34
f1 (ppm)

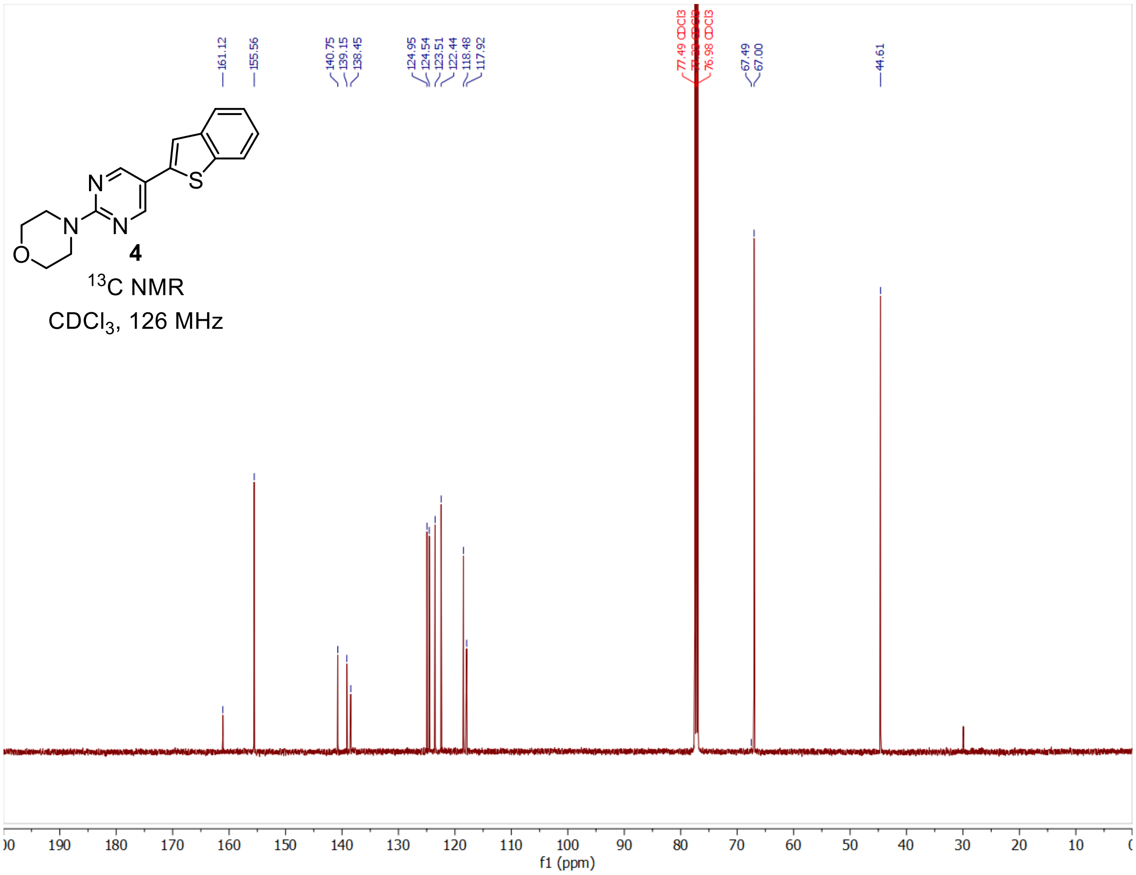
161.12
155.56
140.75
139.15
138.45
124.95
124.54
123.51
122.44
118.48
117.92
77.49 CDCl3
76.98 CDCl3
67.49
67.00
44.61
N
N
N
O
S
4
13C NMR
CDCl3, 126 MHz
f1 (ppm)

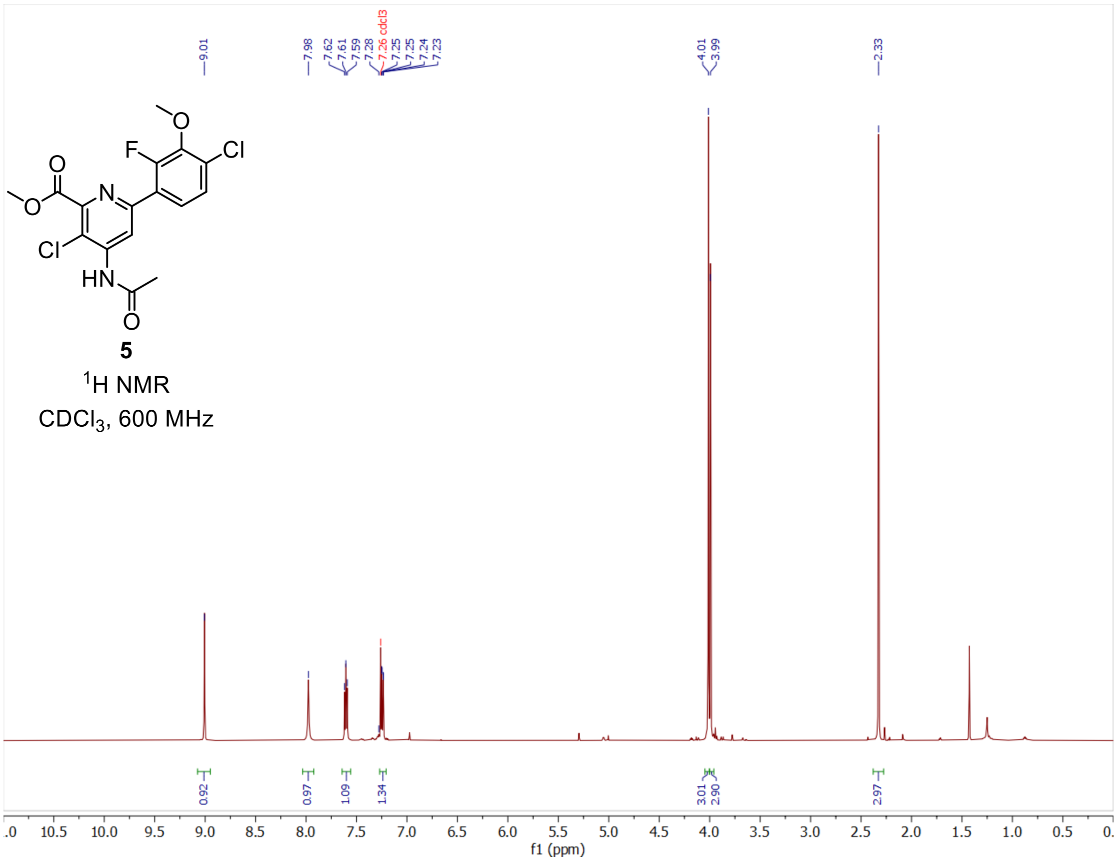
9.01
7.98
7.62
7.61
7.59
7.28
7.26 cdcl3
7.25
7.25
7.25
7.24
7.23
4.01
3.99
2.33
O
F
Cl
O
N
O
Cl
HN
O
5
1H NMR
CDCl3, 600 MHz
0.92
0.97
1.09
1.34
3.01
2.90
2.97
f1 (ppm)

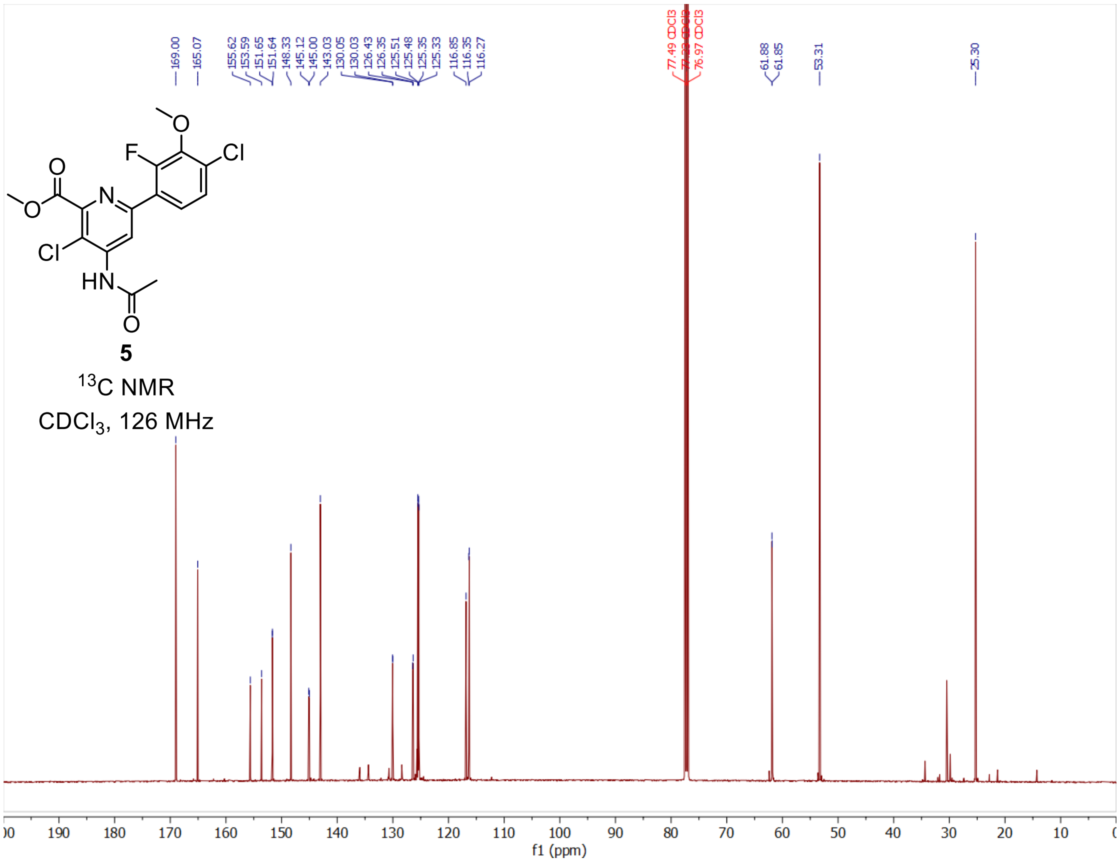
169.00
165.07
155.62
153.59
151.65
151.64
148.33
145.12
145.00
143.03
130.05
130.03
126.43
126.35
125.51
125.48
125.35
125.33
116.85
116.35
116.27
77.49 CDCl3
76.97 CDCl3
61.88
61.85
53.31
25.30
O
F
Cl
O
N
O
Cl
HN
O
5
13C NMR
CDCl3, 126 MHz
f1 (ppm)

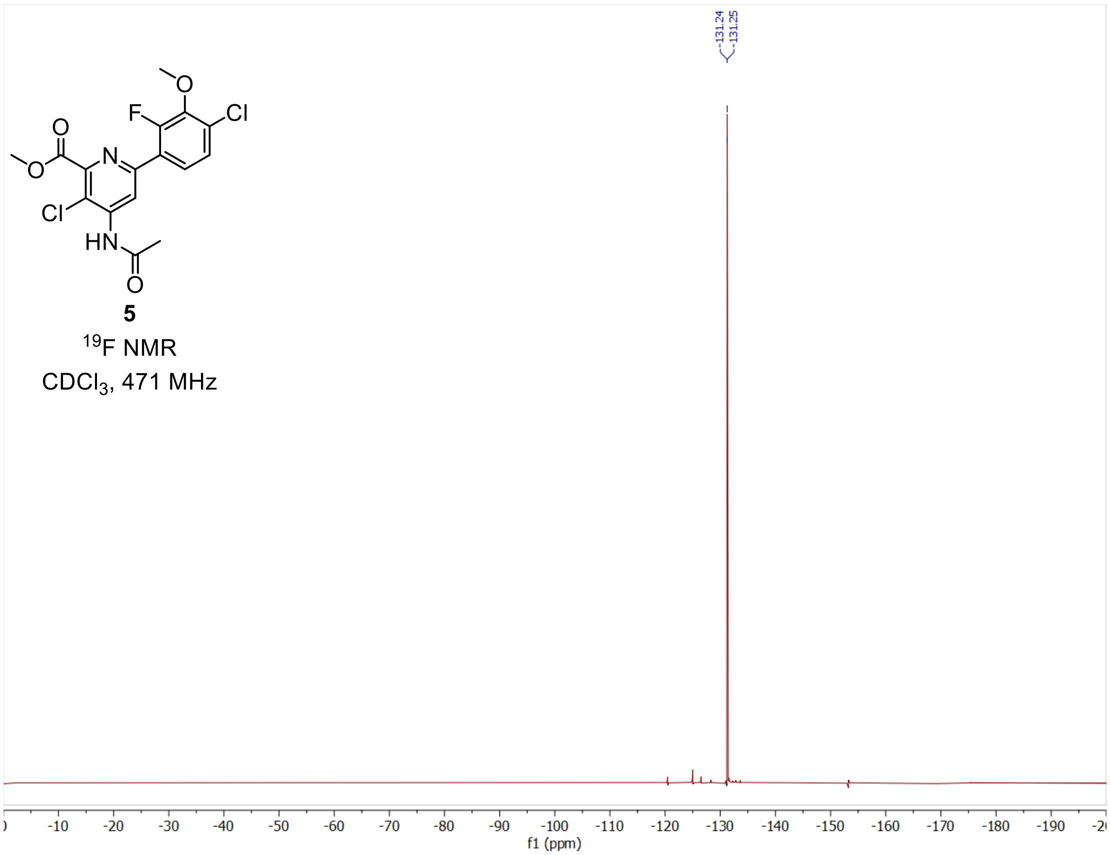
-131.24
-131.25
5
19F NMR
CDCl3, 471 MHz
-10
-20
-30
-40
-50
-60
-70
-80
-90
-100
-110
-120
-130
-140
-150
-160
-170
-180
-190
f1 (ppm)

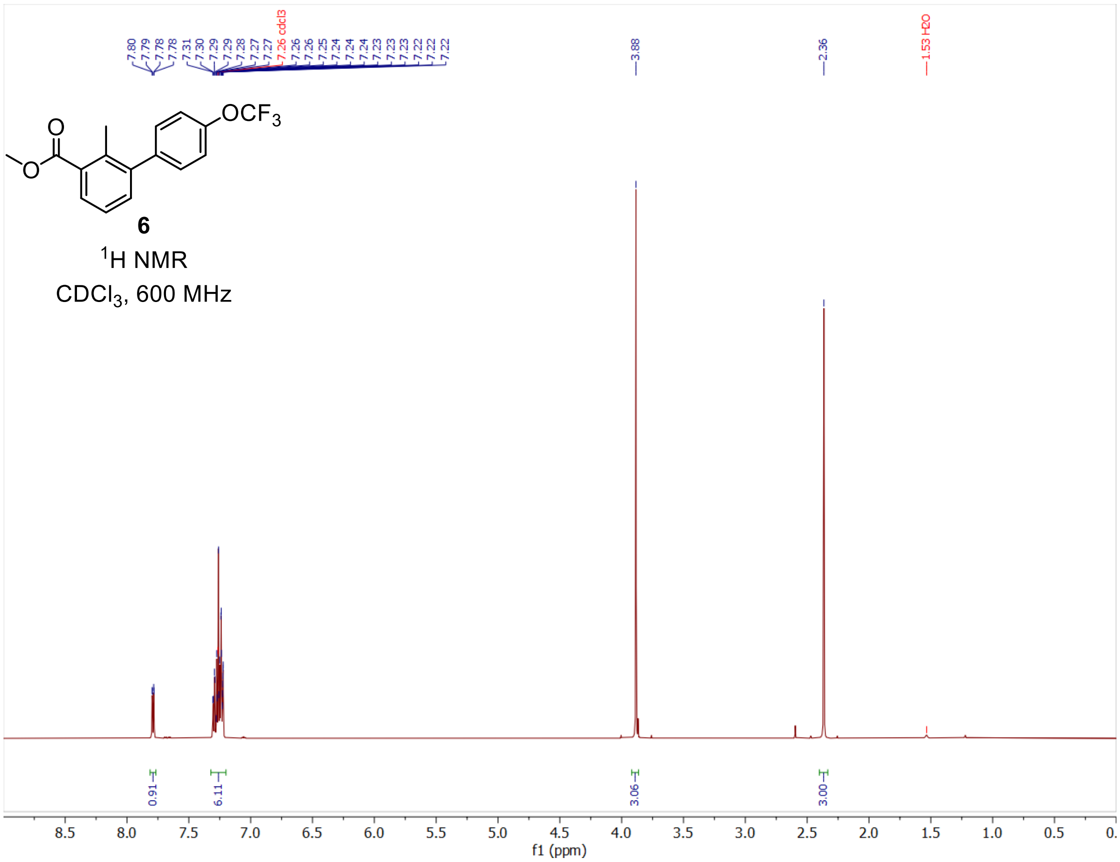

OCF3
6
1H NMR
CDCl3, 600 MHz
7.80 7.79 7.78 7.78 7.31 7.30 7.29 7.29 7.28 7.27 7.27 7.26 cdcl3 7.26 7.26 7.25 7.24 7.24 7.24 7.23 7.23 7.23 7.22 7.22 7.22 7.22
3.88
2.36
1.53 H2O
0.91
6.11
3.06
3.00
f1 (ppm)

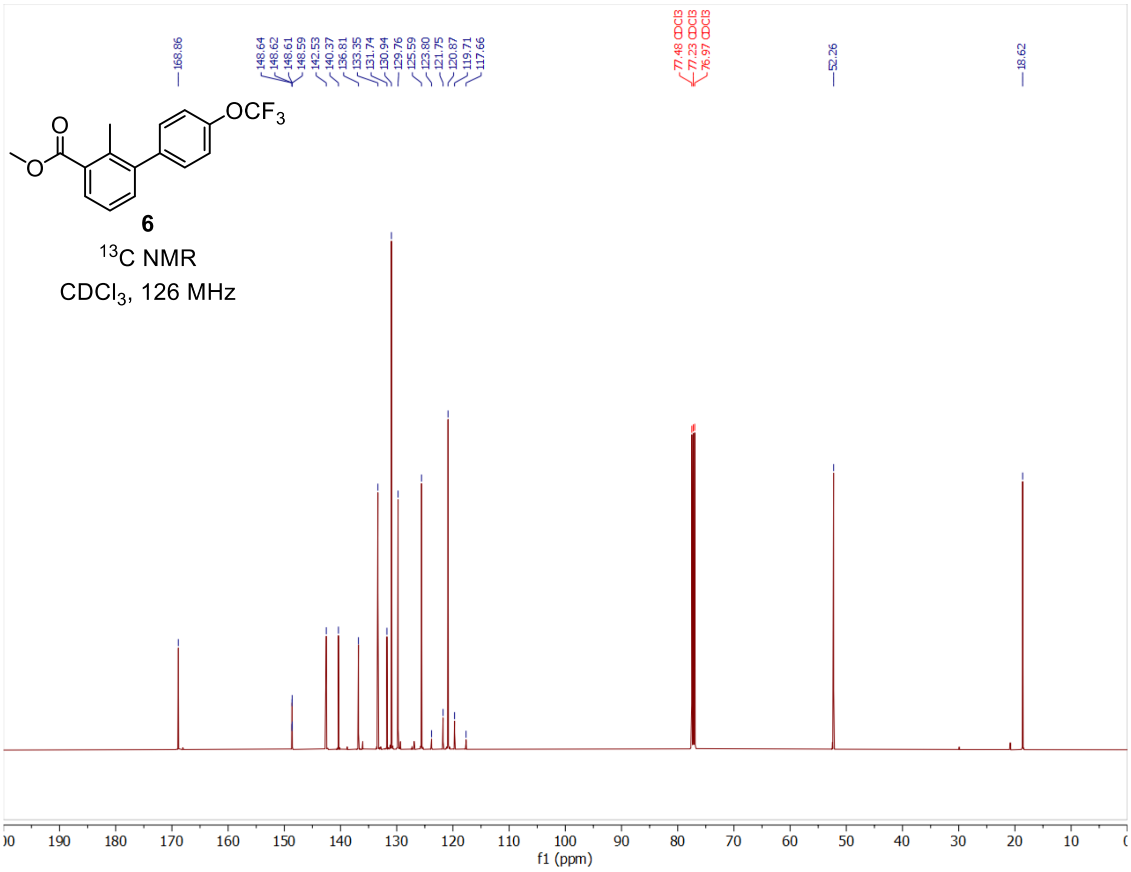

OCF3
6
13C NMR
CDCl3, 126 MHz
168.86
148.64 148.62 148.61 148.59 142.53 140.37 136.81 133.35 131.74 130.94 129.76 125.59 123.80 121.75 120.87 119.71 117.66
77.48 CDCl3 77.23 CDCl3 76.97 CDCl3
52.26
18.62
f1 (ppm)

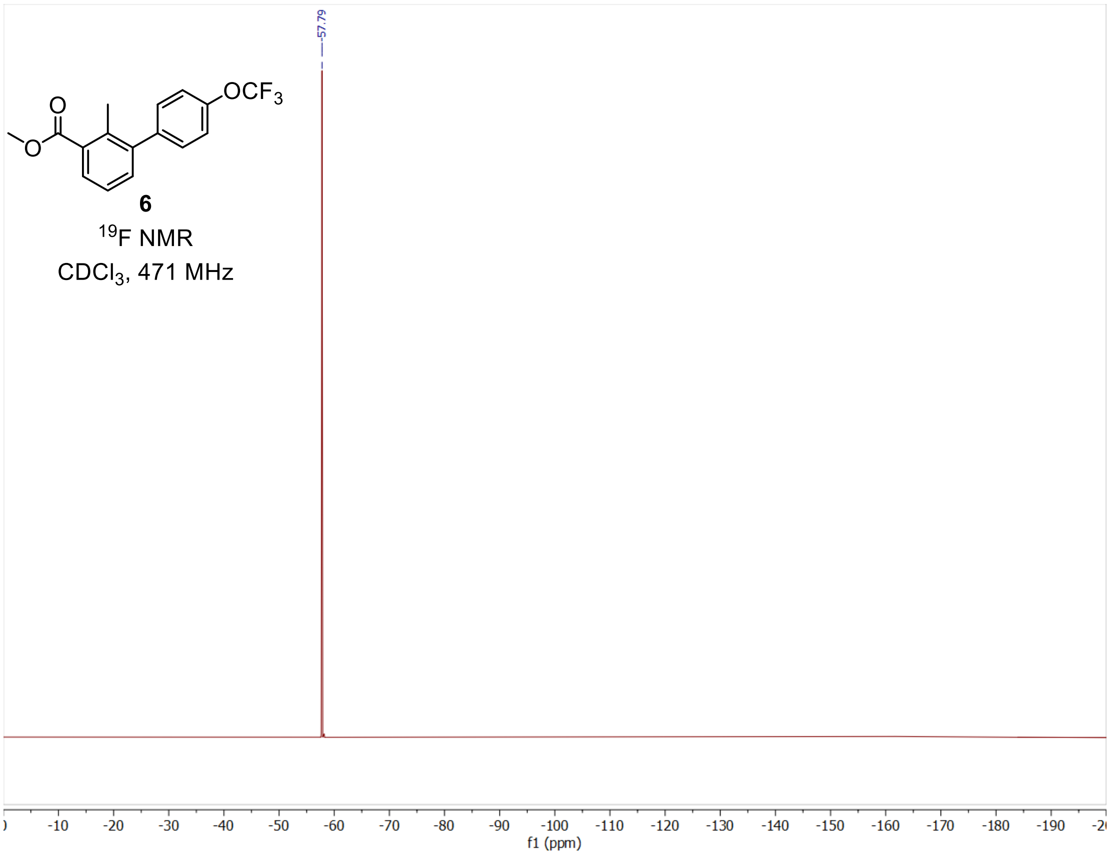
-57.79
OCF3
O
O
6
19F NMR
CDCl3, 471 MHz
-10
-20
-30
-40
-50
-60
-70
-80
-90
-100
-110
-120
-130
-140
-150
-160
-170
-180
-190
f1 (ppm)

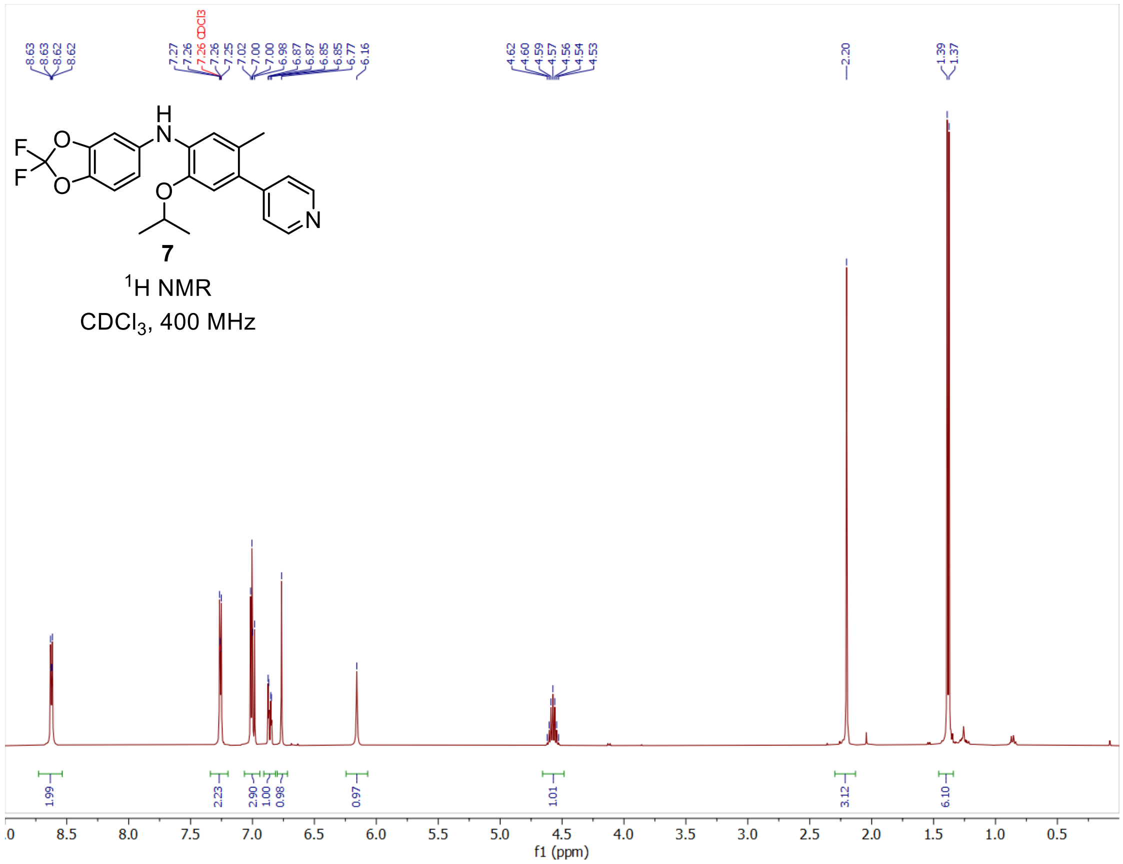
8.63 8.63 8.62 8.62 7.27 7.26 7.26 CDCl3 7.26 7.25 7.02 7.00 7.00 6.98 6.87 6.87 6.85 6.85 6.77 6.16 4.62 4.60 4.59 4.57 4.56 4.54 4.53 2.20 1.39 1.37
7
1H NMR
CDCl3, 400 MHz
1.99 2.23 2.90 1.00 0.98 0.97 1.01 3.12 6.10
f1 (ppm)

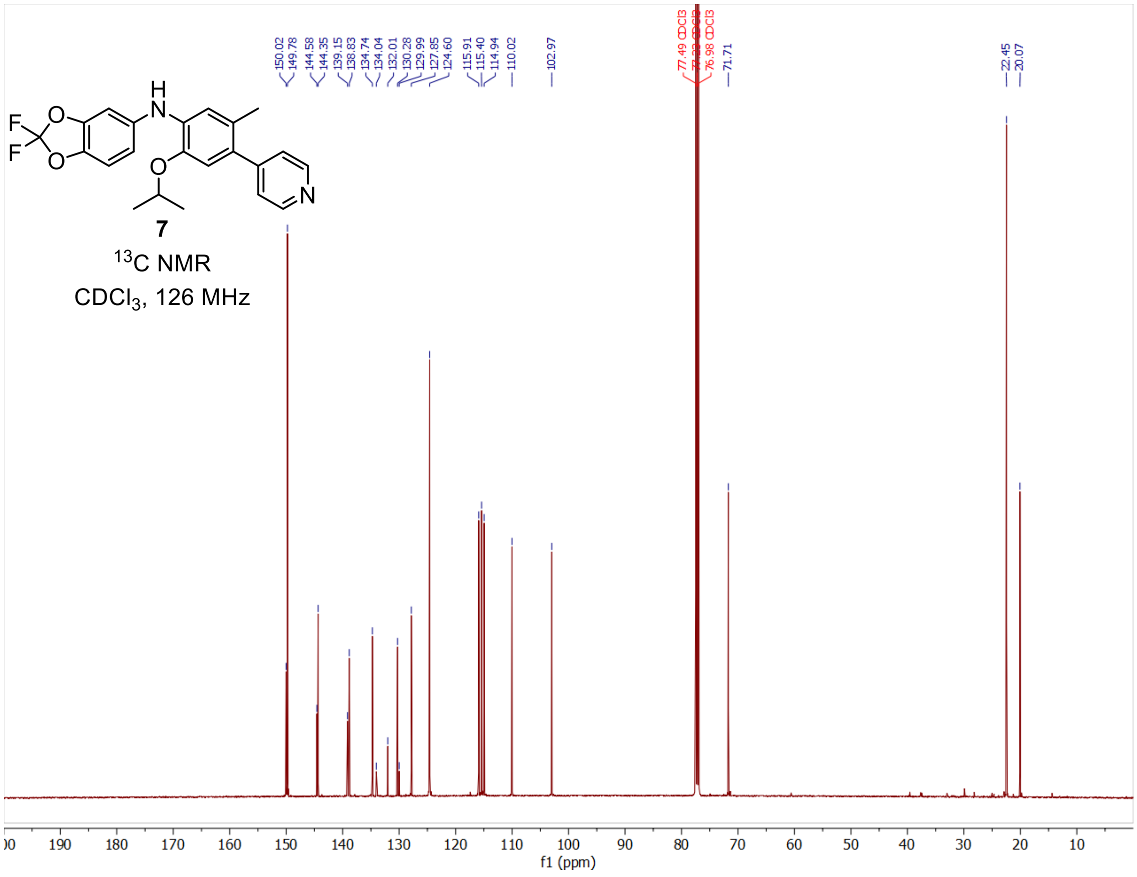
150.02 149.78 144.58 144.35 139.15 138.83 134.74 134.04 132.01 130.28 129.99 127.85 124.60 115.91 115.40 114.94 110.02 102.97 77.49 CDCl3 76.98 CDCl3 71.71 22.45 20.07
7
13C NMR
CDCl3, 126 MHz
f1 (ppm)

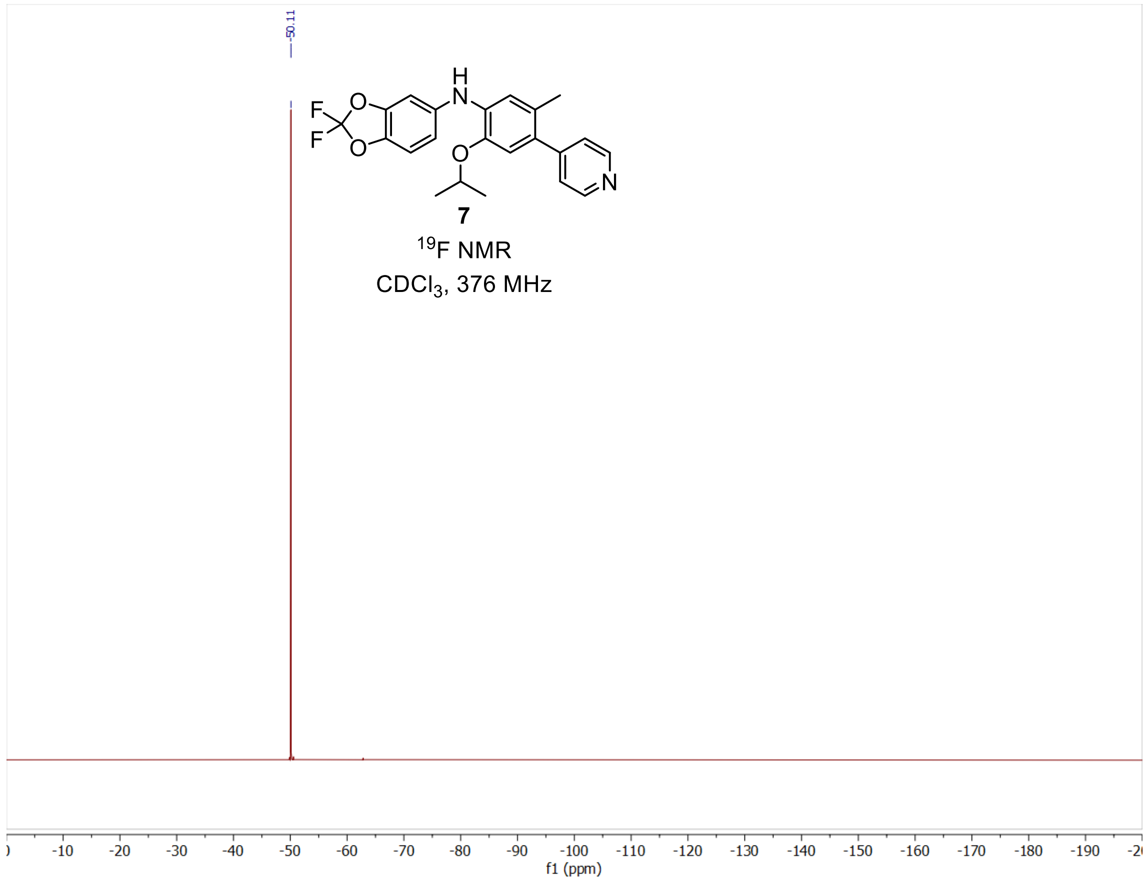
-50.11
7
19F NMR
CDCl3, 376 MHz
0
-10
-20
-30
-40
-50
-60
-70
-80
-90
-100
-110
-120
-130
-140
-150
-160
-170
-180
-190
f1 (ppm)

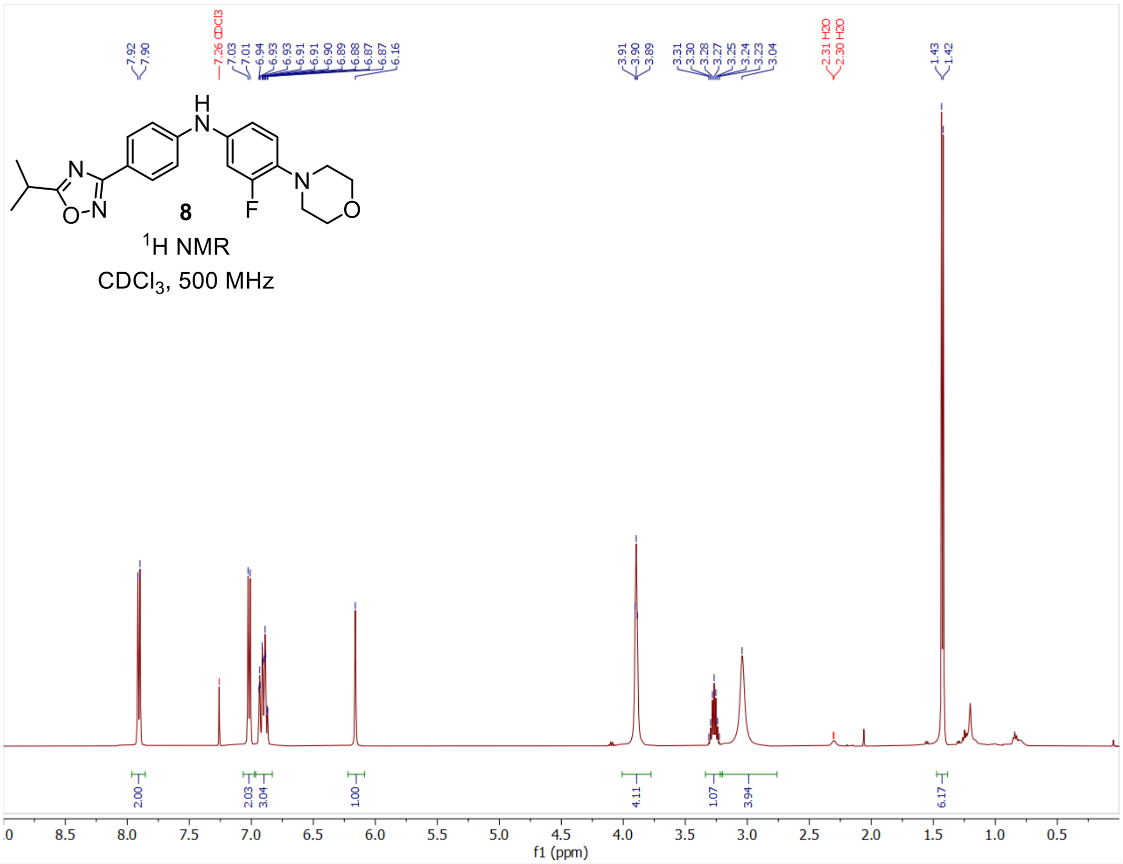
8
^{1}H NMR
$CDCl_3$, 500 MHz
f1 (ppm)

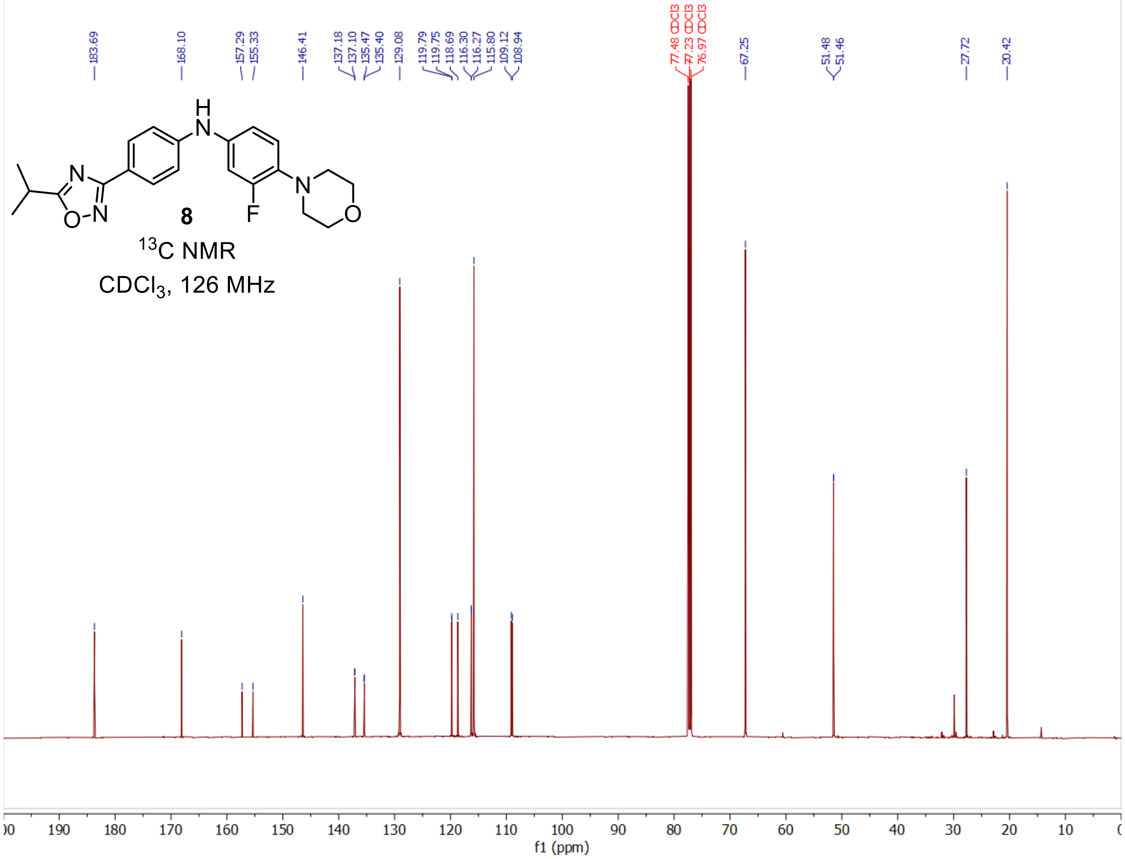
8
^{13}C NMR
$CDCl_3$, 126 MHz
f1 (ppm)

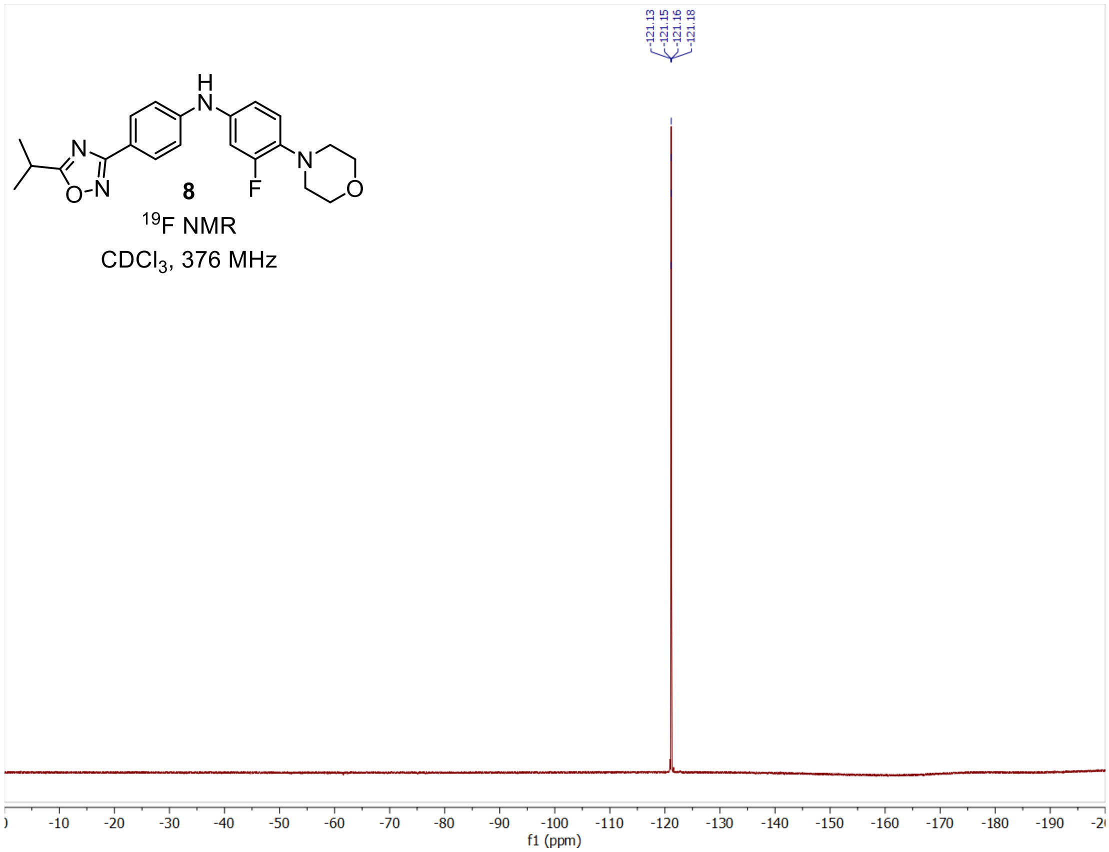

-121.13
-121.15
-121.16
-121.18
H
N
N
O-N
F
N
O
8
19F NMR
CDCl3, 376 MHz
-10 -20 -30 -40 -50 -60 -70 -80 -90 -100 -110 -120 -130 -140 -150 -160 -170 -180 -190
f1 (ppm)

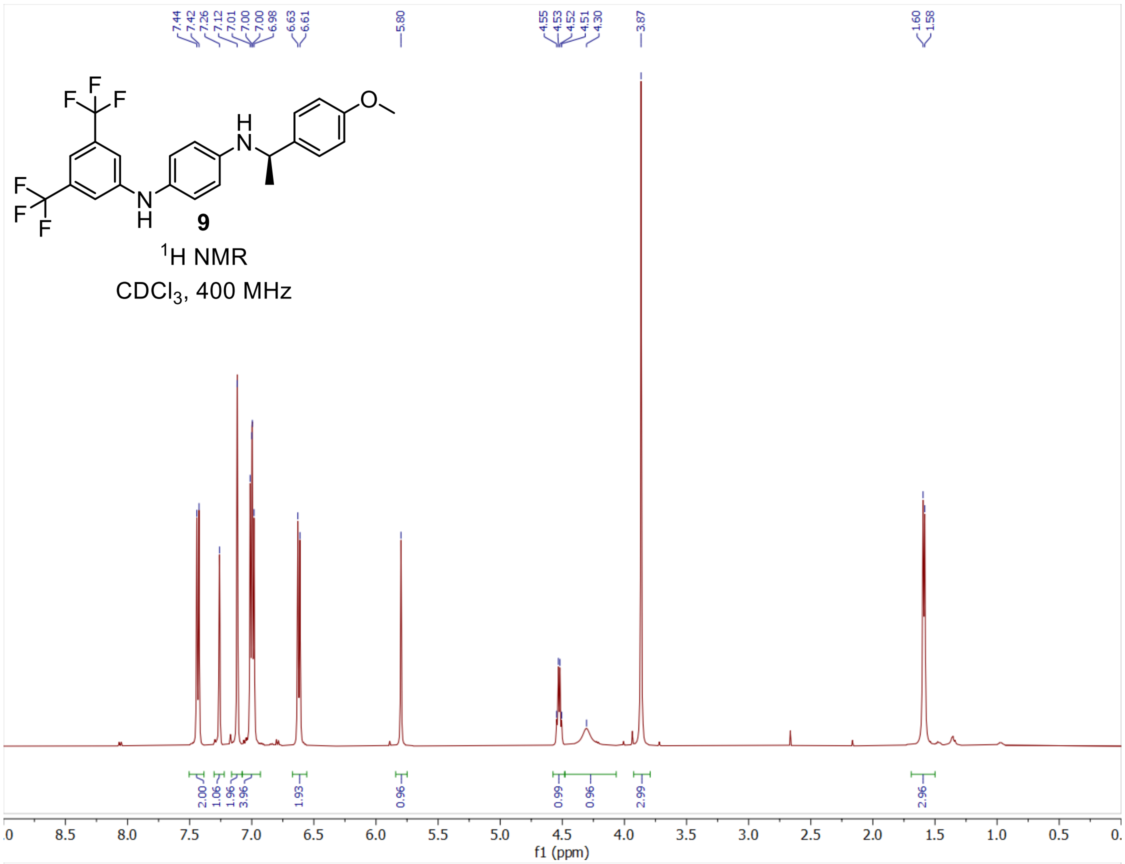
9
^{1}H NMR
CDCl$_3$, 400 MHz
7.44 7.42 7.26 7.12 7.01 7.00 7.00 6.98 6.63 6.61 5.80 4.55 4.53 4.52 4.51 4.30 3.87 1.60 1.58
2.00 1.06 1.96 3.96 1.93 0.96 0.99 0.96 2.99 2.96
f1 (ppm)

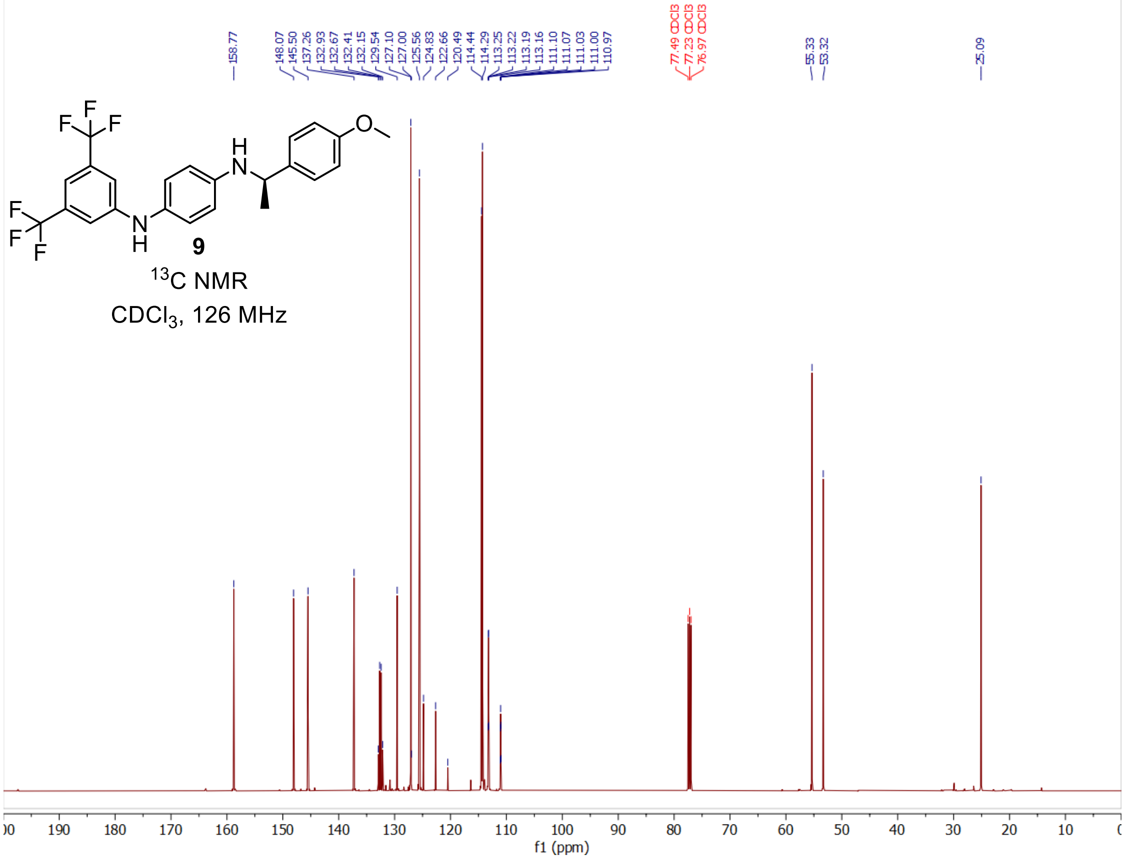
9
^{13}C NMR
CDCl$_3$, 126 MHz
158.77 148.07 145.50 137.26 132.93 132.67 132.41 132.15 129.54 127.10 127.00 125.56 124.83 122.66 120.49 114.44 114.29 113.25 113.22 113.19 113.16 111.10 111.07 111.03 111.00 110.97
77.49 CDCl3 77.23 CDCl3 76.97 CDCl3
55.33 53.32 25.09
f1 (ppm)

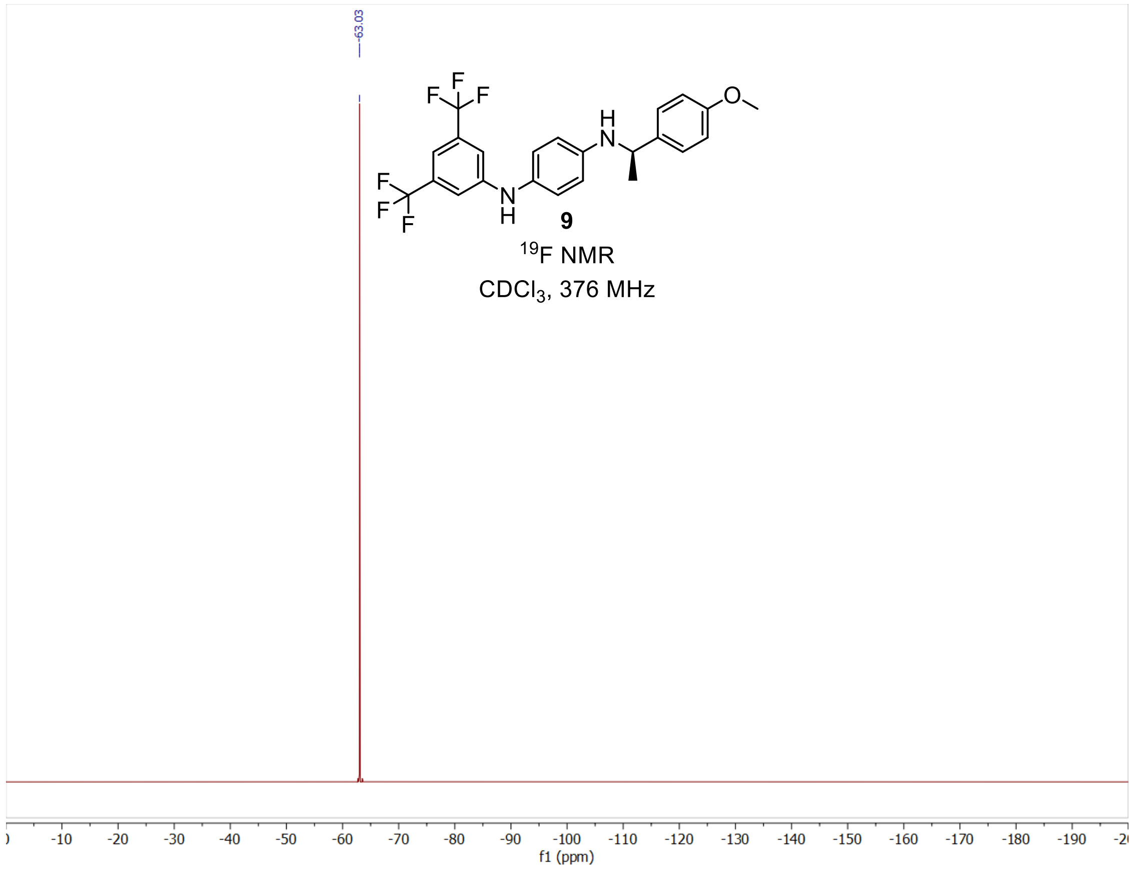

-63.03
F
N
H
9
19F NMR
CDCl3, 376 MHz
0
-10
-20
-30
-40
-50
-60
-70
-80
-90
-100
-110
-120
-130
-140
-150
-160
-170
-180
-190
f1 (ppm)

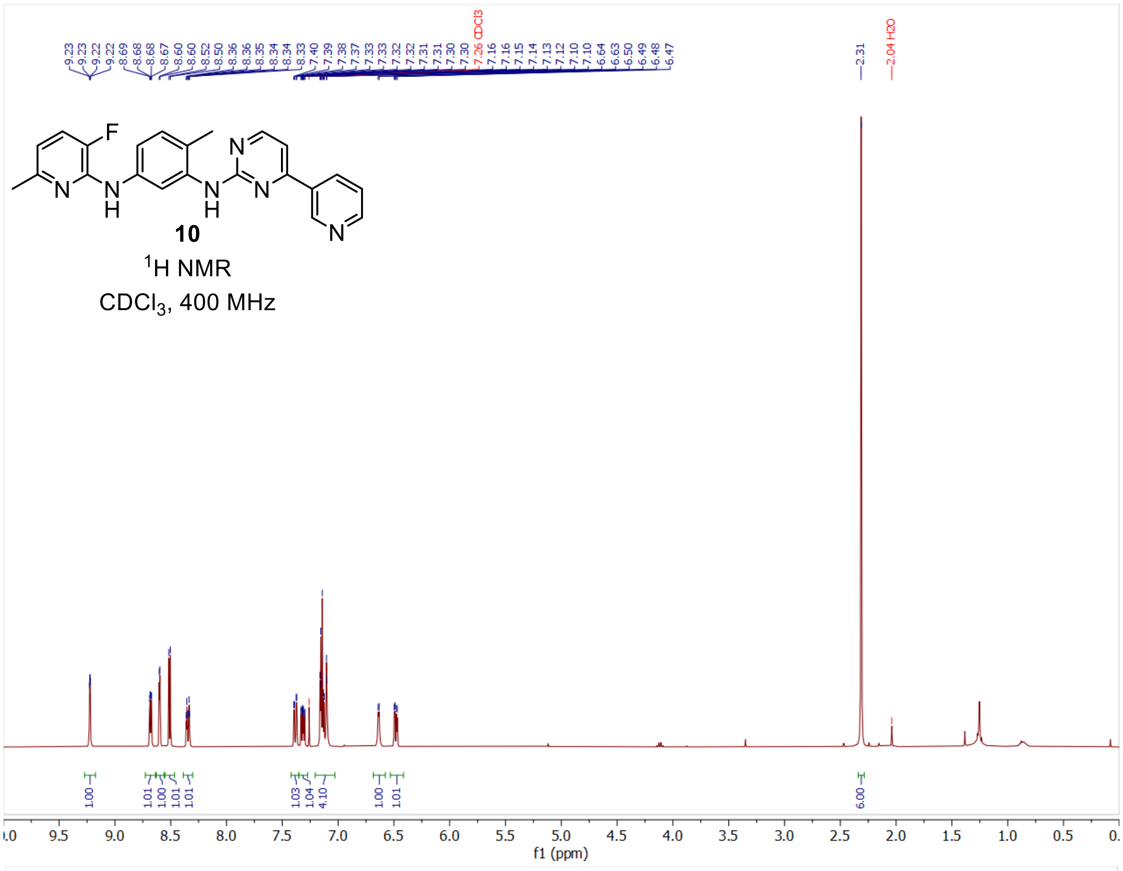
9.23 9.23 9.22 9.22 8.69 8.68 8.68 8.67 8.60 8.60 8.52 8.50 8.36 8.36 8.35 8.34 8.34 8.33 7.40 7.39 7.38 7.37 7.33 7.33 7.32 7.32 7.31 7.31 7.30 7.30 7.26 CDCl3 7.16 7.16 7.15 7.14 7.13 7.12 7.10 7.10 6.64 6.63 6.50 6.49 6.48 6.47
2.31
2.04 H2O
F
N
N
H
N
N
N
H
N
10
1H NMR
CDCl3, 400 MHz
1.00
1.01 1.00 1.01 1.01
1.03 1.04 4.10
1.00 1.01
6.00
f1 (ppm)

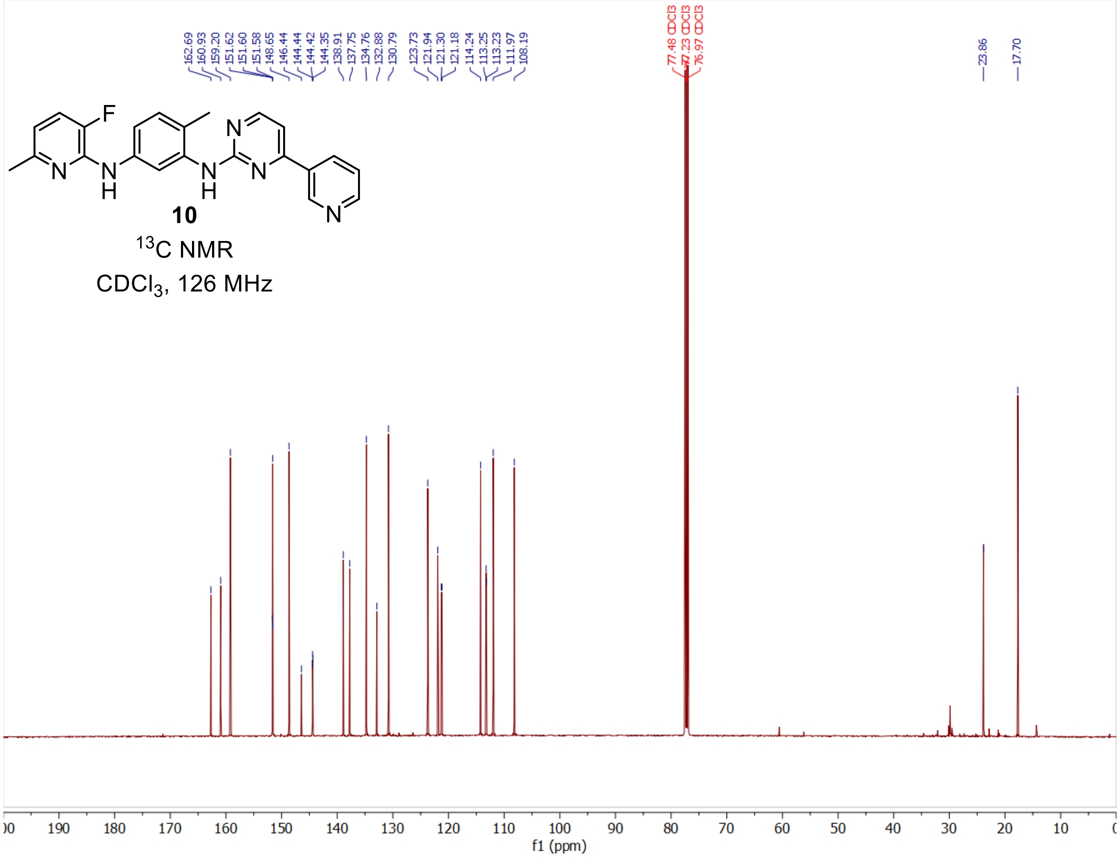
162.69 160.93 159.20 151.62 151.60 151.58 148.65 146.44 144.44 144.42 144.35 138.91 137.75 134.76 132.88 130.79
123.73 121.94 121.30 121.18 114.24 113.25 113.23 111.97 108.19
77.48 CDCl3 77.23 CDCl3 76.97 CDCl3
23.86
17.70
10
13C NMR
CDCl3, 126 MHz
f1 (ppm)

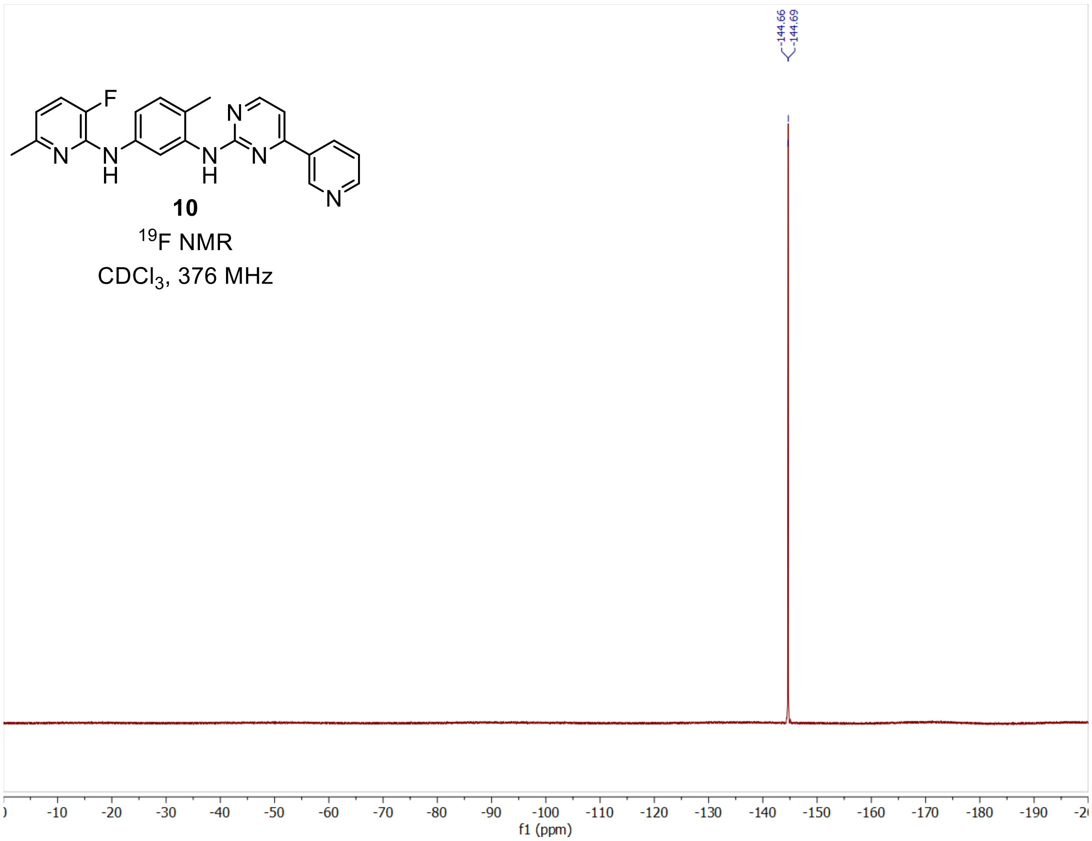

-144.66
-144.69
F
N
N
H
N
N
N
H
N
10
19F NMR
CDCl3, 376 MHz
-10
-20
-30
-40
-50
-60
-70
-80
-90
-100
-110
-120
-130
-140
-150
-160
-170
-180
-190
f1 (ppm)

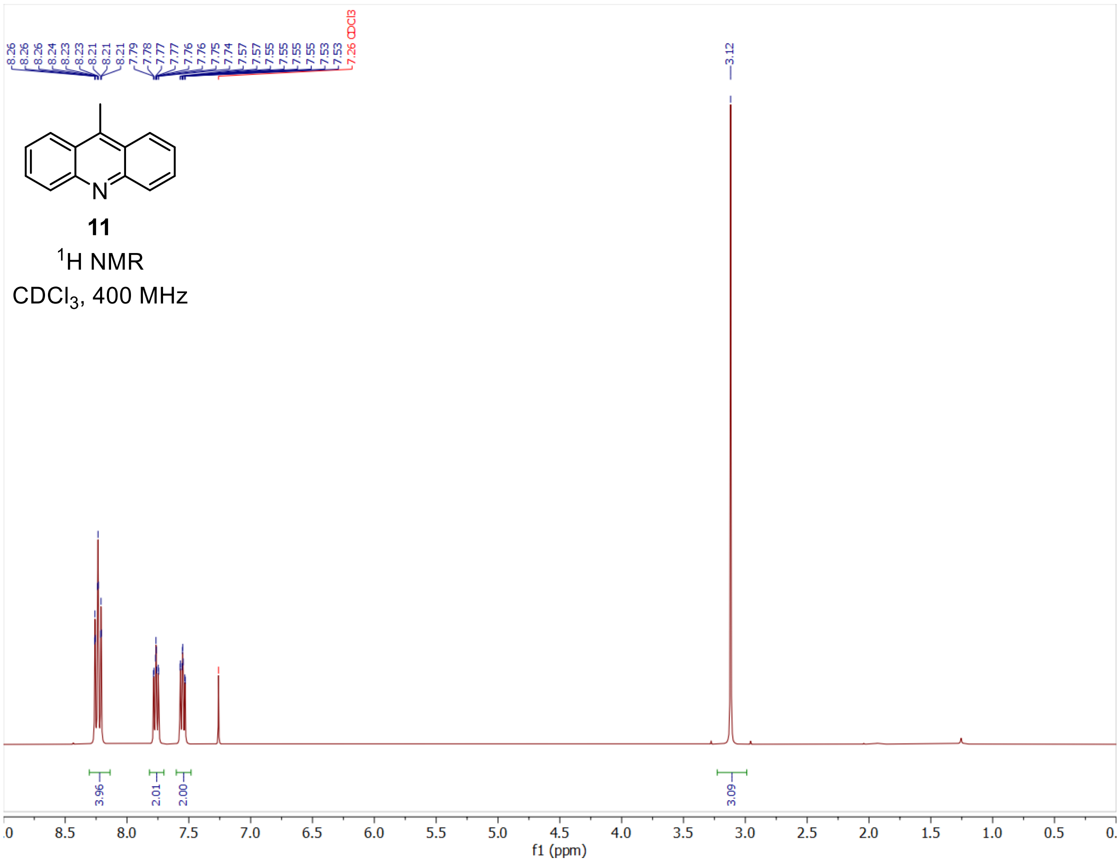

8.26
8.26
8.26
8.24
8.23
8.23
8.21
8.21
8.21
7.79
7.78
7.77
7.77
7.76
7.76
7.75
7.74
7.57
7.57
7.55
7.55
7.55
7.55
7.55
7.53
7.53
7.26 CDCl3
3.12
11
1H NMR
CDCl3, 400 MHz
3.96
2.01
2.00
3.09
f1 (ppm)

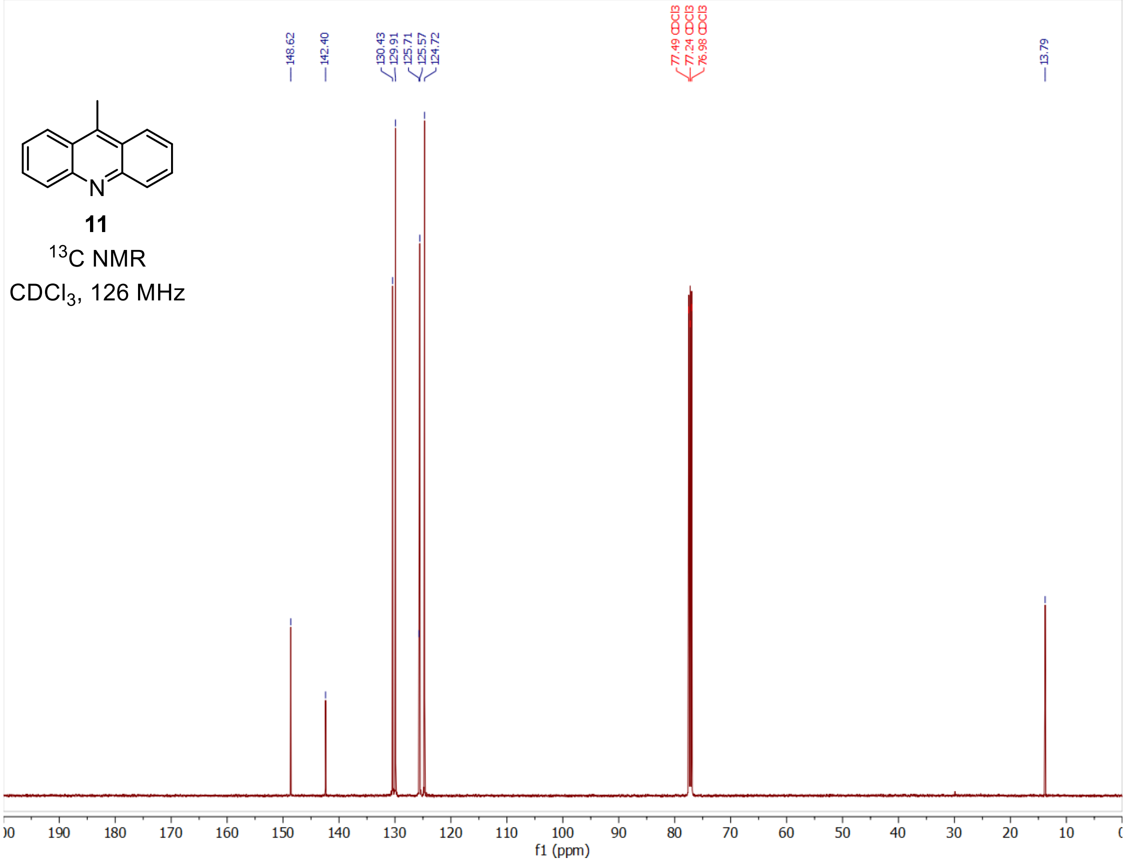

148.62
142.40
130.43
129.91
125.71
125.57
124.72
77.49 CDCl3
77.24 CDCl3
76.98 CDCl3
13.79
11
13C NMR
CDCl3, 126 MHz
f1 (ppm)

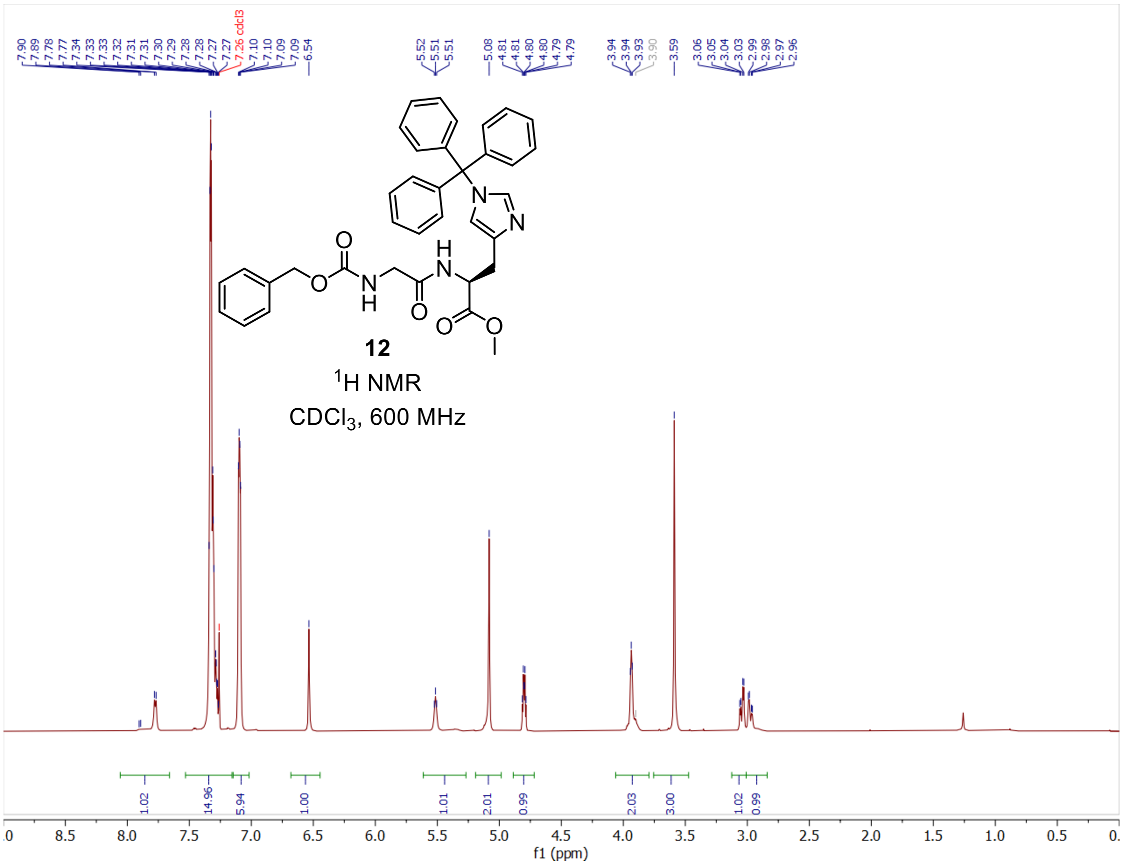
12
1H NMR
CDCl3, 600 MHz
7.90 7.89 7.78 7.77 7.34 7.33 7.33 7.32 7.31 7.31 7.30 7.29 7.28 7.28 7.27 7.27 7.26 cdcl3 7.10 7.10 7.09 7.09 6.54
5.52 5.51 5.51 5.08 4.81 4.81 4.80 4.80 4.79 4.79
3.94 3.94 3.93 3.90 3.59 3.06 3.05 3.04 3.03 2.99 2.98 2.97 2.96
1.02 14.96 5.94 1.00 1.01 2.01 0.99 2.03 3.00 1.02 0.99
f1 (ppm)

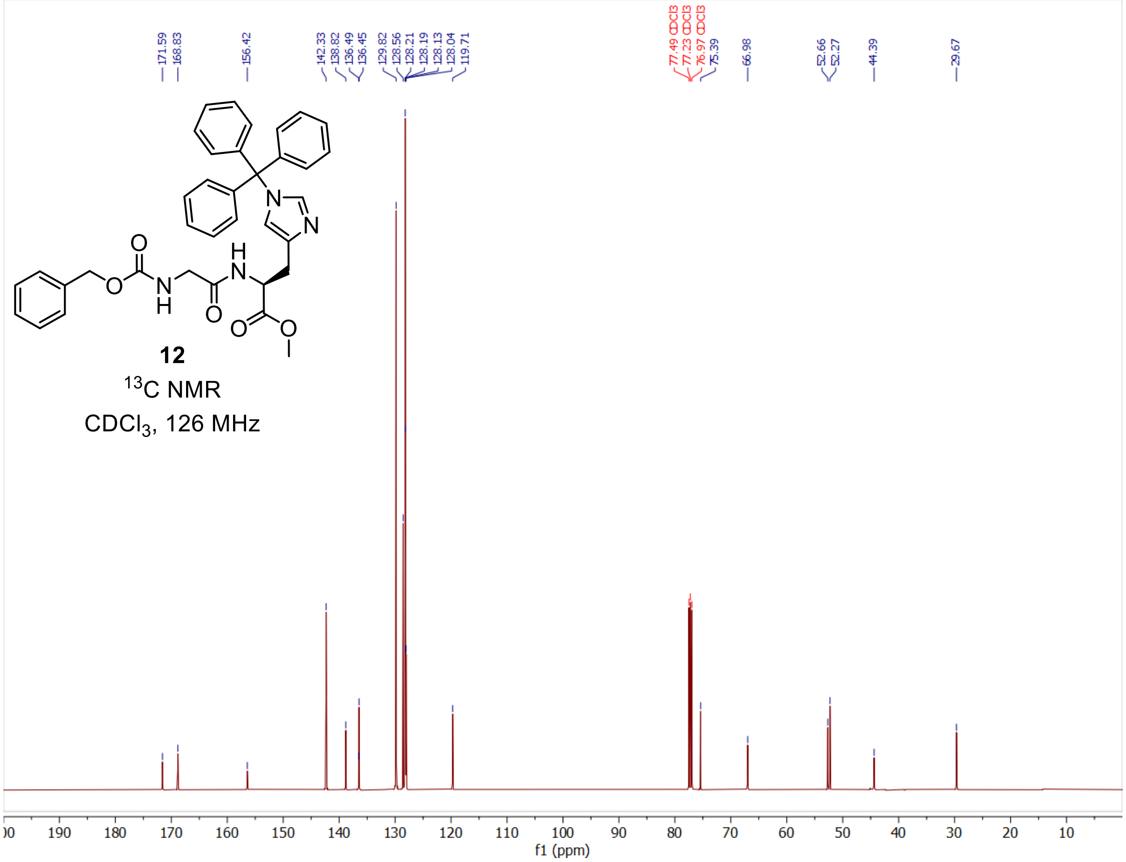
12
13C NMR
CDCl3, 126 MHz
171.59 168.83 156.42 142.33 138.82 136.49 136.45 129.82 128.56 128.21 128.19 128.13 128.04 119.71
77.49 CDCl3 77.23 CDCl3 76.97 CDCl3 75.39 66.98 52.66 52.27 44.39 29.67
f1 (ppm)

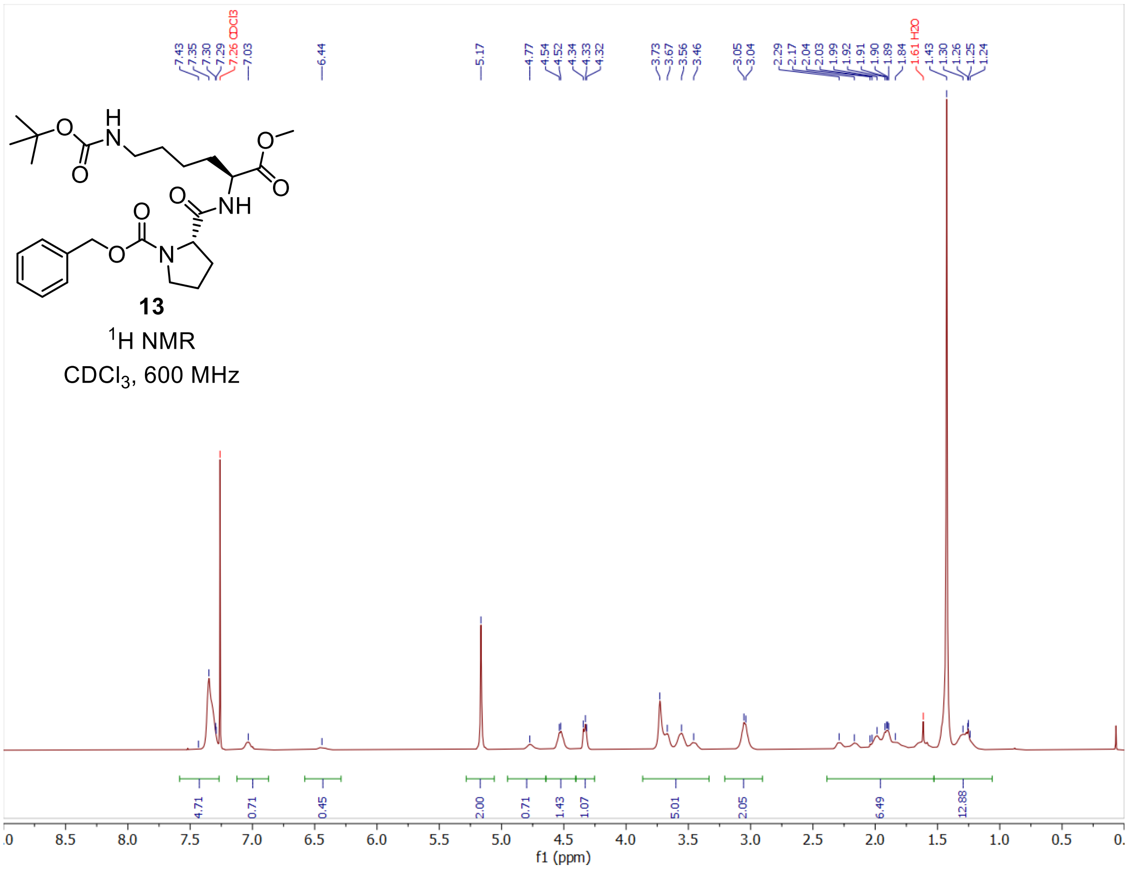
13
^{1}H NMR
CDCl3, 600 MHz
7.43
7.35
7.30
7.29
7.26 CDCl3
7.03
6.44
5.17
4.77
4.54
4.52
4.34
4.33
4.32
3.73
3.67
3.56
3.46
3.05
3.04
2.29
2.17
2.04
2.03
1.99
1.92
1.91
1.90
1.89
1.84
1.61 H2O
1.43
1.30
1.26
1.25
1.24
4.71
0.71
0.45
2.00
0.71
1.43
1.07
5.01
2.05
6.49
12.88
f1 (ppm)

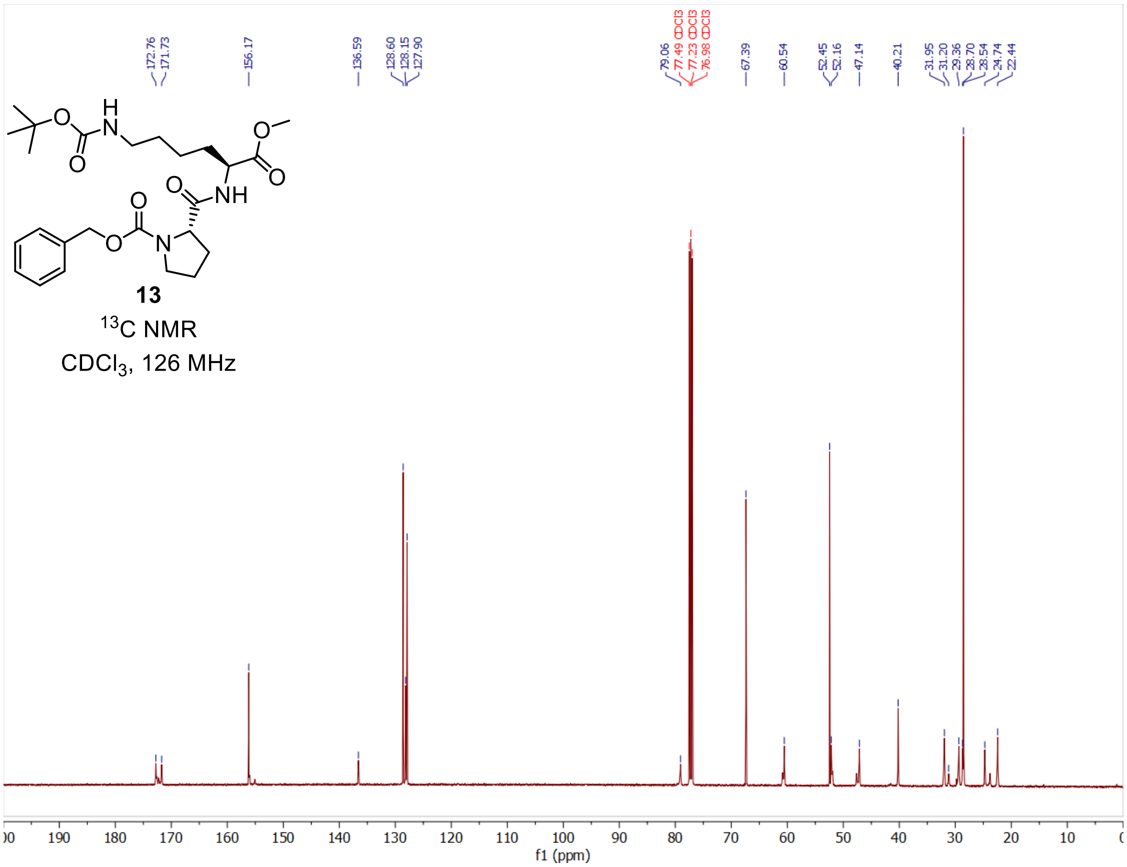
13
^{13}C NMR
CDCl3, 126 MHz
172.76
171.73
156.17
136.59
128.60
128.15
127.90
79.06
77.49 CDCl3
77.23 CDCl3
76.98 CDCl3
67.39
60.54
52.45
52.16
47.14
40.21
31.95
31.20
29.36
28.70
28.54
24.74
22.44
f1 (ppm)

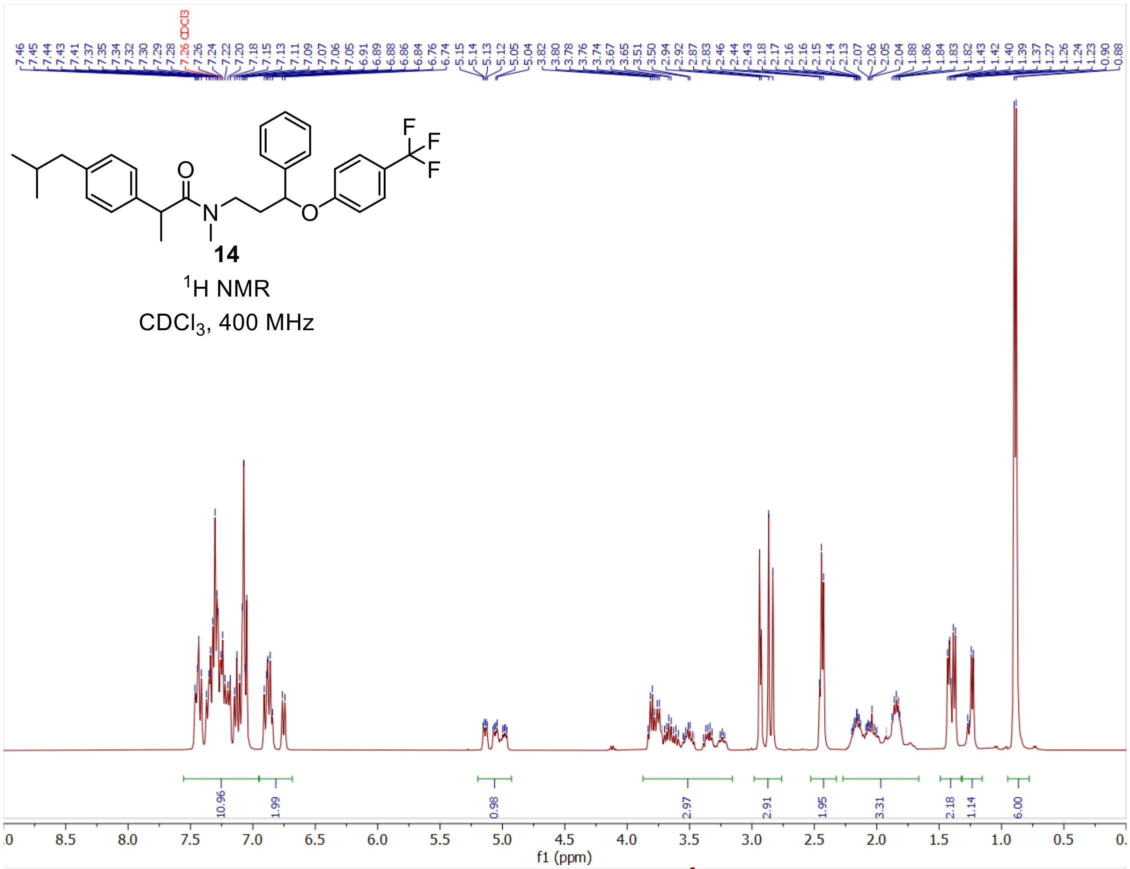
14
^{1}H NMR
$CDCl_3$, 400 MHz
f1 (ppm)

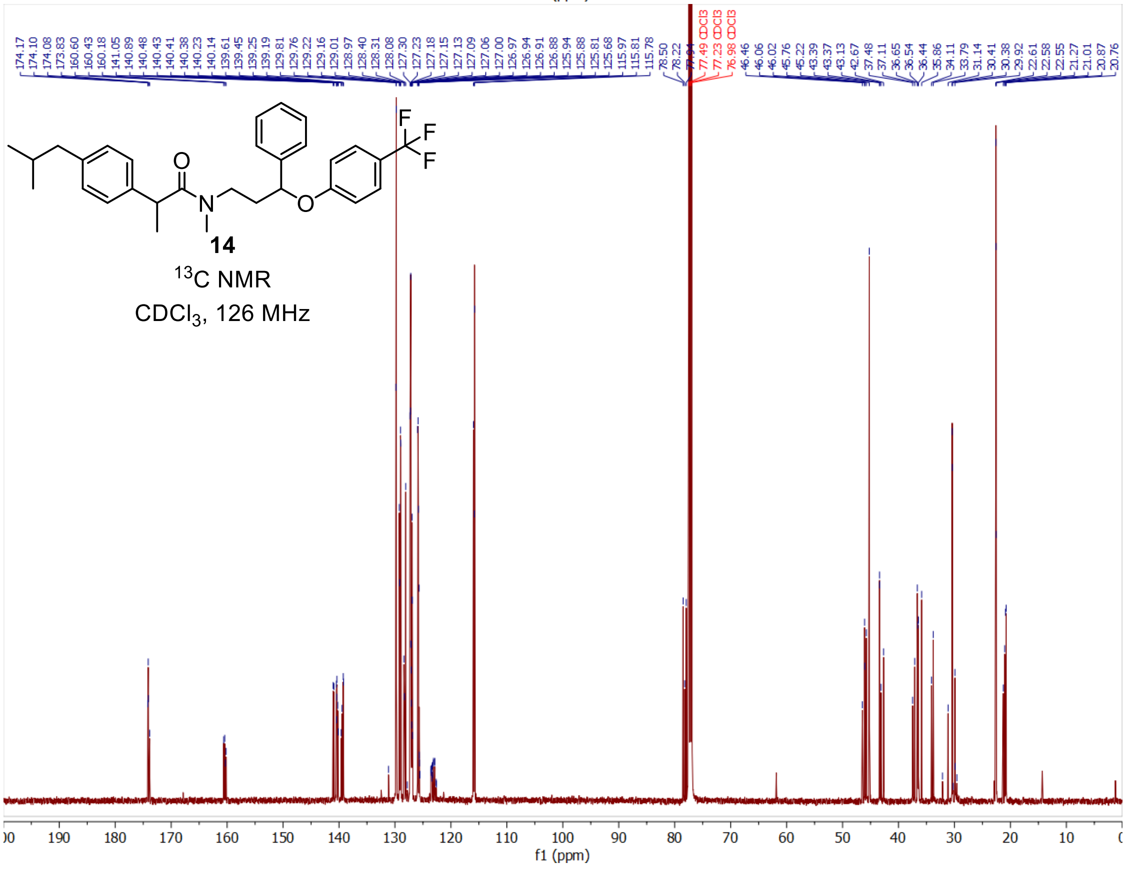
14
^{13}C NMR
$CDCl_3$, 126 MHz
f1 (ppm)

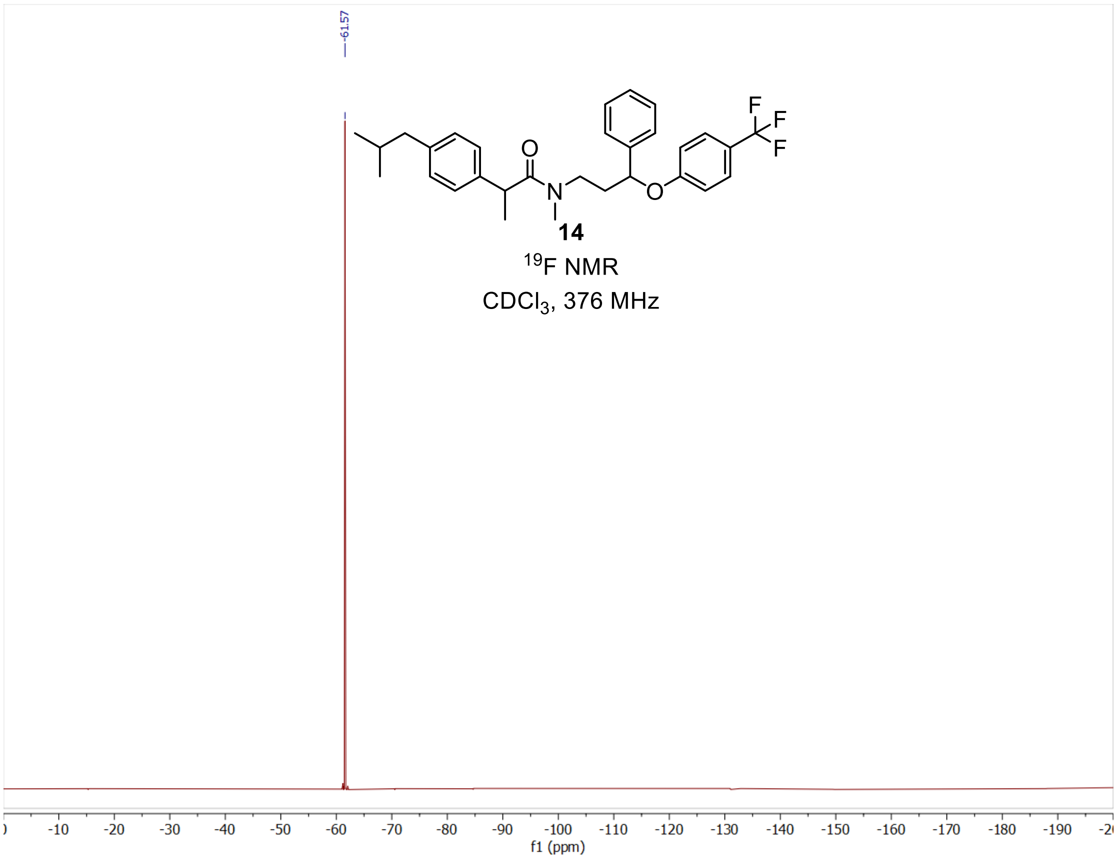
-61.57
14
19F NMR
CDCl3, 376 MHz
f1 (ppm)

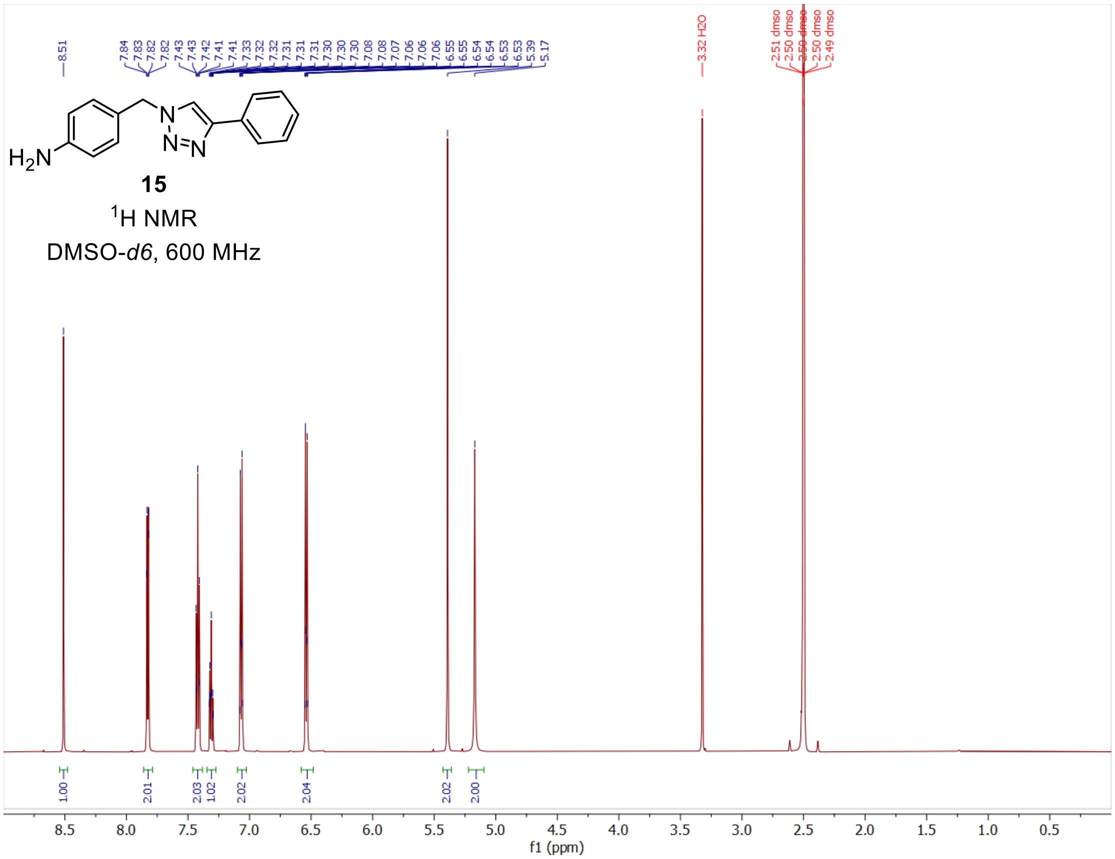
8.51
7.84
7.83
7.82
7.82
7.43
7.43
7.42
7.41
7.41
7.33
7.32
7.32
7.31
7.31
7.31
7.30
7.30
7.08
7.08
7.07
7.06
7.06
7.06
6.55
6.55
6.54
6.54
6.53
6.53
5.39
5.17
3.32 H2O
2.51 dmso
2.50 dmso
2.50 dmso
2.50 dmso
2.49 dmso
H2N
N
N=N
15
1H NMR
DMSO-d6, 600 MHz
1.00
2.01
2.03
1.02
2.02
2.04
2.02
2.00
f1 (ppm)

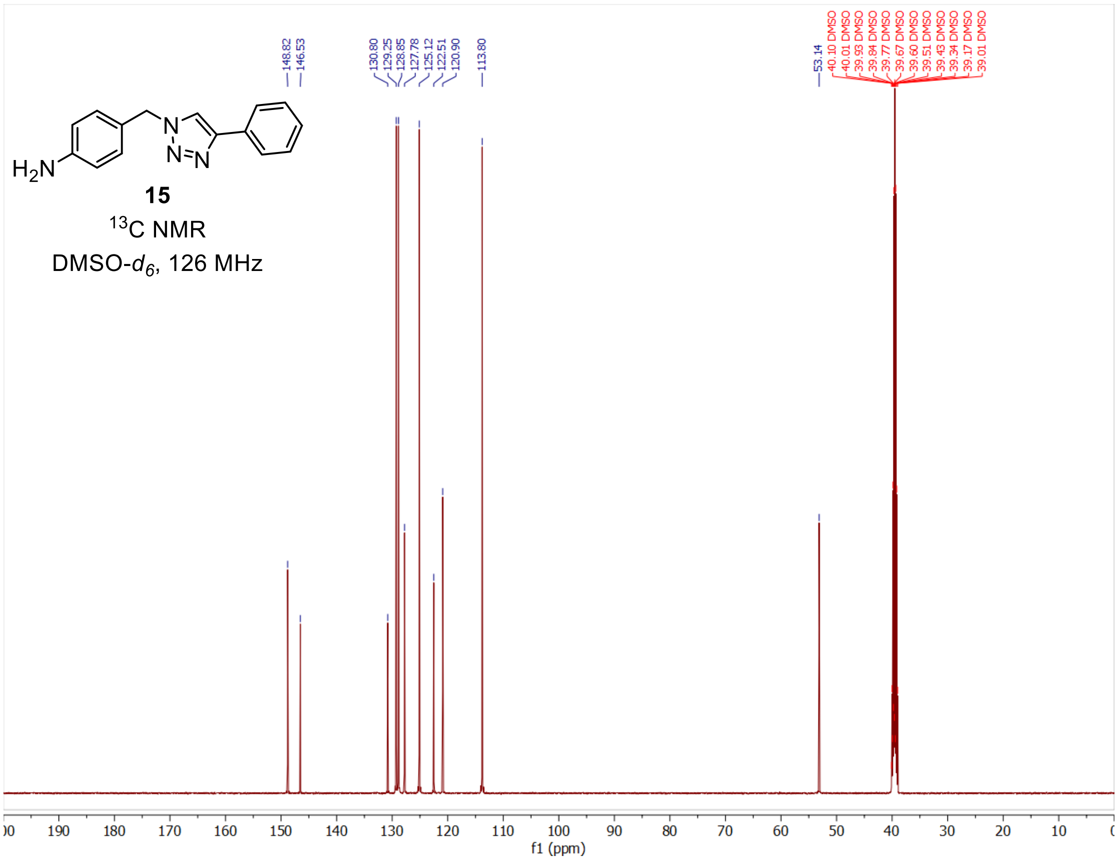
148.82
146.53
130.80
129.25
128.85
127.78
125.12
122.51
120.90
113.80
53.14
40.10 DMSO
40.01 DMSO
39.93 DMSO
39.84 DMSO
39.77 DMSO
39.67 DMSO
39.60 DMSO
39.51 DMSO
39.43 DMSO
39.34 DMSO
39.17 DMSO
39.01 DMSO
H2N
N
N=N
15
13C NMR
DMSO-d6, 126 MHz
f1 (ppm)

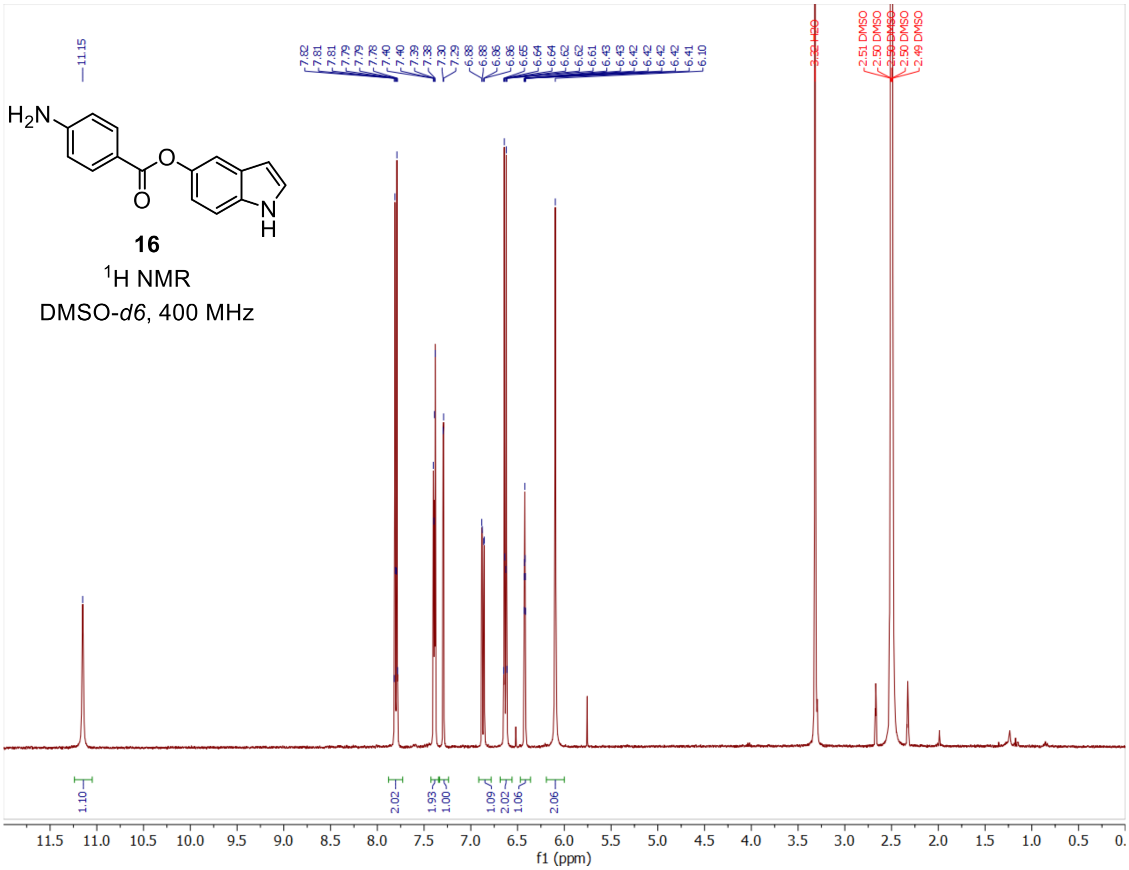

16

^{1}H NMR

DMSO-*d6*, 400 MHz

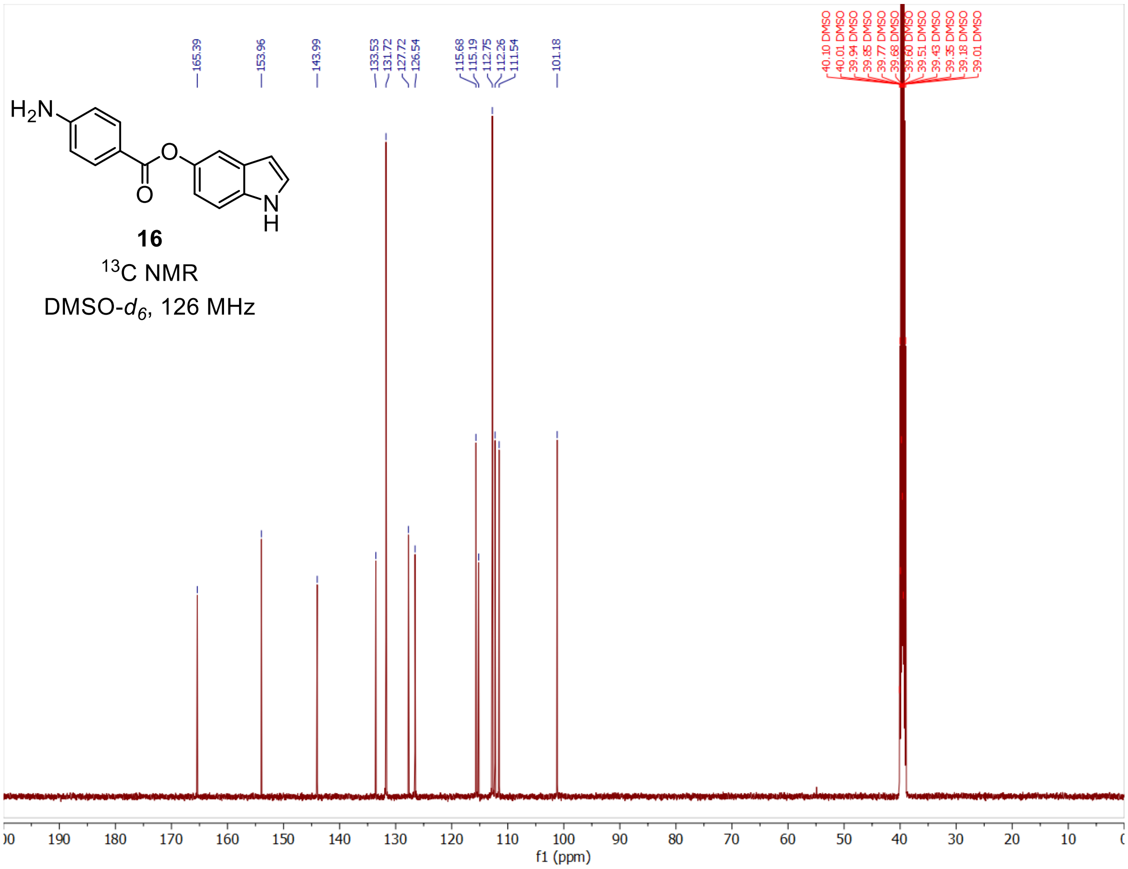

16

^{13}C NMR

DMSO-d_6, 126 MHz

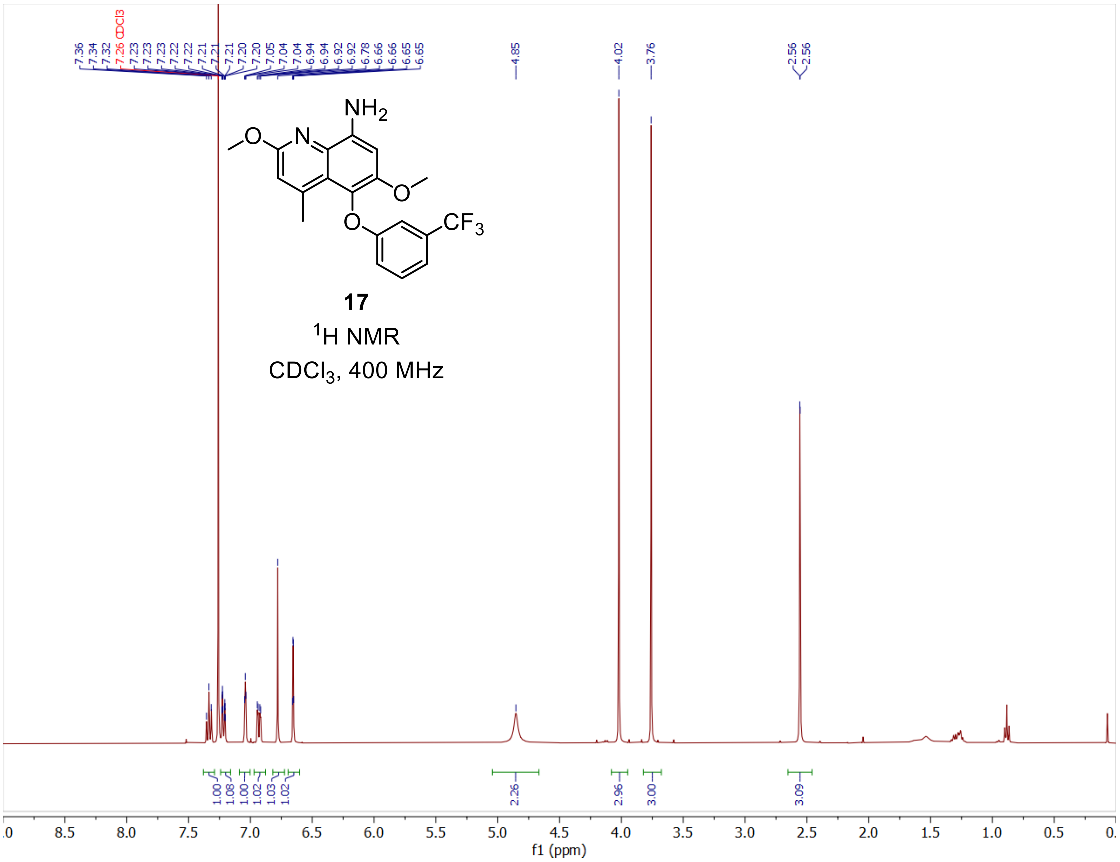
7.36 7.34 7.32 7.26 CDCl3 7.23 7.23 7.23 7.22 7.22 7.21 7.21 7.21 7.20 7.20 7.05 7.04 7.04 6.94 6.94 6.92 6.92 6.78 6.66 6.66 6.65 6.65
4.85
4.02
3.76
2.56 2.56
NH2
O
N
O
O
CF3
17
1H NMR
CDCl3, 400 MHz
1.00 1.08 1.00 1.02 1.03 1.02
2.26
2.96
3.00
3.09
f1 (ppm)

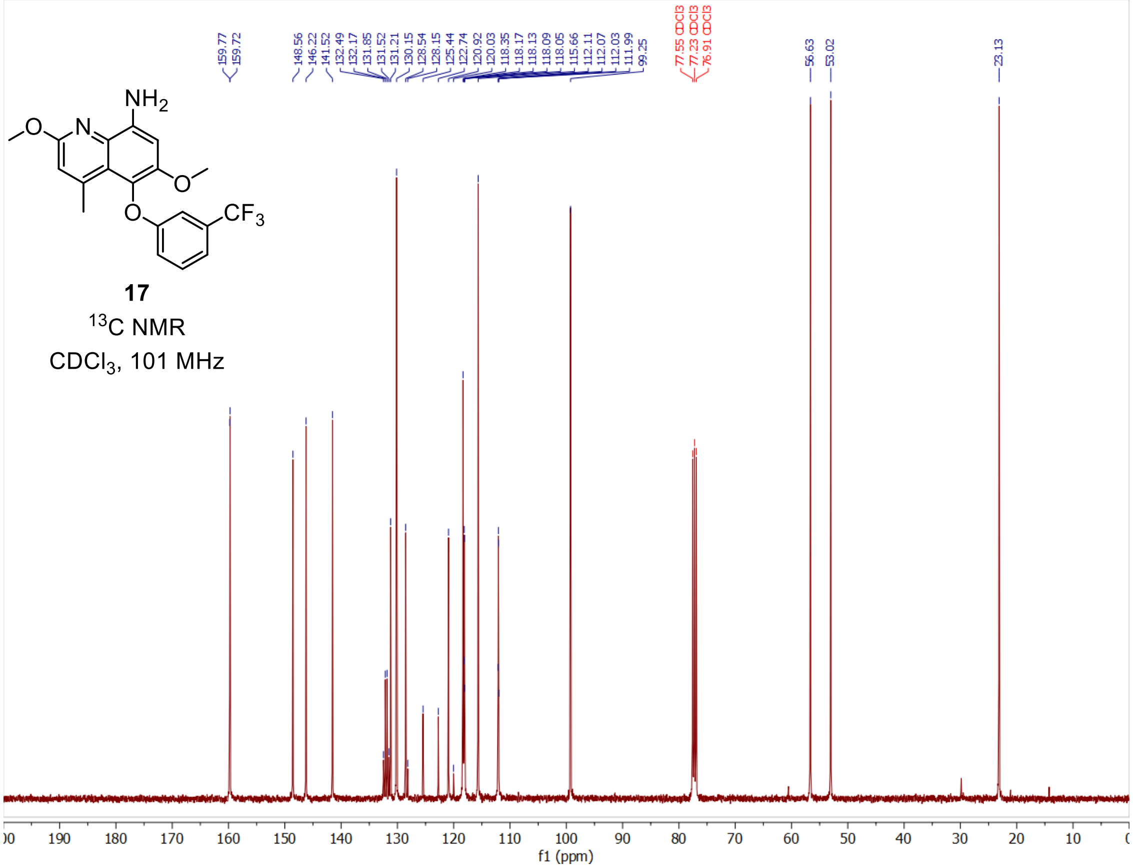
159.77 159.72
148.56 146.22 141.52 132.49 132.17 131.85 131.52 131.52 131.21 130.15 128.54 128.15 125.44 122.74 120.92 120.03 118.35 118.17 118.13 118.09 118.05 115.66 112.11 112.07 112.03 111.99 99.25
77.55 CDCl3 77.23 CDCl3 76.91 CDCl3
56.63
53.02
23.13
NH2
O
N
O
O
CF3
17
13C NMR
CDCl3, 101 MHz
f1 (ppm)

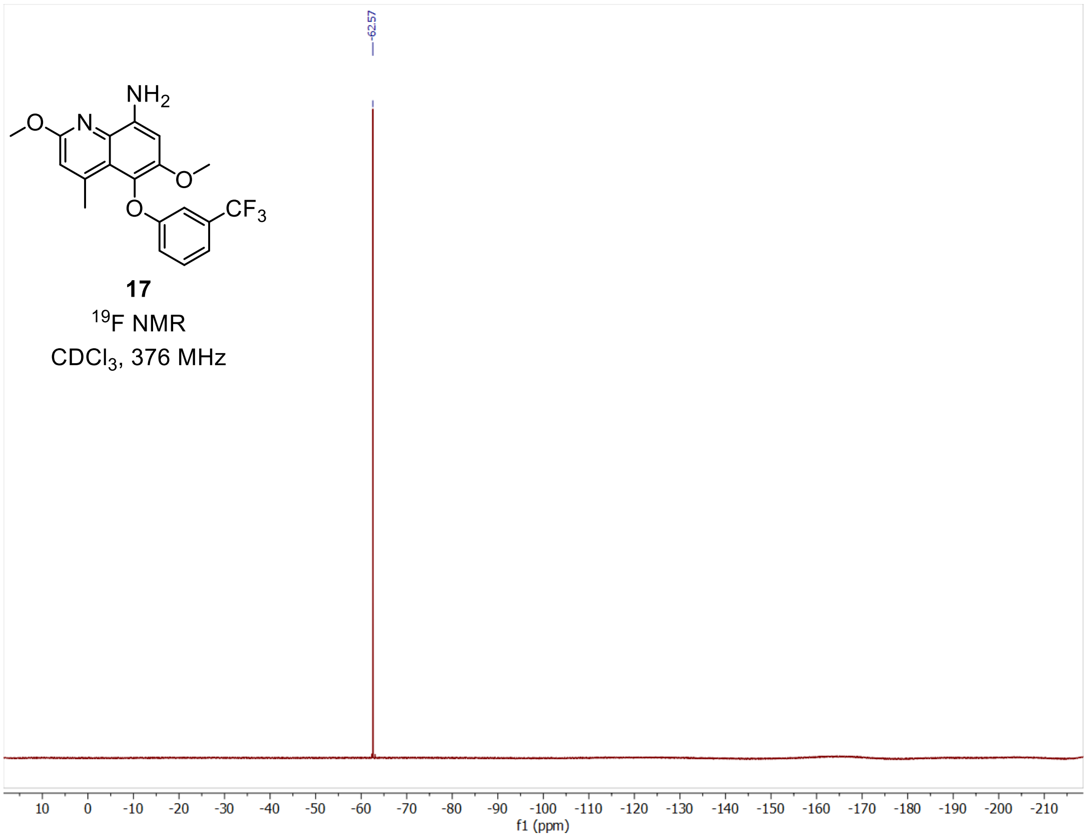
-62.57
NH2
N
O
CF3
17
19F NMR
CDCl3, 376 MHz
10 0 -10 -20 -30 -40 -50 -60 -70 -80 -90 -100 -110 -120 -130 -140 -150 -160 -170 -180 -190 -200 -210
f1 (ppm)

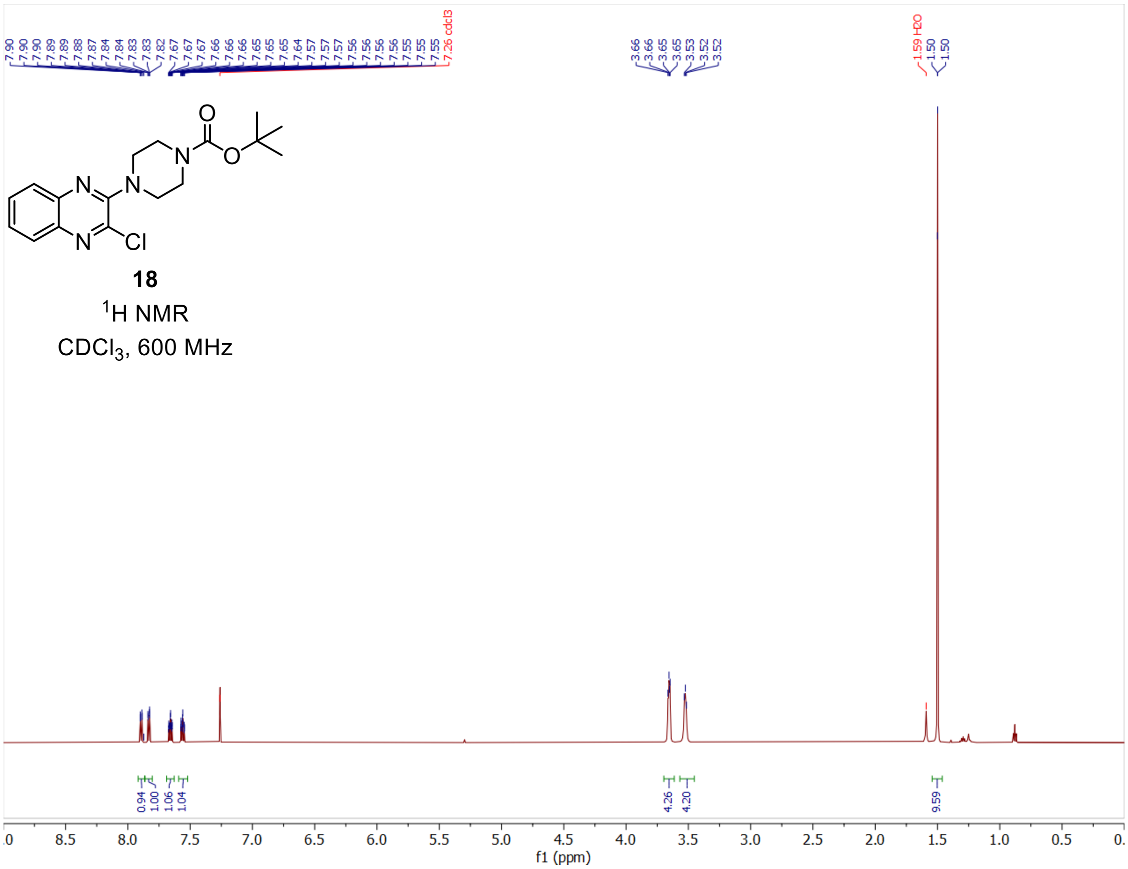

18

^{1}H NMR

$CDCl_3$, 600 MHz

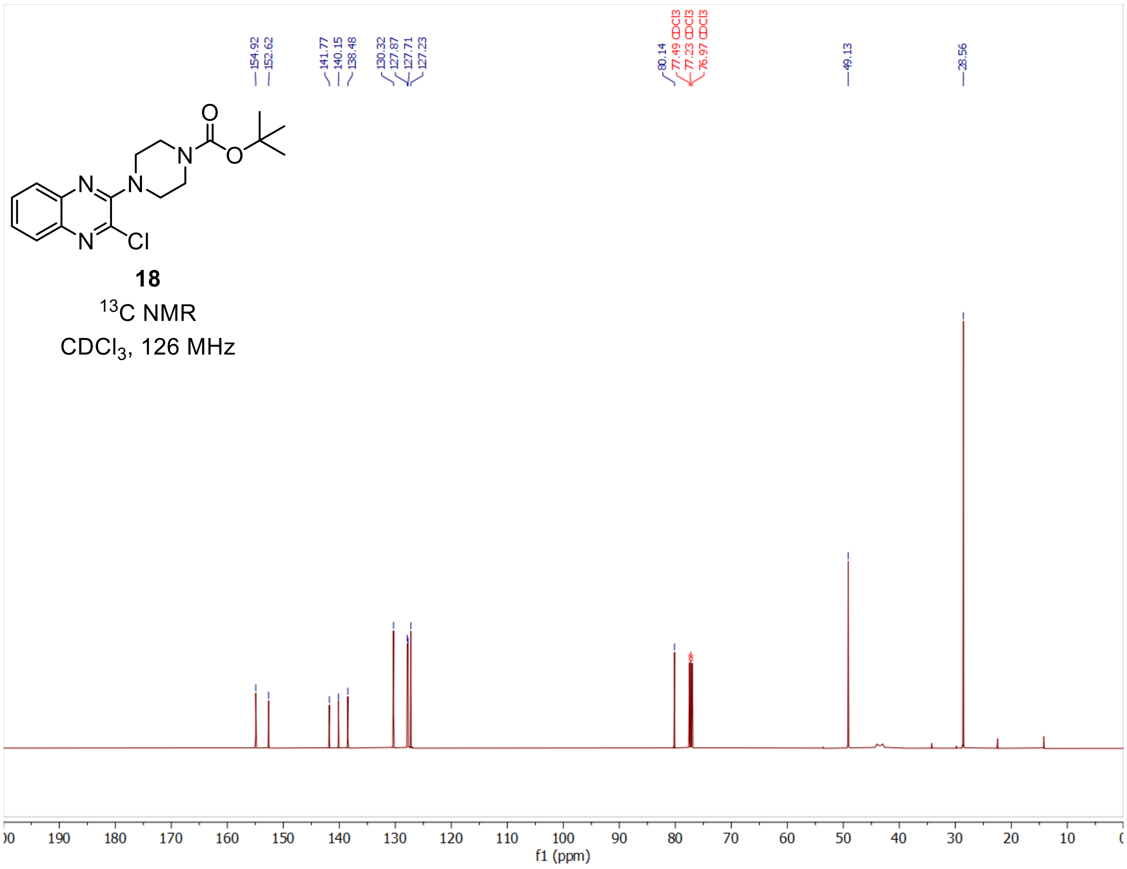

18

^{13}C NMR

$CDCl_3$, 126 MHz

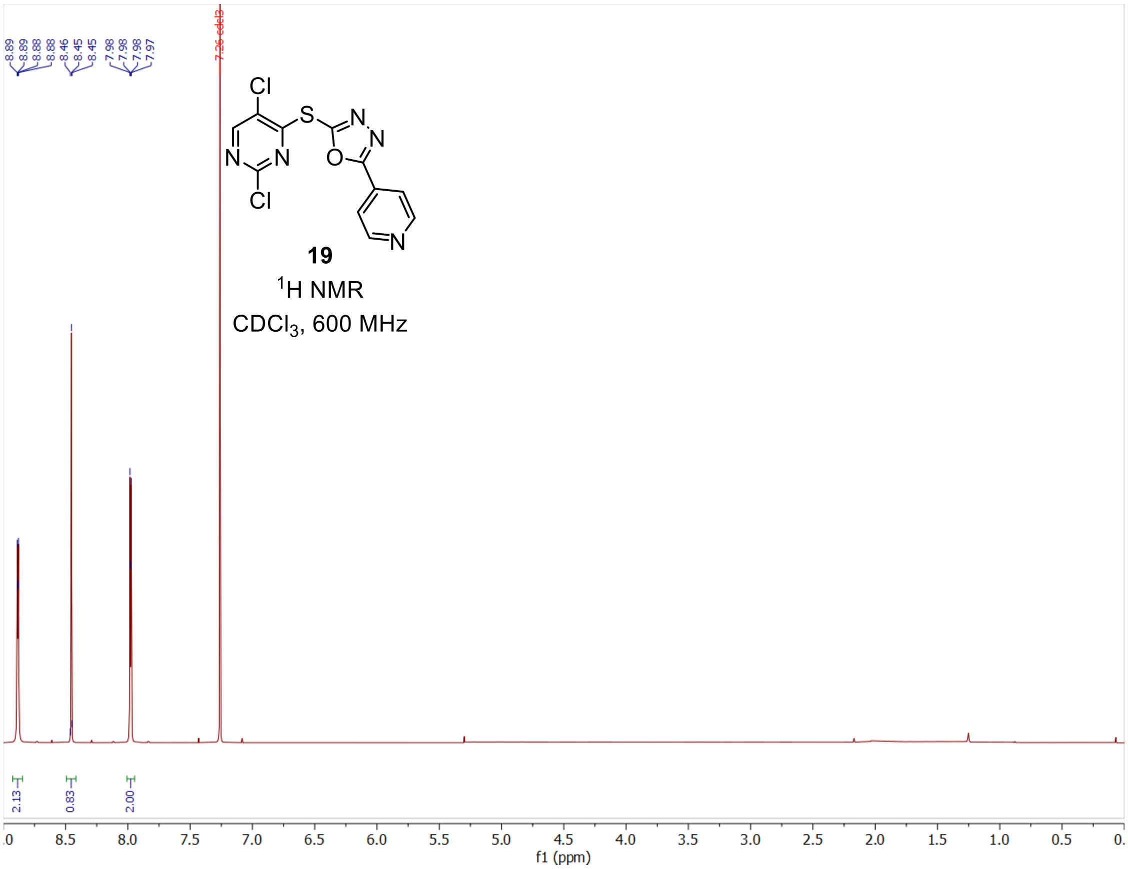
8.89
8.89
8.88
8.88
8.46
8.45
8.45
7.98
7.98
7.98
7.97
7.26 cdcl3
Cl
S
N
N
N
N
O
Cl
N
19
1H NMR
CDCl3, 600 MHz
2.13
0.83
2.00
f1 (ppm)

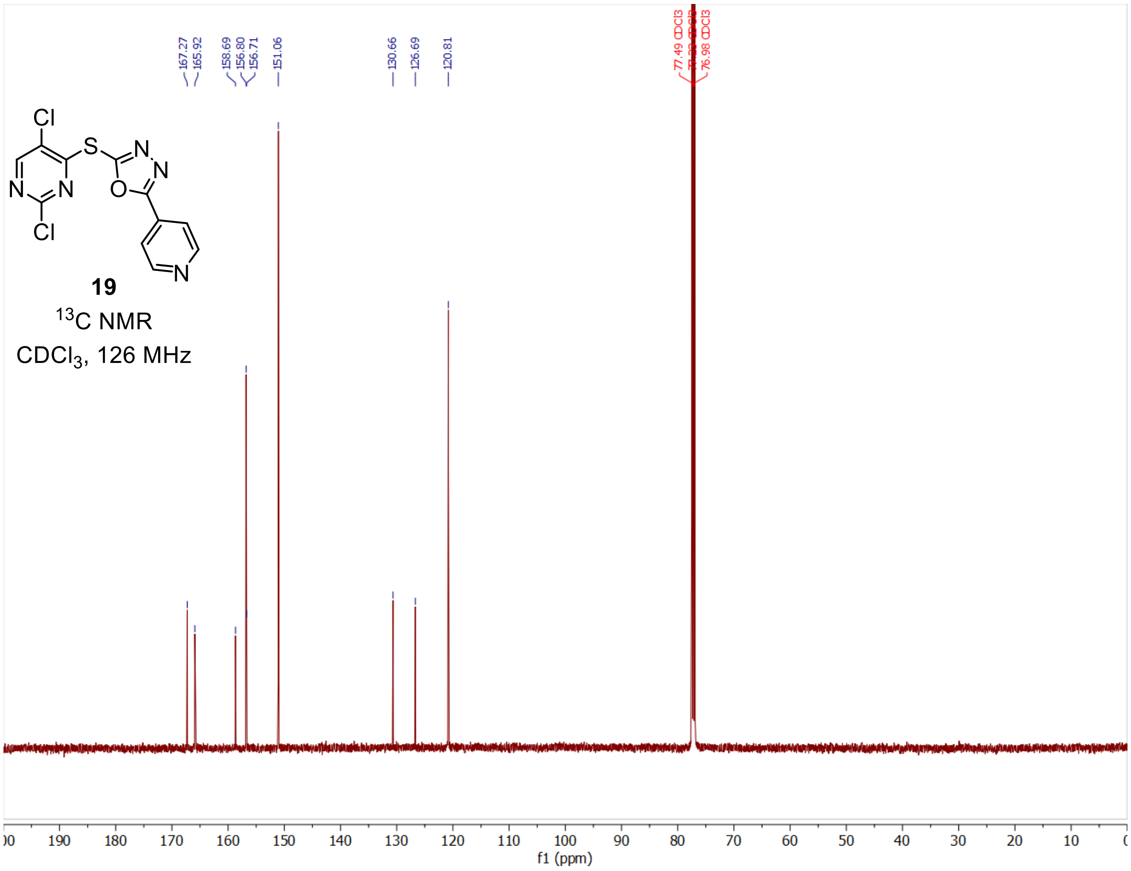
167.27
165.92
158.69
156.80
156.71
151.06
130.66
126.69
120.81
77.49 CDCl3
76.98 CDCl3
Cl
S
N
N
N
N
O
Cl
N
19
13C NMR
CDCl3, 126 MHz
f1 (ppm)

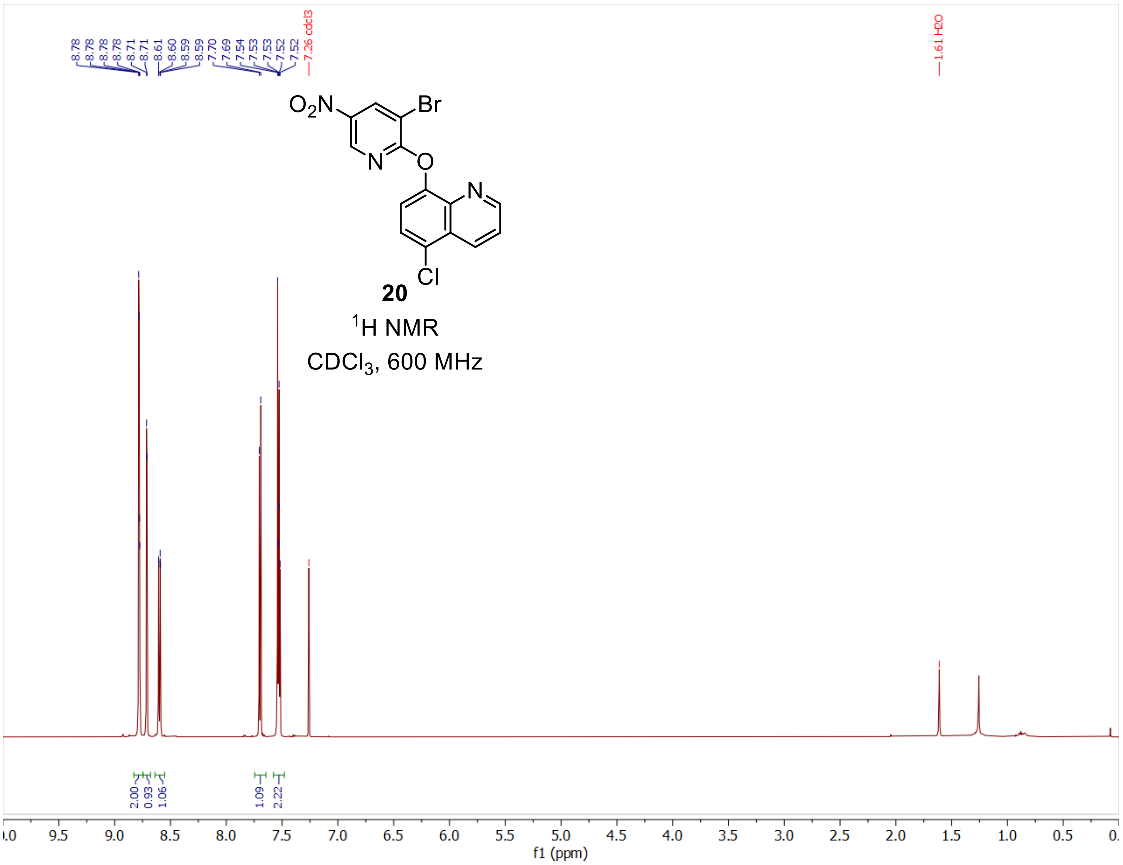
8.78
8.78
8.78
8.78
8.71
8.71
8.61
8.60
8.59
8.59
7.70
7.69
7.54
7.53
7.53
7.52
7.52
7.26 cdcl3
1.61 H2O
O_2N
Br
N
O
N
Cl
20
^{1}H NMR
$CDCl_3$, 600 MHz
2.00
0.93
1.06
1.09
2.22
f1 (ppm)

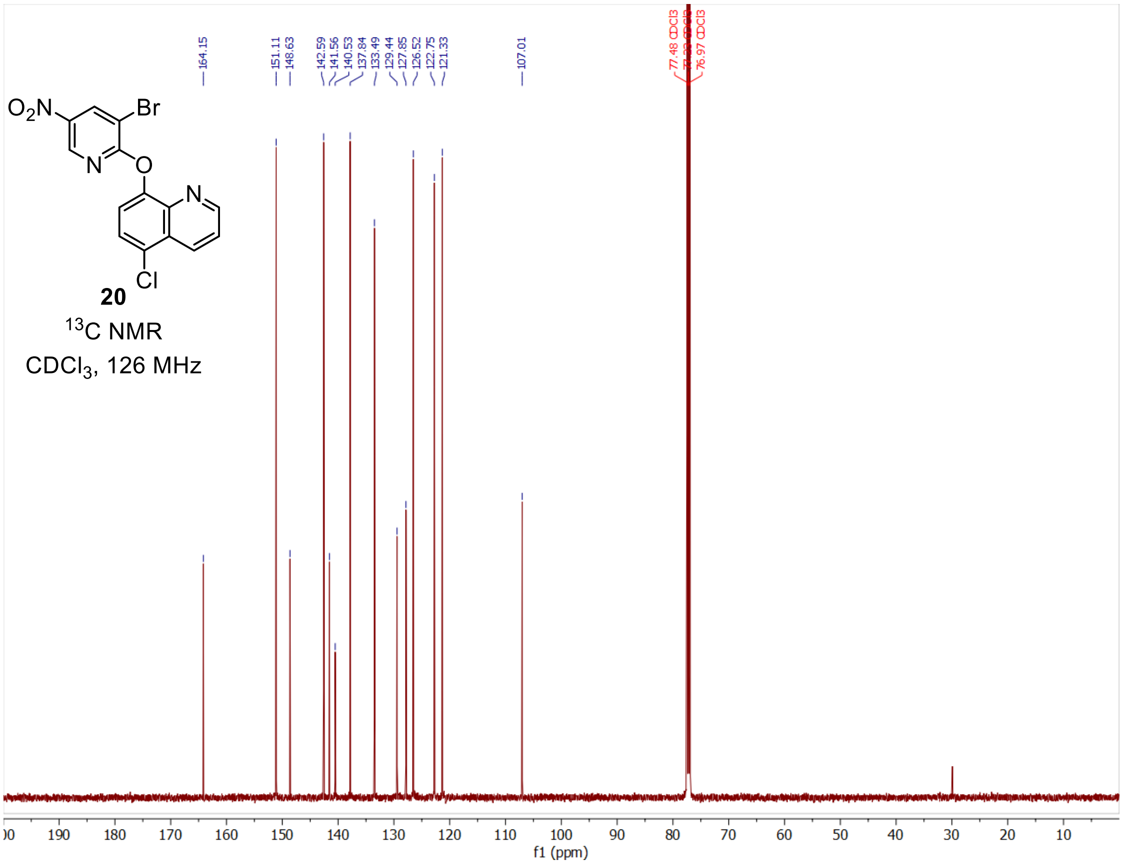
164.15
151.11
148.63
142.59
141.56
140.53
137.84
133.49
129.44
127.85
126.52
122.75
121.33
107.01
77.48 CDCl3
76.97 CDCl3
O_2N
Br
N
O
N
Cl
20
^{13}C NMR
$CDCl_3$, 126 MHz
f1 (ppm)

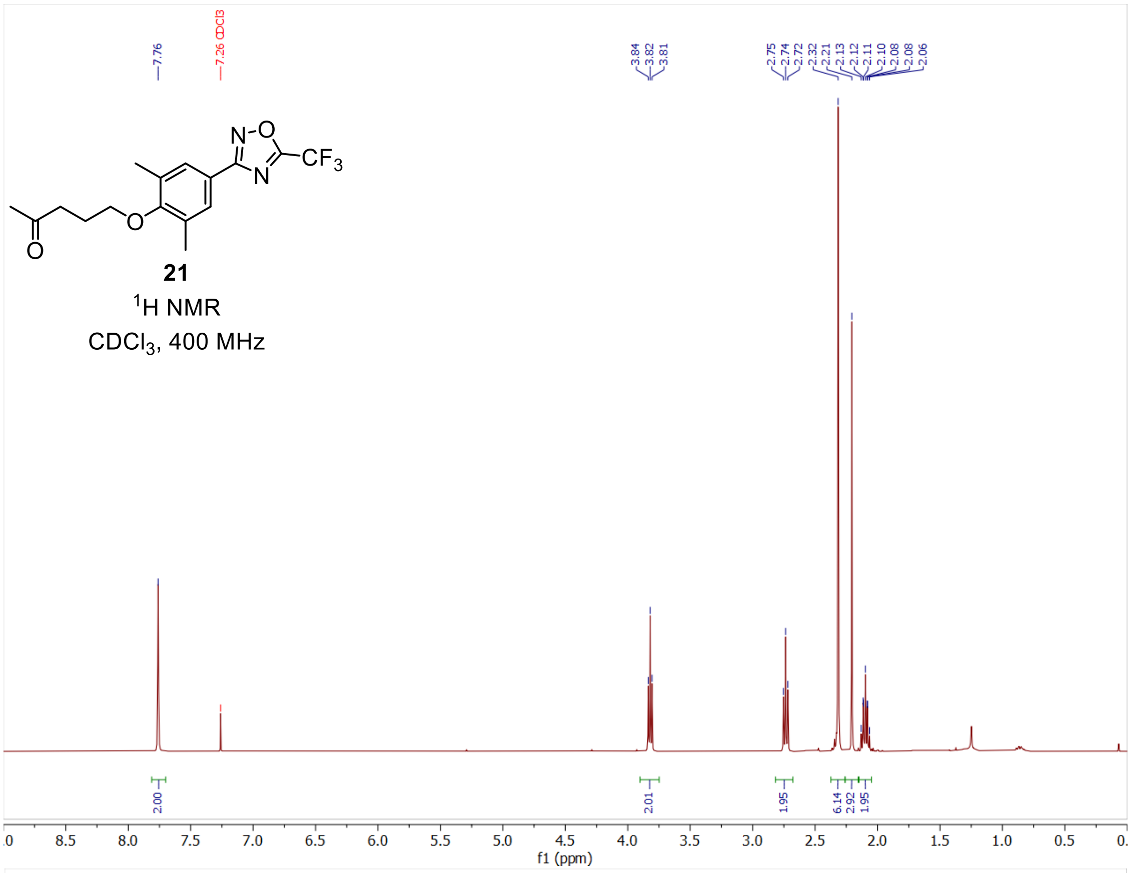

21
^{1}H NMR
CDCl$_3$, 400 MHz
7.76
7.26 CDCl3
3.84
3.82
3.81
2.75
2.74
2.72
2.32
2.21
2.13
2.12
2.11
2.10
2.08
2.08
2.06
2.00
2.01
1.95
6.14
2.92
1.95
f1 (ppm)

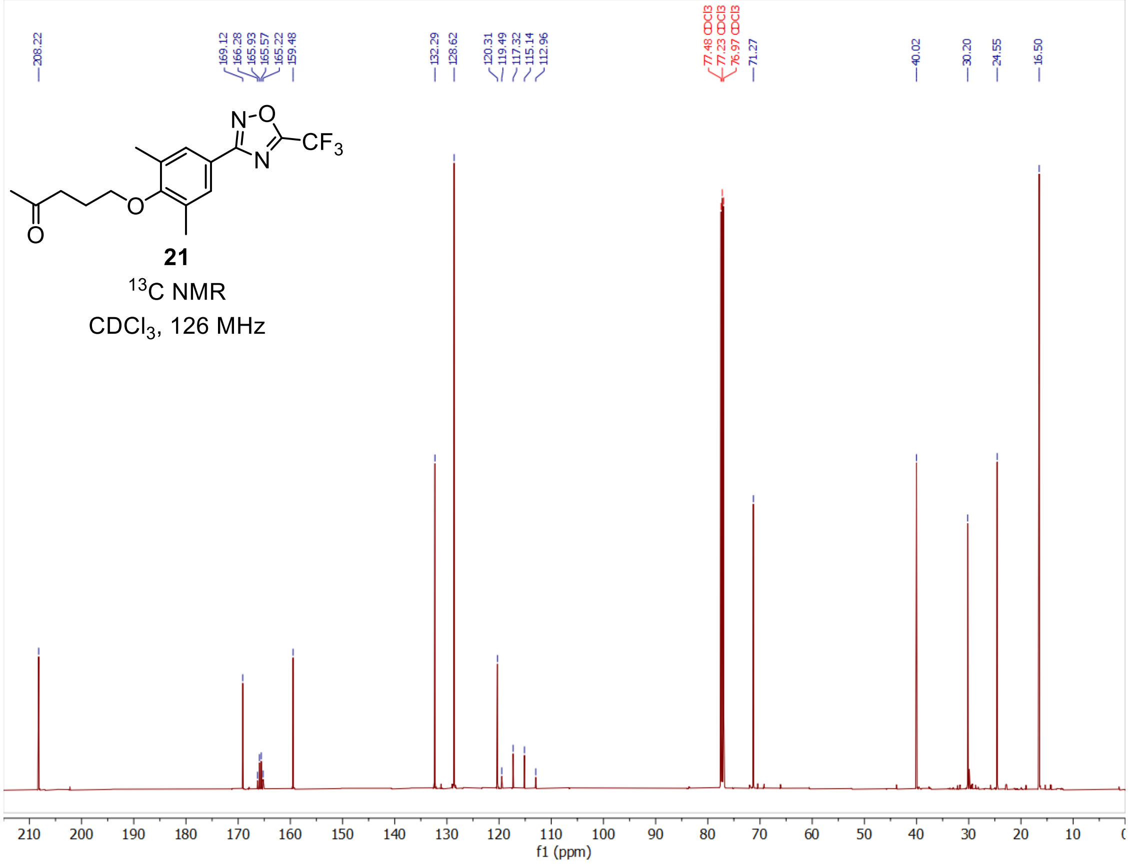

21
^{13}C NMR
CDCl$_3$, 126 MHz
208.22
169.12
166.28
165.93
165.57
165.22
159.48
132.29
128.62
120.31
119.49
117.32
115.14
112.96
77.48 CDCl3
77.23 CDCl3
76.97 CDCl3
71.27
40.02
30.20
24.55
16.50
f1 (ppm)

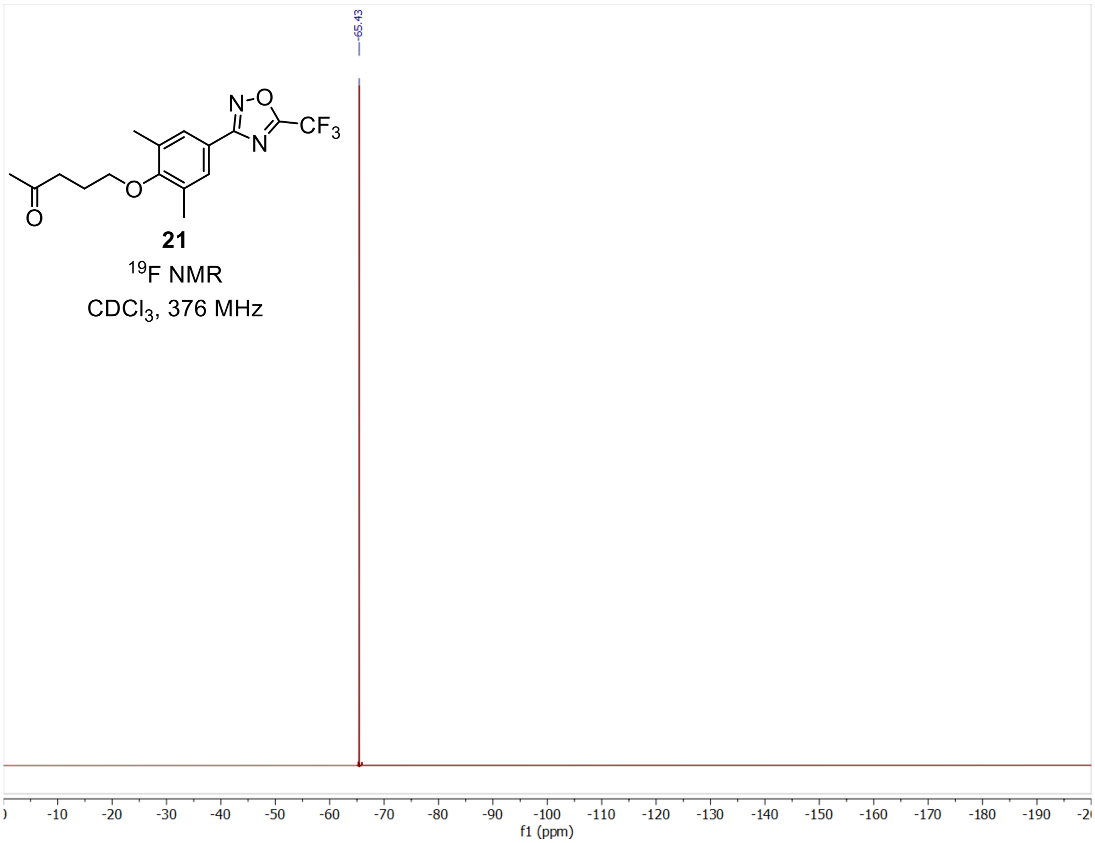

-65.43
N
O
N
CF3
O
O
21
19F NMR
CDCl3, 376 MHz
-10
-20
-30
-40
-50
-60
-70
-80
-90
-100
-110
-120
-130
-140
-150
-160
-170
-180
-190
f1 (ppm)

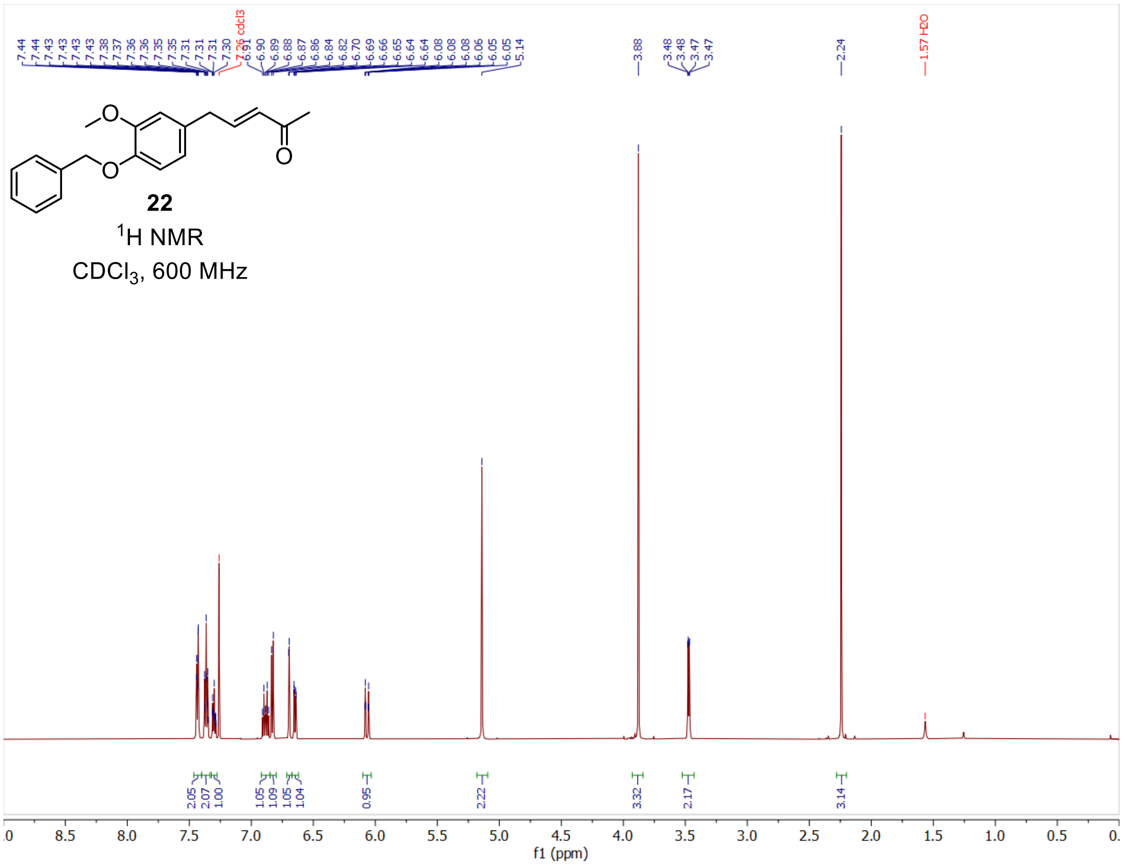

22
^{1}H NMR
$CDCl_3$, 600 MHz
7.26 cdcl3
3.88
3.48
3.48
3.47
3.47
2.24
1.57 H2O
2.05
2.07
1.00
1.05
1.09
1.05
1.04
0.95
2.22
3.32
2.17
3.14
f1 (ppm)

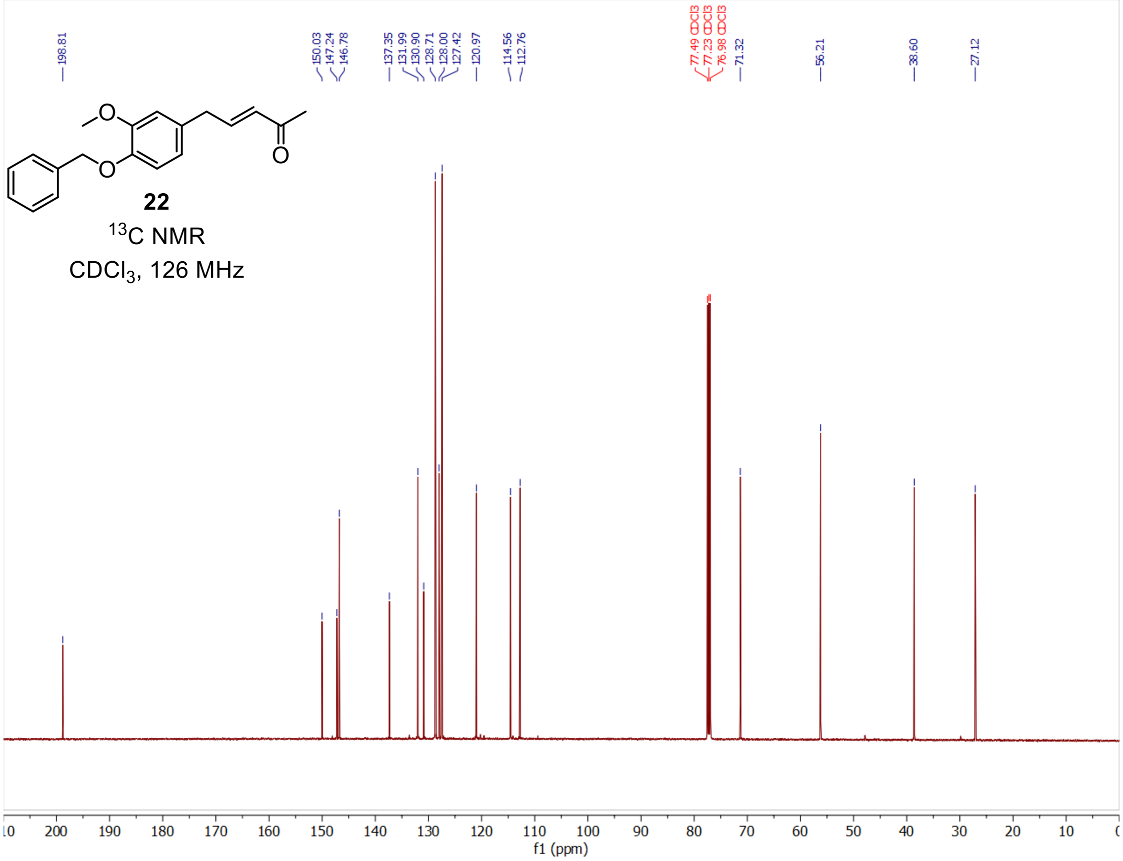

22
^{13}C NMR
$CDCl_3$, 126 MHz
198.81
150.03
147.24
146.78
137.35
131.99
130.90
128.71
128.00
127.42
120.97
114.56
112.76
77.49 CDCl3
77.23 CDCl3
76.98 CDCl3
71.32
56.21
38.60
27.12
f1 (ppm)

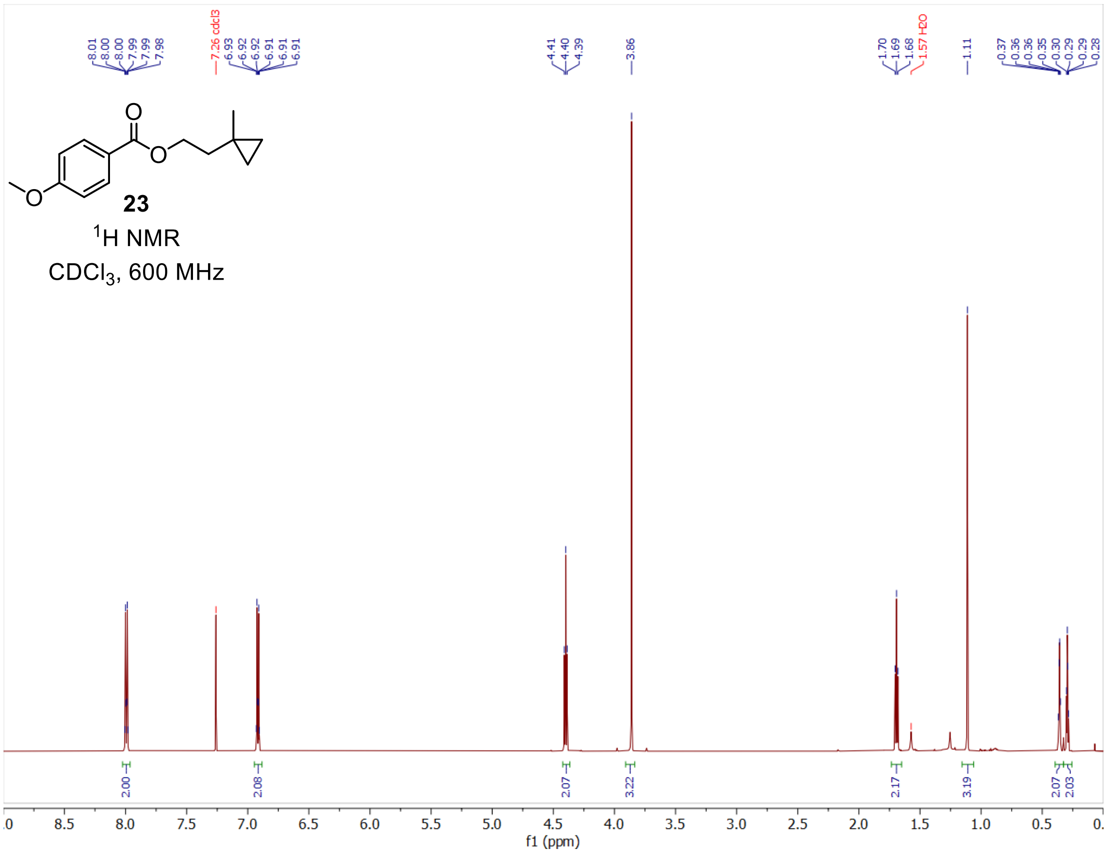
23
^{1}H NMR
$CDCl_3$, 600 MHz
f1 (ppm)

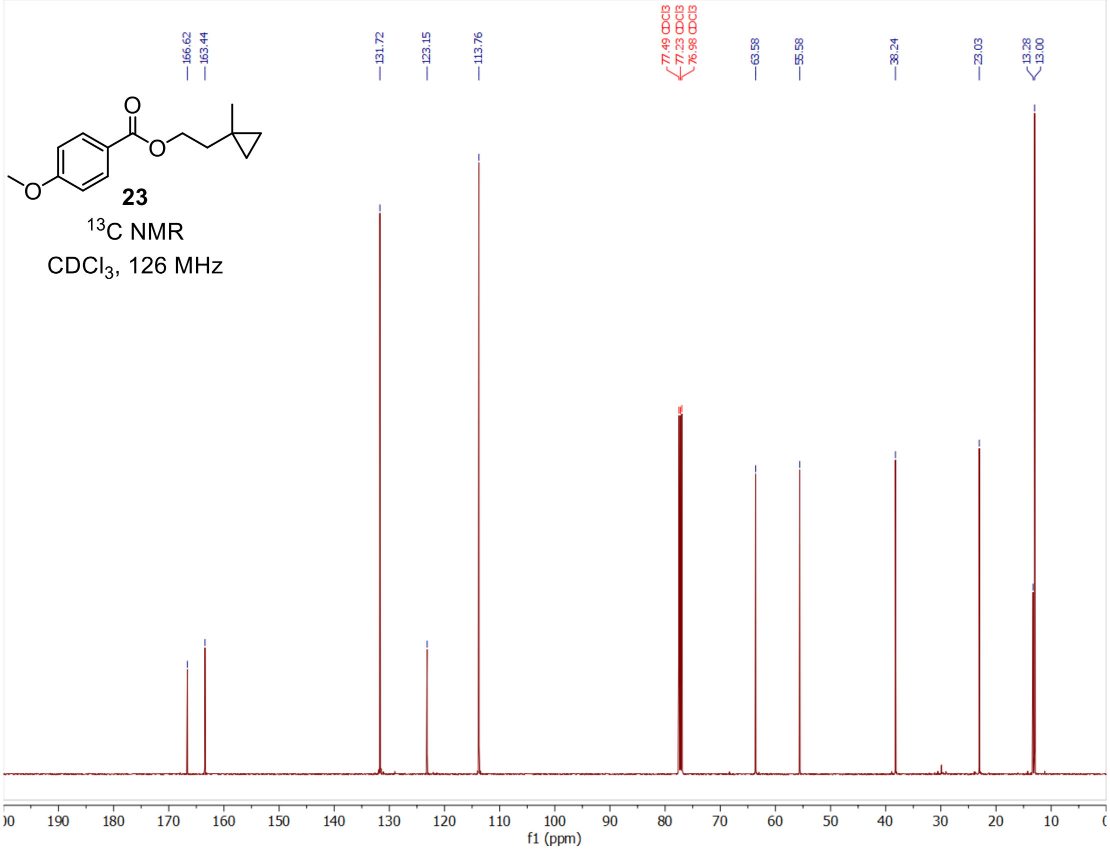
23
^{13}C NMR
$CDCl_3$, 126 MHz
f1 (ppm)

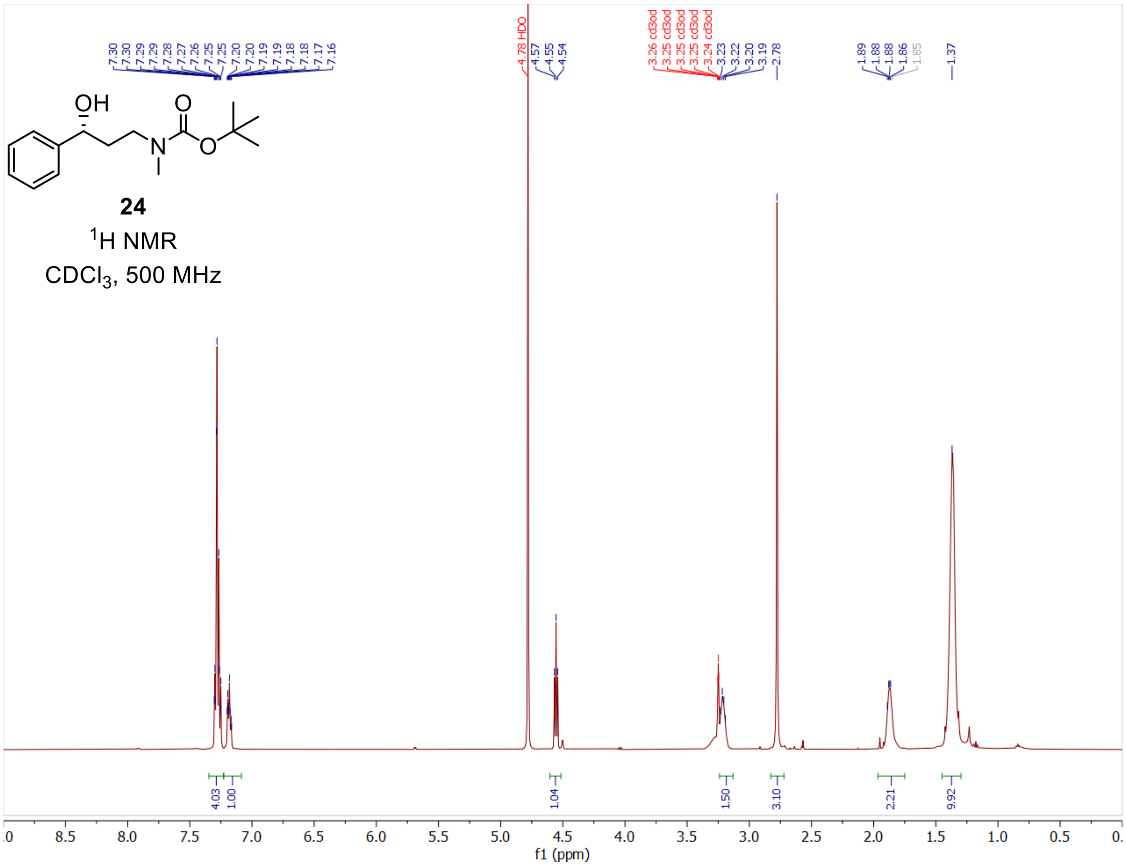
OH
O
N
O
24
1H NMR
CDCl3, 500 MHz
7.30 7.30 7.29 7.29 7.28 7.27 7.26 7.25 7.25 7.25 7.20 7.20 7.19 7.19 7.18 7.17 7.16
4.78 HDO
4.57 4.55 4.54
3.26 cd3od 3.25 cd3od 3.25 cd3od 3.25 cd3od 3.24 cd3od
3.23 3.22 3.20 3.19
2.78
1.89 1.88 1.88 1.86 1.85
1.37
4.03 1.00 1.04 1.50 3.10 2.21 9.92
f1 (ppm)

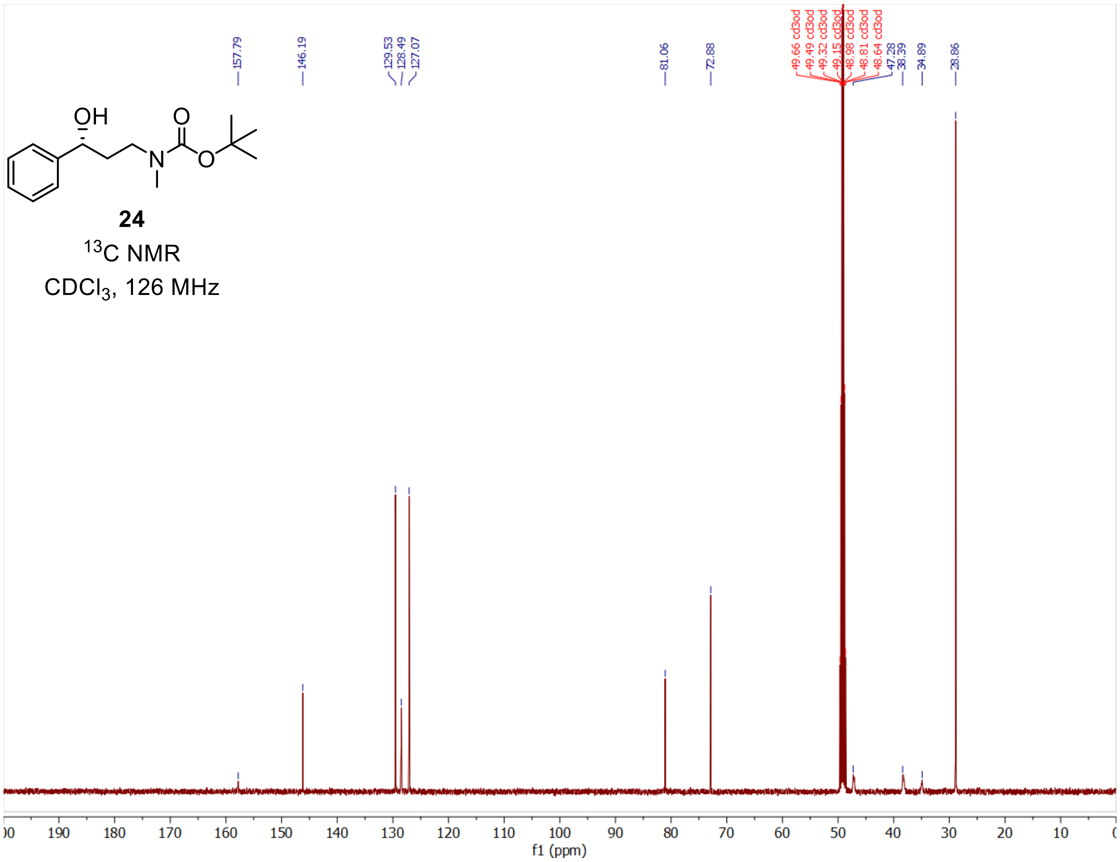
OH
O
N
O
24
13C NMR
CDCl3, 126 MHz
157.79
146.19
129.53 128.49 127.07
81.06
72.88
49.66 cd3od 49.49 cd3od 49.32 cd3od 49.15 cd3od 48.98 cd3od 48.81 cd3od 48.64 cd3od
47.28 38.39
34.89
28.86
f1 (ppm)

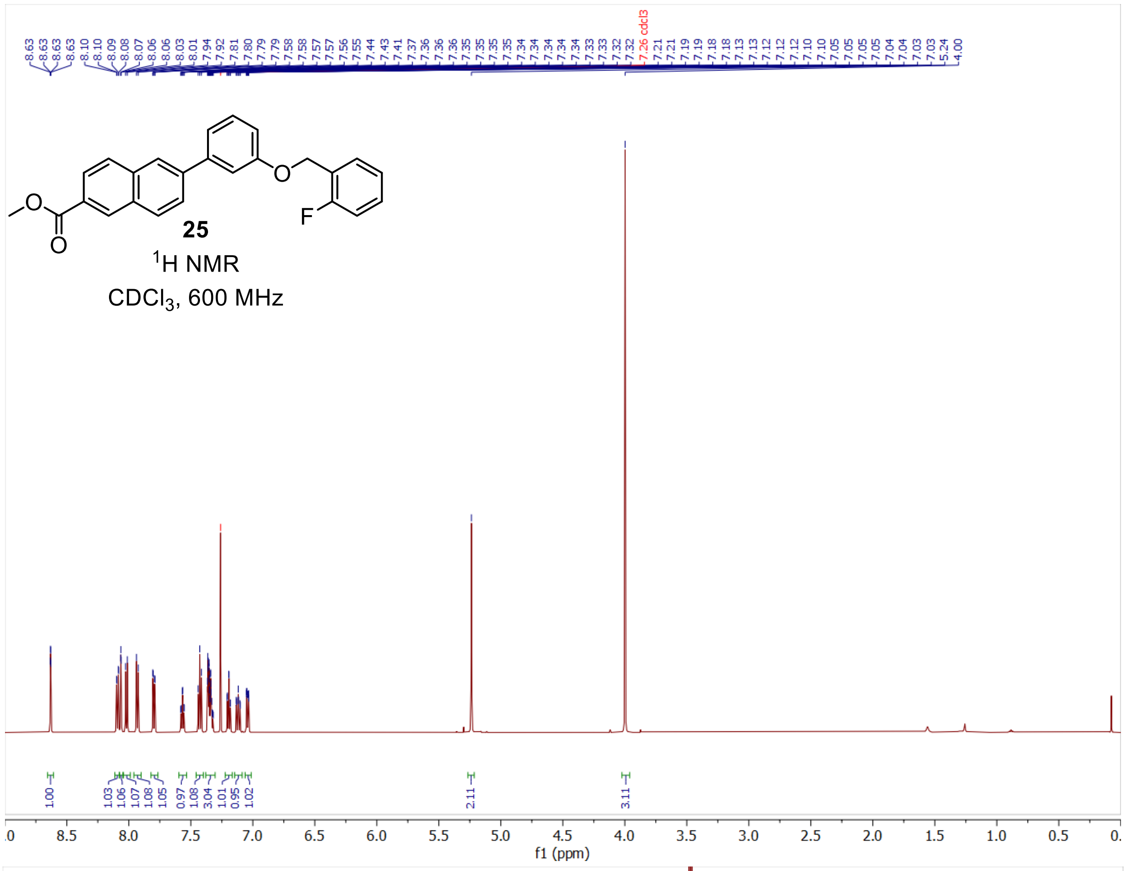
8.63
8.63
8.63
8.63
8.10
8.10
8.09
8.08
8.07
8.06
8.06
8.03
8.01
7.94
7.92
7.81
7.80
7.79
7.79
7.58
7.58
7.57
7.57
7.56
7.55
7.44
7.43
7.41
7.37
7.36
7.36
7.36
7.35
7.35
7.35
7.35
7.34
7.34
7.34
7.34
7.33
7.33
7.32
7.32
7.26 cdcl3
7.21
7.21
7.19
7.19
7.18
7.18
7.13
7.13
7.12
7.12
7.12
7.10
7.10
7.05
7.05
7.05
7.04
7.04
7.03
5.24
4.00
O
O
O
F
25
1H NMR
CDCl3, 600 MHz
1.00
1.03
1.06
1.07
1.08
1.05
0.97
1.08
3.04
1.01
0.95
1.02
2.11
3.11
f1 (ppm)

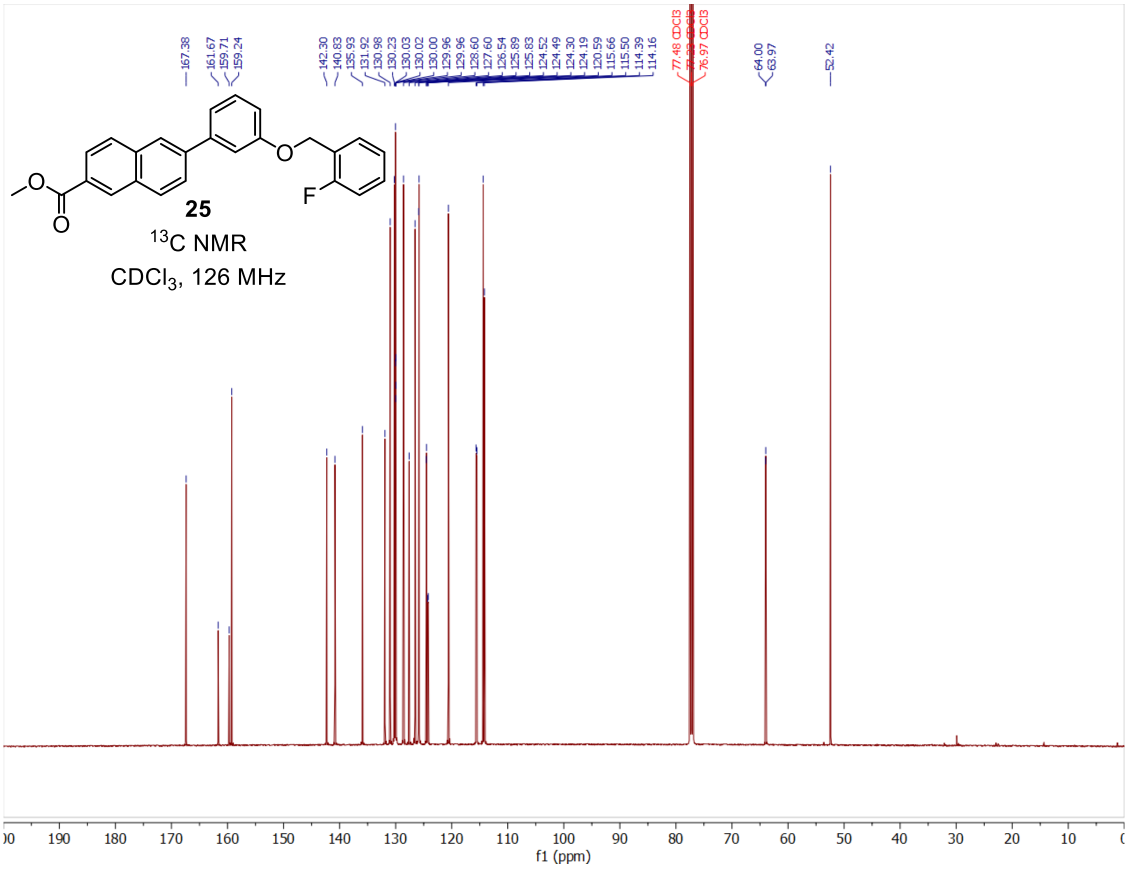
167.38
161.67
159.71
159.24
142.30
140.83
135.93
131.92
130.98
130.23
130.03
130.02
130.00
129.96
129.96
128.60
127.60
126.54
125.89
125.83
124.52
124.49
124.30
124.19
120.59
115.66
115.50
114.39
114.16
77.48 CDCl3
76.97 CDCl3
64.00
63.97
52.42
O
O
O
F
25
13C NMR
CDCl3, 126 MHz
f1 (ppm)

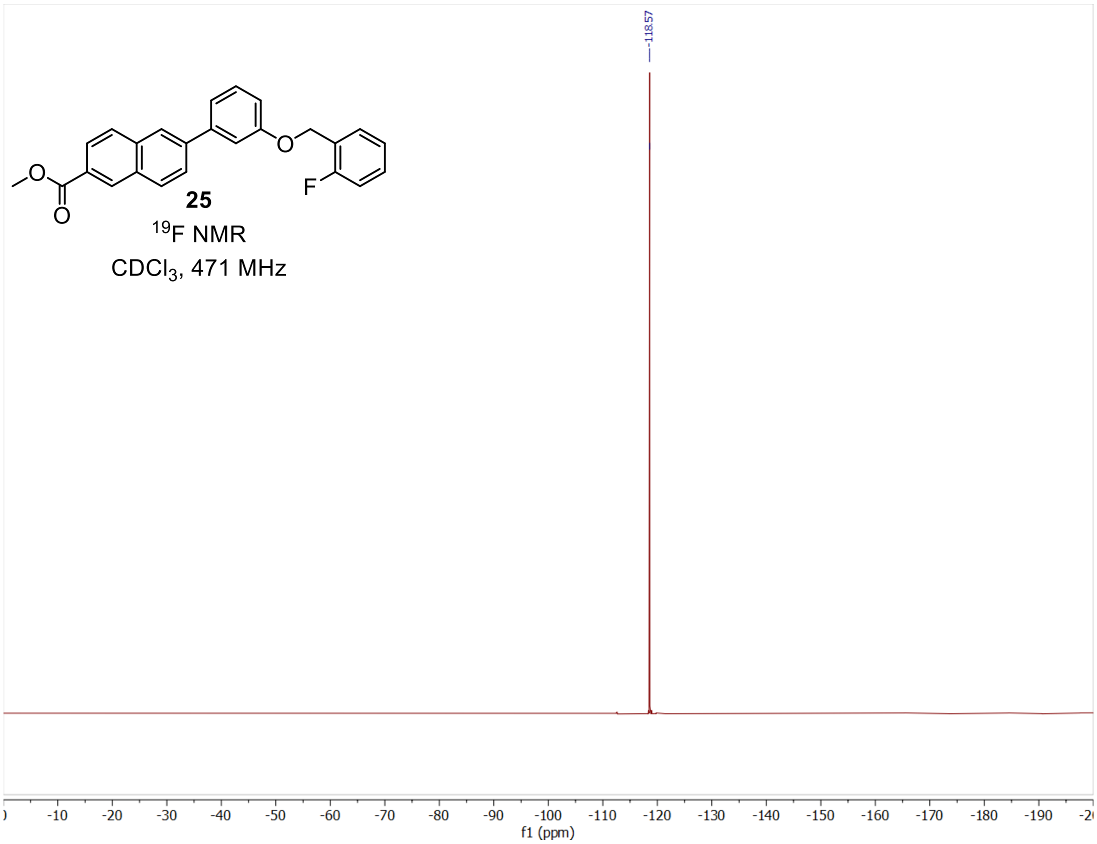

--118.57
25
19F NMR
CDCl3, 471 MHz
f1 (ppm)

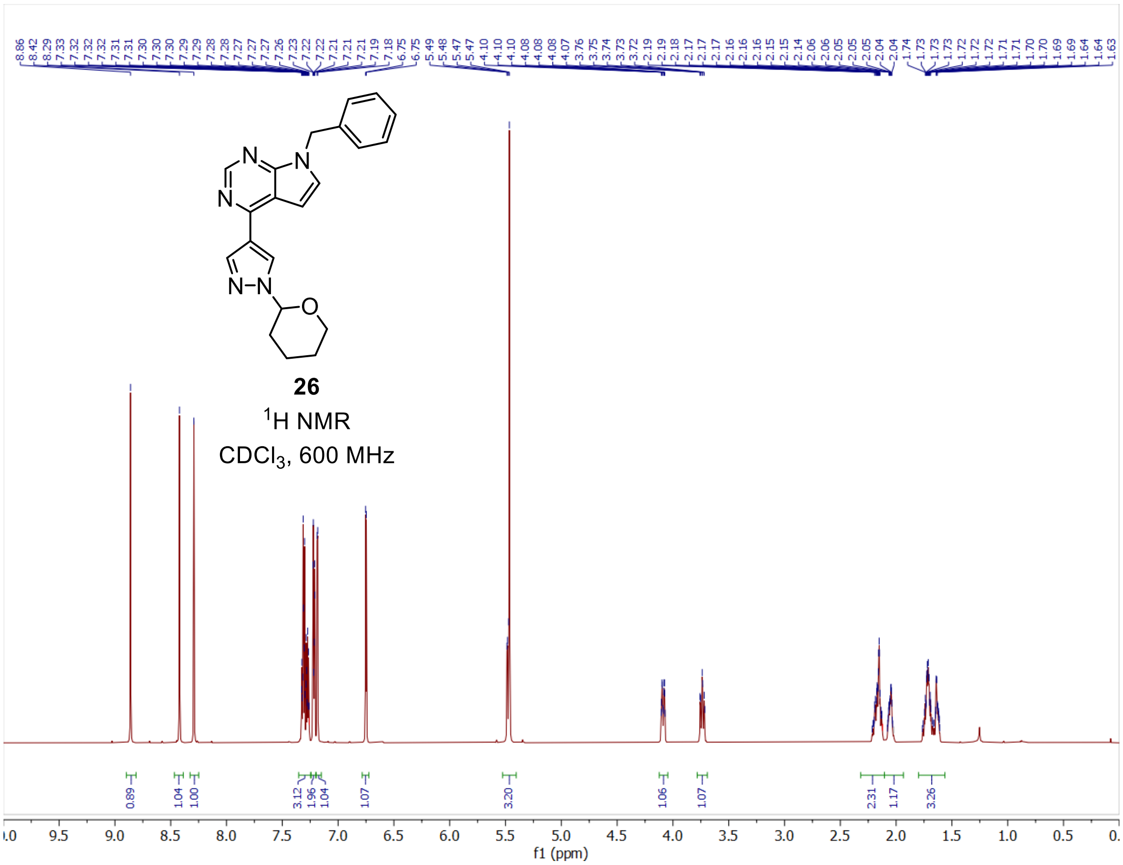
8.86
8.42
8.29
7.33
7.32
7.32
7.32
7.31
7.31
7.30
7.30
7.30
7.29
7.29
7.28
7.28
7.27
7.27
7.27
7.26
7.23
7.22
7.22
7.21
7.21
7.21
7.19
7.18
6.75
6.75
5.49
5.48
5.47
5.47
4.10
4.10
4.08
4.08
4.08
4.07
3.76
3.75
3.74
3.73
3.72
2.19
2.18
2.17
2.17
2.17
2.16
2.16
2.15
2.15
2.14
2.06
2.06
2.05
2.05
2.05
2.04
2.04
1.74
1.73
1.73
1.73
1.72
1.72
1.72
1.71
1.71
1.70
1.69
1.69
1.64
1.63
N
N
N
N-N
O
26
1H NMR
CDCl3, 600 MHz
0.89
1.04
1.00
3.12
1.96
1.04
1.07
3.20
1.06
1.07
2.31
1.17
3.26
f1 (ppm)

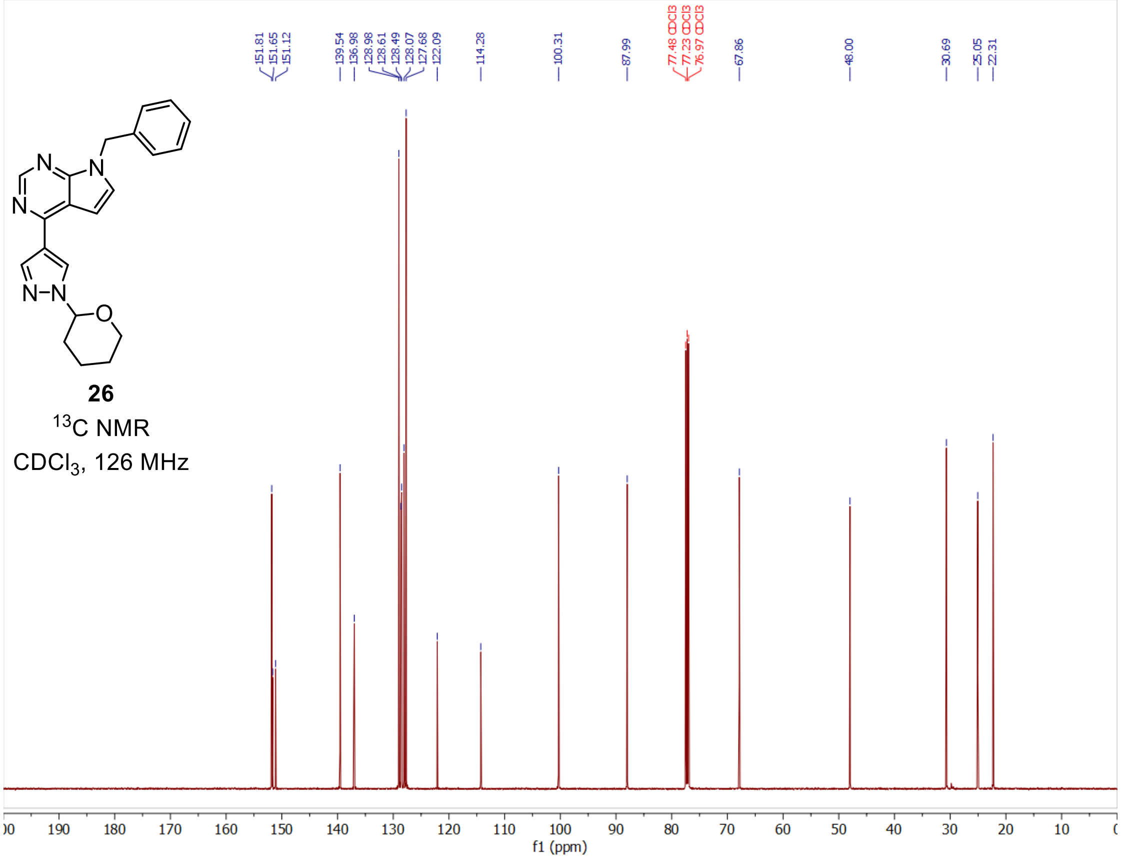
151.81
151.65
151.12
139.54
136.98
128.98
128.61
128.49
128.07
127.68
122.09
114.28
100.31
87.99
77.48 CDCl3
77.23 CDCl3
76.97 CDCl3
67.86
48.00
30.69
25.05
22.31
N
N
N
N-N
O
26
13C NMR
CDCl3, 126 MHz
f1 (ppm)

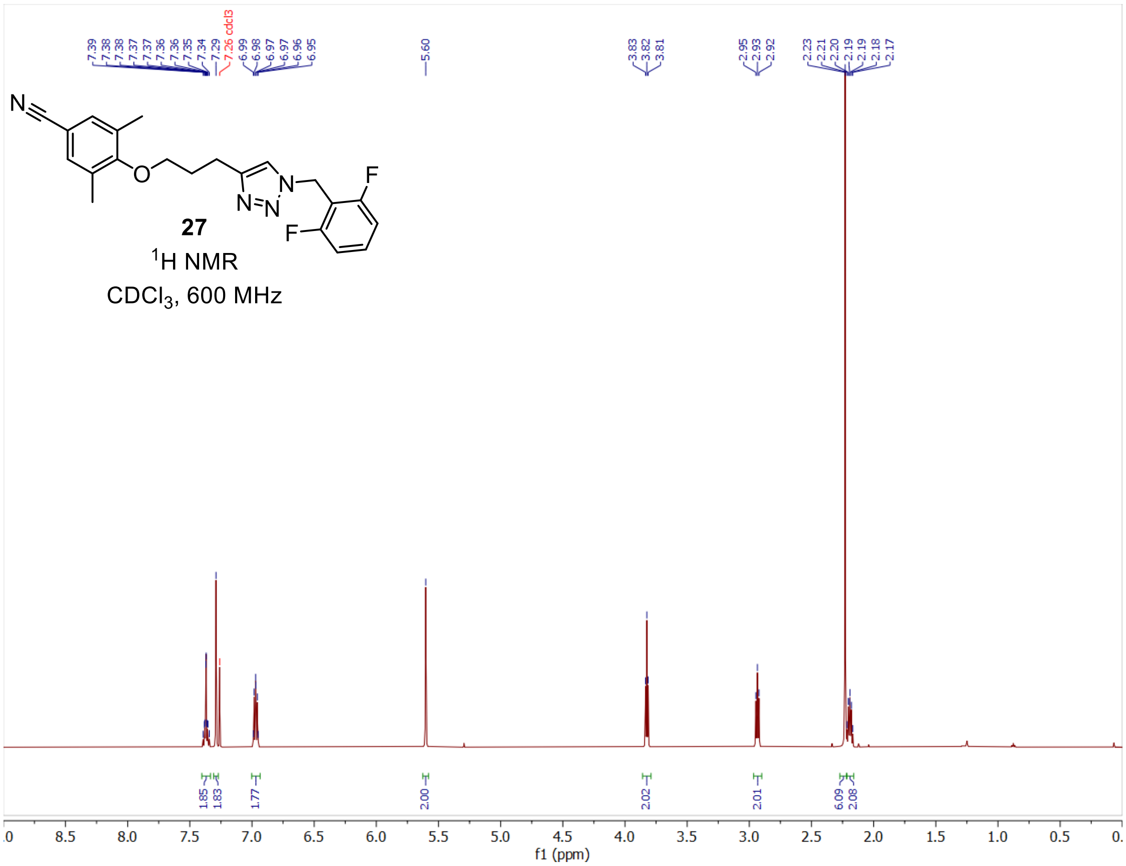
7.39 7.38 7.38 7.37 7.37 7.36 7.36 7.35 7.34 7.29 7.26 cdcl3 6.99 6.98 6.97 6.97 6.96 6.95
5.60
3.83 3.82 3.81
2.95 2.93 2.92
2.23 2.21 2.20 2.19 2.19 2.18 2.17
27
^{1}H NMR
$CDCl_3$, 600 MHz
1.85 1.83 1.77 2.00 2.02 2.01 6.09 2.08
f1 (ppm)

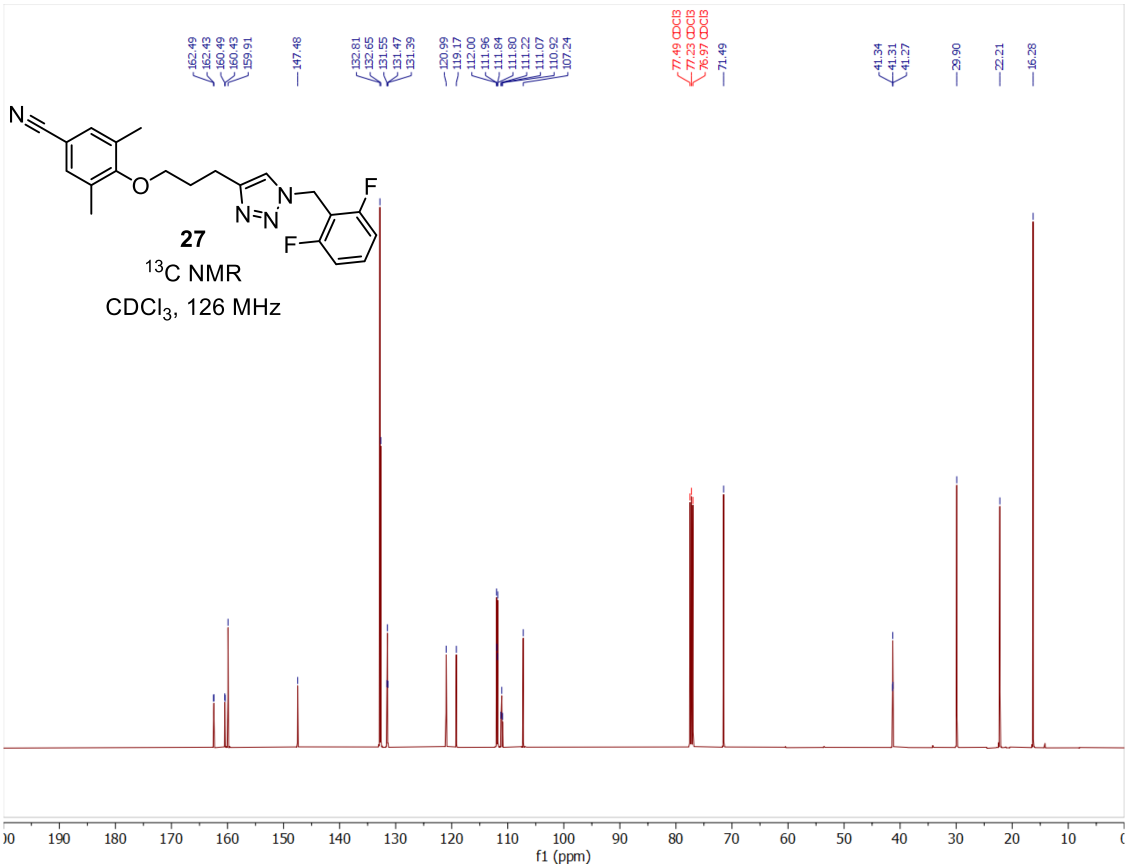
162.49 162.43 160.49 160.43 159.91
147.48
132.81 132.65 131.55 131.47 131.39
120.99 119.17 112.00 111.96 111.84 111.80 111.22 111.07 110.92 107.24
77.49 CDCl3 77.23 CDCl3 76.97 CDCl3
71.49
41.34 41.31 41.27
29.90
22.21
16.28
27
^{13}C NMR
$CDCl_3$, 126 MHz
f1 (ppm)

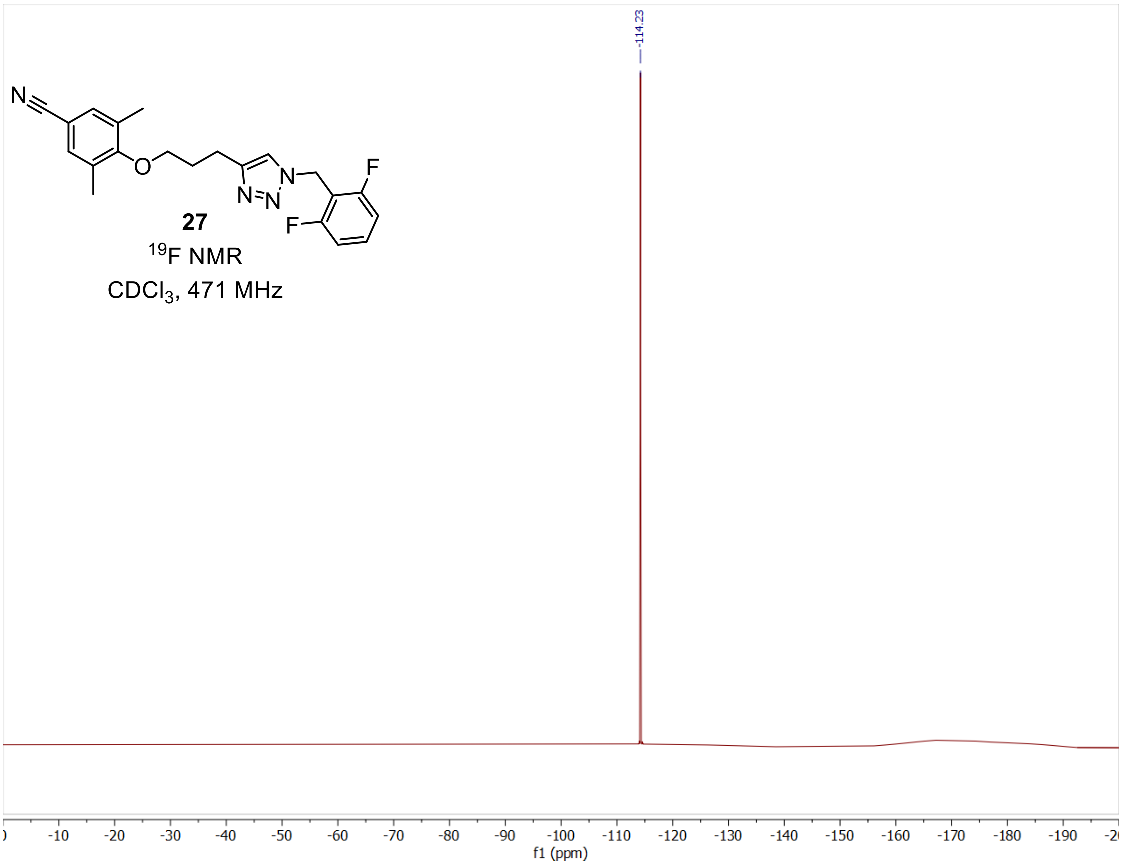

-114.23
N
O
N
N
N
F
F
27
19F NMR
CDCl3, 471 MHz
0
-10
-20
-30
-40
-50
-60
-70
-80
-90
-100
-110
-120
-130
-140
-150
-160
-170
-180
-190
f1 (ppm)

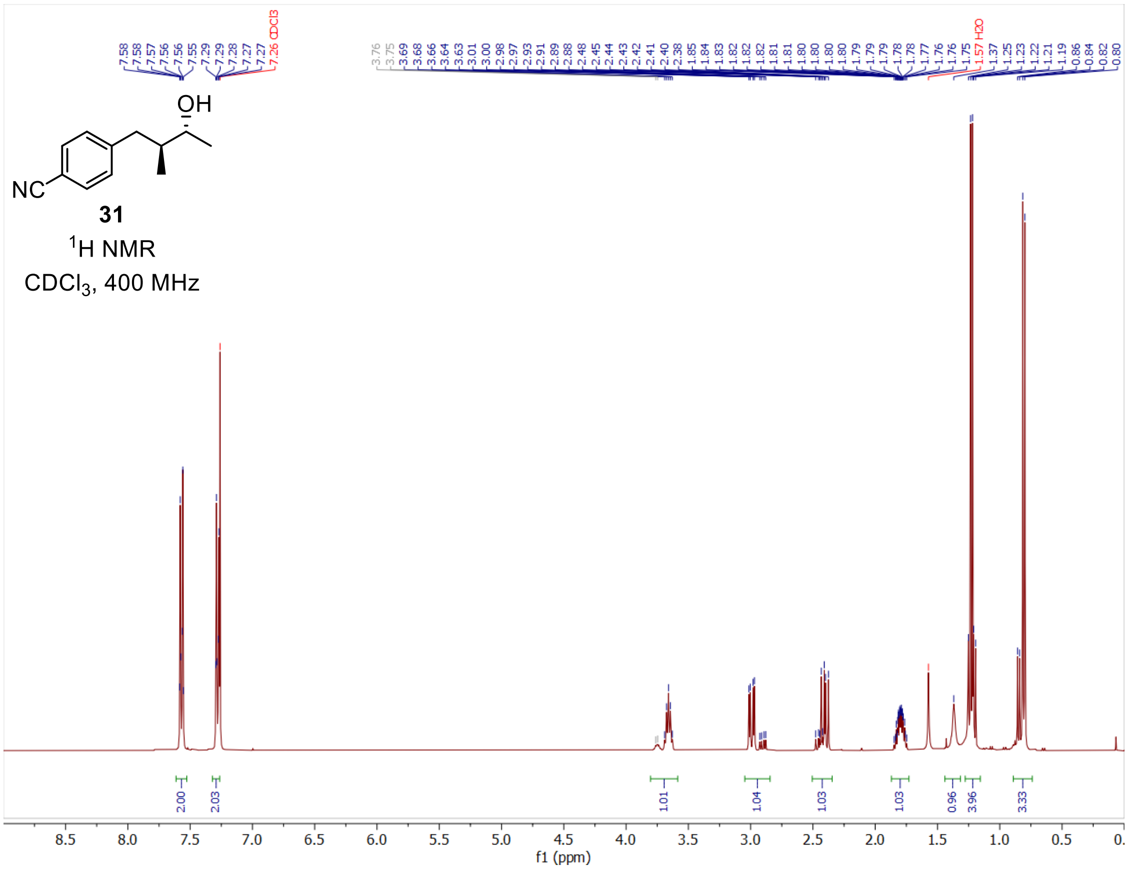
OH
NC
31
1H NMR
CDCl3, 400 MHz
f1 (ppm)

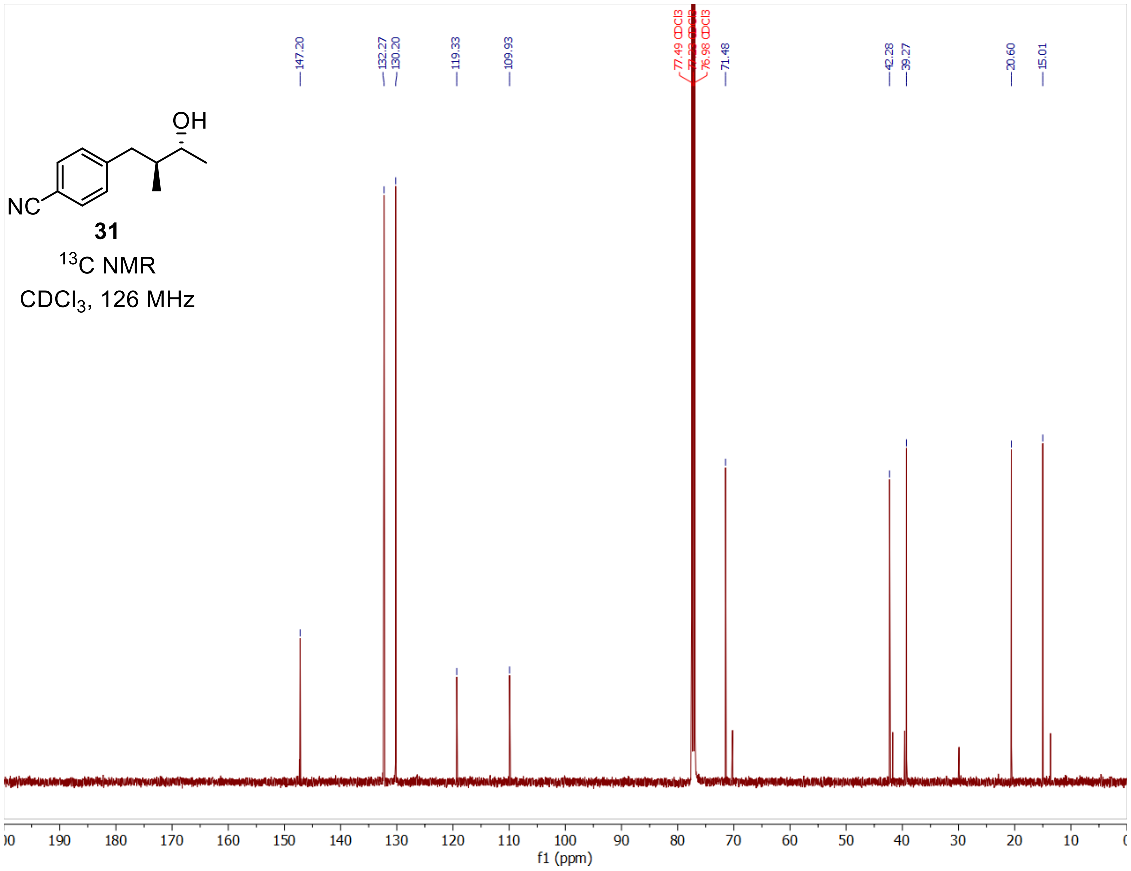
OH
NC
31
13C NMR
CDCl3, 126 MHz
147.20
132.27
130.20
119.33
109.93
77.49 CDCl3
76.98 CDCl3
71.48
42.28
39.27
20.60
15.01
f1 (ppm)

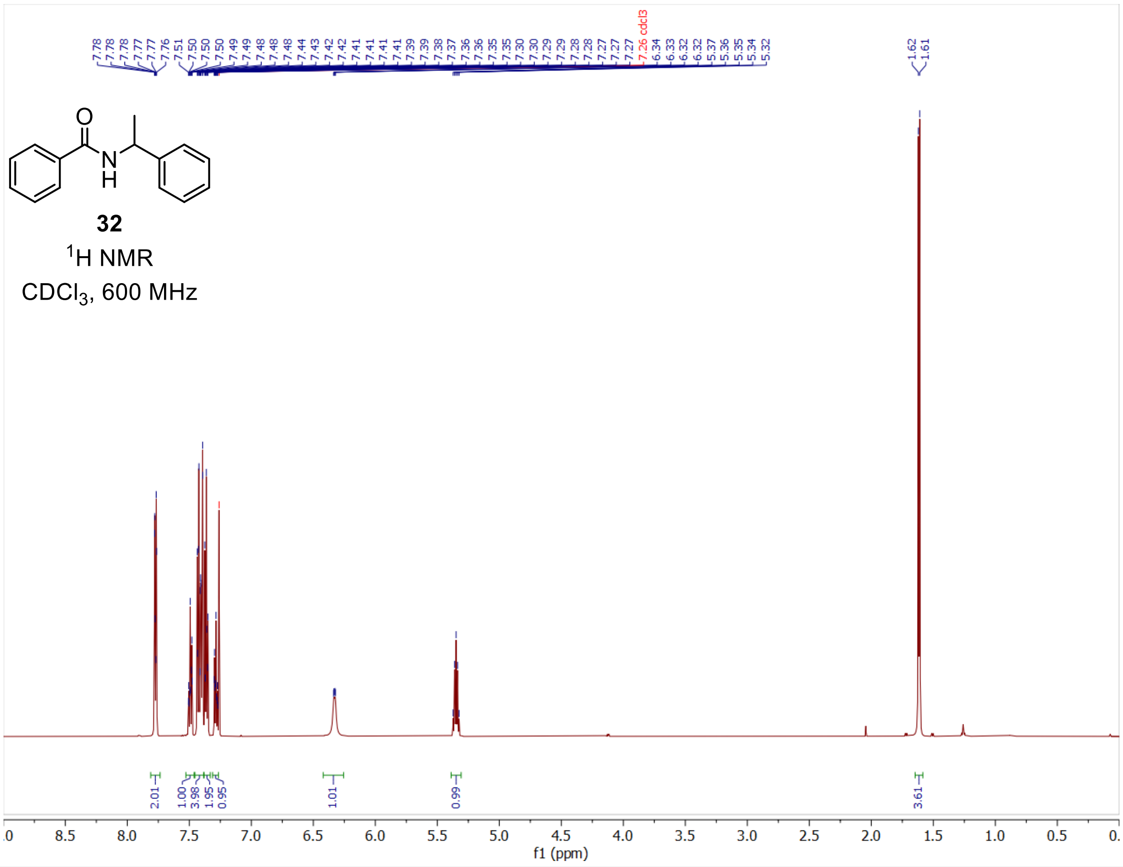
32
^{1}H NMR
$CDCl_3$, 600 MHz
7.26 cdcl3
1.62
1.61
2.01
1.00
3.98
1.95
0.95
1.01
0.99
3.61
f1 (ppm)

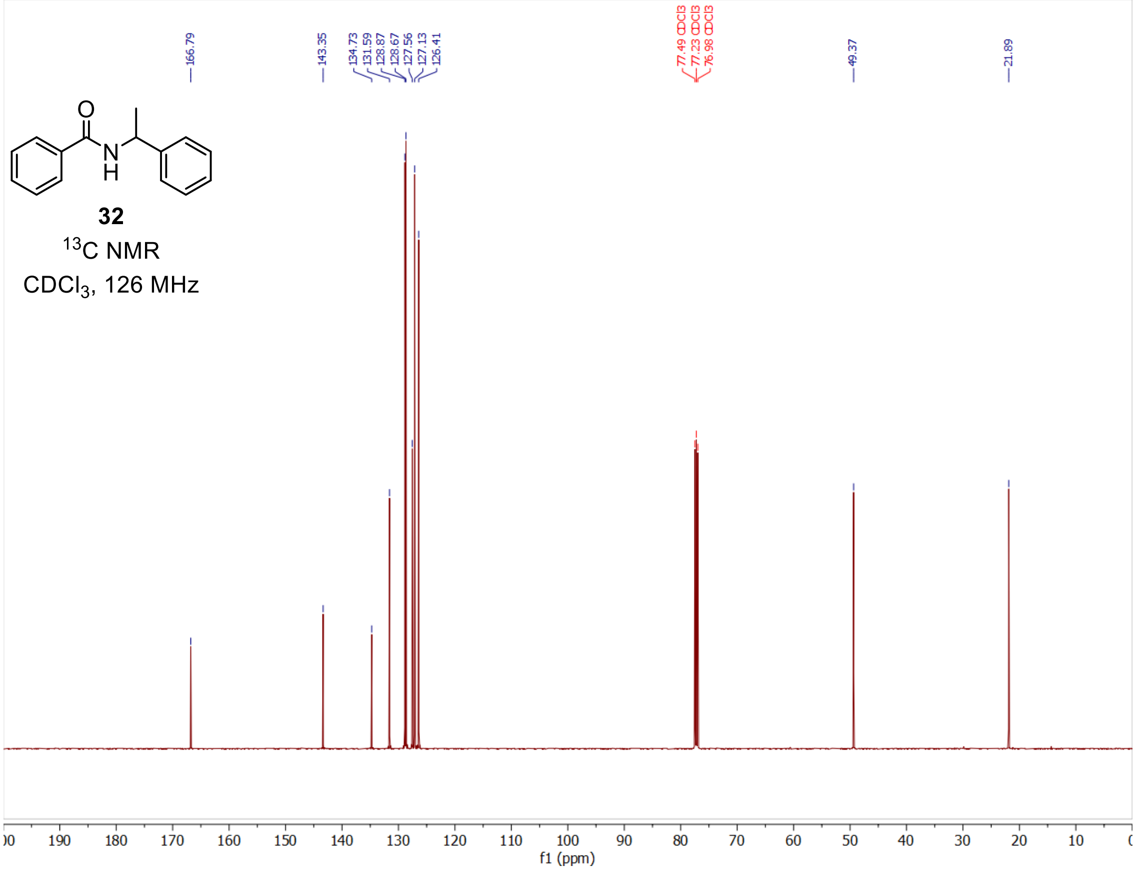
32
^{13}C NMR
$CDCl_3$, 126 MHz
166.79
143.35
134.73
131.59
128.87
128.67
127.56
127.13
126.41
77.49 CDCl3
77.23 CDCl3
76.98 CDCl3
49.37
21.89
f1 (ppm)

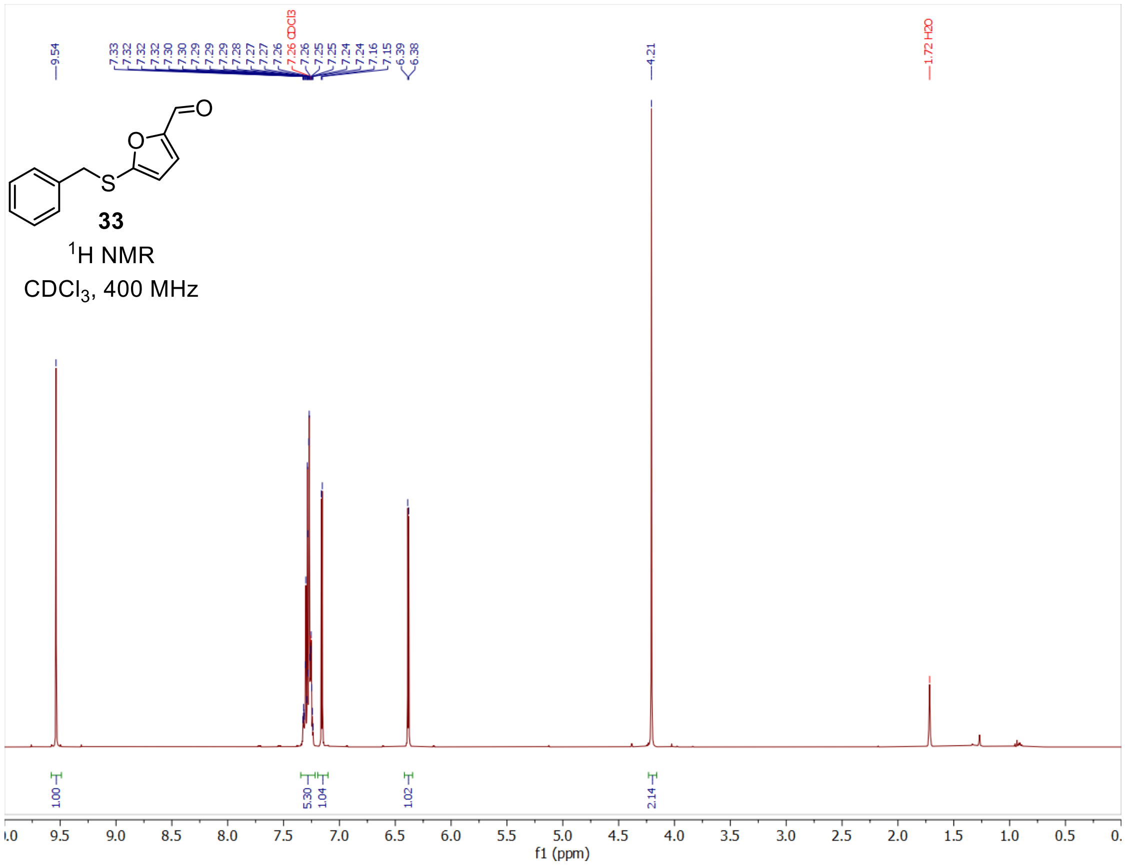
33
1H NMR
CDCl3, 400 MHz
f1 (ppm)

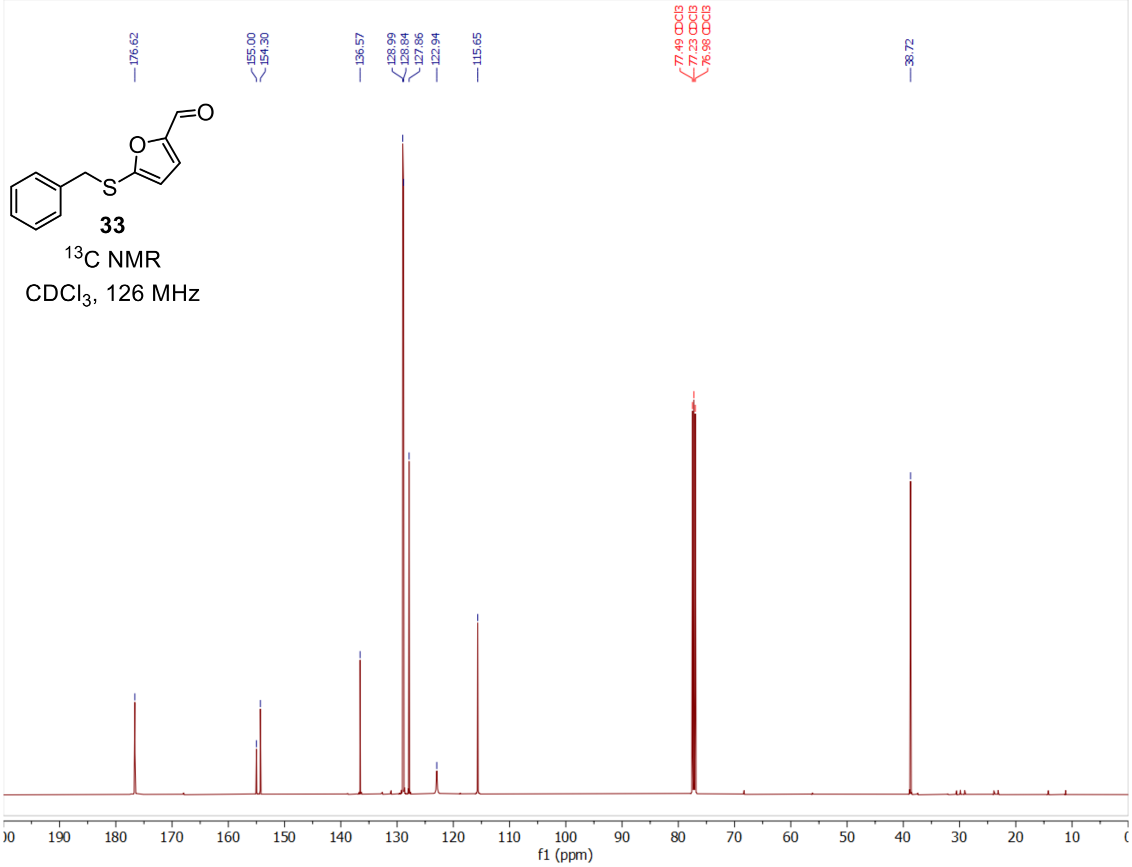
33
13C NMR
CDCl3, 126 MHz
f1 (ppm)
